# Medical student preferences for the internal medicine residency interview day: A cross-sectional study

**DOI:** 10.1371/journal.pone.0199382

**Published:** 2018-07-06

**Authors:** Amar R. Chadaga, Dana Villines, Armand Krikorian

**Affiliations:** 1 Department of Internal Medicine, Advocate Health Care, Oak Lawn, Illinois, United States of America; 2 University of Illinois at Chicago, Department of Medicine, Chicago, Illinois, United States of America; 3 Rosalind Franklin University of Medicine and Science, Department of Medicine, North Chicago, Illinois, United States of America; 4 Department of Administration, Advocate Health Care, Chicago, Illinois, United States of America; Indiana University, UNITED STATES

## Abstract

**Background:**

Applicant recruitment is an essential part of a residency program’s activities with valuable resources dedicated to ensuring its success. Most programs design interview days based on a mix of tradition, budget availability and perception of applicant preferences. There is a paucity of available data on preferences of applicants for interview days.

**Objective:**

We sought to investigate Internal Medicine applicant preferences for a residency recruitment day in aggregate and stratified by medical school background: United States vs. International Medical School Graduate.

**Methods:**

A survey was developed and used in a cross-sectional study of Internal Medicine categorical and preliminary medicine candidates. Applicants ranked different facets of the interview day using a Likert scale. Variables included interview type, start time, length of interview day, number of interviews, length of each interview, background of interviewers, types of questions, interaction time with residents, month of interview, and components of interview day.

**Results:**

265 applicants received the surveys and 215 completed them correctly (81%). Overall, applicants tended to favor an 8–9 am start time (81.9%) and an optimal duration of four hours (82.8%). The interview was the most preferred component of the day (80.0%) with one-on-one (98.1%) and 15–30 min (95.3%) interviews preferred. Several statistically significant differences were found between the United States and International students as well as Categorical and Preliminary applicants.

**Conclusion:**

Our findings offer insights into various factors of the interview day that may appeal to Internal Medicine candidates. This information will be useful to graduate medical education departments engaged in recruitment.

## Introduction

Each year, thousands of medical students and recent graduates interview for Internal Medicine categorical and preliminary first-year positions in the United States (U.S.). According to the National Resident Matching Program (NRMP), there were 6,770 Internal Medicine positions offered in 2015, with 49% filled by U.S. medical school graduates and the rest by International Medical Graduates (IMGs).[1] Despite a tremendous amount of material resources and time dedicated by programs and applicants to recruitment, little data is available on applicant preferences for an ‘ideal’ recruitment day experience. While the ideal family practice[2], emergency medicine[3], urology[4], and radiology[5] interview days have been described in the literature, few studies have examined the components favored by different Internal Medicine applicants[6]. The purpose of our study was to conduct a survey of U.S. medical school (both categorical and preliminary) and IMG candidates applying for a post graduate year one (PGY-1) position in Internal Medicine to determine their preferences toward various elements of the interview day.

## Methods

We created a paper-based survey exploring 10 facets of the interview day: start time, interview type, interview components, number of interviews, length of each interview, types of questions asked, length of interview day, month of interview, interaction time with residents, and background of interviewers. Responses were based on a five point Likert-type scale, where 1 was most preferred and 5 was the least. Three yes/no questions followed regarding candidates preferences towards a pre-interview dinner, a hospital tour and an exit interview.

The population for our study consisted of all candidates who interviewed for Internal Medicine at the University of Illinois at Chicago/Advocate Christ Medical Center program in Oak Lawn, Illinois between November 3, 2014 and January 14, 2015 (205 categorical and 60 preliminary). They were selected from a pool of over 3,000 ERAS applicants for 22 categorical and 6 preliminary positions offered in the 2015 NRMP match.

Candidates completed anonymously a paper survey and deposited it in a sealed collection box. Oral instructions described the purpose of the study and stressed that participation was voluntary, the collection box would not be opened until after the interview season, and that there would be no impact on prospects in the NRMP Match.

Survey responses were dichotomized for analysis to represent either “preferred” for responses ranked as 1 or 2 or “not preferred” for responses ranked as 4 or 5. Responses ranked as a 3 were considered neutral for this analysis and therefore were not included in the data presentation. Between-group analysis was performed using Chi-square analysis and analysis groups were as follows: Categorical (US + IMG) versus Preliminary and US categorical versus IMG categorical. Data was analyzed using SPSS 22 (IBM SPSS Statistics for Windows, Version 22.0. Armonk, NY: IBM Corp) and statistical significance was determined at p ≤ 0.05.

On 1.16.14, the Advocate Health Care Institutional Review Board reviewed the protocol and determined that the proposed activity is not research involving human subjects as defined by DHHS and FDA regulations as this study is designed as a local QA/QI activity specific to the site(s) characteristics, with a limited population and is therefore not generalizable. IRB review and approval by this organization is not required.

## Results

### Sample

Of the 265 distributed questionnaires, 81% were filled out completely and 17% included omissions. Two percent of candidates declined to participate in the study with a final response rate for analysis of 81% (*n* = 215). Interviewee cohorts included categorical Internal Medicine U.S. medical school applicants (*n* = 96, 44.7%), categorical Internal Medicine international medical school applicants (*n* = 65, 30.2%), and preliminary Internal Medicine U.S. medical school applicants (*n* = 54, 25.1%). The proportion of incomplete surveys differed significantly between categorical U.S. medical school applicants and categorical international applicants (incomplete: 12% vs. 32%; error: 12% vs. 29%, p < 0.01 for both comparisons). Further demographic data was not obtained to maintain subject anonymity.

### Overall preferences

**Fig 1:**
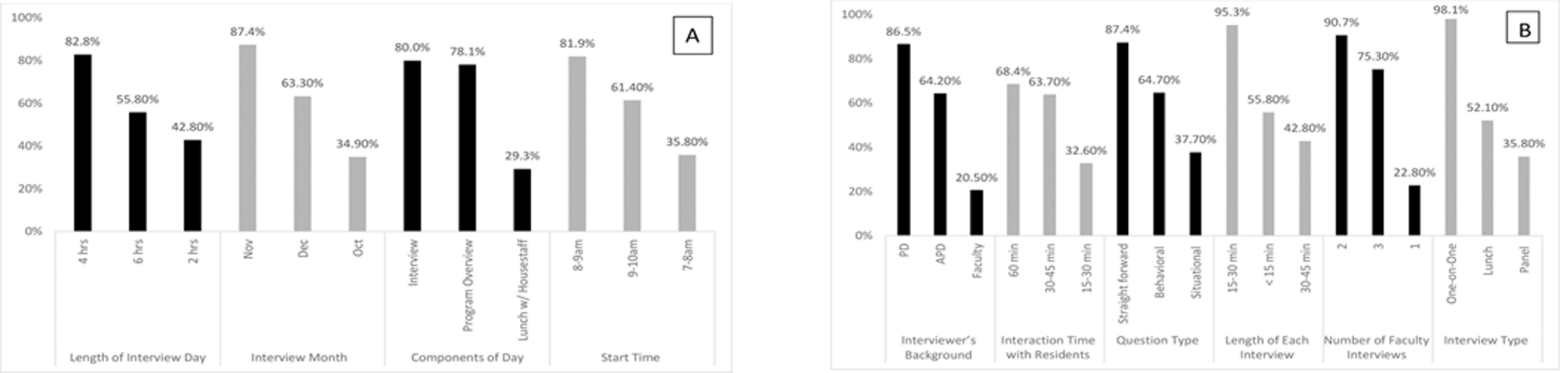
**A-B: Percentages for Most Preferred Responses for Total Sample on ‘Optimal Interview Day Format’ Questions: (A) Structure of Day and (B) Interview Format**.

Fig 1 summarizes the “optimal interview format” responses for the 10 facets of the interview day. Overall, the preferred start time was 8–9 am (81.9%) and the actual interview was the most favored component of the day (80.0%) with one-on-one interviews preferred by almost all applicants (98.1%). The optimal number of faculty interviews was two (90.7%) with each interview preferably lasting 15–30 minutes (95.3%) and consisting of straightforward questions (87.4%). Applicants would rather the interview day occur in November (87.4%), last for one-half day (four hours, 82.8%) and include 45–60 minutes of interaction with residents (68.4%). They were also more partial to interviewing with the program director (86.5%).

### Preference differences for US and IMG categorical versus preliminary applicants

Table 1 summarizes the responses with US and IMG categorical applicants combined and in comparison to preliminary applicants for the 10 facets of the interview day. The preferred categories were similar to the overall findings reported in Fig 1.

**Table 1:** Preference for categorical (US +IMG) versus preliminary applicants.

Question	Response Options									
	C	P	C	P	C	P	C	P	C	P
**Start Time**	7AM-8AM		8AM-9AM		9AM-10AM		10AM-11am		11am-noon	
	50.8%	40.5%	86.2%	91.8%	94.9%	95.0%	21.3%	20.0%	6.0%	4.0%
**Components of Interview Day**	Program Overview		Interviews		Tour		Morning Report		Lunch w/ Housestaff	
	88.7%	95.5%	90.1%	95.7%	4.8%	2.1%	14.4%*	2.0%	41.7%*	69.0%
**Interview Type**	Phone Interviews		Lunch Interviews		One-on-One Interviews		Panel Interviews‡		Group Interviews‡‡	
	7.0%**	36.1%	78.2%	74.3%	97.5%	100.0%	56.4%*	34.1%	5.9%	0.0%
**Number of Faculty Interviews with**	One Interview		Two Interviews		Three Interviews		Four Interviews		>Four Interviews	
	36.1%	37.8%	94.2%	96.2%	98.4%	100.0%	13.4%	11.4%	3.2%	0.0%
**Length of Each Interview**	< 15 minutes		15–30 minutes		30–45 minutes		45–60 minutes		>60 minutes	
	69.4%**	97.7%	96.8%	100.0%	98.8%	100.0%	6.7%*	0.0%	3.1%	0.0%
**Question Type**	Straightforward questions		Behavioral questions		Situational questions		Brainteasers		Medical questions	
	90.6%	98.1%	93.8%	87.2%	79.5%*	57.7%	8.9%	13.0%	1.9%	0.0%
**Interview Month**	October		November		December		January		February	
	41.8%	58.5%	96.7%*	87.2%	97.3%**	85.3%	13.9%	26.2%	1.9%	5.8%
**Length of Interview Day**	< 2 hrs		¼ day (~2 hrs)		½ day (~4 hrs)		¾ day (~6 hrs)		Full day (~8 hrs)	
	7.9%**	27.8%	50.9%**	95.0%	97.1%	100.0%	79.4%**	36.4%	14.1%*	1.9%
**Interaction Time with Residents**	< 15 minutes		15–30 minutes		30–45 minutes		60 minutes		>60 minutes	
	6.0%	8.3%	34.8%	50.0%	100.0%	100.0%	84.4%	76.7%	42.6%*	23.3%
**Interviewer’s Background**	Residents & Chief Residents		Faculty		Assoc. Program Directors		Program Director		Chairman	
	16.4%	11.9%	29.5%	33.3%	89.4%	88.1%	94.0%	97.9%	19.7%	15.2%

‡ Panel interview represents one applicant & more than one interviewer

‡‡ Group interview represents one interviewer & more than one applicant

* p < 0.05

** p < 0.01

Between-group differences were reported in components of the interview day with more categorical than preliminary applicants preferring to attend morning report (14.4% vs 2.0%, p < 0.05) and more preliminary than categorical applicants preferring lunch with house staff (41.7% vs 69.0%, p < 0.05). Although all applicants preferred one-on-one interviews, differences were found for other interview formats. Phone interviews were preferred by preliminary applicants (36.1% vs 7.0%, p < 0.01) while panel interviews were favored by categoricals (56.4% vs 34.1%, p < 0.05). Almost all preliminary applicants preferred interviews lasting < 15 minutes (97.7% vs 69.4%, p < 0.01). They also preferred shorter interview days (p < 0.01 for < 2 hours and 2 hours) while categoricals preferred longer time frames (p < 0.01 for 6 hours and < 0.05 for 8 hours). A similar pattern was reported for longer interaction time with residents with categoricals favoring > 60 minutes more than preliminary (42.6% vs 23.3%, p < 0.05).

Although there was a statistically significant difference between November and December, more than 85% of each group preferred to interview during these months. There were no statistically significant between-group differences for start time, number of faculty interviews and interviewer’s background.

### Preference differences for US categorical versus IMG categorical applicants

Table 2 summarizes the responses of US versus IMG categorical applicants for the 10 facets of the interview day. The preferred categories were similar to the overall findings in Fig 1.

**Table 2:** Preference for US vs IMG applicants.

Question	Response Options									
	US	IMG	US	IMG	US	IMG	US	IMG	US	IMG
**Start Time**	7AM-8AM		8AM-9AM		9AM-10AM		10AM-11am		11am-noon	
	58.1%*	39.6%	91.3%*	78.3%	92.7%	97.7%	15.1%*	30.6%	3.3%	10.2%
**Components of Interview Day**	Program Overview		Interviews		Tour		Morning Report		Lunch w/ Housestaff	
	89.7%	87.5%	85.9%*	96.5%	4.9%	4.5%	15.2%	13.2%	55.0%**	23.3%
**Interview Type**	Phone Interviews		Lunch Interviews		One-on-One Interviews		Panel Interviews‡		Group Interviews‡‡	
	4.2%	10.7%	90.8%**	60.0%	97.9%	96.9%	49.3%	67.4%	3.6%	9.8%
**Number of Faculty Interviews with**	One Interview		Two Interviews		Three Interviews		Four Interviews		>Four Interviews	
	34.0%	38.6%	95.7%	91.7%	97.4%	100.0%	9.2%	19.1%	3.2%	3.2%
**Length of Each Interview**	< 15 minutes		15–30 minutes		30–45 minutes		45–60 minutes		>60 minutes	
	55.7%**	86.0%	97.8%	95.3%	98.4%	100.0%	8.1%	5.0%	2.1%	4.6%
**Question Type**	Straightforward questions		Behavioral questions		Situational questions		Brainteasers		Medical questions	
	95.7%**	82.1%	97.0%	89.1%	80.0%	79.1%	6.5%	13.2%	1.1%	3.2%
**Interview Month**	October		November		December		January		February	
	43.7%	39.2%	97.9%	94.8%	98.4%	95.7%	6.8%*	23.8%	2.1%	1.6%
**Length of Interview Day**	< 2 hrs		¼ day (~2 hrs)		½ day (~4 hrs)		¾ day (~6 hrs)		Full day (~8 hrs)	
	3.8%*	13.3%	53.2%	47.7%	97.6%	96.3%	78.8%	80.4%	13.0%	15.7%
**Interaction Time with Residents**	< 15 minutes		15–30 minutes		30–45 minutes		60 minutes		>60 minutes	
	1.1%**	13.3%	31.8%	39.6%	100.0%	100.0%	90.8%*	76.3%	50.0%*	31.3%
**Interviewer’s Background**	Residents & Chief Residents		Faculty		Assoc. Program Directors		Program Director		Chairman	
	21.7%*	8.8%	40.4%**	16.7%	93.1%	82.9%	91.9%	96.8%	5.8%**	43.1%

‡ Panel interview represents one applicant & more than one interviewer

‡‡ Group interview represents one interviewer & more than one applicant

* p < 0.05

** p < 0.01

More US applicants than IMGs preferred the interview day to start between 7am-9am (for 7am-8am: 58.1% vs 39.6%, p < 0.05; for 8am-9am: 91.3% vs 78.3%, p < 0.05) while twice as many IMG than US applicants preferred a later (10am-11am) start (30.6% vs 15.1%, p < 0.05). Alternatively, more than twice as many US than IMG applicants preferred to have lunch with the house staff (55.0% vs. 23.3%, p < 0.01) or lunch interviews (90.8% vs 60.0%, p < 0.01).

IMGs preferred shorter (< 15 minutes) interviews (55.7% vs 86.0%, p < 0.01), shorter (<2 hours) interview days (13.3% vs 3.8%, p < 0.05) and brief (< 15 minutes) interactions with residents (13.3% vs 1.1%, p < 0.05). Conversely, US applicants preferred more interaction with residents than IMGs (45–60 minutes: 90.8% vs 76.3%, p < 0.05; > 60 minutes: 50.0% vs 31.3%, p < 0.05) and more US candidates would rather interview with residents/chief residents than IMG (21.7% vs 8.8%, p < 0.05). Faculty interviews were more preferred by US applicants than IMG (40.4% vs 16.7%, p < 0.05) while more IMGs preferred to interview with the Chairperson than US (43.1% vs 5.8%, p < 0.05).

Although there was a statistically significant difference among the interview components of the day, more than 85% of each group rated the interviews as the most preferred component of the interview day with no statistically significant between-group differences for the number of faculty interviews.

### Preferences for pre-interview dinners, tours, and necessity of exit interviews

Overall, 56.3% of applicants preferred a pre-interview dinner, a trend significantly more prominent among both US and IMG categoricals (65.6% and 63.1%) compared to preliminary (31.5%, p<0.01), while only a third of respondents preferred the tour being optional (34.4%). Conversely, preliminary applicants preferred the tour to be optional (57.4%) while US and IMG categorical applicants did not (30.2% and 21.5%; p < 0.01). 55.3% of applicants thought an exit interview was unnecessary, with IMGs the only group to consider the exit interview as a requisite component of the day (70.8% versus 40.6% for US and 20.4% for preliminary applicants; p < 0.01).

## Discussion

Residency programs navigate several challenges in organizing interview days, including restricted budgets, complex scheduling and limited faculty availability. Similarly, applicants endure the hardship of a lengthy interview season with possible financial and psychological stressors. While several metrics are considered by both applicants and programs during the selection process, the interview day remains a key component. There has been a paucity of studies examining applicants’ preferences regarding the optimal interview day. To our knowledge, our study is the first to explore differences between categorical and preliminary candidates as well as United States Medical School Graduate vs. International Medical Student Graduates.

Overall, Internal Medicine residency applicants preferred an interview day lasting ~ 4 hours (½ day) with start time between 8am-9am, favored the interviews over other components of the day, and would rather have two, one-on-one interviews lasting 15–30 minutes with straightforward questions. Applicants also favored interviewing with the Program Director over other physicians. A slight majority of applicants favored a pre-interview dinner, preferred the tour be part of the interview day, and viewed the exit interview as unnecessary.

In previous studies, Jacobs et al. indicated that urology applicants preferred 5–7 faculty interviews[4] while others found that 2 faculty interviews of 20–30 minutes were ideal for Family Medicine and Internal Medicine applicants[2], [6], potentially highlighting differences between nonsurgical and surgical interview processes. Schneeweiss et al. also found interviews and informal meetings with residents were regarded as most helpful to applicants, followed by interviews with the program director or faculty.[2]

Unique to our study was the stratification of applicants based on medical school background (US vs IMG) and preliminary year in Internal Medicine vs categorical. Nearly all preliminary applicants preferred shorter overall interview days, with interviews that were ≤ 15 minutes, less interaction with housestaff and no pre-interview dinners. These differences, while not surprising given that preliminary candidates may focus more on the interview for their advanced specialty of choice, could have important budgetary ramifications for program directors and coordinators planning interview days for various constituencies.

When comparing US versus IMG candidates, IMGs preferred shorter interviews compared to US applicants and most intriguing was the dichotomy in how each cohort viewed certain interviewers: Faculty interviews were preferred by US graduates while more IMGs preferred to interview with the Chairperson. This may be explained by cultural difference as IMGs could place more importance on seniority whereas US applicants preferred ‘boots on the ground’ interviewers.

Certain limitations should be considered in interpreting our results. While our questionnaire was based on surveys from previously published studies, it would have benefited from cross-cultural validation as evidenced by the statistically significant number of IMG respondents not completing the survey. The study was conducted at a single community-based, university-sponsored Internal Medicine program near a major metropolitan city and may not be applicable to other institutions or locations. It is also limited by common caveats inherent to survey designs and self-report: Cross-sectional data limits the conclusions that can be drawn about causal sequence while subjective self-reporting may not correlate to the actual preferences of candidates. Further, qualitative data was not obtained which could have identified unique aspects of the interview day that we neglected to explore. Future directions consist of including qualitative questions as well as expanding this study to other institutions to investigate if results remain similar across different types of internal medicine residency programs.

As a follow-up, our program overhauled the interview day during the 2015–2016 interview season. For categorical applicants, the start time was changed to 8am, interviews were shortened from 30 minutes to 10–20 minutes and conducted only during the months of November and December. The day was truncated from roughly 6 hours to 4 hours, exit interviews eliminated, and all candidates interviewed with the program director. In addition, lunch was eliminated and the tour made optional for preliminary applicants. In a quality improvement survey, applicants’ response was overwhelmingly positive: 100% approved of the interview month, 99% were in favor of the interviewer’s background, 96% endorsed the overall length of the day, 95% thought the 8am start time was just right, and 83% supported the length of the interviews.

## Conclusions

Our findings offer insight into crucial factors of the interview day that may appeal to Internal Medicine residency candidates. While residency programs are restricted by program specific constraints in conducting interviews, this information will be useful to further tailor recruitment in an ‘applicant-centric’ manner.

## Supporting information

S1 File Appendix S1: Contains questionnaire used in this study. (DOCX)

S2 File Appendix S2: Contains the minimal data set used in this study. (DOC)
