# Supplementary material for: Medical student preferences for the internal medicine residency interview day: A cross-sectional study
Source: PLoS One. 2018 Jul 6;13(7):e0199382. doi: 10.1371/journal.pone.0199382 (PMC6034786; doi:10.1371/journal.pone.0199382)
Supplement: S1 File — (DOCX) [file pone.0199382.s001.docx]

**Survey of Medical Student Preferences on Residency Interview Day**

This is an anonymous quality improvement survey. Participation is voluntary, but greatly appreciated.
Data may be used for future research purposes.

Participation will have no impact on future prospects at the UIC-Advocate Christ Internal Medicine Residency Program.

**When you are finished with your survey, please leave in designated collection box in the 131NOB Conference Room The collection box will not be opened until after the recruitment season is over (end of January 2015).*

**For the following, please rank your preference, with 1 being most preferred and 5 being least preferred.**

|  | Phone Interviews | Lunch Interviews | One-on-One Interviews | Panel Interviews (i.e. – one applicant & more than one interviewer) | Group Interviews (i.e. – one interviewer & more than one applicant) |
| --- | --- | --- | --- | --- | --- |
| 1.Interview Type |  |  |  |  |  |
|  | 7AM-8AM | 8AM-9AM | 9AM-10AM | 10AM-11am | 11am-noon |
| 2.Start Time |  |  |  |  |  |
|  | < 2 hours | ¼ day (~2 hours) | ½ day (~4 hours) | ¾ day (~6 hours) | Full day (~8 hours) |
| 3.Length of Interview Day |  |  |  |  |  |
|  | One Interview | Two Interviews | Three Interviews | Four Interviews | >Four Interviews |
| 4.Number of Interviews with Faculty |  |  |  |  |  |
|  | < 15 minutes | 15-30 minutes | 30-45 minutes | - 1. minutes | >60 minutes |
| 5.Length of Each Interview |  |  |  |  |  |
|  | Residents & Chief Residents | Faculty | Associate Program Directors | Program Director | Chairman |
| 6.Interviewers Background |  |  |  |  |  |
|  | Straightforward questions | Behavioral questions | Situational questions | Brainteasers | Medical questions |
| 7.Types of Questions |  |  |  |  |  |
|  | < 15 minutes | 15-30 minutes | 30-45 minutes | - 1. minutes | >60 minutes |
| 8.Interaction Time with Residents |  |  |  |  |  |
|  | October | November | December | January | February |
| 9. Month of Interview |  |  |  |  |  |

**For the following, please rank the importance, with 1 being most important and 5 being least important.**

|  | Program Overview | Interviews | Tour | Morning Report | Lunch (Interaction) w/ Housestaff |
| --- | --- | --- | --- | --- | --- |
| 10. Components of the Interview Day |  |  |  |  |  |

**For the following, please circle one answer choice per question.**

1. I prefer a pre-interview day dinner
   - Yes
   - No
2. I prefer the tour be optional
   1. Yes
   2. No
3. The exit interview is
   1. Necessary
   2. Unnecessary
