# Supplementary material for: Medical student preferences for the internal medicine residency interview day: A cross-sectional study
Source: PLoS One. 2018 Jul 6;13(7):e0199382. doi: 10.1371/journal.pone.0199382 (PMC6034786; doi:10.1371/journal.pone.0199382)
Supplement: S2 File — (DOC) [file pone.0199382.s002.doc]

DESCRIPTIVES

| **candidate** | | | | | |
| --- | --- | --- | --- | --- | --- |
|  | | Frequency | Percent | Valid Percent | Cumulative Percent |
| Valid | US categorical | 96 | 44.7 | 44.7 | 44.7 |
|  | IM categorical | 65 | 30.2 | 30.2 | 74.9 |
|  | Preliminary | 54 | 25.1 | 25.1 | 100.0 |
|  | Total | 215 | 100.0 | 100.0 |  |

| **Cat_prelim** | | | | | |
| --- | --- | --- | --- | --- | --- |
|  | | Frequency | Percent | Valid Percent | Cumulative Percent |
| Valid | categorical | 161 | 74.9 | 74.9 | 74.9 |
|  | preliminary | 54 | 25.1 | 25.1 | 100.0 |
|  | Total | 215 | 100.0 | 100.0 |  |

| **INT_phone** | | | | | |
| --- | --- | --- | --- | --- | --- |
|  | | Frequency | Percent | Valid Percent | Cumulative Percent |
| Valid | 1 | 4 | 1.9 | 1.9 | 1.9 |
|  | 2 | 18 | 8.4 | 8.4 | 10.2 |
|  | 3 | 51 | 23.7 | 23.7 | 34.0 |
|  | 4 | 58 | 27.0 | 27.0 | 60.9 |
|  | 5 | 84 | 39.1 | 39.1 | 100.0 |
|  | Total | 215 | 100.0 | 100.0 |  |

| **INT_lunch** | | | | | |
| --- | --- | --- | --- | --- | --- |
|  | | Frequency | Percent | Valid Percent | Cumulative Percent |
| Valid | 1 | 1 | .5 | .5 | .5 |
|  | 2 | 111 | 51.6 | 51.6 | 52.1 |
|  | 3 | 70 | 32.6 | 32.6 | 84.7 |
|  | 4 | 29 | 13.5 | 13.5 | 98.1 |
|  | 5 | 4 | 1.9 | 1.9 | 100.0 |
|  | Total | 215 | 100.0 | 100.0 |  |

| **INT_one_on_one** | | | | | |
| --- | --- | --- | --- | --- | --- |
|  | | Frequency | Percent | Valid Percent | Cumulative Percent |
| Valid | 1 | 205 | 95.3 | 95.3 | 95.3 |
|  | 2 | 6 | 2.8 | 2.8 | 98.1 |
|  | 4 | 1 | .5 | .5 | 98.6 |
|  | 5 | 3 | 1.4 | 1.4 | 100.0 |
|  | Total | 215 | 100.0 | 100.0 |  |

| **INT_panel** | | | | | |
| --- | --- | --- | --- | --- | --- |
|  | | Frequency | Percent | Valid Percent | Cumulative Percent |
| 77Valid | 1 | 2 | .9 | .9 | .9 |
|  | 2 | 75 | 34.9 | 34.9 | 35.8 |
|  | 3 | 61 | 28.4 | 28.4 | 64.2 |
|  | 4 | 65 | 30.2 | 30.2 | 94.4 |
|  | 5 | 12 | 5.6 | 5.6 | 100.0 |
|  | Total | 215 | 100.0 | 100.0 |  |

| **INT_group** | | | | | |
| --- | --- | --- | --- | --- | --- |
|  | | Frequency | Percent | Valid Percent | Cumulative Percent |
| Valid | 1 | 3 | 1.4 | 1.4 | 1.4 |
|  | 2 | 5 | 2.3 | 2.3 | 3.7 |
|  | 3 | 33 | 15.3 | 15.3 | 19.1 |
|  | 4 | 62 | 28.8 | 28.8 | 47.9 |
|  | 5 | 112 | 52.1 | 52.1 | 100.0 |
|  | Total | 215 | 100.0 | 100.0 |  |

| **start7_8** | | | | | |
| --- | --- | --- | --- | --- | --- |
|  | | Frequency | Percent | Valid Percent | Cumulative Percent |
| Valid | 1 | 32 | 14.9 | 14.9 | 14.9 |
|  | 2 | 45 | 20.9 | 20.9 | 35.8 |
|  | 3 | 56 | 26.0 | 26.0 | 61.9 |
|  | 4 | 30 | 14.0 | 14.0 | 75.8 |
|  | 5 | 52 | 24.2 | 24.2 | 100.0 |
|  | Total | 215 | 100.0 | 100.0 |  |
| **start8_9** | | | | | |
|  | | Frequency | Percent | Valid Percent | Cumulative Percent |
| Valid | 1 | 115 | 53.5 | 53.5 | 53.5 |
|  | 2 | 61 | 28.4 | 28.4 | 81.9 |
|  | 3 | 14 | 6.5 | 6.5 | 88.4 |
|  | 4 | 21 | 9.8 | 9.8 | 98.1 |
|  | 5 | 4 | 1.9 | 1.9 | 100.0 |
|  | Total | 215 | 100.0 | 100.0 |  |

| **start9_10** | | | | | |
| --- | --- | --- | --- | --- | --- |
|  | | Frequency | Percent | Valid Percent | Cumulative Percent |
| Valid | 1 | 56 | 26.0 | 26.0 | 26.0 |
|  | 2 | 76 | 35.3 | 35.3 | 61.4 |
|  | 3 | 76 | 35.3 | 35.3 | 96.7 |
|  | 4 | 5 | 2.3 | 2.3 | 99.1 |
|  | 5 | 2 | .9 | .9 | 100.0 |
|  | Total | 215 | 100.0 | 100.0 |  |

| **start10_11** | | | | | |
| --- | --- | --- | --- | --- | --- |
|  | | Frequency | Percent | Valid Percent | Cumulative Percent |
| Valid | 1 | 6 | 2.8 | 2.8 | 2.8 |
|  | 2 | 28 | 13.0 | 13.0 | 15.8 |
|  | 3 | 53 | 24.7 | 24.7 | 40.5 |
|  | 4 | 126 | 58.6 | 58.6 | 99.1 |
|  | 5 | 2 | .9 | .9 | 100.0 |
|  | Total | 215 | 100.0 | 100.0 |  |

| **start11_12** | | | | | |
| --- | --- | --- | --- | --- | --- |
|  | | Frequency | Percent | Valid Percent | Cumulative Percent |
| Valid | 1 | 6 | 2.8 | 2.8 | 2.8 |
|  | 2 | 5 | 2.3 | 2.3 | 5.1 |
|  | 3 | 16 | 7.4 | 7.4 | 12.6 |
|  | 4 | 33 | 15.3 | 15.3 | 27.9 |
|  | 5 | 155 | 72.1 | 72.1 | 100.0 |
|  | Total | 215 | 100.0 | 100.0 |  |

| **lengthLT2** | | | | | |
| --- | --- | --- | --- | --- | --- |
|  | | Frequency | Percent | Valid Percent | Cumulative Percent |
| Valid | 1 | 6 | 2.8 | 2.8 | 2.8 |
|  | 2 | 15 | 7.0 | 7.0 | 9.8 |
|  | 3 | 39 | 18.1 | 18.1 | 27.9 |
|  | 4 | 68 | 31.6 | 31.6 | 59.5 |
|  | 5 | 87 | 40.5 | 40.5 | 100.0 |
|  | Total | 215 | 100.0 | 100.0 |  |

| **length2** | | | | | |
| --- | --- | --- | --- | --- | --- |
|  | | Frequency | Percent | Valid Percent | Cumulative Percent |
| Valid | 1 | 31 | 14.4 | 14.4 | 14.4 |
|  | 2 | 61 | 28.4 | 28.4 | 42.8 |
|  | 3 | 69 | 32.1 | 32.1 | 74.9 |
|  | 4 | 54 | 25.1 | 25.1 | 100.0 |
|  | Total | 215 | 100.0 | 100.0 |  |

| **length4** | | | | | |
| --- | --- | --- | --- | --- | --- |
|  | | Frequency | Percent | Valid Percent | Cumulative Percent |
| Valid | 1 | 128 | 59.5 | 59.5 | 59.5 |
|  | 2 | 50 | 23.3 | 23.3 | 82.8 |
|  | 3 | 33 | 15.3 | 15.3 | 98.1 |
|  | 4 | 2 | .9 | .9 | 99.1 |
|  | 5 | 2 | .9 | .9 | 100.0 |
|  | Total | 215 | 100.0 | 100.0 |  |

| **length6** | | | | | |
| --- | --- | --- | --- | --- | --- |
|  | | Frequency | Percent | Valid Percent | Cumulative Percent |
| Valid | 1 | 44 | 20.5 | 20.5 | 20.5 |
|  | 2 | 76 | 35.3 | 35.3 | 55.8 |
|  | 3 | 40 | 18.6 | 18.6 | 74.4 |
|  | 4 | 53 | 24.7 | 24.7 | 99.1 |
|  | 5 | 2 | .9 | .9 | 100.0 |
|  | Total | 215 | 100.0 | 100.0 |  |

| **length8** | | | | | |
| --- | --- | --- | --- | --- | --- |
|  | | Frequency | Percent | Valid Percent | Cumulative Percent |
| Valid | 1 | 6 | 2.8 | 2.8 | 2.8 |
|  | 2 | 13 | 6.0 | 6.0 | 8.8 |
|  | 3 | 34 | 15.8 | 15.8 | 24.7 |
|  | 4 | 38 | 17.7 | 17.7 | 42.3 |
|  | 5 | 124 | 57.7 | 57.7 | 100.0 |
|  | Total | 215 | 100.0 | 100.0 |  |

| **faculty1** | | | | | |
| --- | --- | --- | --- | --- | --- |
|  | | Frequency | Percent | Valid Percent | Cumulative Percent |
| Valid | 1 | 10 | 4.7 | 4.7 | 4.7 |
|  | 2 | 39 | 18.1 | 18.1 | 22.8 |
|  | 3 | 81 | 37.7 | 37.7 | 60.5 |
|  | 4 | 51 | 23.7 | 23.7 | 84.2 |
|  | 5 | 34 | 15.8 | 15.8 | 100.0 |
|  | Total | 215 | 100.0 | 100.0 |  |

| **faculty2** | | | | | |
| --- | --- | --- | --- | --- | --- |
|  | | Frequency | Percent | Valid Percent | Cumulative Percent |
| Valid | 1 | 147 | 68.4 | 68.4 | 68.4 |
|  | 2 | 48 | 22.3 | 22.3 | 90.7 |
|  | 3 | 9 | 4.2 | 4.2 | 94.9 |
|  | 4 | 7 | 3.3 | 3.3 | 98.1 |
|  | 5 | 4 | 1.9 | 1.9 | 100.0 |
|  | Total | 215 | 100.0 | 100.0 |  |

| **faculty3** | | | | | |
| --- | --- | --- | --- | --- | --- |
|  | | Frequency | Percent | Valid Percent | Cumulative Percent |
| Valid | 1 | 50 | 23.3 | 23.3 | 23.3 |
|  | 2 | 112 | 52.1 | 52.1 | 75.3 |
|  | 3 | 51 | 23.7 | 23.7 | 99.1 |
|  | 4 | 2 | .9 | .9 | 100.0 |
|  | Total | 215 | 100.0 | 100.0 |  |

| **faculty4** | | | | | |
| --- | --- | --- | --- | --- | --- |
|  | | Frequency | Percent | Valid Percent | Cumulative Percent |
| Valid | 1 | 4 | 1.9 | 1.9 | 1.9 |
|  | 2 | 15 | 7.0 | 7.0 | 8.8 |
|  | 3 | 68 | 31.6 | 31.6 | 40.5 |
|  | 4 | 128 | 59.5 | 59.5 | 100.0 |
|  | Total | 215 | 100.0 | 100.0 |  |

| **facultyGT4** | | | | | |
| --- | --- | --- | --- | --- | --- |
|  | | Frequency | Percent | Valid Percent | Cumulative Percent |
| Valid | 1 | 4 | 1.9 | 1.9 | 1.9 |
|  | 2 | 1 | .5 | .5 | 2.3 |
|  | 3 | 6 | 2.8 | 2.8 | 5.1 |
|  | 4 | 27 | 12.6 | 12.6 | 17.7 |
|  | 5 | 177 | 82.3 | 82.3 | 100.0 |
|  | Total | 215 | 100.0 | 100.0 |  |

| **lengthLT15** | | | | | |
| --- | --- | --- | --- | --- | --- |
|  | | Frequency | Percent | Valid Percent | Cumulative Percent |
| Valid | 1 | 22 | 10.2 | 10.2 | 10.2 |
|  | 2 | 98 | 45.6 | 45.6 | 55.8 |
|  | 3 | 60 | 27.9 | 27.9 | 83.7 |
|  | 4 | 19 | 8.8 | 8.8 | 92.6 |
|  | 5 | 16 | 7.4 | 7.4 | 100.0 |
|  | Total | 215 | 100.0 | 100.0 |  |

| **length15_30** | | | | | |
| --- | --- | --- | --- | --- | --- |
|  | | Frequency | Percent | Valid Percent | Cumulative Percent |
| Valid | 1 | 166 | 77.2 | 77.2 | 77.2 |
|  | 2 | 39 | 18.1 | 18.1 | 95.3 |
|  | 3 | 5 | 2.3 | 2.3 | 97.7 |
|  | 4 | 4 | 1.9 | 1.9 | 99.5 |
|  | 5 | 1 | .5 | .5 | 100.0 |
|  | Total | 215 | 100.0 | 100.0 |  |
| **length30_45** | | | | | |
|  | | Frequency | Percent | Valid Percent | Cumulative Percent |
| Valid | 1 | 21 | 9.8 | 9.8 | 9.8 |
|  | 2 | 71 | 33.0 | 33.0 | 42.8 |
|  | 3 | 122 | 56.7 | 56.7 | 99.5 |
|  | 4 | 1 | .5 | .5 | 100.0 |
|  | Total | 215 | 100.0 | 100.0 |  |

| **length45_60** | | | | | |
| --- | --- | --- | --- | --- | --- |
|  | | Frequency | Percent | Valid Percent | Cumulative Percent |
| Valid | 1 | 3 | 1.4 | 1.4 | 1.4 |
|  | 2 | 6 | 2.8 | 2.8 | 4.2 |
|  | 3 | 28 | 13.0 | 13.0 | 17.2 |
|  | 4 | 178 | 82.8 | 82.8 | 100.0 |
|  | Total | 215 | 100.0 | 100.0 |  |

| **lengthGT60** | | | | | |
| --- | --- | --- | --- | --- | --- |
|  | | Frequency | Percent | Valid Percent | Cumulative Percent |
| Valid | 1 | 4 | 1.9 | 1.9 | 1.9 |
|  | 2 | 1 | .5 | .5 | 2.3 |
|  | 4 | 13 | 6.0 | 6.0 | 8.4 |
|  | 5 | 197 | 91.6 | 91.6 | 100.0 |
|  | Total | 215 | 100.0 | 100.0 |  |

| **INT_res_chief** | | | | | |
| --- | --- | --- | --- | --- | --- |
|  | | Frequency | Percent | Valid Percent | Cumulative Percent |
| Valid | 1 | 7 | 3.3 | 3.3 | 3.3 |
|  | 2 | 21 | 9.8 | 9.8 | 13.0 |
|  | 3 | 33 | 15.3 | 15.3 | 28.4 |
|  | 4 | 71 | 33.0 | 33.0 | 61.4 |
|  | 5 | 83 | 38.6 | 38.6 | 100.0 |
|  | Total | 215 | 100.0 | 100.0 |  |

| **INT_faculty** | | | | | |
| --- | --- | --- | --- | --- | --- |
|  | | Frequency | Percent | Valid Percent | Cumulative Percent |
| Valid | 1 | 27 | 12.6 | 12.6 | 12.6 |
|  | 2 | 17 | 7.9 | 7.9 | 20.5 |
|  | 3 | 71 | 33.0 | 33.0 | 53.5 |
|  | 4 | 86 | 40.0 | 40.0 | 93.5 |
|  | 5 | 14 | 6.5 | 6.5 | 100.0 |
|  | Total | 215 | 100.0 | 100.0 |  |

| **INT_APD** | | | | | |
| --- | --- | --- | --- | --- | --- |
|  | | Frequency | Percent | Valid Percent | Cumulative Percent |
| Valid | 1 | 18 | 8.4 | 8.4 | 8.4 |
|  | 2 | 120 | 55.8 | 55.8 | 64.2 |
|  | 3 | 60 | 27.9 | 27.9 | 92.1 |
|  | 4 | 16 | 7.4 | 7.4 | 99.5 |
|  | 5 | 1 | .5 | .5 | 100.0 |
|  | Total | 215 | 100.0 | 100.0 |  |

| **INT_PD** | | | | | |
| --- | --- | --- | --- | --- | --- |
|  | | Frequency | Percent | Valid Percent | Cumulative Percent |
| Valid | 1 | 156 | 72.6 | 72.6 | 72.6 |
|  | 2 | 30 | 14.0 | 14.0 | 86.5 |
|  | 3 | 19 | 8.8 | 8.8 | 95.3 |
|  | 4 | 6 | 2.8 | 2.8 | 98.1 |
|  | 5 | 4 | 1.9 | 1.9 | 100.0 |
|  | Total | 215 | 100.0 | 100.0 |  |
| **INT_chair** | | | | | |
|  | | Frequency | Percent | Valid Percent | Cumulative Percent |
| Valid | 1 | 7 | 3.3 | 3.3 | 3.3 |
|  | 2 | 27 | 12.6 | 12.6 | 15.8 |
|  | 3 | 32 | 14.9 | 14.9 | 30.7 |
|  | 4 | 36 | 16.7 | 16.7 | 47.4 |
|  | 5 | 113 | 52.6 | 52.6 | 100.0 |
|  | Total | 215 | 100.0 | 100.0 |  |

| **Qstraightforward** | | | | | |
| --- | --- | --- | --- | --- | --- |
|  | | Frequency | Percent | Valid Percent | Cumulative Percent |
| Valid | 1 | 168 | 78.1 | 78.1 | 78.1 |
|  | 2 | 20 | 9.3 | 9.3 | 87.4 |
|  | 3 | 12 | 5.6 | 5.6 | 93.0 |
|  | 4 | 12 | 5.6 | 5.6 | 98.6 |
|  | 5 | 3 | 1.4 | 1.4 | 100.0 |
|  | Total | 215 | 100.0 | 100.0 |  |

| **Qbehavior** | | | | | |
| --- | --- | --- | --- | --- | --- |
|  | | Frequency | Percent | Valid Percent | Cumulative Percent |
| Valid | 1 | 29 | 13.5 | 13.5 | 13.5 |
|  | 2 | 110 | 51.2 | 51.2 | 64.7 |
|  | 3 | 64 | 29.8 | 29.8 | 94.4 |
|  | 4 | 8 | 3.7 | 3.7 | 98.1 |
|  | 5 | 4 | 1.9 | 1.9 | 100.0 |
|  | Total | 215 | 100.0 | 100.0 |  |

| **Qsituation** | | | | | |
| --- | --- | --- | --- | --- | --- |
|  | | Frequency | Percent | Valid Percent | Cumulative Percent |
| Valid | 1 | 11 | 5.1 | 5.1 | 5.1 |
|  | 2 | 70 | 32.6 | 32.6 | 37.7 |
|  | 3 | 106 | 49.3 | 49.3 | 87.0 |
|  | 4 | 22 | 10.2 | 10.2 | 97.2 |
|  | 5 | 6 | 2.8 | 2.8 | 100.0 |
|  | Total | 215 | 100.0 | 100.0 |  |

| **Qteaser** | | | | | |
| --- | --- | --- | --- | --- | --- |
|  | | Frequency | Percent | Valid Percent | Cumulative Percent |
| Valid | 1 | 4 | 1.9 | 1.9 | 1.9 |
|  | 2 | 15 | 7.0 | 7.0 | 8.8 |
|  | 3 | 23 | 10.7 | 10.7 | 19.5 |
|  | 4 | 124 | 57.7 | 57.7 | 77.2 |
|  | 5 | 49 | 22.8 | 22.8 | 100.0 |
|  | Total | 215 | 100.0 | 100.0 |  |

| **Qmedical** | | | | | |
| --- | --- | --- | --- | --- | --- |
|  | | Frequency | Percent | Valid Percent | Cumulative Percent |
| Valid | 1 | 3 | 1.4 | 1.4 | 1.4 |
|  | 3 | 10 | 4.7 | 4.7 | 6.0 |
|  | 4 | 49 | 22.8 | 22.8 | 28.8 |
|  | 5 | 153 | 71.2 | 71.2 | 100.0 |
|  | Total | 215 | 100.0 | 100.0 |  |

| **ResIA_LT15** | | | | | |
| --- | --- | --- | --- | --- | --- |
|  | | Frequency | Percent | Valid Percent | Cumulative Percent |
| Valid | 1 | 4 | 1.9 | 1.9 | 1.9 |
|  | 2 | 9 | 4.2 | 4.2 | 6.0 |
|  | 3 | 18 | 8.4 | 8.4 | 14.4 |
|  | 4 | 35 | 16.3 | 16.3 | 30.7 |
|  | 5 | 149 | 69.3 | 69.3 | 100.0 |
|  | Total | 215 | 100.0 | 100.0 |  |

| **ResIA15_30** | | | | | |
| --- | --- | --- | --- | --- | --- |
|  | | Frequency | Percent | Valid Percent | Cumulative Percent |
| Valid | 1 | 27 | 12.6 | 12.6 | 12.6 |
|  | 2 | 43 | 20.0 | 20.0 | 32.6 |
|  | 3 | 32 | 14.9 | 14.9 | 47.4 |
|  | 4 | 111 | 51.6 | 51.6 | 99.1 |
|  | 5 | 2 | .9 | .9 | 100.0 |
|  | Total | 215 | 100.0 | 100.0 |  |

| **ResIA30_45** | | | | | |
| --- | --- | --- | --- | --- | --- |
|  | | Frequency | Percent | Valid Percent | Cumulative Percent |
| Valid | 1 | 84 | 39.1 | 39.1 | 39.1 |
|  | 2 | 53 | 24.7 | 24.7 | 63.7 |
|  | 3 | 78 | 36.3 | 36.3 | 100.0 |
|  | Total | 215 | 100.0 | 100.0 |  |

| **ResIA45_60** | | | | | |
| --- | --- | --- | --- | --- | --- |
|  | | Frequency | Percent | Valid Percent | Cumulative Percent |
| Valid | 1 | 65 | 30.2 | 30.2 | 30.2 |
|  | 2 | 82 | 38.1 | 38.1 | 68.4 |
|  | 3 | 37 | 17.2 | 17.2 | 85.6 |
|  | 4 | 29 | 13.5 | 13.5 | 99.1 |
|  | 5 | 2 | .9 | .9 | 100.0 |
|  | Total | 215 | 100.0 | 100.0 |  |

| **ResIA_GT60** | | | | | |
| --- | --- | --- | --- | --- | --- |
|  | | Frequency | Percent | Valid Percent | Cumulative Percent |
| Valid | 1 | 35 | 16.3 | 16.3 | 16.3 |
|  | 2 | 27 | 12.6 | 12.6 | 28.8 |
|  | 3 | 50 | 23.3 | 23.3 | 52.1 |
|  | 4 | 40 | 18.6 | 18.6 | 70.7 |
|  | 5 | 63 | 29.3 | 29.3 | 100.0 |
|  | Total | 215 | 100.0 | 100.0 |  |

| **october** | | | | | |
| --- | --- | --- | --- | --- | --- |
|  | | Frequency | Percent | Valid Percent | Cumulative Percent |
| Valid | 1 | 33 | 15.3 | 15.3 | 15.3 |
|  | 2 | 42 | 19.5 | 19.5 | 34.9 |
|  | 3 | 52 | 24.2 | 24.2 | 59.1 |
|  | 4 | 66 | 30.7 | 30.7 | 89.8 |
|  | 5 | 22 | 10.2 | 10.2 | 100.0 |
|  | Total | 215 | 100.0 | 100.0 |  |

| **november** | | | | | |
| --- | --- | --- | --- | --- | --- |
|  | | Frequency | Percent | Valid Percent | Cumulative Percent |
| Valid | 1 | 128 | 59.5 | 59.5 | 59.5 |
|  | 2 | 60 | 27.9 | 27.9 | 87.4 |
|  | 3 | 16 | 7.4 | 7.4 | 94.9 |
|  | 4 | 10 | 4.7 | 4.7 | 99.5 |
|  | 5 | 1 | .5 | .5 | 100.0 |
|  | Total | 215 | 100.0 | 100.0 |  |
| **december** | | | | | |
|  | | Frequency | Percent | Valid Percent | Cumulative Percent |
| Valid | 1 | 44 | 20.5 | 20.5 | 20.5 |
|  | 2 | 92 | 42.8 | 42.8 | 63.3 |
|  | 3 | 71 | 33.0 | 33.0 | 96.3 |
|  | 4 | 3 | 1.4 | 1.4 | 97.7 |
|  | 5 | 5 | 2.3 | 2.3 | 100.0 |
|  | Total | 215 | 100.0 | 100.0 |  |

| **january** | | | | | |
| --- | --- | --- | --- | --- | --- |
|  | | Frequency | Percent | Valid Percent | Cumulative Percent |
| Valid | 1 | 7 | 3.3 | 3.3 | 3.3 |
|  | 2 | 18 | 8.4 | 8.4 | 11.6 |
|  | 3 | 72 | 33.5 | 33.5 | 45.1 |
|  | 4 | 116 | 54.0 | 54.0 | 99.1 |
|  | 5 | 2 | .9 | .9 | 100.0 |
|  | Total | 215 | 100.0 | 100.0 |  |

| **february** | | | | | |
| --- | --- | --- | --- | --- | --- |
|  | | Frequency | Percent | Valid Percent | Cumulative Percent |
| Valid | 1 | 3 | 1.4 | 1.4 | 1.4 |
|  | 2 | 3 | 1.4 | 1.4 | 2.8 |
|  | 3 | 4 | 1.9 | 1.9 | 4.7 |
|  | 4 | 20 | 9.3 | 9.3 | 14.0 |
|  | 5 | 185 | 86.0 | 86.0 | 100.0 |
|  | Total | 215 | 100.0 | 100.0 |  |

| **overview** | | | | | |
| --- | --- | --- | --- | --- | --- |
|  | | Frequency | Percent | Valid Percent | Cumulative Percent |
| Valid | 1 | 89 | 41.4 | 41.4 | 41.4 |
|  | 2 | 79 | 36.7 | 36.7 | 78.1 |
|  | 3 | 29 | 13.5 | 13.5 | 91.6 |
|  | 4 | 10 | 4.7 | 4.7 | 96.3 |
|  | 5 | 8 | 3.7 | 3.7 | 100.0 |
|  | Total | 215 | 100.0 | 100.0 |  |
| **interview** | | | | | |
|  | | Frequency | Percent | Valid Percent | Cumulative Percent |
| Valid | 1 | 92 | 42.8 | 42.8 | 42.8 |
|  | 2 | 80 | 37.2 | 37.2 | 80.0 |
|  | 3 | 27 | 12.6 | 12.6 | 92.6 |
|  | 4 | 12 | 5.6 | 5.6 | 98.1 |
|  | 5 | 4 | 1.9 | 1.9 | 100.0 |
|  | Total | 215 | 100.0 | 100.0 |  |

| **tour** | | | | | |
| --- | --- | --- | --- | --- | --- |
|  | | Frequency | Percent | Valid Percent | Cumulative Percent |
| Valid | 1 | 3 | 1.4 | 1.4 | 1.4 |
|  | 2 | 4 | 1.9 | 1.9 | 3.3 |
|  | 3 | 42 | 19.5 | 19.5 | 22.8 |
|  | 4 | 80 | 37.2 | 37.2 | 60.0 |
|  | 5 | 86 | 40.0 | 40.0 | 100.0 |
|  | Total | 215 | 100.0 | 100.0 |  |

| **morning_report** | | | | | |
| --- | --- | --- | --- | --- | --- |
|  | | Frequency | Percent | Valid Percent | Cumulative Percent |
| Valid | 1 | 6 | 2.8 | 2.8 | 2.8 |
|  | 2 | 14 | 6.5 | 6.5 | 9.3 |
|  | 3 | 33 | 15.3 | 15.3 | 24.7 |
|  | 4 | 66 | 30.7 | 30.7 | 55.3 |
|  | 5 | 96 | 44.7 | 44.7 | 100.0 |
|  | Total | 215 | 100.0 | 100.0 |  |

| **lunch** | | | | | |
| --- | --- | --- | --- | --- | --- |
|  | | Frequency | Percent | Valid Percent | Cumulative Percent |
| Valid | 1 | 25 | 11.6 | 11.6 | 11.6 |
|  | 2 | 38 | 17.7 | 17.7 | 29.3 |
|  | 3 | 82 | 38.1 | 38.1 | 67.4 |
|  | 4 | 47 | 21.9 | 21.9 | 89.3 |
|  | 5 | 22 | 10.2 | 10.2 | 99.5 |
|  | 33 | 1 | .5 | .5 | 100.0 |
|  | Total | 215 | 100.0 | 100.0 |  |

| **dinner** | | | | | |
| --- | --- | --- | --- | --- | --- |
|  | | Frequency | Percent | Valid Percent | Cumulative Percent |
| Valid | yes | 121 | 56.3 | 56.3 | 56.3 |
|  | no | 94 | 43.7 | 43.7 | 100.0 |
|  | Total | 215 | 100.0 | 100.0 |  |

| **tour_optional** | | | | | |
| --- | --- | --- | --- | --- | --- |
|  | | Frequency | Percent | Valid Percent | Cumulative Percent |
| Valid | yes | 74 | 34.4 | 34.4 | 34.4 |
|  | no | 141 | 65.6 | 65.6 | 100.0 |
|  | Total | 215 | 100.0 | 100.0 |  |

| **exit_interview** | | | | | |
| --- | --- | --- | --- | --- | --- |
|  | | Frequency | Percent | Valid Percent | Cumulative Percent |
| Valid | neccesary | 96 | 44.7 | 44.7 | 44.7 |
|  | unneccesary | 119 | 55.3 | 55.3 | 100.0 |
|  | Total | 215 | 100.0 | 100.0 |  |

**DESCRIPTIVES RECODE**

1 = PREFERRED (i.e., 1 or 2)

2 = NOT PREFFERED (i.e., 4 or 5)

| **INT_phone_recode** | | | | | |
| --- | --- | --- | --- | --- | --- |
|  | | Frequency | Percent | Valid Percent | Cumulative Percent |
| Valid | 1 | 22 | 10.2 | 13.4 | 13.4 |
|  | 2 | 142 | 66.0 | 86.6 | 100.0 |
|  | Total | 164 | 76.3 | 100.0 |  |
| Missing | System | 51 | 23.7 |  |  |
| Total | | 215 | 100.0 |  |  |

| **INT_lunch_recode** | | | | | |
| --- | --- | --- | --- | --- | --- |
|  | | Frequency | Percent | Valid Percent | Cumulative Percent |
| Valid | 1 | 112 | 52.1 | 77.2 | 77.2 |
|  | 2 | 33 | 15.3 | 22.8 | 100.0 |
|  | Total | 145 | 67.4 | 100.0 |  |
| Missing | System | 70 | 32.6 |  |  |
| Total | | 215 | 100.0 |  |  |

| **INT_one_on_one_recode** | | | | | |
| --- | --- | --- | --- | --- | --- |
|  | | Frequency | Percent | Valid Percent | Cumulative Percent |
| Valid | 1 | 211 | 98.1 | 98.1 | 98.1 |
|  | 2 | 4 | 1.9 | 1.9 | 100.0 |
|  | Total | 215 | 100.0 | 100.0 |  |

| **INT_panel2_recode** | | | | | |
| --- | --- | --- | --- | --- | --- |
|  | | Frequency | Percent | Valid Percent | Cumulative Percent |
| Valid | 1 | 77 | 35.8 | 50.0 | 50.0 |
|  | 2 | 77 | 35.8 | 50.0 | 100.0 |
|  | Total | 154 | 71.6 | 100.0 |  |
| Missing | System | 61 | 28.4 |  |  |
| Total | | 215 | 100.0 |  |  |

| **INT_group_recode** | | | | | |
| --- | --- | --- | --- | --- | --- |
|  | | Frequency | Percent | Valid Percent | Cumulative Percent |
| Valid | 1 | 8 | 3.7 | 4.4 | 4.4 |
|  | 2 | 174 | 80.9 | 95.6 | 100.0 |
|  | Total | 182 | 84.7 | 100.0 |  |
| Missing | System | 33 | 15.3 |  |  |
| Total | | 215 | 100.0 |  |  |

| **start7_8_recode** | | | | | |
| --- | --- | --- | --- | --- | --- |
|  | | Frequency | Percent | Valid Percent | Cumulative Percent |
| Valid | 1 | 77 | 35.8 | 48.4 | 48.4 |
|  | 2 | 82 | 38.1 | 51.6 | 100.0 |
|  | Total | 159 | 74.0 | 100.0 |  |
| Missing | System | 56 | 26.0 |  |  |
| Total | | 215 | 100.0 |  |  |

| **start8_9_recode** | | | | | |
| --- | --- | --- | --- | --- | --- |
|  | | Frequency | Percent | Valid Percent | Cumulative Percent |
| Valid | 1 | 176 | 81.9 | 87.6 | 87.6 |
|  | 2 | 25 | 11.6 | 12.4 | 100.0 |
|  | Total | 201 | 93.5 | 100.0 |  |
| Missing | System | 14 | 6.5 |  |  |
| Total | | 215 | 100.0 |  |  |

| **start9_10_recode** | | | | | |
| --- | --- | --- | --- | --- | --- |
|  | | Frequency | Percent | Valid Percent | Cumulative Percent |
| Valid | 1 | 132 | 61.4 | 95.0 | 95.0 |
|  | 2 | 7 | 3.3 | 5.0 | 100.0 |
|  | Total | 139 | 64.7 | 100.0 |  |
| Missing | System | 76 | 35.3 |  |  |
| Total | | 215 | 100.0 |  |  |

| **start10_11_recode** | | | | | |
| --- | --- | --- | --- | --- | --- |
|  | | Frequency | Percent | Valid Percent | Cumulative Percent |
| Valid | 1 | 34 | 15.8 | 21.0 | 21.0 |
|  | 2 | 128 | 59.5 | 79.0 | 100.0 |
|  | Total | 162 | 75.3 | 100.0 |  |
| Missing | System | 53 | 24.7 |  |  |
| Total | | 215 | 100.0 |  |  |

| **start11_12_recode** | | | | | |
| --- | --- | --- | --- | --- | --- |
|  | | Frequency | Percent | Valid Percent | Cumulative Percent |
| Valid | 1 | 11 | 5.1 | 5.5 | 5.5 |
|  | 2 | 188 | 87.4 | 94.5 | 100.0 |
|  | Total | 199 | 92.6 | 100.0 |  |
| Missing | System | 16 | 7.4 |  |  |
| Total | | 215 | 100.0 |  |  |

| **lengthLT2_recode** | | | | | |
| --- | --- | --- | --- | --- | --- |
|  | | Frequency | Percent | Valid Percent | Cumulative Percent |
| Valid | 1 | 21 | 9.8 | 11.9 | 11.9 |
|  | 2 | 155 | 72.1 | 88.1 | 100.0 |
|  | Total | 176 | 81.9 | 100.0 |  |
| Missing | System | 39 | 18.1 |  |  |
| Total | | 215 | 100.0 |  |  |

| **length2_recode** | | | | | |
| --- | --- | --- | --- | --- | --- |
|  | | Frequency | Percent | Valid Percent | Cumulative Percent |
| Valid | 1 | 92 | 42.8 | 63.0 | 63.0 |
|  | 2 | 54 | 25.1 | 37.0 | 100.0 |
|  | Total | 146 | 67.9 | 100.0 |  |
| Missing | System | 69 | 32.1 |  |  |
| Total | | 215 | 100.0 |  |  |

| **length4_recode** | | | | | |
| --- | --- | --- | --- | --- | --- |
|  | | Frequency | Percent | Valid Percent | Cumulative Percent |
| Valid | 1 | 178 | 82.8 | 97.8 | 97.8 |
|  | 2 | 4 | 1.9 | 2.2 | 100.0 |
|  | Total | 182 | 84.7 | 100.0 |  |
| Missing | System | 33 | 15.3 |  |  |
| Total | | 215 | 100.0 |  |  |

| **length6_recode** | | | | | |
| --- | --- | --- | --- | --- | --- |
|  | | Frequency | Percent | Valid Percent | Cumulative Percent |
| Valid | 1 | 120 | 55.8 | 68.6 | 68.6 |
|  | 2 | 55 | 25.6 | 31.4 | 100.0 |
|  | Total | 175 | 81.4 | 100.0 |  |
| Missing | System | 40 | 18.6 |  |  |
| Total | | 215 | 100.0 |  |  |

| **length8_recode** | | | | | |
| --- | --- | --- | --- | --- | --- |
|  | | Frequency | Percent | Valid Percent | Cumulative Percent |
| Valid | 1 | 19 | 8.8 | 10.5 | 10.5 |
|  | 2 | 162 | 75.3 | 89.5 | 100.0 |
|  | Total | 181 | 84.2 | 100.0 |  |
| Missing | System | 34 | 15.8 |  |  |
| Total | | 215 | 100.0 |  |  |

| **faculty1_recode** | | | | | |
| --- | --- | --- | --- | --- | --- |
|  | | Frequency | Percent | Valid Percent | Cumulative Percent |
| Valid | 1 | 49 | 22.8 | 36.6 | 36.6 |
|  | 2 | 85 | 39.5 | 63.4 | 100.0 |
|  | Total | 134 | 62.3 | 100.0 |  |
| Missing | System | 81 | 37.7 |  |  |
| Total | | 215 | 100.0 |  |  |

| **faculty2_recode** | | | | | |
| --- | --- | --- | --- | --- | --- |
|  | | Frequency | Percent | Valid Percent | Cumulative Percent |
| Valid | 1 | 195 | 90.7 | 94.7 | 94.7 |
|  | 2 | 11 | 5.1 | 5.3 | 100.0 |
|  | Total | 206 | 95.8 | 100.0 |  |
| Missing | System | 9 | 4.2 |  |  |
| Total | | 215 | 100.0 |  |  |

| **faculty3_recode** | | | | | |
| --- | --- | --- | --- | --- | --- |
|  | | Frequency | Percent | Valid Percent | Cumulative Percent |
| Valid | 1 | 162 | 75.3 | 98.8 | 98.8 |
|  | 2 | 2 | .9 | 1.2 | 100.0 |
|  | Total | 164 | 76.3 | 100.0 |  |
| Missing | System | 51 | 23.7 |  |  |
| Total | | 215 | 100.0 |  |  |

| **faculty4_recode** | | | | | |
| --- | --- | --- | --- | --- | --- |
|  | | Frequency | Percent | Valid Percent | Cumulative Percent |
| Valid | 1 | 19 | 8.8 | 12.9 | 12.9 |
|  | 2 | 128 | 59.5 | 87.1 | 100.0 |
|  | Total | 147 | 68.4 | 100.0 |  |
| Missing | System | 68 | 31.6 |  |  |
| Total | | 215 | 100.0 |  |  |

| **facultyGT4_recode** | | | | | |
| --- | --- | --- | --- | --- | --- |
|  | | Frequency | Percent | Valid Percent | Cumulative Percent |
| Valid | 1 | 5 | 2.3 | 2.4 | 2.4 |
|  | 2 | 204 | 94.9 | 97.6 | 100.0 |
|  | Total | 209 | 97.2 | 100.0 |  |
| Missing | System | 6 | 2.8 |  |  |
| Total | | 215 | 100.0 |  |  |

| **lengthLT15_recode** | | | | | |
| --- | --- | --- | --- | --- | --- |
|  | | Frequency | Percent | Valid Percent | Cumulative Percent |
| Valid | 1 | 120 | 55.8 | 77.4 | 77.4 |
|  | 2 | 35 | 16.3 | 22.6 | 100.0 |
|  | Total | 155 | 72.1 | 100.0 |  |
| Missing | System | 60 | 27.9 |  |  |
| Total | | 215 | 100.0 |  |  |

| **length15_30_recode** | | | | | |
| --- | --- | --- | --- | --- | --- |
|  | | Frequency | Percent | Valid Percent | Cumulative Percent |
| Valid | 1 | 205 | 95.3 | 97.6 | 97.6 |
|  | 2 | 5 | 2.3 | 2.4 | 100.0 |
|  | Total | 210 | 97.7 | 100.0 |  |
| Missing | System | 5 | 2.3 |  |  |
| Total | | 215 | 100.0 |  |  |

| **length30_45_recode** | | | | | |
| --- | --- | --- | --- | --- | --- |
|  | | Frequency | Percent | Valid Percent | Cumulative Percent |
| Valid | 1 | 92 | 42.8 | 98.9 | 98.9 |
|  | 2 | 1 | .5 | 1.1 | 100.0 |
|  | Total | 93 | 43.3 | 100.0 |  |
| Missing | System | 122 | 56.7 |  |  |
| Total | | 215 | 100.0 |  |  |

| **length45_60_recode** | | | | | |
| --- | --- | --- | --- | --- | --- |
|  | | Frequency | Percent | Valid Percent | Cumulative Percent |
| Valid | 1 | 9 | 4.2 | 4.8 | 4.8 |
|  | 2 | 178 | 82.8 | 95.2 | 100.0 |
|  | Total | 187 | 87.0 | 100.0 |  |
| Missing | System | 28 | 13.0 |  |  |
| Total | | 215 | 100.0 |  |  |

| **lengthGT60_recode** | | | | | |
| --- | --- | --- | --- | --- | --- |
|  | | Frequency | Percent | Valid Percent | Cumulative Percent |
| Valid | 1 | 5 | 2.3 | 2.3 | 2.3 |
|  | 2 | 210 | 97.7 | 97.7 | 100.0 |
|  | Total | 215 | 100.0 | 100.0 |  |

| **INT_res_chief_recode** | | | | | |
| --- | --- | --- | --- | --- | --- |
|  | | Frequency | Percent | Valid Percent | Cumulative Percent |
| Valid | 1 | 28 | 13.0 | 15.4 | 15.4 |
|  | 2 | 154 | 71.6 | 84.6 | 100.0 |
|  | Total | 182 | 84.7 | 100.0 |  |
| Missing | System | 33 | 15.3 |  |  |
| Total | | 215 | 100.0 |  |  |

| **INT_faculty_recode** | | | | | |
| --- | --- | --- | --- | --- | --- |
|  | | Frequency | Percent | Valid Percent | Cumulative Percent |
| Valid | 1 | 44 | 20.5 | 30.6 | 30.6 |
|  | 2 | 100 | 46.5 | 69.4 | 100.0 |
|  | Total | 144 | 67.0 | 100.0 |  |
| Missing | System | 71 | 33.0 |  |  |
| Total | | 215 | 100.0 |  |  |

| **INT_APD_recode** | | | | | |
| --- | --- | --- | --- | --- | --- |
|  | | Frequency | Percent | Valid Percent | Cumulative Percent |
| Valid | 1 | 138 | 64.2 | 89.0 | 89.0 |
|  | 2 | 17 | 7.9 | 11.0 | 100.0 |
|  | Total | 155 | 72.1 | 100.0 |  |
| Missing | System | 60 | 27.9 |  |  |
| Total | | 215 | 100.0 |  |  |

| **INT_PD_recode** | | | | | |
| --- | --- | --- | --- | --- | --- |
|  | | Frequency | Percent | Valid Percent | Cumulative Percent |
| Valid | 1 | 186 | 86.5 | 94.9 | 94.9 |
|  | 2 | 10 | 4.7 | 5.1 | 100.0 |
|  | Total | 196 | 91.2 | 100.0 |  |
| Missing | System | 19 | 8.8 |  |  |
| Total | | 215 | 100.0 |  |  |

| **INT_chair_recode** | | | | | |
| --- | --- | --- | --- | --- | --- |
|  | | Frequency | Percent | Valid Percent | Cumulative Percent |
| Valid | 1 | 34 | 15.8 | 18.6 | 18.6 |
|  | 2 | 149 | 69.3 | 81.4 | 100.0 |
|  | Total | 183 | 85.1 | 100.0 |  |
| Missing | System | 32 | 14.9 |  |  |
| Total | | 215 | 100.0 |  |  |

| **Qstraightforward_recode** | | | | | |
| --- | --- | --- | --- | --- | --- |
|  | | Frequency | Percent | Valid Percent | Cumulative Percent |
| Valid | 1 | 188 | 87.4 | 92.6 | 92.6 |
|  | 2 | 15 | 7.0 | 7.4 | 100.0 |
|  | Total | 203 | 94.4 | 100.0 |  |
| Missing | System | 12 | 5.6 |  |  |
| Total | | 215 | 100.0 |  |  |

| **Qbehavior_recode** | | | | | |
| --- | --- | --- | --- | --- | --- |
|  | | Frequency | Percent | Valid Percent | Cumulative Percent |
| Valid | 1 | 139 | 64.7 | 92.1 | 92.1 |
|  | 2 | 12 | 5.6 | 7.9 | 100.0 |
|  | Total | 151 | 70.2 | 100.0 |  |
| Missing | System | 64 | 29.8 |  |  |
| Total | | 215 | 100.0 |  |  |

| **Qsituation_recode** | | | | | |
| --- | --- | --- | --- | --- | --- |
|  | | Frequency | Percent | Valid Percent | Cumulative Percent |
| Valid | 1 | 81 | 37.7 | 74.3 | 74.3 |
|  | 2 | 28 | 13.0 | 25.7 | 100.0 |
|  | Total | 109 | 50.7 | 100.0 |  |
| Missing | System | 106 | 49.3 |  |  |
| Total | | 215 | 100.0 |  |  |

| **Qteaser_recode** | | | | | |
| --- | --- | --- | --- | --- | --- |
|  | | Frequency | Percent | Valid Percent | Cumulative Percent |
| Valid | 1 | 19 | 8.8 | 9.9 | 9.9 |
|  | 2 | 173 | 80.5 | 90.1 | 100.0 |
|  | Total | 192 | 89.3 | 100.0 |  |
| Missing | System | 23 | 10.7 |  |  |
| Total | | 215 | 100.0 |  |  |

| **Qmedical_recode** | | | | | |
| --- | --- | --- | --- | --- | --- |
|  | | Frequency | Percent | Valid Percent | Cumulative Percent |
| Valid | 1 | 3 | 1.4 | 1.5 | 1.5 |
|  | 2 | 202 | 94.0 | 98.5 | 100.0 |
|  | Total | 205 | 95.3 | 100.0 |  |
| Missing | System | 10 | 4.7 |  |  |
| Total | | 215 | 100.0 |  |  |

| **ResIA_LT15_recode** | | | | | |
| --- | --- | --- | --- | --- | --- |
|  | | Frequency | Percent | Valid Percent | Cumulative Percent |
| Valid | 1 | 13 | 6.0 | 6.6 | 6.6 |
|  | 2 | 184 | 85.6 | 93.4 | 100.0 |
|  | Total | 197 | 91.6 | 100.0 |  |
| Missing | System | 18 | 8.4 |  |  |
| Total | | 215 | 100.0 |  |  |

| **ResIA15_30_recode** | | | | | |
| --- | --- | --- | --- | --- | --- |
|  | | Frequency | Percent | Valid Percent | Cumulative Percent |
| Valid | 1 | 70 | 32.6 | 38.3 | 38.3 |
|  | 2 | 113 | 52.6 | 61.7 | 100.0 |
|  | Total | 183 | 85.1 | 100.0 |  |
| Missing | System | 32 | 14.9 |  |  |
| Total | | 215 | 100.0 |  |  |

| **ResIA30_45_recode** | | | | | |
| --- | --- | --- | --- | --- | --- |
|  | | Frequency | Percent | Valid Percent | Cumulative Percent |
| Valid | 1 | 137 | 63.7 | 100.0 | 100.0 |
| Missing | System | 78 | 36.3 |  |  |
| Total | | 215 | 100.0 |  |  |

| **ResIA45_60_recode** | | | | | |
| --- | --- | --- | --- | --- | --- |
|  | | Frequency | Percent | Valid Percent | Cumulative Percent |
| Valid | 1 | 147 | 68.4 | 82.6 | 82.6 |
|  | 2 | 31 | 14.4 | 17.4 | 100.0 |
|  | Total | 178 | 82.8 | 100.0 |  |
| Missing | System | 37 | 17.2 |  |  |
| Total | | 215 | 100.0 |  |  |

| **ResIA_GT60_recode** | | | | | |
| --- | --- | --- | --- | --- | --- |
|  | | Frequency | Percent | Valid Percent | Cumulative Percent |
| Valid | 1 | 62 | 28.8 | 37.6 | 37.6 |
|  | 2 | 103 | 47.9 | 62.4 | 100.0 |
|  | Total | 165 | 76.7 | 100.0 |  |
| Missing | System | 50 | 23.3 |  |  |
| Total | | 215 | 100.0 |  |  |

| **october_recode** | | | | | |
| --- | --- | --- | --- | --- | --- |
|  | | Frequency | Percent | Valid Percent | Cumulative Percent |
| Valid | 1 | 75 | 34.9 | 46.0 | 46.0 |
|  | 2 | 88 | 40.9 | 54.0 | 100.0 |
|  | Total | 163 | 75.8 | 100.0 |  |
| Missing | System | 52 | 24.2 |  |  |
| Total | | 215 | 100.0 |  |  |

| **november_recode** | | | | | |
| --- | --- | --- | --- | --- | --- |
|  | | Frequency | Percent | Valid Percent | Cumulative Percent |
| Valid | 1 | 188 | 87.4 | 94.5 | 94.5 |
|  | 2 | 11 | 5.1 | 5.5 | 100.0 |
|  | Total | 199 | 92.6 | 100.0 |  |
| Missing | System | 16 | 7.4 |  |  |
| Total | | 215 | 100.0 |  |  |

| **december_recode** | | | | | |
| --- | --- | --- | --- | --- | --- |
|  | | Frequency | Percent | Valid Percent | Cumulative Percent |
| Valid | 1 | 136 | 63.3 | 94.4 | 94.4 |
|  | 2 | 8 | 3.7 | 5.6 | 100.0 |
|  | Total | 144 | 67.0 | 100.0 |  |
| Missing | System | 71 | 33.0 |  |  |
| Total | | 215 | 100.0 |  |  |

| **january_recode** | | | | | |
| --- | --- | --- | --- | --- | --- |
|  | | Frequency | Percent | Valid Percent | Cumulative Percent |
| Valid | 1 | 25 | 11.6 | 17.5 | 17.5 |
|  | 2 | 118 | 54.9 | 82.5 | 100.0 |
|  | Total | 143 | 66.5 | 100.0 |  |
| Missing | System | 72 | 33.5 |  |  |
| Total | | 215 | 100.0 |  |  |

| **february_recode** | | | | | |
| --- | --- | --- | --- | --- | --- |
|  | | Frequency | Percent | Valid Percent | Cumulative Percent |
| Valid | 1 | 6 | 2.8 | 2.8 | 2.8 |
|  | 2 | 205 | 95.3 | 97.2 | 100.0 |
|  | Total | 211 | 98.1 | 100.0 |  |
| Missing | System | 4 | 1.9 |  |  |
| Total | | 215 | 100.0 |  |  |

| **overview_recode** | | | | | |
| --- | --- | --- | --- | --- | --- |
|  | | Frequency | Percent | Valid Percent | Cumulative Percent |
| Valid | 1 | 168 | 78.1 | 90.3 | 90.3 |
|  | 2 | 18 | 8.4 | 9.7 | 100.0 |
|  | Total | 186 | 86.5 | 100.0 |  |
| Missing | System | 29 | 13.5 |  |  |
| Total | | 215 | 100.0 |  |  |

| **interview_recode** | | | | | |
| --- | --- | --- | --- | --- | --- |
|  | | Frequency | Percent | Valid Percent | Cumulative Percent |
| Valid | 1 | 172 | 80.0 | 91.5 | 91.5 |
|  | 2 | 16 | 7.4 | 8.5 | 100.0 |
|  | Total | 188 | 87.4 | 100.0 |  |
| Missing | System | 27 | 12.6 |  |  |
| Total | | 215 | 100.0 |  |  |

| **tour_recode** | | | | | |
| --- | --- | --- | --- | --- | --- |
|  | | Frequency | Percent | Valid Percent | Cumulative Percent |
| Valid | 1 | 7 | 3.3 | 4.0 | 4.0 |
|  | 2 | 166 | 77.2 | 96.0 | 100.0 |
|  | Total | 173 | 80.5 | 100.0 |  |
| Missing | System | 42 | 19.5 |  |  |
| Total | | 215 | 100.0 |  |  |

| **morning_report_recode** | | | | | |
| --- | --- | --- | --- | --- | --- |
|  | | Frequency | Percent | Valid Percent | Cumulative Percent |
| Valid | 1 | 20 | 9.3 | 11.0 | 11.0 |
|  | 2 | 162 | 75.3 | 89.0 | 100.0 |
|  | Total | 182 | 84.7 | 100.0 |  |
| Missing | System | 33 | 15.3 |  |  |
| Total | | 215 | 100.0 |  |  |

| **lunch_recode** | | | | | |
| --- | --- | --- | --- | --- | --- |
|  | | Frequency | Percent | Valid Percent | Cumulative Percent |
| Valid | 1 | 63 | 29.3 | 47.7 | 47.7 |
|  | 2 | 69 | 32.1 | 52.3 | 100.0 |
|  | Total | 132 | 61.4 | 100.0 |  |
| Missing | System | 83 | 38.6 |  |  |
| Total | | 215 | 100.0 |  |  |

**candidate * INT_phone**

| **Crosstab** | | | | | | |
| --- | --- | --- | --- | --- | --- | --- |
|  | | | INT_phone | | | |
|  |  |  | 1 | 2 | 3 | 4 |
| candidate | US categorical | Count | 1 | 2 | 24 | 29 |
|  |  | % within candidate | 1.0% | 2.1% | 25.0% | 30.2% |
|  |  | % of Total | 0.5% | 0.9% | 11.2% | 13.5% |
|  | IM categorical | Count | 1 | 5 | 9 | 19 |
|  |  | % within candidate | 1.5% | 7.7% | 13.8% | 29.2% |
|  |  | % of Total | 0.5% | 2.3% | 4.2% | 8.8% |
|  | Preliminary | Count | 2 | 11 | 18 | 10 |
|  |  | % within candidate | 3.7% | 20.4% | 33.3% | 18.5% |
|  |  | % of Total | 0.9% | 5.1% | 8.4% | 4.7% |
| Total | | Count | 4 | 18 | 51 | 58 |
|  |  | % within candidate | 1.9% | 8.4% | 23.7% | 27.0% |
|  |  | % of Total | 1.9% | 8.4% | 23.7% | 27.0% |

| **Crosstab** | | | | |
| --- | --- | --- | --- | --- |
|  | | | INT_phone | Total |
|  |  |  | 5 |  |
| candidate | US categorical | Count | 40 | 96 |
|  |  | % within candidate | 41.7% | 100.0% |
|  |  | % of Total | 18.6% | 44.7% |
|  | IM categorical | Count | 31 | 65 |
|  |  | % within candidate | 47.7% | 100.0% |
|  |  | % of Total | 14.4% | 30.2% |
|  | Preliminary | Count | 13 | 54 |
|  |  | % within candidate | 24.1% | 100.0% |
|  |  | % of Total | 6.0% | 25.1% |
| Total | | Count | 84 | 215 |
|  |  | % within candidate | 39.1% | 100.0% |
|  |  | % of Total | 39.1% | 100.0% |

| **Chi-Square Tests** | | | |
| --- | --- | --- | --- |
|  | Value | df | Asymp. Sig. (2-sided) |
| Pearson Chi-Square | 26.503^a^ | 8 | .001 |
| Likelihood Ratio | 26.453 | 8 | .001 |
| Linear-by-Linear Association | 12.592 | 1 | .000 |
| N of Valid Cases | 215 |  |  |

| a. 4 cells (26.7%) have expected count less than 5. The minimum expected count is 1.00. |
| --- |

**candidate * INT_lunch**

| **Crosstab** | | | | | | |
| --- | --- | --- | --- | --- | --- | --- |
|  | | | INT_lunch | | | |
|  |  |  | 1 | 2 | 3 | 4 |
| candidate | US categorical | Count | 1 | 58 | 31 | 5 |
|  |  | % within candidate | 1.0% | 60.4% | 32.3% | 5.2% |
|  |  | % of Total | 0.5% | 27.0% | 14.4% | 2.3% |
|  | IM categorical | Count | 0 | 27 | 20 | 15 |
|  |  | % within candidate | 0.0% | 41.5% | 30.8% | 23.1% |
|  |  | % of Total | 0.0% | 12.6% | 9.3% | 7.0% |
|  | Preliminary | Count | 0 | 26 | 19 | 9 |
|  |  | % within candidate | 0.0% | 48.1% | 35.2% | 16.7% |
|  |  | % of Total | 0.0% | 12.1% | 8.8% | 4.2% |
| Total | | Count | 1 | 111 | 70 | 29 |
|  |  | % within candidate | 0.5% | 51.6% | 32.6% | 13.5% |
|  |  | % of Total | 0.5% | 51.6% | 32.6% | 13.5% |

| **Crosstab** | | | | |
| --- | --- | --- | --- | --- |
|  | | | INT_lunch | Total |
|  |  |  | 5 |  |
| candidate | US categorical | Count | 1 | 96 |
|  |  | % within candidate | 1.0% | 100.0% |
|  |  | % of Total | 0.5% | 44.7% |
|  | IM categorical | Count | 3 | 65 |
|  |  | % within candidate | 4.6% | 100.0% |
|  |  | % of Total | 1.4% | 30.2% |
|  | Preliminary | Count | 0 | 54 |
|  |  | % within candidate | 0.0% | 100.0% |
|  |  | % of Total | 0.0% | 25.1% |
| Total | | Count | 4 | 215 |
|  |  | % within candidate | 1.9% | 100.0% |
|  |  | % of Total | 1.9% | 100.0% |

| **Chi-Square Tests** | | | |
| --- | --- | --- | --- |
|  | Value | df | Asymp. Sig. (2-sided) |
| Pearson Chi-Square | 17.981^a^ | 8 | .021 |
| Likelihood Ratio | 19.351 | 8 | .013 |
| Linear-by-Linear Association | 5.073 | 1 | .024 |
| N of Valid Cases | 215 |  |  |

| a. 6 cells (40.0%) have expected count less than 5. The minimum expected count is .25. |
| --- |

**candidate * INT_one_on_one**

| **Crosstab** | | | | | | |
| --- | --- | --- | --- | --- | --- | --- |
|  | | | INT_one_on_one | | | |
|  |  |  | 1 | 2 | 4 | 5 |
| candidate | US categorical | Count | 93 | 1 | 0 | 2 |
|  |  | % within candidate | 96.9% | 1.0% | 0.0% | 2.1% |
|  |  | % of Total | 43.3% | 0.5% | 0.0% | 0.9% |
|  | IM categorical | Count | 60 | 3 | 1 | 1 |
|  |  | % within candidate | 92.3% | 4.6% | 1.5% | 1.5% |
|  |  | % of Total | 27.9% | 1.4% | 0.5% | 0.5% |
|  | Preliminary | Count | 52 | 2 | 0 | 0 |
|  |  | % within candidate | 96.3% | 3.7% | 0.0% | 0.0% |
|  |  | % of Total | 24.2% | 0.9% | 0.0% | 0.0% |
| Total | | Count | 205 | 6 | 1 | 3 |
|  |  | % within candidate | 95.3% | 2.8% | 0.5% | 1.4% |
|  |  | % of Total | 95.3% | 2.8% | 0.5% | 1.4% |

| **Crosstab** | | | |
| --- | --- | --- | --- |
|  | | | Total |
|  |  |  |  |
| candidate | US categorical | Count | 96 |
|  |  | % within candidate | 100.0% |
|  |  | % of Total | 44.7% |
|  | IM categorical | Count | 65 |
|  |  | % within candidate | 100.0% |
|  |  | % of Total | 30.2% |
|  | Preliminary | Count | 54 |
|  |  | % within candidate | 100.0% |
|  |  | % of Total | 25.1% |
| Total | | Count | 215 |
|  |  | % within candidate | 100.0% |
|  |  | % of Total | 100.0% |

| **Chi-Square Tests** | | | |
| --- | --- | --- | --- |
|  | Value | df | Asymp. Sig. (2-sided) |
| Pearson Chi-Square | 5.477^a^ | 6 | .484 |
| Likelihood Ratio | 6.463 | 6 | .373 |
| Linear-by-Linear Association | .208 | 1 | .648 |
| N of Valid Cases | 215 |  |  |

| a. 9 cells (75.0%) have expected count less than 5. The minimum expected count is .25. |
| --- |

**candidate * INT_panel**

| **Crosstab** | | | | | | |
| --- | --- | --- | --- | --- | --- | --- |
|  | | | INT_panel | | | |
|  |  |  | 1 | 2 | 3 | 4 |
| candidate | US categorical | Count | 0 | 33 | 29 | 30 |
|  |  | % within candidate | 0.0% | 34.4% | 30.2% | 31.3% |
|  |  | % of Total | 0.0% | 15.3% | 13.5% | 14.0% |
|  | IM categorical | Count | 2 | 27 | 22 | 11 |
|  |  | % within candidate | 3.1% | 41.5% | 33.8% | 16.9% |
|  |  | % of Total | 0.9% | 12.6% | 10.2% | 5.1% |
|  | Preliminary | Count | 0 | 15 | 10 | 24 |
|  |  | % within candidate | 0.0% | 27.8% | 18.5% | 44.4% |
|  |  | % of Total | 0.0% | 7.0% | 4.7% | 11.2% |
| Total | | Count | 2 | 75 | 61 | 65 |
|  |  | % within candidate | 0.9% | 34.9% | 28.4% | 30.2% |
|  |  | % of Total | 0.9% | 34.9% | 28.4% | 30.2% |

| **Crosstab** | | | | |
| --- | --- | --- | --- | --- |
|  | | | INT_panel | Total |
|  |  |  | 5 |  |
| candidate | US categorical | Count | 4 | 96 |
|  |  | % within candidate | 4.2% | 100.0% |
|  |  | % of Total | 1.9% | 44.7% |
|  | IM categorical | Count | 3 | 65 |
|  |  | % within candidate | 4.6% | 100.0% |
|  |  | % of Total | 1.4% | 30.2% |
|  | Preliminary | Count | 5 | 54 |
|  |  | % within candidate | 9.3% | 100.0% |
|  |  | % of Total | 2.3% | 25.1% |
| Total | | Count | 12 | 215 |
|  |  | % within candidate | 5.6% | 100.0% |
|  |  | % of Total | 5.6% | 100.0% |

| **Chi-Square Tests** | | | |
| --- | --- | --- | --- |
|  | Value | df | Asymp. Sig. (2-sided) |
| Pearson Chi-Square | 18.089^a^ | 8 | .021 |
| Likelihood Ratio | 18.576 | 8 | .017 |
| Linear-by-Linear Association | 1.964 | 1 | .161 |
| N of Valid Cases | 215 |  |  |

| a. 5 cells (33.3%) have expected count less than 5. The minimum expected count is .50. |
| --- |

**candidate * INT_group**

| **Crosstab** | | | | | | |
| --- | --- | --- | --- | --- | --- | --- |
|  | | | INT_group | | | |
|  |  |  | 1 | 2 | 3 | 4 |
| candidate | US categorical | Count | 1 | 2 | 12 | 32 |
|  |  | % within candidate | 1.0% | 2.1% | 12.5% | 33.3% |
|  |  | % of Total | 0.5% | 0.9% | 5.6% | 14.9% |
|  | IM categorical | Count | 2 | 3 | 14 | 19 |
|  |  | % within candidate | 3.1% | 4.6% | 21.5% | 29.2% |
|  |  | % of Total | 0.9% | 1.4% | 6.5% | 8.8% |
|  | Preliminary | Count | 0 | 0 | 7 | 11 |
|  |  | % within candidate | 0.0% | 0.0% | 13.0% | 20.4% |
|  |  | % of Total | 0.0% | 0.0% | 3.3% | 5.1% |
| Total | | Count | 3 | 5 | 33 | 62 |
|  |  | % within candidate | 1.4% | 2.3% | 15.3% | 28.8% |
|  |  | % of Total | 1.4% | 2.3% | 15.3% | 28.8% |

| **Crosstab** | | | | |
| --- | --- | --- | --- | --- |
|  | | | INT_group | Total |
|  |  |  | 5 |  |
| candidate | US categorical | Count | 49 | 96 |
|  |  | % within candidate | 51.0% | 100.0% |
|  |  | % of Total | 22.8% | 44.7% |
|  | IM categorical | Count | 27 | 65 |
|  |  | % within candidate | 41.5% | 100.0% |
|  |  | % of Total | 12.6% | 30.2% |
|  | Preliminary | Count | 36 | 54 |
|  |  | % within candidate | 66.7% | 100.0% |
|  |  | % of Total | 16.7% | 25.1% |
| Total | | Count | 112 | 215 |
|  |  | % within candidate | 52.1% | 100.0% |
|  |  | % of Total | 52.1% | 100.0% |

| **Chi-Square Tests** | | | |
| --- | --- | --- | --- |
|  | Value | df | Asymp. Sig. (2-sided) |
| Pearson Chi-Square | 12.864^a^ | 8 | .117 |
| Likelihood Ratio | 14.122 | 8 | .079 |
| Linear-by-Linear Association | .990 | 1 | .320 |
| N of Valid Cases | 215 |  |  |

| a. 6 cells (40.0%) have expected count less than 5. The minimum expected count is .75. |
| --- |

**candidate * start7_8**

| **Crosstab** | | | | | | |
| --- | --- | --- | --- | --- | --- | --- |
|  | | | start7_8 | | | |
|  |  |  | 1 | 2 | 3 | 4 |
| candidate | US categorical | Count | 17 | 26 | 22 | 13 |
|  |  | % within candidate | 17.7% | 27.1% | 22.9% | 13.5% |
|  |  | % of Total | 7.9% | 12.1% | 10.2% | 6.0% |
|  | IM categorical | Count | 8 | 11 | 17 | 9 |
|  |  | % within candidate | 12.3% | 16.9% | 26.2% | 13.8% |
|  |  | % of Total | 3.7% | 5.1% | 7.9% | 4.2% |
|  | Preliminary | Count | 7 | 8 | 17 | 8 |
|  |  | % within candidate | 13.0% | 14.8% | 31.5% | 14.8% |
|  |  | % of Total | 3.3% | 3.7% | 7.9% | 3.7% |
| Total | | Count | 32 | 45 | 56 | 30 |
|  |  | % within candidate | 14.9% | 20.9% | 26.0% | 14.0% |
|  |  | % of Total | 14.9% | 20.9% | 26.0% | 14.0% |

| **Crosstab** | | | | |
| --- | --- | --- | --- | --- |
|  | | | start7_8 | Total |
|  |  |  | 5 |  |
| candidate | US categorical | Count | 18 | 96 |
|  |  | % within candidate | 18.8% | 100.0% |
|  |  | % of Total | 8.4% | 44.7% |
|  | IM categorical | Count | 20 | 65 |
|  |  | % within candidate | 30.8% | 100.0% |
|  |  | % of Total | 9.3% | 30.2% |
|  | Preliminary | Count | 14 | 54 |
|  |  | % within candidate | 25.9% | 100.0% |
|  |  | % of Total | 6.5% | 25.1% |
| Total | | Count | 52 | 215 |
|  |  | % within candidate | 24.2% | 100.0% |
|  |  | % of Total | 24.2% | 100.0% |

| **Chi-Square Tests** | | | |
| --- | --- | --- | --- |
|  | Value | df | Asymp. Sig. (2-sided) |
| Pearson Chi-Square | 7.558^a^ | 8 | .478 |
| Likelihood Ratio | 7.531 | 8 | .481 |
| Linear-by-Linear Association | 3.303 | 1 | .069 |
| N of Valid Cases | 215 |  |  |

| a. 0 cells (0.0%) have expected count less than 5. The minimum expected count is 7.53. |
| --- |

**candidate * start8_9**

| **Crosstab** | | | | | | |
| --- | --- | --- | --- | --- | --- | --- |
|  | | | start8_9 | | | |
|  |  |  | 1 | 2 | 3 | 4 |
| candidate | US categorical | Count | 57 | 27 | 4 | 6 |
|  |  | % within candidate | 59.4% | 28.1% | 4.2% | 6.3% |
|  |  | % of Total | 26.5% | 12.6% | 1.9% | 2.8% |
|  | IM categorical | Count | 27 | 20 | 5 | 11 |
|  |  | % within candidate | 41.5% | 30.8% | 7.7% | 16.9% |
|  |  | % of Total | 12.6% | 9.3% | 2.3% | 5.1% |
|  | Preliminary | Count | 31 | 14 | 5 | 4 |
|  |  | % within candidate | 57.4% | 25.9% | 9.3% | 7.4% |
|  |  | % of Total | 14.4% | 6.5% | 2.3% | 1.9% |
| Total | | Count | 115 | 61 | 14 | 21 |
|  |  | % within candidate | 53.5% | 28.4% | 6.5% | 9.8% |
|  |  | % of Total | 53.5% | 28.4% | 6.5% | 9.8% |

| **Crosstab** | | | | |
| --- | --- | --- | --- | --- |
|  | | | start8_9 | Total |
|  |  |  | 5 |  |
| candidate | US categorical | Count | 2 | 96 |
|  |  | % within candidate | 2.1% | 100.0% |
|  |  | % of Total | 0.9% | 44.7% |
|  | IM categorical | Count | 2 | 65 |
|  |  | % within candidate | 3.1% | 100.0% |
|  |  | % of Total | 0.9% | 30.2% |
|  | Preliminary | Count | 0 | 54 |
|  |  | % within candidate | 0.0% | 100.0% |
|  |  | % of Total | 0.0% | 25.1% |
| Total | | Count | 4 | 215 |
|  |  | % within candidate | 1.9% | 100.0% |
|  |  | % of Total | 1.9% | 100.0% |

| **Chi-Square Tests** | | | |
| --- | --- | --- | --- |
|  | Value | df | Asymp. Sig. (2-sided) |
| Pearson Chi-Square | 10.815^a^ | 8 | .212 |
| Likelihood Ratio | 11.482 | 8 | .176 |
| Linear-by-Linear Association | .386 | 1 | .534 |
| N of Valid Cases | 215 |  |  |

| a. 5 cells (33.3%) have expected count less than 5. The minimum expected count is 1.00. |
| --- |

**candidate * start9_10**

| **Crosstab** | | | | | | |
| --- | --- | --- | --- | --- | --- | --- |
|  | | | start9_10 | | | |
|  |  |  | 1 | 2 | 3 | 4 |
| candidate | US categorical | Count | 18 | 33 | 41 | 3 |
|  |  | % within candidate | 18.8% | 34.4% | 42.7% | 3.1% |
|  |  | % of Total | 8.4% | 15.3% | 19.1% | 1.4% |
|  | IM categorical | Count | 24 | 19 | 21 | 1 |
|  |  | % within candidate | 36.9% | 29.2% | 32.3% | 1.5% |
|  |  | % of Total | 11.2% | 8.8% | 9.8% | 0.5% |
|  | Preliminary | Count | 14 | 24 | 14 | 1 |
|  |  | % within candidate | 25.9% | 44.4% | 25.9% | 1.9% |
|  |  | % of Total | 6.5% | 11.2% | 6.5% | 0.5% |
| Total | | Count | 56 | 76 | 76 | 5 |
|  |  | % within candidate | 26.0% | 35.3% | 35.3% | 2.3% |
|  |  | % of Total | 26.0% | 35.3% | 35.3% | 2.3% |

| **Crosstab** | | | | |
| --- | --- | --- | --- | --- |
|  | | | start9_10 | Total |
|  |  |  | 5 |  |
| candidate | US categorical | Count | 1 | 96 |
|  |  | % within candidate | 1.0% | 100.0% |
|  |  | % of Total | 0.5% | 44.7% |
|  | IM categorical | Count | 0 | 65 |
|  |  | % within candidate | 0.0% | 100.0% |
|  |  | % of Total | 0.0% | 30.2% |
|  | Preliminary | Count | 1 | 54 |
|  |  | % within candidate | 1.9% | 100.0% |
|  |  | % of Total | 0.5% | 25.1% |
| Total | | Count | 2 | 215 |
|  |  | % within candidate | 0.9% | 100.0% |
|  |  | % of Total | 0.9% | 100.0% |

| **Chi-Square Tests** | | | |
| --- | --- | --- | --- |
|  | Value | df | Asymp. Sig. (2-sided) |
| Pearson Chi-Square | 11.490^a^ | 8 | .175 |
| Likelihood Ratio | 11.856 | 8 | .158 |
| Linear-by-Linear Association | 3.670 | 1 | .055 |
| N of Valid Cases | 215 |  |  |

| a. 6 cells (40.0%) have expected count less than 5. The minimum expected count is .50. |
| --- |

**candidate * start10_11**

| **Crosstab** | | | | | | |
| --- | --- | --- | --- | --- | --- | --- |
|  | | | start10_11 | | | |
|  |  |  | 1 | 2 | 3 | 4 |
| candidate | US categorical | Count | 3 | 8 | 23 | 61 |
|  |  | % within candidate | 3.1% | 8.3% | 24.0% | 63.5% |
|  |  | % of Total | 1.4% | 3.7% | 10.7% | 28.4% |
|  | IM categorical | Count | 2 | 13 | 16 | 34 |
|  |  | % within candidate | 3.1% | 20.0% | 24.6% | 52.3% |
|  |  | % of Total | 0.9% | 6.0% | 7.4% | 15.8% |
|  | Preliminary | Count | 1 | 7 | 14 | 31 |
|  |  | % within candidate | 1.9% | 13.0% | 25.9% | 57.4% |
|  |  | % of Total | 0.5% | 3.3% | 6.5% | 14.4% |
| Total | | Count | 6 | 28 | 53 | 126 |
|  |  | % within candidate | 2.8% | 13.0% | 24.7% | 58.6% |
|  |  | % of Total | 2.8% | 13.0% | 24.7% | 58.6% |

| **Crosstab** | | | | |
| --- | --- | --- | --- | --- |
|  | | | start10_11 | Total |
|  |  |  | 5 |  |
| candidate | US categorical | Count | 1 | 96 |
|  |  | % within candidate | 1.0% | 100.0% |
|  |  | % of Total | 0.5% | 44.7% |
|  | IM categorical | Count | 0 | 65 |
|  |  | % within candidate | 0.0% | 100.0% |
|  |  | % of Total | 0.0% | 30.2% |
|  | Preliminary | Count | 1 | 54 |
|  |  | % within candidate | 1.9% | 100.0% |
|  |  | % of Total | 0.5% | 25.1% |
| Total | | Count | 2 | 215 |
|  |  | % within candidate | 0.9% | 100.0% |
|  |  | % of Total | 0.9% | 100.0% |

| **Chi-Square Tests** | | | |
| --- | --- | --- | --- |
|  | Value | df | Asymp. Sig. (2-sided) |
| Pearson Chi-Square | 6.296^a^ | 8 | .614 |
| Likelihood Ratio | 6.710 | 8 | .568 |
| Linear-by-Linear Association | .560 | 1 | .454 |
| N of Valid Cases | 215 |  |  |

| a. 6 cells (40.0%) have expected count less than 5. The minimum expected count is .50. |
| --- |

**candidate * start11_12**

| **Crosstab** | | | | | | |
| --- | --- | --- | --- | --- | --- | --- |
|  | | | start11_12 | | | |
|  |  |  | 1 | 2 | 3 | 4 |
| candidate | US categorical | Count | 1 | 2 | 6 | 13 |
|  |  | % within candidate | 1.0% | 2.1% | 6.3% | 13.5% |
|  |  | % of Total | 0.5% | 0.9% | 2.8% | 6.0% |
|  | IM categorical | Count | 4 | 2 | 6 | 10 |
|  |  | % within candidate | 6.2% | 3.1% | 9.2% | 15.4% |
|  |  | % of Total | 1.9% | 0.9% | 2.8% | 4.7% |
|  | Preliminary | Count | 1 | 1 | 4 | 10 |
|  |  | % within candidate | 1.9% | 1.9% | 7.4% | 18.5% |
|  |  | % of Total | 0.5% | 0.5% | 1.9% | 4.7% |
| Total | | Count | 6 | 5 | 16 | 33 |
|  |  | % within candidate | 2.8% | 2.3% | 7.4% | 15.3% |
|  |  | % of Total | 2.8% | 2.3% | 7.4% | 15.3% |

| **Crosstab** | | | | |
| --- | --- | --- | --- | --- |
|  | | | start11_12 | Total |
|  |  |  | 5 |  |
| candidate | US categorical | Count | 74 | 96 |
|  |  | % within candidate | 77.1% | 100.0% |
|  |  | % of Total | 34.4% | 44.7% |
|  | IM categorical | Count | 43 | 65 |
|  |  | % within candidate | 66.2% | 100.0% |
|  |  | % of Total | 20.0% | 30.2% |
|  | Preliminary | Count | 38 | 54 |
|  |  | % within candidate | 70.4% | 100.0% |
|  |  | % of Total | 17.7% | 25.1% |
| Total | | Count | 155 | 215 |
|  |  | % within candidate | 72.1% | 100.0% |
|  |  | % of Total | 72.1% | 100.0% |

| **Chi-Square Tests** | | | |
| --- | --- | --- | --- |
|  | Value | df | Asymp. Sig. (2-sided) |
| Pearson Chi-Square | 5.784^a^ | 8 | .671 |
| Likelihood Ratio | 5.431 | 8 | .711 |
| Linear-by-Linear Association | .856 | 1 | .355 |
| N of Valid Cases | 215 |  |  |

| a. 8 cells (53.3%) have expected count less than 5. The minimum expected count is 1.26. |
| --- |

**candidate * lengthLT2**

| **Crosstab** | | | | | | |
| --- | --- | --- | --- | --- | --- | --- |
|  | | | lengthLT2 | | | |
|  |  |  | 1 | 2 | 3 | 4 |
| candidate | US categorical | Count | 1 | 2 | 16 | 33 |
|  |  | % within candidate | 1.0% | 2.1% | 16.7% | 34.4% |
|  |  | % of Total | 0.5% | 0.9% | 7.4% | 15.3% |
|  | IM categorical | Count | 2 | 6 | 5 | 17 |
|  |  | % within candidate | 3.1% | 9.2% | 7.7% | 26.2% |
|  |  | % of Total | 0.9% | 2.8% | 2.3% | 7.9% |
|  | Preliminary | Count | 3 | 7 | 18 | 18 |
|  |  | % within candidate | 5.6% | 13.0% | 33.3% | 33.3% |
|  |  | % of Total | 1.4% | 3.3% | 8.4% | 8.4% |
| Total | | Count | 6 | 15 | 39 | 68 |
|  |  | % within candidate | 2.8% | 7.0% | 18.1% | 31.6% |
|  |  | % of Total | 2.8% | 7.0% | 18.1% | 31.6% |

| **Crosstab** | | | | |
| --- | --- | --- | --- | --- |
|  | | | lengthLT2 | Total |
|  |  |  | 5 |  |
| candidate | US categorical | Count | 44 | 96 |
|  |  | % within candidate | 45.8% | 100.0% |
|  |  | % of Total | 20.5% | 44.7% |
|  | IM categorical | Count | 35 | 65 |
|  |  | % within candidate | 53.8% | 100.0% |
|  |  | % of Total | 16.3% | 30.2% |
|  | Preliminary | Count | 8 | 54 |
|  |  | % within candidate | 14.8% | 100.0% |
|  |  | % of Total | 3.7% | 25.1% |
| Total | | Count | 87 | 215 |
|  |  | % within candidate | 40.5% | 100.0% |
|  |  | % of Total | 40.5% | 100.0% |

| **Chi-Square Tests** | | | |
| --- | --- | --- | --- |
|  | Value | df | Asymp. Sig. (2-sided) |
| Pearson Chi-Square | 33.225^a^ | 8 | .000 |
| Likelihood Ratio | 36.175 | 8 | .000 |
| Linear-by-Linear Association | 18.337 | 1 | .000 |
| N of Valid Cases | 215 |  |  |

| a. 5 cells (33.3%) have expected count less than 5. The minimum expected count is 1.51. |
| --- |

**candidate * length2**

| **Crosstab** | | | | | | |
| --- | --- | --- | --- | --- | --- | --- |
|  | | | length2 | | | |
|  |  |  | 1 | 2 | 3 | 4 |
| candidate | US categorical | Count | 6 | 27 | 34 | 29 |
|  |  | % within candidate | 6.3% | 28.1% | 35.4% | 30.2% |
|  |  | % of Total | 2.8% | 12.6% | 15.8% | 13.5% |
|  | IM categorical | Count | 9 | 12 | 21 | 23 |
|  |  | % within candidate | 13.8% | 18.5% | 32.3% | 35.4% |
|  |  | % of Total | 4.2% | 5.6% | 9.8% | 10.7% |
|  | Preliminary | Count | 16 | 22 | 14 | 2 |
|  |  | % within candidate | 29.6% | 40.7% | 25.9% | 3.7% |
|  |  | % of Total | 7.4% | 10.2% | 6.5% | 0.9% |
| Total | | Count | 31 | 61 | 69 | 54 |
|  |  | % within candidate | 14.4% | 28.4% | 32.1% | 25.1% |
|  |  | % of Total | 14.4% | 28.4% | 32.1% | 25.1% |

| **Crosstab** | | | |
| --- | --- | --- | --- |
|  | | | Total |
|  |  |  |  |
| candidate | US categorical | Count | 96 |
|  |  | % within candidate | 100.0% |
|  |  | % of Total | 44.7% |
|  | IM categorical | Count | 65 |
|  |  | % within candidate | 100.0% |
|  |  | % of Total | 30.2% |
|  | Preliminary | Count | 54 |
|  |  | % within candidate | 100.0% |
|  |  | % of Total | 25.1% |
| Total | | Count | 215 |
|  |  | % within candidate | 100.0% |
|  |  | % of Total | 100.0% |

| **Chi-Square Tests** | | | |
| --- | --- | --- | --- |
|  | Value | df | Asymp. Sig. (2-sided) |
| Pearson Chi-Square | 32.836^a^ | 6 | .000 |
| Likelihood Ratio | 37.245 | 6 | .000 |
| Linear-by-Linear Association | 21.421 | 1 | .000 |
| N of Valid Cases | 215 |  |  |

| a. 0 cells (0.0%) have expected count less than 5. The minimum expected count is 7.79. |
| --- |

**candidate * length4**

| **Crosstab** | | | | | | |
| --- | --- | --- | --- | --- | --- | --- |
|  | | | length4 | | | |
|  |  |  | 1 | 2 | 3 | 4 |
| candidate | US categorical | Count | 63 | 20 | 11 | 1 |
|  |  | % within candidate | 65.6% | 20.8% | 11.5% | 1.0% |
|  |  | % of Total | 29.3% | 9.3% | 5.1% | 0.5% |
|  | IM categorical | Count | 32 | 20 | 11 | 1 |
|  |  | % within candidate | 49.2% | 30.8% | 16.9% | 1.5% |
|  |  | % of Total | 14.9% | 9.3% | 5.1% | 0.5% |
|  | Preliminary | Count | 33 | 10 | 11 | 0 |
|  |  | % within candidate | 61.1% | 18.5% | 20.4% | 0.0% |
|  |  | % of Total | 15.3% | 4.7% | 5.1% | 0.0% |
| Total | | Count | 128 | 50 | 33 | 2 |
|  |  | % within candidate | 59.5% | 23.3% | 15.3% | 0.9% |
|  |  | % of Total | 59.5% | 23.3% | 15.3% | 0.9% |

| **Crosstab** | | | | |
| --- | --- | --- | --- | --- |
|  | | | length4 | Total |
|  |  |  | 5 |  |
| candidate | US categorical | Count | 1 | 96 |
|  |  | % within candidate | 1.0% | 100.0% |
|  |  | % of Total | 0.5% | 44.7% |
|  | IM categorical | Count | 1 | 65 |
|  |  | % within candidate | 1.5% | 100.0% |
|  |  | % of Total | 0.5% | 30.2% |
|  | Preliminary | Count | 0 | 54 |
|  |  | % within candidate | 0.0% | 100.0% |
|  |  | % of Total | 0.0% | 25.1% |
| Total | | Count | 2 | 215 |
|  |  | % within candidate | 0.9% | 100.0% |
|  |  | % of Total | 0.9% | 100.0% |

| **Chi-Square Tests** | | | |
| --- | --- | --- | --- |
|  | Value | df | Asymp. Sig. (2-sided) |
| Pearson Chi-Square | 7.607^a^ | 8 | .473 |
| Likelihood Ratio | 8.485 | 8 | .388 |
| Linear-by-Linear Association | .693 | 1 | .405 |
| N of Valid Cases | 215 |  |  |

| a. 6 cells (40.0%) have expected count less than 5. The minimum expected count is .50. |
| --- |

**candidate * length6**

| **Crosstab** | | | | | | |
| --- | --- | --- | --- | --- | --- | --- |
|  | | | length6 | | | |
|  |  |  | 1 | 2 | 3 | 4 |
| candidate | US categorical | Count | 24 | 39 | 16 | 16 |
|  |  | % within candidate | 25.0% | 40.6% | 16.7% | 16.7% |
|  |  | % of Total | 11.2% | 18.1% | 7.4% | 7.4% |
|  | IM categorical | Count | 18 | 23 | 14 | 9 |
|  |  | % within candidate | 27.7% | 35.4% | 21.5% | 13.8% |
|  |  | % of Total | 8.4% | 10.7% | 6.5% | 4.2% |
|  | Preliminary | Count | 2 | 14 | 10 | 28 |
|  |  | % within candidate | 3.7% | 25.9% | 18.5% | 51.9% |
|  |  | % of Total | 0.9% | 6.5% | 4.7% | 13.0% |
| Total | | Count | 44 | 76 | 40 | 53 |
|  |  | % within candidate | 20.5% | 35.3% | 18.6% | 24.7% |
|  |  | % of Total | 20.5% | 35.3% | 18.6% | 24.7% |

| **Crosstab** | | | | |
| --- | --- | --- | --- | --- |
|  | | | length6 | Total |
|  |  |  | 5 |  |
| candidate | US categorical | Count | 1 | 96 |
|  |  | % within candidate | 1.0% | 100.0% |
|  |  | % of Total | 0.5% | 44.7% |
|  | IM categorical | Count | 1 | 65 |
|  |  | % within candidate | 1.5% | 100.0% |
|  |  | % of Total | 0.5% | 30.2% |
|  | Preliminary | Count | 0 | 54 |
|  |  | % within candidate | 0.0% | 100.0% |
|  |  | % of Total | 0.0% | 25.1% |
| Total | | Count | 2 | 215 |
|  |  | % within candidate | 0.9% | 100.0% |
|  |  | % of Total | 0.9% | 100.0% |

| **Chi-Square Tests** | | | |
| --- | --- | --- | --- |
|  | Value | df | Asymp. Sig. (2-sided) |
| Pearson Chi-Square | 35.186^a^ | 8 | .000 |
| Likelihood Ratio | 36.326 | 8 | .000 |
| Linear-by-Linear Association | 19.612 | 1 | .000 |
| N of Valid Cases | 215 |  |  |

| a. 3 cells (20.0%) have expected count less than 5. The minimum expected count is .50. |
| --- |

**candidate * length8**

| **Crosstab** | | | | | | |
| --- | --- | --- | --- | --- | --- | --- |
|  | | | length8 | | | |
|  |  |  | 1 | 2 | 3 | 4 |
| candidate | US categorical | Count | 2 | 8 | 19 | 17 |
|  |  | % within candidate | 2.1% | 8.3% | 19.8% | 17.7% |
|  |  | % of Total | 0.9% | 3.7% | 8.8% | 7.9% |
|  | IM categorical | Count | 4 | 4 | 14 | 15 |
|  |  | % within candidate | 6.2% | 6.2% | 21.5% | 23.1% |
|  |  | % of Total | 1.9% | 1.9% | 6.5% | 7.0% |
|  | Preliminary | Count | 0 | 1 | 1 | 6 |
|  |  | % within candidate | 0.0% | 1.9% | 1.9% | 11.1% |
|  |  | % of Total | 0.0% | 0.5% | 0.5% | 2.8% |
| Total | | Count | 6 | 13 | 34 | 38 |
|  |  | % within candidate | 2.8% | 6.0% | 15.8% | 17.7% |
|  |  | % of Total | 2.8% | 6.0% | 15.8% | 17.7% |

| **Crosstab** | | | | |
| --- | --- | --- | --- | --- |
|  | | | length8 | Total |
|  |  |  | 5 |  |
| candidate | US categorical | Count | 50 | 96 |
|  |  | % within candidate | 52.1% | 100.0% |
|  |  | % of Total | 23.3% | 44.7% |
|  | IM categorical | Count | 28 | 65 |
|  |  | % within candidate | 43.1% | 100.0% |
|  |  | % of Total | 13.0% | 30.2% |
|  | Preliminary | Count | 46 | 54 |
|  |  | % within candidate | 85.2% | 100.0% |
|  |  | % of Total | 21.4% | 25.1% |
| Total | | Count | 124 | 215 |
|  |  | % within candidate | 57.7% | 100.0% |
|  |  | % of Total | 57.7% | 100.0% |

| **Chi-Square Tests** | | | |
| --- | --- | --- | --- |
|  | Value | df | Asymp. Sig. (2-sided) |
| Pearson Chi-Square | 28.078^a^ | 8 | .000 |
| Likelihood Ratio | 32.789 | 8 | .000 |
| Linear-by-Linear Association | 10.972 | 1 | .001 |
| N of Valid Cases | 215 |  |  |

| a. 5 cells (33.3%) have expected count less than 5. The minimum expected count is 1.51. |
| --- |

**candidate * faculty1**

| **Crosstab** | | | | | | |
| --- | --- | --- | --- | --- | --- | --- |
|  | | | faculty1 | | | |
|  |  |  | 1 | 2 | 3 | 4 |
| candidate | US categorical | Count | 1 | 17 | 43 | 23 |
|  |  | % within candidate | 1.0% | 17.7% | 44.8% | 24.0% |
|  |  | % of Total | 0.5% | 7.9% | 20.0% | 10.7% |
|  | IM categorical | Count | 6 | 11 | 21 | 14 |
|  |  | % within candidate | 9.2% | 16.9% | 32.3% | 21.5% |
|  |  | % of Total | 2.8% | 5.1% | 9.8% | 6.5% |
|  | Preliminary | Count | 3 | 11 | 17 | 14 |
|  |  | % within candidate | 5.6% | 20.4% | 31.5% | 25.9% |
|  |  | % of Total | 1.4% | 5.1% | 7.9% | 6.5% |
| Total | | Count | 10 | 39 | 81 | 51 |
|  |  | % within candidate | 4.7% | 18.1% | 37.7% | 23.7% |
|  |  | % of Total | 4.7% | 18.1% | 37.7% | 23.7% |

| **Crosstab** | | | | |
| --- | --- | --- | --- | --- |
|  | | | faculty1 | Total |
|  |  |  | 5 |  |
| candidate | US categorical | Count | 12 | 96 |
|  |  | % within candidate | 12.5% | 100.0% |
|  |  | % of Total | 5.6% | 44.7% |
|  | IM categorical | Count | 13 | 65 |
|  |  | % within candidate | 20.0% | 100.0% |
|  |  | % of Total | 6.0% | 30.2% |
|  | Preliminary | Count | 9 | 54 |
|  |  | % within candidate | 16.7% | 100.0% |
|  |  | % of Total | 4.2% | 25.1% |
| Total | | Count | 34 | 215 |
|  |  | % within candidate | 15.8% | 100.0% |
|  |  | % of Total | 15.8% | 100.0% |

| **Chi-Square Tests** | | | |
| --- | --- | --- | --- |
|  | Value | df | Asymp. Sig. (2-sided) |
| Pearson Chi-Square | 9.919^a^ | 8 | .271 |
| Likelihood Ratio | 10.478 | 8 | .233 |
| Linear-by-Linear Association | .010 | 1 | .921 |
| N of Valid Cases | 215 |  |  |

| a. 3 cells (20.0%) have expected count less than 5. The minimum expected count is 2.51. |
| --- |

**candidate * faculty2**

| **Crosstab** | | | | | | |
| --- | --- | --- | --- | --- | --- | --- |
|  | | | faculty2 | | | |
|  |  |  | 1 | 2 | 3 | 4 |
| candidate | US categorical | Count | 73 | 17 | 2 | 2 |
|  |  | % within candidate | 76.0% | 17.7% | 2.1% | 2.1% |
|  |  | % of Total | 34.0% | 7.9% | 0.9% | 0.9% |
|  | IM categorical | Count | 40 | 15 | 5 | 3 |
|  |  | % within candidate | 61.5% | 23.1% | 7.7% | 4.6% |
|  |  | % of Total | 18.6% | 7.0% | 2.3% | 1.4% |
|  | Preliminary | Count | 34 | 16 | 2 | 2 |
|  |  | % within candidate | 63.0% | 29.6% | 3.7% | 3.7% |
|  |  | % of Total | 15.8% | 7.4% | 0.9% | 0.9% |
| Total | | Count | 147 | 48 | 9 | 7 |
|  |  | % within candidate | 68.4% | 22.3% | 4.2% | 3.3% |
|  |  | % of Total | 68.4% | 22.3% | 4.2% | 3.3% |

| **Crosstab** | | | | |
| --- | --- | --- | --- | --- |
|  | | | faculty2 | Total |
|  |  |  | 5 |  |
| candidate | US categorical | Count | 2 | 96 |
|  |  | % within candidate | 2.1% | 100.0% |
|  |  | % of Total | 0.9% | 44.7% |
|  | IM categorical | Count | 2 | 65 |
|  |  | % within candidate | 3.1% | 100.0% |
|  |  | % of Total | 0.9% | 30.2% |
|  | Preliminary | Count | 0 | 54 |
|  |  | % within candidate | 0.0% | 100.0% |
|  |  | % of Total | 0.0% | 25.1% |
| Total | | Count | 4 | 215 |
|  |  | % within candidate | 1.9% | 100.0% |
|  |  | % of Total | 1.9% | 100.0% |

| **Chi-Square Tests** | | | |
| --- | --- | --- | --- |
|  | Value | df | Asymp. Sig. (2-sided) |
| Pearson Chi-Square | 9.032^a^ | 8 | .340 |
| Likelihood Ratio | 9.758 | 8 | .282 |
| Linear-by-Linear Association | 1.155 | 1 | .283 |
| N of Valid Cases | 215 |  |  |

| a. 9 cells (60.0%) have expected count less than 5. The minimum expected count is 1.00. |
| --- |

**candidate * faculty3**

| **Crosstab** | | | | | | |
| --- | --- | --- | --- | --- | --- | --- |
|  | | | faculty3 | | | |
|  |  |  | 1 | 2 | 3 | 4 |
| candidate | US categorical | Count | 19 | 56 | 19 | 2 |
|  |  | % within candidate | 19.8% | 58.3% | 19.8% | 2.1% |
|  |  | % of Total | 8.8% | 26.0% | 8.8% | 0.9% |
|  | IM categorical | Count | 14 | 33 | 18 | 0 |
|  |  | % within candidate | 21.5% | 50.8% | 27.7% | 0.0% |
|  |  | % of Total | 6.5% | 15.3% | 8.4% | 0.0% |
|  | Preliminary | Count | 17 | 23 | 14 | 0 |
|  |  | % within candidate | 31.5% | 42.6% | 25.9% | 0.0% |
|  |  | % of Total | 7.9% | 10.7% | 6.5% | 0.0% |
| Total | | Count | 50 | 112 | 51 | 2 |
|  |  | % within candidate | 23.3% | 52.1% | 23.7% | 0.9% |
|  |  | % of Total | 23.3% | 52.1% | 23.7% | 0.9% |

| **Crosstab** | | | |
| --- | --- | --- | --- |
|  | | | Total |
|  |  |  |  |
| candidate | US categorical | Count | 96 |
|  |  | % within candidate | 100.0% |
|  |  | % of Total | 44.7% |
|  | IM categorical | Count | 65 |
|  |  | % within candidate | 100.0% |
|  |  | % of Total | 30.2% |
|  | Preliminary | Count | 54 |
|  |  | % within candidate | 100.0% |
|  |  | % of Total | 25.1% |
| Total | | Count | 215 |
|  |  | % within candidate | 100.0% |
|  |  | % of Total | 100.0% |

| **Chi-Square Tests** | | | |
| --- | --- | --- | --- |
|  | Value | df | Asymp. Sig. (2-sided) |
| Pearson Chi-Square | 7.471^a^ | 6 | .279 |
| Likelihood Ratio | 8.135 | 6 | .228 |
| Linear-by-Linear Association | .503 | 1 | .478 |
| N of Valid Cases | 215 |  |  |

| a. 3 cells (25.0%) have expected count less than 5. The minimum expected count is .50. |
| --- |

**candidate * faculty4**

| **Crosstab** | | | | | | |
| --- | --- | --- | --- | --- | --- | --- |
|  | | | faculty4 | | | |
|  |  |  | 1 | 2 | 3 | 4 |
| candidate | US categorical | Count | 1 | 5 | 31 | 59 |
|  |  | % within candidate | 1.0% | 5.2% | 32.3% | 61.5% |
|  |  | % of Total | 0.5% | 2.3% | 14.4% | 27.4% |
|  | IM categorical | Count | 3 | 6 | 18 | 38 |
|  |  | % within candidate | 4.6% | 9.2% | 27.7% | 58.5% |
|  |  | % of Total | 1.4% | 2.8% | 8.4% | 17.7% |
|  | Preliminary | Count | 0 | 4 | 19 | 31 |
|  |  | % within candidate | 0.0% | 7.4% | 35.2% | 57.4% |
|  |  | % of Total | 0.0% | 1.9% | 8.8% | 14.4% |
| Total | | Count | 4 | 15 | 68 | 128 |
|  |  | % within candidate | 1.9% | 7.0% | 31.6% | 59.5% |
|  |  | % of Total | 1.9% | 7.0% | 31.6% | 59.5% |

| **Crosstab** | | | |
| --- | --- | --- | --- |
|  | | | Total |
|  |  |  |  |
| candidate | US categorical | Count | 96 |
|  |  | % within candidate | 100.0% |
|  |  | % of Total | 44.7% |
|  | IM categorical | Count | 65 |
|  |  | % within candidate | 100.0% |
|  |  | % of Total | 30.2% |
|  | Preliminary | Count | 54 |
|  |  | % within candidate | 100.0% |
|  |  | % of Total | 25.1% |
| Total | | Count | 215 |
|  |  | % within candidate | 100.0% |
|  |  | % of Total | 100.0% |

| **Chi-Square Tests** | | | |
| --- | --- | --- | --- |
|  | Value | df | Asymp. Sig. (2-sided) |
| Pearson Chi-Square | 5.581^a^ | 6 | .472 |
| Likelihood Ratio | 5.874 | 6 | .437 |
| Linear-by-Linear Association | .282 | 1 | .595 |
| N of Valid Cases | 215 |  |  |

| a. 5 cells (41.7%) have expected count less than 5. The minimum expected count is 1.00. |
| --- |

**candidate * facultyGT4**

| **Crosstab** | | | | | | |
| --- | --- | --- | --- | --- | --- | --- |
|  | | | facultyGT4 | | | |
|  |  |  | 1 | 2 | 3 | 4 |
| candidate | US categorical | Count | 2 | 1 | 1 | 10 |
|  |  | % within candidate | 2.1% | 1.0% | 1.0% | 10.4% |
|  |  | % of Total | 0.9% | 0.5% | 0.5% | 4.7% |
|  | IM categorical | Count | 2 | 0 | 3 | 10 |
|  |  | % within candidate | 3.1% | 0.0% | 4.6% | 15.4% |
|  |  | % of Total | 0.9% | 0.0% | 1.4% | 4.7% |
|  | Preliminary | Count | 0 | 0 | 2 | 7 |
|  |  | % within candidate | 0.0% | 0.0% | 3.7% | 13.0% |
|  |  | % of Total | 0.0% | 0.0% | 0.9% | 3.3% |
| Total | | Count | 4 | 1 | 6 | 27 |
|  |  | % within candidate | 1.9% | 0.5% | 2.8% | 12.6% |
|  |  | % of Total | 1.9% | 0.5% | 2.8% | 12.6% |

| **Crosstab** | | | | |
| --- | --- | --- | --- | --- |
|  | | | facultyGT4 | Total |
|  |  |  | 5 |  |
| candidate | US categorical | Count | 82 | 96 |
|  |  | % within candidate | 85.4% | 100.0% |
|  |  | % of Total | 38.1% | 44.7% |
|  | IM categorical | Count | 50 | 65 |
|  |  | % within candidate | 76.9% | 100.0% |
|  |  | % of Total | 23.3% | 30.2% |
|  | Preliminary | Count | 45 | 54 |
|  |  | % within candidate | 83.3% | 100.0% |
|  |  | % of Total | 20.9% | 25.1% |
| Total | | Count | 177 | 215 |
|  |  | % within candidate | 82.3% | 100.0% |
|  |  | % of Total | 82.3% | 100.0% |

| **Chi-Square Tests** | | | |
| --- | --- | --- | --- |
|  | Value | df | Asymp. Sig. (2-sided) |
| Pearson Chi-Square | 5.896^a^ | 8 | .659 |
| Likelihood Ratio | 7.375 | 8 | .497 |
| Linear-by-Linear Association | .006 | 1 | .937 |
| N of Valid Cases | 215 |  |  |

| a. 9 cells (60.0%) have expected count less than 5. The minimum expected count is .25. |
| --- |

**candidate * lengthLT15**

| **Crosstab** | | | | | | |
| --- | --- | --- | --- | --- | --- | --- |
|  | | | lengthLT15 | | | |
|  |  |  | 1 | 2 | 3 | 4 |
| candidate | US categorical | Count | 2 | 32 | 35 | 15 |
|  |  | % within candidate | 2.1% | 33.3% | 36.5% | 15.6% |
|  |  | % of Total | 0.9% | 14.9% | 16.3% | 7.0% |
|  | IM categorical | Count | 10 | 33 | 15 | 3 |
|  |  | % within candidate | 15.4% | 50.8% | 23.1% | 4.6% |
|  |  | % of Total | 4.7% | 15.3% | 7.0% | 1.4% |
|  | Preliminary | Count | 10 | 33 | 10 | 1 |
|  |  | % within candidate | 18.5% | 61.1% | 18.5% | 1.9% |
|  |  | % of Total | 4.7% | 15.3% | 4.7% | 0.5% |
| Total | | Count | 22 | 98 | 60 | 19 |
|  |  | % within candidate | 10.2% | 45.6% | 27.9% | 8.8% |
|  |  | % of Total | 10.2% | 45.6% | 27.9% | 8.8% |

| **Crosstab** | | | | |
| --- | --- | --- | --- | --- |
|  | | | lengthLT15 | Total |
|  |  |  | 5 |  |
| candidate | US categorical | Count | 12 | 96 |
|  |  | % within candidate | 12.5% | 100.0% |
|  |  | % of Total | 5.6% | 44.7% |
|  | IM categorical | Count | 4 | 65 |
|  |  | % within candidate | 6.2% | 100.0% |
|  |  | % of Total | 1.9% | 30.2% |
|  | Preliminary | Count | 0 | 54 |
|  |  | % within candidate | 0.0% | 100.0% |
|  |  | % of Total | 0.0% | 25.1% |
| Total | | Count | 16 | 215 |
|  |  | % within candidate | 7.4% | 100.0% |
|  |  | % of Total | 7.4% | 100.0% |

| **Chi-Square Tests** | | | |
| --- | --- | --- | --- |
|  | Value | df | Asymp. Sig. (2-sided) |
| Pearson Chi-Square | 39.466^a^ | 8 | .000 |
| Likelihood Ratio | 45.897 | 8 | .000 |
| Linear-by-Linear Association | 34.681 | 1 | .000 |
| N of Valid Cases | 215 |  |  |

| a. 3 cells (20.0%) have expected count less than 5. The minimum expected count is 4.02. |
| --- |

**candidate * length15_30**

| **Crosstab** | | | | | | |
| --- | --- | --- | --- | --- | --- | --- |
|  | | | length15_30 | | | |
|  |  |  | 1 | 2 | 3 | 4 |
| candidate | US categorical | Count | 75 | 15 | 4 | 1 |
|  |  | % within candidate | 78.1% | 15.6% | 4.2% | 1.0% |
|  |  | % of Total | 34.9% | 7.0% | 1.9% | 0.5% |
|  | IM categorical | Count | 48 | 13 | 1 | 3 |
|  |  | % within candidate | 73.8% | 20.0% | 1.5% | 4.6% |
|  |  | % of Total | 22.3% | 6.0% | 0.5% | 1.4% |
|  | Preliminary | Count | 43 | 11 | 0 | 0 |
|  |  | % within candidate | 79.6% | 20.4% | 0.0% | 0.0% |
|  |  | % of Total | 20.0% | 5.1% | 0.0% | 0.0% |
| Total | | Count | 166 | 39 | 5 | 4 |
|  |  | % within candidate | 77.2% | 18.1% | 2.3% | 1.9% |
|  |  | % of Total | 77.2% | 18.1% | 2.3% | 1.9% |

| **Crosstab** | | | | |
| --- | --- | --- | --- | --- |
|  | | | length15_30 | Total |
|  |  |  | 5 |  |
| candidate | US categorical | Count | 1 | 96 |
|  |  | % within candidate | 1.0% | 100.0% |
|  |  | % of Total | 0.5% | 44.7% |
|  | IM categorical | Count | 0 | 65 |
|  |  | % within candidate | 0.0% | 100.0% |
|  |  | % of Total | 0.0% | 30.2% |
|  | Preliminary | Count | 0 | 54 |
|  |  | % within candidate | 0.0% | 100.0% |
|  |  | % of Total | 0.0% | 25.1% |
| Total | | Count | 1 | 215 |
|  |  | % within candidate | 0.5% | 100.0% |
|  |  | % of Total | 0.5% | 100.0% |

| **Chi-Square Tests** | | | |
| --- | --- | --- | --- |
|  | Value | df | Asymp. Sig. (2-sided) |
| Pearson Chi-Square | 8.823^a^ | 8 | .357 |
| Likelihood Ratio | 10.504 | 8 | .231 |
| Linear-by-Linear Association | .656 | 1 | .418 |
| N of Valid Cases | 215 |  |  |

| a. 9 cells (60.0%) have expected count less than 5. The minimum expected count is .25. |
| --- |

**candidate * length30_45**

| **Crosstab** | | | | | | |
| --- | --- | --- | --- | --- | --- | --- |
|  | | | length30_45 | | | |
|  |  |  | 1 | 2 | 3 | 4 |
| candidate | US categorical | Count | 17 | 43 | 35 | 1 |
|  |  | % within candidate | 17.7% | 44.8% | 36.5% | 1.0% |
|  |  | % of Total | 7.9% | 20.0% | 16.3% | 0.5% |
|  | IM categorical | Count | 3 | 18 | 44 | 0 |
|  |  | % within candidate | 4.6% | 27.7% | 67.7% | 0.0% |
|  |  | % of Total | 1.4% | 8.4% | 20.5% | 0.0% |
|  | Preliminary | Count | 1 | 10 | 43 | 0 |
|  |  | % within candidate | 1.9% | 18.5% | 79.6% | 0.0% |
|  |  | % of Total | 0.5% | 4.7% | 20.0% | 0.0% |
| Total | | Count | 21 | 71 | 122 | 1 |
|  |  | % within candidate | 9.8% | 33.0% | 56.7% | 0.5% |
|  |  | % of Total | 9.8% | 33.0% | 56.7% | 0.5% |

| **Crosstab** | | | |
| --- | --- | --- | --- |
|  | | | Total |
|  |  |  |  |
| candidate | US categorical | Count | 96 |
|  |  | % within candidate | 100.0% |
|  |  | % of Total | 44.7% |
|  | IM categorical | Count | 65 |
|  |  | % within candidate | 100.0% |
|  |  | % of Total | 30.2% |
|  | Preliminary | Count | 54 |
|  |  | % within candidate | 100.0% |
|  |  | % of Total | 25.1% |
| Total | | Count | 215 |
|  |  | % within candidate | 100.0% |
|  |  | % of Total | 100.0% |

| **Chi-Square Tests** | | | |
| --- | --- | --- | --- |
|  | Value | df | Asymp. Sig. (2-sided) |
| Pearson Chi-Square | 34.013^a^ | 6 | .000 |
| Likelihood Ratio | 36.023 | 6 | .000 |
| Linear-by-Linear Association | 27.419 | 1 | .000 |
| N of Valid Cases | 215 |  |  |

| a. 3 cells (25.0%) have expected count less than 5. The minimum expected count is .25. |
| --- |

**candidate * length45_60**

| **Crosstab** | | | | | | |
| --- | --- | --- | --- | --- | --- | --- |
|  | | | length45_60 | | | |
|  |  |  | 1 | 2 | 3 | 4 |
| candidate | US categorical | Count | 0 | 6 | 22 | 68 |
|  |  | % within candidate | 0.0% | 6.3% | 22.9% | 70.8% |
|  |  | % of Total | 0.0% | 2.8% | 10.2% | 31.6% |
|  | IM categorical | Count | 3 | 0 | 5 | 57 |
|  |  | % within candidate | 4.6% | 0.0% | 7.7% | 87.7% |
|  |  | % of Total | 1.4% | 0.0% | 2.3% | 26.5% |
|  | Preliminary | Count | 0 | 0 | 1 | 53 |
|  |  | % within candidate | 0.0% | 0.0% | 1.9% | 98.1% |
|  |  | % of Total | 0.0% | 0.0% | 0.5% | 24.7% |
| Total | | Count | 3 | 6 | 28 | 178 |
|  |  | % within candidate | 1.4% | 2.8% | 13.0% | 82.8% |
|  |  | % of Total | 1.4% | 2.8% | 13.0% | 82.8% |

| **Crosstab** | | | |
| --- | --- | --- | --- |
|  | | | Total |
|  |  |  |  |
| candidate | US categorical | Count | 96 |
|  |  | % within candidate | 100.0% |
|  |  | % of Total | 44.7% |
|  | IM categorical | Count | 65 |
|  |  | % within candidate | 100.0% |
|  |  | % of Total | 30.2% |
|  | Preliminary | Count | 54 |
|  |  | % within candidate | 100.0% |
|  |  | % of Total | 25.1% |
| Total | | Count | 215 |
|  |  | % within candidate | 100.0% |
|  |  | % of Total | 100.0% |

| **Chi-Square Tests** | | | |
| --- | --- | --- | --- |
|  | Value | df | Asymp. Sig. (2-sided) |
| Pearson Chi-Square | 31.554^a^ | 6 | .000 |
| Likelihood Ratio | 35.933 | 6 | .000 |
| Linear-by-Linear Association | 12.212 | 1 | .000 |
| N of Valid Cases | 215 |  |  |

| a. 6 cells (50.0%) have expected count less than 5. The minimum expected count is .75. |
| --- |

**candidate * lengthGT60**

| **Crosstab** | | | | | | |
| --- | --- | --- | --- | --- | --- | --- |
|  | | | lengthGT60 | | | |
|  |  |  | 1 | 2 | 4 | 5 |
| candidate | US categorical | Count | 2 | 0 | 11 | 83 |
|  |  | % within candidate | 2.1% | 0.0% | 11.5% | 86.5% |
|  |  | % of Total | 0.9% | 0.0% | 5.1% | 38.6% |
|  | IM categorical | Count | 2 | 1 | 2 | 60 |
|  |  | % within candidate | 3.1% | 1.5% | 3.1% | 92.3% |
|  |  | % of Total | 0.9% | 0.5% | 0.9% | 27.9% |
|  | Preliminary | Count | 0 | 0 | 0 | 54 |
|  |  | % within candidate | 0.0% | 0.0% | 0.0% | 100.0% |
|  |  | % of Total | 0.0% | 0.0% | 0.0% | 25.1% |
| Total | | Count | 4 | 1 | 13 | 197 |
|  |  | % within candidate | 1.9% | 0.5% | 6.0% | 91.6% |
|  |  | % of Total | 1.9% | 0.5% | 6.0% | 91.6% |

| **Crosstab** | | | |
| --- | --- | --- | --- |
|  | | | Total |
|  |  |  |  |
| candidate | US categorical | Count | 96 |
|  |  | % within candidate | 100.0% |
|  |  | % of Total | 44.7% |
|  | IM categorical | Count | 65 |
|  |  | % within candidate | 100.0% |
|  |  | % of Total | 30.2% |
|  | Preliminary | Count | 54 |
|  |  | % within candidate | 100.0% |
|  |  | % of Total | 25.1% |
| Total | | Count | 215 |
|  |  | % within candidate | 100.0% |
|  |  | % of Total | 100.0% |

| **Chi-Square Tests** | | | |
| --- | --- | --- | --- |
|  | Value | df | Asymp. Sig. (2-sided) |
| Pearson Chi-Square | 13.414^a^ | 6 | .037 |
| Likelihood Ratio | 16.908 | 6 | .010 |
| Linear-by-Linear Association | 3.020 | 1 | .082 |
| N of Valid Cases | 215 |  |  |

| a. 8 cells (66.7%) have expected count less than 5. The minimum expected count is .25. |
| --- |

**candidate * INT_res_chief**

| **Crosstab** | | | | | | |
| --- | --- | --- | --- | --- | --- | --- |
|  | | | INT_res_chief | | | |
|  |  |  | 1 | 2 | 3 | 4 |
| candidate | US categorical | Count | 3 | 15 | 13 | 37 |
|  |  | % within candidate | 3.1% | 15.6% | 13.5% | 38.5% |
|  |  | % of Total | 1.4% | 7.0% | 6.0% | 17.2% |
|  | IM categorical | Count | 2 | 3 | 8 | 17 |
|  |  | % within candidate | 3.1% | 4.6% | 12.3% | 26.2% |
|  |  | % of Total | 0.9% | 1.4% | 3.7% | 7.9% |
|  | Preliminary | Count | 2 | 3 | 12 | 17 |
|  |  | % within candidate | 3.7% | 5.6% | 22.2% | 31.5% |
|  |  | % of Total | 0.9% | 1.4% | 5.6% | 7.9% |
| Total | | Count | 7 | 21 | 33 | 71 |
|  |  | % within candidate | 3.3% | 9.8% | 15.3% | 33.0% |
|  |  | % of Total | 3.3% | 9.8% | 15.3% | 33.0% |

| **Crosstab** | | | | |
| --- | --- | --- | --- | --- |
|  | | | INT_res_chief | Total |
|  |  |  | 5 |  |
| candidate | US categorical | Count | 28 | 96 |
|  |  | % within candidate | 29.2% | 100.0% |
|  |  | % of Total | 13.0% | 44.7% |
|  | IM categorical | Count | 35 | 65 |
|  |  | % within candidate | 53.8% | 100.0% |
|  |  | % of Total | 16.3% | 30.2% |
|  | Preliminary | Count | 20 | 54 |
|  |  | % within candidate | 37.0% | 100.0% |
|  |  | % of Total | 9.3% | 25.1% |
| Total | | Count | 83 | 215 |
|  |  | % within candidate | 38.6% | 100.0% |
|  |  | % of Total | 38.6% | 100.0% |

| **Chi-Square Tests** | | | |
| --- | --- | --- | --- |
|  | Value | df | Asymp. Sig. (2-sided) |
| Pearson Chi-Square | 16.436^a^ | 8 | .037 |
| Likelihood Ratio | 16.161 | 8 | .040 |
| Linear-by-Linear Association | 1.755 | 1 | .185 |
| N of Valid Cases | 215 |  |  |

| a. 3 cells (20.0%) have expected count less than 5. The minimum expected count is 1.76. |
| --- |

**candidate * INT_faculty**

| **Crosstab** | | | | | | |
| --- | --- | --- | --- | --- | --- | --- |
|  | | | INT_faculty | | | |
|  |  |  | 1 | 2 | 3 | 4 |
| candidate | US categorical | Count | 17 | 6 | 39 | 31 |
|  |  | % within candidate | 17.7% | 6.3% | 40.6% | 32.3% |
|  |  | % of Total | 7.9% | 2.8% | 18.1% | 14.4% |
|  | IM categorical | Count | 4 | 4 | 17 | 33 |
|  |  | % within candidate | 6.2% | 6.2% | 26.2% | 50.8% |
|  |  | % of Total | 1.9% | 1.9% | 7.9% | 15.3% |
|  | Preliminary | Count | 6 | 7 | 15 | 22 |
|  |  | % within candidate | 11.1% | 13.0% | 27.8% | 40.7% |
|  |  | % of Total | 2.8% | 3.3% | 7.0% | 10.2% |
| Total | | Count | 27 | 17 | 71 | 86 |
|  |  | % within candidate | 12.6% | 7.9% | 33.0% | 40.0% |
|  |  | % of Total | 12.6% | 7.9% | 33.0% | 40.0% |

| **Crosstab** | | | | |
| --- | --- | --- | --- | --- |
|  | | | INT_faculty | Total |
|  |  |  | 5 |  |
| candidate | US categorical | Count | 3 | 96 |
|  |  | % within candidate | 3.1% | 100.0% |
|  |  | % of Total | 1.4% | 44.7% |
|  | IM categorical | Count | 7 | 65 |
|  |  | % within candidate | 10.8% | 100.0% |
|  |  | % of Total | 3.3% | 30.2% |
|  | Preliminary | Count | 4 | 54 |
|  |  | % within candidate | 7.4% | 100.0% |
|  |  | % of Total | 1.9% | 25.1% |
| Total | | Count | 14 | 215 |
|  |  | % within candidate | 6.5% | 100.0% |
|  |  | % of Total | 6.5% | 100.0% |

| **Chi-Square Tests** | | | |
| --- | --- | --- | --- |
|  | Value | df | Asymp. Sig. (2-sided) |
| Pearson Chi-Square | 16.516^a^ | 8 | .036 |
| Likelihood Ratio | 16.583 | 8 | .035 |
| Linear-by-Linear Association | 2.931 | 1 | .087 |
| N of Valid Cases | 215 |  |  |

| a. 3 cells (20.0%) have expected count less than 5. The minimum expected count is 3.52. |
| --- |

**candidate * INT_APD**

| **Crosstab** | | | | | | |
| --- | --- | --- | --- | --- | --- | --- |
|  | | | INT_APD | | | |
|  |  |  | 1 | 2 | 3 | 4 |
| candidate | US categorical | Count | 11 | 56 | 24 | 5 |
|  |  | % within candidate | 11.5% | 58.3% | 25.0% | 5.2% |
|  |  | % of Total | 5.1% | 26.0% | 11.2% | 2.3% |
|  | IM categorical | Count | 2 | 32 | 24 | 6 |
|  |  | % within candidate | 3.1% | 49.2% | 36.9% | 9.2% |
|  |  | % of Total | 0.9% | 14.9% | 11.2% | 2.8% |
|  | Preliminary | Count | 5 | 32 | 12 | 5 |
|  |  | % within candidate | 9.3% | 59.3% | 22.2% | 9.3% |
|  |  | % of Total | 2.3% | 14.9% | 5.6% | 2.3% |
| Total | | Count | 18 | 120 | 60 | 16 |
|  |  | % within candidate | 8.4% | 55.8% | 27.9% | 7.4% |
|  |  | % of Total | 8.4% | 55.8% | 27.9% | 7.4% |

| **Crosstab** | | | | |
| --- | --- | --- | --- | --- |
|  | | | INT_APD | Total |
|  |  |  | 5 |  |
| candidate | US categorical | Count | 0 | 96 |
|  |  | % within candidate | 0.0% | 100.0% |
|  |  | % of Total | 0.0% | 44.7% |
|  | IM categorical | Count | 1 | 65 |
|  |  | % within candidate | 1.5% | 100.0% |
|  |  | % of Total | 0.5% | 30.2% |
|  | Preliminary | Count | 0 | 54 |
|  |  | % within candidate | 0.0% | 100.0% |
|  |  | % of Total | 0.0% | 25.1% |
| Total | | Count | 1 | 215 |
|  |  | % within candidate | 0.5% | 100.0% |
|  |  | % of Total | 0.5% | 100.0% |

| **Chi-Square Tests** | | | |
| --- | --- | --- | --- |
|  | Value | df | Asymp. Sig. (2-sided) |
| Pearson Chi-Square | 10.328^a^ | 8 | .243 |
| Likelihood Ratio | 10.938 | 8 | .205 |
| Linear-by-Linear Association | 1.000 | 1 | .317 |
| N of Valid Cases | 215 |  |  |

| a. 6 cells (40.0%) have expected count less than 5. The minimum expected count is .25. |
| --- |

**candidate * INT_PD**

| **Crosstab** | | | | | | |
| --- | --- | --- | --- | --- | --- | --- |
|  | | | INT_PD | | | |
|  |  |  | 1 | 2 | 3 | 4 |
| candidate | US categorical | Count | 63 | 16 | 10 | 5 |
|  |  | % within candidate | 65.6% | 16.7% | 10.4% | 5.2% |
|  |  | % of Total | 29.3% | 7.4% | 4.7% | 2.3% |
|  | IM categorical | Count | 54 | 7 | 2 | 0 |
|  |  | % within candidate | 83.1% | 10.8% | 3.1% | 0.0% |
|  |  | % of Total | 25.1% | 3.3% | 0.9% | 0.0% |
|  | Preliminary | Count | 39 | 7 | 7 | 1 |
|  |  | % within candidate | 72.2% | 13.0% | 13.0% | 1.9% |
|  |  | % of Total | 18.1% | 3.3% | 3.3% | 0.5% |
| Total | | Count | 156 | 30 | 19 | 6 |
|  |  | % within candidate | 72.6% | 14.0% | 8.8% | 2.8% |
|  |  | % of Total | 72.6% | 14.0% | 8.8% | 2.8% |

| **Crosstab** | | | | |
| --- | --- | --- | --- | --- |
|  | | | INT_PD | Total |
|  |  |  | 5 |  |
| candidate | US categorical | Count | 2 | 96 |
|  |  | % within candidate | 2.1% | 100.0% |
|  |  | % of Total | 0.9% | 44.7% |
|  | IM categorical | Count | 2 | 65 |
|  |  | % within candidate | 3.1% | 100.0% |
|  |  | % of Total | 0.9% | 30.2% |
|  | Preliminary | Count | 0 | 54 |
|  |  | % within candidate | 0.0% | 100.0% |
|  |  | % of Total | 0.0% | 25.1% |
| Total | | Count | 4 | 215 |
|  |  | % within candidate | 1.9% | 100.0% |
|  |  | % of Total | 1.9% | 100.0% |

| **Chi-Square Tests** | | | |
| --- | --- | --- | --- |
|  | Value | df | Asymp. Sig. (2-sided) |
| Pearson Chi-Square | 11.939^a^ | 8 | .154 |
| Likelihood Ratio | 14.950 | 8 | .060 |
| Linear-by-Linear Association | 1.953 | 1 | .162 |
| N of Valid Cases | 215 |  |  |

| a. 7 cells (46.7%) have expected count less than 5. The minimum expected count is 1.00. |
| --- |

**candidate * INT_chair**

| **Crosstab** | | | | | | |
| --- | --- | --- | --- | --- | --- | --- |
|  | | | INT_chair | | | |
|  |  |  | 1 | 2 | 3 | 4 |
| candidate | US categorical | Count | 2 | 3 | 10 | 18 |
|  |  | % within candidate | 2.1% | 3.1% | 10.4% | 18.8% |
|  |  | % of Total | 0.9% | 1.4% | 4.7% | 8.4% |
|  | IM categorical | Count | 3 | 19 | 14 | 9 |
|  |  | % within candidate | 4.6% | 29.2% | 21.5% | 13.8% |
|  |  | % of Total | 1.4% | 8.8% | 6.5% | 4.2% |
|  | Preliminary | Count | 2 | 5 | 8 | 9 |
|  |  | % within candidate | 3.7% | 9.3% | 14.8% | 16.7% |
|  |  | % of Total | 0.9% | 2.3% | 3.7% | 4.2% |
| Total | | Count | 7 | 27 | 32 | 36 |
|  |  | % within candidate | 3.3% | 12.6% | 14.9% | 16.7% |
|  |  | % of Total | 3.3% | 12.6% | 14.9% | 16.7% |

| **Crosstab** | | | | |
| --- | --- | --- | --- | --- |
|  | | | INT_chair | Total |
|  |  |  | 5 |  |
| candidate | US categorical | Count | 63 | 96 |
|  |  | % within candidate | 65.6% | 100.0% |
|  |  | % of Total | 29.3% | 44.7% |
|  | IM categorical | Count | 20 | 65 |
|  |  | % within candidate | 30.8% | 100.0% |
|  |  | % of Total | 9.3% | 30.2% |
|  | Preliminary | Count | 30 | 54 |
|  |  | % within candidate | 55.6% | 100.0% |
|  |  | % of Total | 14.0% | 25.1% |
| Total | | Count | 113 | 215 |
|  |  | % within candidate | 52.6% | 100.0% |
|  |  | % of Total | 52.6% | 100.0% |

| **Chi-Square Tests** | | | |
| --- | --- | --- | --- |
|  | Value | df | Asymp. Sig. (2-sided) |
| Pearson Chi-Square | 35.326^a^ | 8 | .000 |
| Likelihood Ratio | 35.128 | 8 | .000 |
| Linear-by-Linear Association | 5.468 | 1 | .019 |
| N of Valid Cases | 215 |  |  |

| a. 3 cells (20.0%) have expected count less than 5. The minimum expected count is 1.76. |
| --- |

**candidate * Qstraightforward**

| **Crosstab** | | | | | | |
| --- | --- | --- | --- | --- | --- | --- |
|  | | | Qstraightforward | | | |
|  |  |  | 1 | 2 | 3 | 4 |
| candidate | US categorical | Count | 80 | 9 | 3 | 3 |
|  |  | % within candidate | 83.3% | 9.4% | 3.1% | 3.1% |
|  |  | % of Total | 37.2% | 4.2% | 1.4% | 1.4% |
|  | IM categorical | Count | 36 | 10 | 9 | 8 |
|  |  | % within candidate | 55.4% | 15.4% | 13.8% | 12.3% |
|  |  | % of Total | 16.7% | 4.7% | 4.2% | 3.7% |
|  | Preliminary | Count | 52 | 1 | 0 | 1 |
|  |  | % within candidate | 96.3% | 1.9% | 0.0% | 1.9% |
|  |  | % of Total | 24.2% | 0.5% | 0.0% | 0.5% |
| Total | | Count | 168 | 20 | 12 | 12 |
|  |  | % within candidate | 78.1% | 9.3% | 5.6% | 5.6% |
|  |  | % of Total | 78.1% | 9.3% | 5.6% | 5.6% |

| **Crosstab** | | | | |
| --- | --- | --- | --- | --- |
|  | | | Qstraightforward | Total |
|  |  |  | 5 |  |
| candidate | US categorical | Count | 1 | 96 |
|  |  | % within candidate | 1.0% | 100.0% |
|  |  | % of Total | 0.5% | 44.7% |
|  | IM categorical | Count | 2 | 65 |
|  |  | % within candidate | 3.1% | 100.0% |
|  |  | % of Total | 0.9% | 30.2% |
|  | Preliminary | Count | 0 | 54 |
|  |  | % within candidate | 0.0% | 100.0% |
|  |  | % of Total | 0.0% | 25.1% |
| Total | | Count | 3 | 215 |
|  |  | % within candidate | 1.4% | 100.0% |
|  |  | % of Total | 1.4% | 100.0% |

| **Chi-Square Tests** | | | |
| --- | --- | --- | --- |
|  | Value | df | Asymp. Sig. (2-sided) |
| Pearson Chi-Square | 34.541^a^ | 8 | .000 |
| Likelihood Ratio | 36.636 | 8 | .000 |
| Linear-by-Linear Association | .297 | 1 | .586 |
| N of Valid Cases | 215 |  |  |

| a. 7 cells (46.7%) have expected count less than 5. The minimum expected count is .75. |
| --- |

**candidate * Qbehavior**

| **Crosstab** | | | | | | |
| --- | --- | --- | --- | --- | --- | --- |
|  | | | Qbehavior | | | |
|  |  |  | 1 | 2 | 3 | 4 |
| candidate | US categorical | Count | 11 | 53 | 30 | 1 |
|  |  | % within candidate | 11.5% | 55.2% | 31.3% | 1.0% |
|  |  | % of Total | 5.1% | 24.7% | 14.0% | 0.5% |
|  | IM categorical | Count | 16 | 25 | 19 | 4 |
|  |  | % within candidate | 24.6% | 38.5% | 29.2% | 6.2% |
|  |  | % of Total | 7.4% | 11.6% | 8.8% | 1.9% |
|  | Preliminary | Count | 2 | 32 | 15 | 3 |
|  |  | % within candidate | 3.7% | 59.3% | 27.8% | 5.6% |
|  |  | % of Total | 0.9% | 14.9% | 7.0% | 1.4% |
| Total | | Count | 29 | 110 | 64 | 8 |
|  |  | % within candidate | 13.5% | 51.2% | 29.8% | 3.7% |
|  |  | % of Total | 13.5% | 51.2% | 29.8% | 3.7% |

| **Crosstab** | | | | |
| --- | --- | --- | --- | --- |
|  | | | Qbehavior | Total |
|  |  |  | 5 |  |
| candidate | US categorical | Count | 1 | 96 |
|  |  | % within candidate | 1.0% | 100.0% |
|  |  | % of Total | 0.5% | 44.7% |
|  | IM categorical | Count | 1 | 65 |
|  |  | % within candidate | 1.5% | 100.0% |
|  |  | % of Total | 0.5% | 30.2% |
|  | Preliminary | Count | 2 | 54 |
|  |  | % within candidate | 3.7% | 100.0% |
|  |  | % of Total | 0.9% | 25.1% |
| Total | | Count | 4 | 215 |
|  |  | % within candidate | 1.9% | 100.0% |
|  |  | % of Total | 1.9% | 100.0% |

| **Chi-Square Tests** | | | |
| --- | --- | --- | --- |
|  | Value | df | Asymp. Sig. (2-sided) |
| Pearson Chi-Square | 18.033^a^ | 8 | .021 |
| Likelihood Ratio | 18.939 | 8 | .015 |
| Linear-by-Linear Association | 1.875 | 1 | .171 |
| N of Valid Cases | 215 |  |  |

| a. 6 cells (40.0%) have expected count less than 5. The minimum expected count is 1.00. |
| --- |

**candidate * Qsituation**

| **Crosstab** | | | | | | |
| --- | --- | --- | --- | --- | --- | --- |
|  | | | Qsituation | | | |
|  |  |  | 1 | 2 | 3 | 4 |
| candidate | US categorical | Count | 2 | 30 | 56 | 6 |
|  |  | % within candidate | 2.1% | 31.3% | 58.3% | 6.3% |
|  |  | % of Total | 0.9% | 14.0% | 26.0% | 2.8% |
|  | IM categorical | Count | 9 | 25 | 22 | 7 |
|  |  | % within candidate | 13.8% | 38.5% | 33.8% | 10.8% |
|  |  | % of Total | 4.2% | 11.6% | 10.2% | 3.3% |
|  | Preliminary | Count | 0 | 15 | 28 | 9 |
|  |  | % within candidate | 0.0% | 27.8% | 51.9% | 16.7% |
|  |  | % of Total | 0.0% | 7.0% | 13.0% | 4.2% |
| Total | | Count | 11 | 70 | 106 | 22 |
|  |  | % within candidate | 5.1% | 32.6% | 49.3% | 10.2% |
|  |  | % of Total | 5.1% | 32.6% | 49.3% | 10.2% |

| **Crosstab** | | | | |
| --- | --- | --- | --- | --- |
|  | | | Qsituation | Total |
|  |  |  | 5 |  |
| candidate | US categorical | Count | 2 | 96 |
|  |  | % within candidate | 2.1% | 100.0% |
|  |  | % of Total | 0.9% | 44.7% |
|  | IM categorical | Count | 2 | 65 |
|  |  | % within candidate | 3.1% | 100.0% |
|  |  | % of Total | 0.9% | 30.2% |
|  | Preliminary | Count | 2 | 54 |
|  |  | % within candidate | 3.7% | 100.0% |
|  |  | % of Total | 0.9% | 25.1% |
| Total | | Count | 6 | 215 |
|  |  | % within candidate | 2.8% | 100.0% |
|  |  | % of Total | 2.8% | 100.0% |

| **Chi-Square Tests** | | | |
| --- | --- | --- | --- |
|  | Value | df | Asymp. Sig. (2-sided) |
| Pearson Chi-Square | 24.149^a^ | 8 | .002 |
| Likelihood Ratio | 24.480 | 8 | .002 |
| Linear-by-Linear Association | 1.190 | 1 | .275 |
| N of Valid Cases | 215 |  |  |

| a. 6 cells (40.0%) have expected count less than 5. The minimum expected count is 1.51. |
| --- |

**candidate * Qteaser**

| **Crosstab** | | | | | | |
| --- | --- | --- | --- | --- | --- | --- |
|  | | | Qteaser | | | |
|  |  |  | 1 | 2 | 3 | 4 |
| candidate | US categorical | Count | 2 | 4 | 3 | 62 |
|  |  | % within candidate | 2.1% | 4.2% | 3.1% | 64.6% |
|  |  | % of Total | 0.9% | 1.9% | 1.4% | 28.8% |
|  | IM categorical | Count | 2 | 5 | 12 | 34 |
|  |  | % within candidate | 3.1% | 7.7% | 18.5% | 52.3% |
|  |  | % of Total | 0.9% | 2.3% | 5.6% | 15.8% |
|  | Preliminary | Count | 0 | 6 | 8 | 28 |
|  |  | % within candidate | 0.0% | 11.1% | 14.8% | 51.9% |
|  |  | % of Total | 0.0% | 2.8% | 3.7% | 13.0% |
| Total | | Count | 4 | 15 | 23 | 124 |
|  |  | % within candidate | 1.9% | 7.0% | 10.7% | 57.7% |
|  |  | % of Total | 1.9% | 7.0% | 10.7% | 57.7% |

| **Crosstab** | | | | |
| --- | --- | --- | --- | --- |
|  | | | Qteaser | Total |
|  |  |  | 5 |  |
| candidate | US categorical | Count | 25 | 96 |
|  |  | % within candidate | 26.0% | 100.0% |
|  |  | % of Total | 11.6% | 44.7% |
|  | IM categorical | Count | 12 | 65 |
|  |  | % within candidate | 18.5% | 100.0% |
|  |  | % of Total | 5.6% | 30.2% |
|  | Preliminary | Count | 12 | 54 |
|  |  | % within candidate | 22.2% | 100.0% |
|  |  | % of Total | 5.6% | 25.1% |
| Total | | Count | 49 | 215 |
|  |  | % within candidate | 22.8% | 100.0% |
|  |  | % of Total | 22.8% | 100.0% |

| **Chi-Square Tests** | | | |
| --- | --- | --- | --- |
|  | Value | df | Asymp. Sig. (2-sided) |
| Pearson Chi-Square | 16.093^a^ | 8 | .041 |
| Likelihood Ratio | 18.258 | 8 | .019 |
| Linear-by-Linear Association | 3.309 | 1 | .069 |
| N of Valid Cases | 215 |  |  |

| a. 5 cells (33.3%) have expected count less than 5. The minimum expected count is 1.00. |
| --- |

**candidate * Qmedical**

| **Crosstab** | | | | | | |
| --- | --- | --- | --- | --- | --- | --- |
|  | | | Qmedical | | | |
|  |  |  | 1 | 3 | 4 | 5 |
| candidate | US categorical | Count | 1 | 4 | 24 | 67 |
|  |  | % within candidate | 1.0% | 4.2% | 25.0% | 69.8% |
|  |  | % of Total | 0.5% | 1.9% | 11.2% | 31.2% |
|  | IM categorical | Count | 2 | 3 | 12 | 48 |
|  |  | % within candidate | 3.1% | 4.6% | 18.5% | 73.8% |
|  |  | % of Total | 0.9% | 1.4% | 5.6% | 22.3% |
|  | Preliminary | Count | 0 | 3 | 13 | 38 |
|  |  | % within candidate | 0.0% | 5.6% | 24.1% | 70.4% |
|  |  | % of Total | 0.0% | 1.4% | 6.0% | 17.7% |
| Total | | Count | 3 | 10 | 49 | 153 |
|  |  | % within candidate | 1.4% | 4.7% | 22.8% | 71.2% |
|  |  | % of Total | 1.4% | 4.7% | 22.8% | 71.2% |

| **Crosstab** | | | |
| --- | --- | --- | --- |
|  | | | Total |
|  |  |  |  |
| candidate | US categorical | Count | 96 |
|  |  | % within candidate | 100.0% |
|  |  | % of Total | 44.7% |
|  | IM categorical | Count | 65 |
|  |  | % within candidate | 100.0% |
|  |  | % of Total | 30.2% |
|  | Preliminary | Count | 54 |
|  |  | % within candidate | 100.0% |
|  |  | % of Total | 25.1% |
| Total | | Count | 215 |
|  |  | % within candidate | 100.0% |
|  |  | % of Total | 100.0% |

| **Chi-Square Tests** | | | |
| --- | --- | --- | --- |
|  | Value | df | Asymp. Sig. (2-sided) |
| Pearson Chi-Square | 3.175^a^ | 6 | .787 |
| Likelihood Ratio | 3.623 | 6 | .728 |
| Linear-by-Linear Association | .020 | 1 | .889 |
| N of Valid Cases | 215 |  |  |

| a. 6 cells (50.0%) have expected count less than 5. The minimum expected count is .75. |
| --- |

**candidate * ResIA_LT15**

| **Crosstab** | | | | | | |
| --- | --- | --- | --- | --- | --- | --- |
|  | | | ResIA_LT15 | | | |
|  |  |  | 1 | 2 | 3 | 4 |
| candidate | US categorical | Count | 1 | 0 | 7 | 16 |
|  |  | % within candidate | 1.0% | 0.0% | 7.3% | 16.7% |
|  |  | % of Total | 0.5% | 0.0% | 3.3% | 7.4% |
|  | IM categorical | Count | 2 | 6 | 5 | 8 |
|  |  | % within candidate | 3.1% | 9.2% | 7.7% | 12.3% |
|  |  | % of Total | 0.9% | 2.8% | 2.3% | 3.7% |
|  | Preliminary | Count | 1 | 3 | 6 | 11 |
|  |  | % within candidate | 1.9% | 5.6% | 11.1% | 20.4% |
|  |  | % of Total | 0.5% | 1.4% | 2.8% | 5.1% |
| Total | | Count | 4 | 9 | 18 | 35 |
|  |  | % within candidate | 1.9% | 4.2% | 8.4% | 16.3% |
|  |  | % of Total | 1.9% | 4.2% | 8.4% | 16.3% |

| **Crosstab** | | | | |
| --- | --- | --- | --- | --- |
|  | | | ResIA_LT15 | Total |
|  |  |  | 5 |  |
| candidate | US categorical | Count | 72 | 96 |
|  |  | % within candidate | 75.0% | 100.0% |
|  |  | % of Total | 33.5% | 44.7% |
|  | IM categorical | Count | 44 | 65 |
|  |  | % within candidate | 67.7% | 100.0% |
|  |  | % of Total | 20.5% | 30.2% |
|  | Preliminary | Count | 33 | 54 |
|  |  | % within candidate | 61.1% | 100.0% |
|  |  | % of Total | 15.3% | 25.1% |
| Total | | Count | 149 | 215 |
|  |  | % within candidate | 69.3% | 100.0% |
|  |  | % of Total | 69.3% | 100.0% |

| **Chi-Square Tests** | | | |
| --- | --- | --- | --- |
|  | Value | df | Asymp. Sig. (2-sided) |
| Pearson Chi-Square | 11.920^a^ | 8 | .155 |
| Likelihood Ratio | 14.864 | 8 | .062 |
| Linear-by-Linear Association | 4.641 | 1 | .031 |
| N of Valid Cases | 215 |  |  |

| a. 7 cells (46.7%) have expected count less than 5. The minimum expected count is 1.00. |
| --- |

**candidate * ResIA15_30**

| **Crosstab** | | | | | | |
| --- | --- | --- | --- | --- | --- | --- |
|  | | | ResIA15_30 | | | |
|  |  |  | 1 | 2 | 3 | 4 |
| candidate | US categorical | Count | 9 | 19 | 8 | 59 |
|  |  | % within candidate | 9.4% | 19.8% | 8.3% | 61.5% |
|  |  | % of Total | 4.2% | 8.8% | 3.7% | 27.4% |
|  | IM categorical | Count | 12 | 9 | 12 | 31 |
|  |  | % within candidate | 18.5% | 13.8% | 18.5% | 47.7% |
|  |  | % of Total | 5.6% | 4.2% | 5.6% | 14.4% |
|  | Preliminary | Count | 6 | 15 | 12 | 21 |
|  |  | % within candidate | 11.1% | 27.8% | 22.2% | 38.9% |
|  |  | % of Total | 2.8% | 7.0% | 5.6% | 9.8% |
| Total | | Count | 27 | 43 | 32 | 111 |
|  |  | % within candidate | 12.6% | 20.0% | 14.9% | 51.6% |
|  |  | % of Total | 12.6% | 20.0% | 14.9% | 51.6% |

| **Crosstab** | | | | |
| --- | --- | --- | --- | --- |
|  | | | ResIA15_30 | Total |
|  |  |  | 5 |  |
| candidate | US categorical | Count | 1 | 96 |
|  |  | % within candidate | 1.0% | 100.0% |
|  |  | % of Total | 0.5% | 44.7% |
|  | IM categorical | Count | 1 | 65 |
|  |  | % within candidate | 1.5% | 100.0% |
|  |  | % of Total | 0.5% | 30.2% |
|  | Preliminary | Count | 0 | 54 |
|  |  | % within candidate | 0.0% | 100.0% |
|  |  | % of Total | 0.0% | 25.1% |
| Total | | Count | 2 | 215 |
|  |  | % within candidate | 0.9% | 100.0% |
|  |  | % of Total | 0.9% | 100.0% |

| **Chi-Square Tests** | | | |
| --- | --- | --- | --- |
|  | Value | df | Asymp. Sig. (2-sided) |
| Pearson Chi-Square | 15.278^a^ | 8 | .054 |
| Likelihood Ratio | 15.847 | 8 | .045 |
| Linear-by-Linear Association | 3.943 | 1 | .047 |
| N of Valid Cases | 215 |  |  |

| a. 3 cells (20.0%) have expected count less than 5. The minimum expected count is .50. |
| --- |

**candidate * ResIA30_45**

| **Crosstab** | | | | | | |
| --- | --- | --- | --- | --- | --- | --- |
|  | | | ResIA30_45 | | | Total |
|  |  |  | 1 | 2 | 3 |  |
| candidate | US categorical | Count | 31 | 26 | 39 | 96 |
|  |  | % within candidate | 32.3% | 27.1% | 40.6% | 100.0% |
|  |  | % of Total | 14.4% | 12.1% | 18.1% | 44.7% |
|  | IM categorical | Count | 23 | 17 | 25 | 65 |
|  |  | % within candidate | 35.4% | 26.2% | 38.5% | 100.0% |
|  |  | % of Total | 10.7% | 7.9% | 11.6% | 30.2% |
|  | Preliminary | Count | 30 | 10 | 14 | 54 |
|  |  | % within candidate | 55.6% | 18.5% | 25.9% | 100.0% |
|  |  | % of Total | 14.0% | 4.7% | 6.5% | 25.1% |
| Total | | Count | 84 | 53 | 78 | 215 |
|  |  | % within candidate | 39.1% | 24.7% | 36.3% | 100.0% |
|  |  | % of Total | 39.1% | 24.7% | 36.3% | 100.0% |

| **Chi-Square Tests** | | | |
| --- | --- | --- | --- |
|  | Value | df | Asymp. Sig. (2-sided) |
| Pearson Chi-Square | 8.406^a^ | 4 | .078 |
| Likelihood Ratio | 8.273 | 4 | .082 |
| Linear-by-Linear Association | 5.909 | 1 | .015 |
| N of Valid Cases | 215 |  |  |

| a. 0 cells (0.0%) have expected count less than 5. The minimum expected count is 13.31. |
| --- |

**candidate * ResIA45_60**

| **Crosstab** | | | | | | |
| --- | --- | --- | --- | --- | --- | --- |
|  | | | ResIA45_60 | | | |
|  |  |  | 1 | 2 | 3 | 4 |
| candidate | US categorical | Count | 34 | 35 | 20 | 6 |
|  |  | % within candidate | 35.4% | 36.5% | 20.8% | 6.3% |
|  |  | % of Total | 15.8% | 16.3% | 9.3% | 2.8% |
|  | IM categorical | Count | 20 | 25 | 6 | 13 |
|  |  | % within candidate | 30.8% | 38.5% | 9.2% | 20.0% |
|  |  | % of Total | 9.3% | 11.6% | 2.8% | 6.0% |
|  | Preliminary | Count | 11 | 22 | 11 | 10 |
|  |  | % within candidate | 20.4% | 40.7% | 20.4% | 18.5% |
|  |  | % of Total | 5.1% | 10.2% | 5.1% | 4.7% |
| Total | | Count | 65 | 82 | 37 | 29 |
|  |  | % within candidate | 30.2% | 38.1% | 17.2% | 13.5% |
|  |  | % of Total | 30.2% | 38.1% | 17.2% | 13.5% |

| **Crosstab** | | | | |
| --- | --- | --- | --- | --- |
|  | | | ResIA45_60 | Total |
|  |  |  | 5 |  |
| candidate | US categorical | Count | 1 | 96 |
|  |  | % within candidate | 1.0% | 100.0% |
|  |  | % of Total | 0.5% | 44.7% |
|  | IM categorical | Count | 1 | 65 |
|  |  | % within candidate | 1.5% | 100.0% |
|  |  | % of Total | 0.5% | 30.2% |
|  | Preliminary | Count | 0 | 54 |
|  |  | % within candidate | 0.0% | 100.0% |
|  |  | % of Total | 0.0% | 25.1% |
| Total | | Count | 2 | 215 |
|  |  | % within candidate | 0.9% | 100.0% |
|  |  | % of Total | 0.9% | 100.0% |

| **Chi-Square Tests** | | | |
| --- | --- | --- | --- |
|  | Value | df | Asymp. Sig. (2-sided) |
| Pearson Chi-Square | 13.775^a^ | 8 | .088 |
| Likelihood Ratio | 15.408 | 8 | .052 |
| Linear-by-Linear Association | 4.447 | 1 | .035 |
| N of Valid Cases | 215 |  |  |

| a. 3 cells (20.0%) have expected count less than 5. The minimum expected count is .50. |
| --- |

**candidate * ResIA_GT60**

| **Crosstab** | | | | | | |
| --- | --- | --- | --- | --- | --- | --- |
|  | | | ResIA_GT60 | | | |
|  |  |  | 1 | 2 | 3 | 4 |
| candidate | US categorical | Count | 21 | 16 | 22 | 15 |
|  |  | % within candidate | 21.9% | 16.7% | 22.9% | 15.6% |
|  |  | % of Total | 9.8% | 7.4% | 10.2% | 7.0% |
|  | IM categorical | Count | 8 | 7 | 17 | 13 |
|  |  | % within candidate | 12.3% | 10.8% | 26.2% | 20.0% |
|  |  | % of Total | 3.7% | 3.3% | 7.9% | 6.0% |
|  | Preliminary | Count | 6 | 4 | 11 | 12 |
|  |  | % within candidate | 11.1% | 7.4% | 20.4% | 22.2% |
|  |  | % of Total | 2.8% | 1.9% | 5.1% | 5.6% |
| Total | | Count | 35 | 27 | 50 | 40 |
|  |  | % within candidate | 16.3% | 12.6% | 23.3% | 18.6% |
|  |  | % of Total | 16.3% | 12.6% | 23.3% | 18.6% |

| **Crosstab** | | | | |
| --- | --- | --- | --- | --- |
|  | | | ResIA_GT60 | Total |
|  |  |  | 5 |  |
| candidate | US categorical | Count | 22 | 96 |
|  |  | % within candidate | 22.9% | 100.0% |
|  |  | % of Total | 10.2% | 44.7% |
|  | IM categorical | Count | 20 | 65 |
|  |  | % within candidate | 30.8% | 100.0% |
|  |  | % of Total | 9.3% | 30.2% |
|  | Preliminary | Count | 21 | 54 |
|  |  | % within candidate | 38.9% | 100.0% |
|  |  | % of Total | 9.8% | 25.1% |
| Total | | Count | 63 | 215 |
|  |  | % within candidate | 29.3% | 100.0% |
|  |  | % of Total | 29.3% | 100.0% |

| **Chi-Square Tests** | | | |
| --- | --- | --- | --- |
|  | Value | df | Asymp. Sig. (2-sided) |
| Pearson Chi-Square | 10.375^a^ | 8 | .240 |
| Likelihood Ratio | 10.402 | 8 | .238 |
| Linear-by-Linear Association | 8.803 | 1 | .003 |
| N of Valid Cases | 215 |  |  |

| a. 0 cells (0.0%) have expected count less than 5. The minimum expected count is 6.78. |
| --- |

**candidate * october**

| **Crosstab** | | | | | | |
| --- | --- | --- | --- | --- | --- | --- |
|  | | | october | | | |
|  |  |  | 1 | 2 | 3 | 4 |
| candidate | US categorical | Count | 9 | 22 | 25 | 31 |
|  |  | % within candidate | 9.4% | 22.9% | 26.0% | 32.3% |
|  |  | % of Total | 4.2% | 10.2% | 11.6% | 14.4% |
|  | IM categorical | Count | 10 | 10 | 14 | 21 |
|  |  | % within candidate | 15.4% | 15.4% | 21.5% | 32.3% |
|  |  | % of Total | 4.7% | 4.7% | 6.5% | 9.8% |
|  | Preliminary | Count | 14 | 10 | 13 | 14 |
|  |  | % within candidate | 25.9% | 18.5% | 24.1% | 25.9% |
|  |  | % of Total | 6.5% | 4.7% | 6.0% | 6.5% |
| Total | | Count | 33 | 42 | 52 | 66 |
|  |  | % within candidate | 15.3% | 19.5% | 24.2% | 30.7% |
|  |  | % of Total | 15.3% | 19.5% | 24.2% | 30.7% |

| **Crosstab** | | | | |
| --- | --- | --- | --- | --- |
|  | | | october | Total |
|  |  |  | 5 |  |
| candidate | US categorical | Count | 9 | 96 |
|  |  | % within candidate | 9.4% | 100.0% |
|  |  | % of Total | 4.2% | 44.7% |
|  | IM categorical | Count | 10 | 65 |
|  |  | % within candidate | 15.4% | 100.0% |
|  |  | % of Total | 4.7% | 30.2% |
|  | Preliminary | Count | 3 | 54 |
|  |  | % within candidate | 5.6% | 100.0% |
|  |  | % of Total | 1.4% | 25.1% |
| Total | | Count | 22 | 215 |
|  |  | % within candidate | 10.2% | 100.0% |
|  |  | % of Total | 10.2% | 100.0% |

| **Chi-Square Tests** | | | |
| --- | --- | --- | --- |
|  | Value | df | Asymp. Sig. (2-sided) |
| Pearson Chi-Square | 11.101^a^ | 8 | .196 |
| Likelihood Ratio | 10.830 | 8 | .211 |
| Linear-by-Linear Association | 3.258 | 1 | .071 |
| N of Valid Cases | 215 |  |  |

| a. 0 cells (0.0%) have expected count less than 5. The minimum expected count is 5.53. |
| --- |

**candidate * november**

| **Crosstab** | | | | | | |
| --- | --- | --- | --- | --- | --- | --- |
|  | | | november | | | |
|  |  |  | 1 | 2 | 3 | 4 |
| candidate | US categorical | Count | 68 | 24 | 2 | 1 |
|  |  | % within candidate | 70.8% | 25.0% | 2.1% | 1.0% |
|  |  | % of Total | 31.6% | 11.2% | 0.9% | 0.5% |
|  | IM categorical | Count | 40 | 15 | 7 | 3 |
|  |  | % within candidate | 61.5% | 23.1% | 10.8% | 4.6% |
|  |  | % of Total | 18.6% | 7.0% | 3.3% | 1.4% |
|  | Preliminary | Count | 20 | 21 | 7 | 6 |
|  |  | % within candidate | 37.0% | 38.9% | 13.0% | 11.1% |
|  |  | % of Total | 9.3% | 9.8% | 3.3% | 2.8% |
| Total | | Count | 128 | 60 | 16 | 10 |
|  |  | % within candidate | 59.5% | 27.9% | 7.4% | 4.7% |
|  |  | % of Total | 59.5% | 27.9% | 7.4% | 4.7% |

| **Crosstab** | | | | |
| --- | --- | --- | --- | --- |
|  | | | november | Total |
|  |  |  | 5 |  |
| candidate | US categorical | Count | 1 | 96 |
|  |  | % within candidate | 1.0% | 100.0% |
|  |  | % of Total | 0.5% | 44.7% |
|  | IM categorical | Count | 0 | 65 |
|  |  | % within candidate | 0.0% | 100.0% |
|  |  | % of Total | 0.0% | 30.2% |
|  | Preliminary | Count | 0 | 54 |
|  |  | % within candidate | 0.0% | 100.0% |
|  |  | % of Total | 0.0% | 25.1% |
| Total | | Count | 1 | 215 |
|  |  | % within candidate | 0.5% | 100.0% |
|  |  | % of Total | 0.5% | 100.0% |

| **Chi-Square Tests** | | | |
| --- | --- | --- | --- |
|  | Value | df | Asymp. Sig. (2-sided) |
| Pearson Chi-Square | 25.518^a^ | 8 | .001 |
| Likelihood Ratio | 27.125 | 8 | .001 |
| Linear-by-Linear Association | 17.551 | 1 | .000 |
| N of Valid Cases | 215 |  |  |

| a. 8 cells (53.3%) have expected count less than 5. The minimum expected count is .25. |
| --- |

**candidate * december**

| **Crosstab** | | | | | | |
| --- | --- | --- | --- | --- | --- | --- |
|  | | | december | | | |
|  |  |  | 1 | 2 | 3 | 4 |
| candidate | US categorical | Count | 17 | 46 | 32 | 1 |
|  |  | % within candidate | 17.7% | 47.9% | 33.3% | 1.0% |
|  |  | % of Total | 7.9% | 21.4% | 14.9% | 0.5% |
|  | IM categorical | Count | 12 | 32 | 19 | 0 |
|  |  | % within candidate | 18.5% | 49.2% | 29.2% | 0.0% |
|  |  | % of Total | 5.6% | 14.9% | 8.8% | 0.0% |
|  | Preliminary | Count | 15 | 14 | 20 | 2 |
|  |  | % within candidate | 27.8% | 25.9% | 37.0% | 3.7% |
|  |  | % of Total | 7.0% | 6.5% | 9.3% | 0.9% |
| Total | | Count | 44 | 92 | 71 | 3 |
|  |  | % within candidate | 20.5% | 42.8% | 33.0% | 1.4% |
|  |  | % of Total | 20.5% | 42.8% | 33.0% | 1.4% |

| **Crosstab** | | | | |
| --- | --- | --- | --- | --- |
|  | | | december | Total |
|  |  |  | 5 |  |
| candidate | US categorical | Count | 0 | 96 |
|  |  | % within candidate | 0.0% | 100.0% |
|  |  | % of Total | 0.0% | 44.7% |
|  | IM categorical | Count | 2 | 65 |
|  |  | % within candidate | 3.1% | 100.0% |
|  |  | % of Total | 0.9% | 30.2% |
|  | Preliminary | Count | 3 | 54 |
|  |  | % within candidate | 5.6% | 100.0% |
|  |  | % of Total | 1.4% | 25.1% |
| Total | | Count | 5 | 215 |
|  |  | % within candidate | 2.3% | 100.0% |
|  |  | % of Total | 2.3% | 100.0% |

| **Chi-Square Tests** | | | |
| --- | --- | --- | --- |
|  | Value | df | Asymp. Sig. (2-sided) |
| Pearson Chi-Square | 15.121^a^ | 8 | .057 |
| Likelihood Ratio | 17.341 | 8 | .027 |
| Linear-by-Linear Association | 1.008 | 1 | .315 |
| N of Valid Cases | 215 |  |  |

| a. 6 cells (40.0%) have expected count less than 5. The minimum expected count is .75. |
| --- |

**candidate * january**

| **Crosstab** | | | | | | |
| --- | --- | --- | --- | --- | --- | --- |
|  | | | january | | | |
|  |  |  | 1 | 2 | 3 | 4 |
| candidate | US categorical | Count | 0 | 4 | 37 | 55 |
|  |  | % within candidate | 0.0% | 4.2% | 38.5% | 57.3% |
|  |  | % of Total | 0.0% | 1.9% | 17.2% | 25.6% |
|  | IM categorical | Count | 2 | 8 | 23 | 31 |
|  |  | % within candidate | 3.1% | 12.3% | 35.4% | 47.7% |
|  |  | % of Total | 0.9% | 3.7% | 10.7% | 14.4% |
|  | Preliminary | Count | 5 | 6 | 12 | 30 |
|  |  | % within candidate | 9.3% | 11.1% | 22.2% | 55.6% |
|  |  | % of Total | 2.3% | 2.8% | 5.6% | 14.0% |
| Total | | Count | 7 | 18 | 72 | 116 |
|  |  | % within candidate | 3.3% | 8.4% | 33.5% | 54.0% |
|  |  | % of Total | 3.3% | 8.4% | 33.5% | 54.0% |

| **Crosstab** | | | | |
| --- | --- | --- | --- | --- |
|  | | | january | Total |
|  |  |  | 5 |  |
| candidate | US categorical | Count | 0 | 96 |
|  |  | % within candidate | 0.0% | 100.0% |
|  |  | % of Total | 0.0% | 44.7% |
|  | IM categorical | Count | 1 | 65 |
|  |  | % within candidate | 1.5% | 100.0% |
|  |  | % of Total | 0.5% | 30.2% |
|  | Preliminary | Count | 1 | 54 |
|  |  | % within candidate | 1.9% | 100.0% |
|  |  | % of Total | 0.5% | 25.1% |
| Total | | Count | 2 | 215 |
|  |  | % within candidate | 0.9% | 100.0% |
|  |  | % of Total | 0.9% | 100.0% |

| **Chi-Square Tests** | | | |
| --- | --- | --- | --- |
|  | Value | df | Asymp. Sig. (2-sided) |
| Pearson Chi-Square | 18.013^a^ | 8 | .021 |
| Likelihood Ratio | 20.391 | 8 | .009 |
| Linear-by-Linear Association | 3.573 | 1 | .059 |
| N of Valid Cases | 215 |  |  |

| a. 7 cells (46.7%) have expected count less than 5. The minimum expected count is .50. |
| --- |

**candidate * february**

| **Crosstab** | | | | | | |
| --- | --- | --- | --- | --- | --- | --- |
|  | | | february | | | |
|  |  |  | 1 | 2 | 3 | 4 |
| candidate | US categorical | Count | 2 | 0 | 0 | 8 |
|  |  | % within candidate | 2.1% | 0.0% | 0.0% | 8.3% |
|  |  | % of Total | 0.9% | 0.0% | 0.0% | 3.7% |
|  | IM categorical | Count | 1 | 0 | 2 | 10 |
|  |  | % within candidate | 1.5% | 0.0% | 3.1% | 15.4% |
|  |  | % of Total | 0.5% | 0.0% | 0.9% | 4.7% |
|  | Preliminary | Count | 0 | 3 | 2 | 2 |
|  |  | % within candidate | 0.0% | 5.6% | 3.7% | 3.7% |
|  |  | % of Total | 0.0% | 1.4% | 0.9% | 0.9% |
| Total | | Count | 3 | 3 | 4 | 20 |
|  |  | % within candidate | 1.4% | 1.4% | 1.9% | 9.3% |
|  |  | % of Total | 1.4% | 1.4% | 1.9% | 9.3% |

| **Crosstab** | | | | |
| --- | --- | --- | --- | --- |
|  | | | february | Total |
|  |  |  | 5 |  |
| candidate | US categorical | Count | 86 | 96 |
|  |  | % within candidate | 89.6% | 100.0% |
|  |  | % of Total | 40.0% | 44.7% |
|  | IM categorical | Count | 52 | 65 |
|  |  | % within candidate | 80.0% | 100.0% |
|  |  | % of Total | 24.2% | 30.2% |
|  | Preliminary | Count | 47 | 54 |
|  |  | % within candidate | 87.0% | 100.0% |
|  |  | % of Total | 21.9% | 25.1% |
| Total | | Count | 185 | 215 |
|  |  | % within candidate | 86.0% | 100.0% |
|  |  | % of Total | 86.0% | 100.0% |

| **Chi-Square Tests** | | | |
| --- | --- | --- | --- |
|  | Value | df | Asymp. Sig. (2-sided) |
| Pearson Chi-Square | 18.246^a^ | 8 | .019 |
| Likelihood Ratio | 19.900 | 8 | .011 |
| Linear-by-Linear Association | 1.111 | 1 | .292 |
| N of Valid Cases | 215 |  |  |

| a. 9 cells (60.0%) have expected count less than 5. The minimum expected count is .75. |
| --- |

**candidate * overview**

| **Crosstab** | | | | | | |
| --- | --- | --- | --- | --- | --- | --- |
|  | | | overview | | | |
|  |  |  | 1 | 2 | 3 | 4 |
| candidate | US categorical | Count | 40 | 30 | 18 | 5 |
|  |  | % within candidate | 41.7% | 31.3% | 18.8% | 5.2% |
|  |  | % of Total | 18.6% | 14.0% | 8.4% | 2.3% |
|  | IM categorical | Count | 27 | 29 | 1 | 4 |
|  |  | % within candidate | 41.5% | 44.6% | 1.5% | 6.2% |
|  |  | % of Total | 12.6% | 13.5% | 0.5% | 1.9% |
|  | Preliminary | Count | 22 | 20 | 10 | 1 |
|  |  | % within candidate | 40.7% | 37.0% | 18.5% | 1.9% |
|  |  | % of Total | 10.2% | 9.3% | 4.7% | 0.5% |
| Total | | Count | 89 | 79 | 29 | 10 |
|  |  | % within candidate | 41.4% | 36.7% | 13.5% | 4.7% |
|  |  | % of Total | 41.4% | 36.7% | 13.5% | 4.7% |

| **Crosstab** | | | | |
| --- | --- | --- | --- | --- |
|  | | | overview | Total |
|  |  |  | 5 |  |
| candidate | US categorical | Count | 3 | 96 |
|  |  | % within candidate | 3.1% | 100.0% |
|  |  | % of Total | 1.4% | 44.7% |
|  | IM categorical | Count | 4 | 65 |
|  |  | % within candidate | 6.2% | 100.0% |
|  |  | % of Total | 1.9% | 30.2% |
|  | Preliminary | Count | 1 | 54 |
|  |  | % within candidate | 1.9% | 100.0% |
|  |  | % of Total | 0.5% | 25.1% |
| Total | | Count | 8 | 215 |
|  |  | % within candidate | 3.7% | 100.0% |
|  |  | % of Total | 3.7% | 100.0% |

| **Chi-Square Tests** | | | |
| --- | --- | --- | --- |
|  | Value | df | Asymp. Sig. (2-sided) |
| Pearson Chi-Square | 14.680^a^ | 8 | .066 |
| Likelihood Ratio | 18.830 | 8 | .016 |
| Linear-by-Linear Association | .335 | 1 | .563 |
| N of Valid Cases | 215 |  |  |

| a. 6 cells (40.0%) have expected count less than 5. The minimum expected count is 2.01. |
| --- |

**candidate * interview**

| **Crosstab** | | | | | | |
| --- | --- | --- | --- | --- | --- | --- |
|  | | | interview | | | |
|  |  |  | 1 | 2 | 3 | 4 |
| candidate | US categorical | Count | 37 | 36 | 11 | 8 |
|  |  | % within candidate | 38.5% | 37.5% | 11.5% | 8.3% |
|  |  | % of Total | 17.2% | 16.7% | 5.1% | 3.7% |
|  | IM categorical | Count | 32 | 23 | 8 | 2 |
|  |  | % within candidate | 49.2% | 35.4% | 12.3% | 3.1% |
|  |  | % of Total | 14.9% | 10.7% | 3.7% | 0.9% |
|  | Preliminary | Count | 23 | 21 | 8 | 2 |
|  |  | % within candidate | 42.6% | 38.9% | 14.8% | 3.7% |
|  |  | % of Total | 10.7% | 9.8% | 3.7% | 0.9% |
| Total | | Count | 92 | 80 | 27 | 12 |
|  |  | % within candidate | 42.8% | 37.2% | 12.6% | 5.6% |
|  |  | % of Total | 42.8% | 37.2% | 12.6% | 5.6% |

| **Crosstab** | | | | |
| --- | --- | --- | --- | --- |
|  | | | interview | Total |
|  |  |  | 5 |  |
| candidate | US categorical | Count | 4 | 96 |
|  |  | % within candidate | 4.2% | 100.0% |
|  |  | % of Total | 1.9% | 44.7% |
|  | IM categorical | Count | 0 | 65 |
|  |  | % within candidate | 0.0% | 100.0% |
|  |  | % of Total | 0.0% | 30.2% |
|  | Preliminary | Count | 0 | 54 |
|  |  | % within candidate | 0.0% | 100.0% |
|  |  | % of Total | 0.0% | 25.1% |
| Total | | Count | 4 | 215 |
|  |  | % within candidate | 1.9% | 100.0% |
|  |  | % of Total | 1.9% | 100.0% |

| **Chi-Square Tests** | | | |
| --- | --- | --- | --- |
|  | Value | df | Asymp. Sig. (2-sided) |
| Pearson Chi-Square | 8.784^a^ | 8 | .361 |
| Likelihood Ratio | 10.268 | 8 | .247 |
| Linear-by-Linear Association | 2.641 | 1 | .104 |
| N of Valid Cases | 215 |  |  |

| a. 5 cells (33.3%) have expected count less than 5. The minimum expected count is 1.00. |
| --- |

**candidate * tour**

| **Crosstab** | | | | | | |
| --- | --- | --- | --- | --- | --- | --- |
|  | | | tour | | | |
|  |  |  | 1 | 2 | 3 | 4 |
| candidate | US categorical | Count | 1 | 3 | 14 | 32 |
|  |  | % within candidate | 1.0% | 3.1% | 14.6% | 33.3% |
|  |  | % of Total | 0.5% | 1.4% | 6.5% | 14.9% |
|  | IM categorical | Count | 1 | 1 | 21 | 22 |
|  |  | % within candidate | 1.5% | 1.5% | 32.3% | 33.8% |
|  |  | % of Total | 0.5% | 0.5% | 9.8% | 10.2% |
|  | Preliminary | Count | 1 | 0 | 7 | 26 |
|  |  | % within candidate | 1.9% | 0.0% | 13.0% | 48.1% |
|  |  | % of Total | 0.5% | 0.0% | 3.3% | 12.1% |
| Total | | Count | 3 | 4 | 42 | 80 |
|  |  | % within candidate | 1.4% | 1.9% | 19.5% | 37.2% |
|  |  | % of Total | 1.4% | 1.9% | 19.5% | 37.2% |

| **Crosstab** | | | | |
| --- | --- | --- | --- | --- |
|  | | | tour | Total |
|  |  |  | 5 |  |
| candidate | US categorical | Count | 46 | 96 |
|  |  | % within candidate | 47.9% | 100.0% |
|  |  | % of Total | 21.4% | 44.7% |
|  | IM categorical | Count | 20 | 65 |
|  |  | % within candidate | 30.8% | 100.0% |
|  |  | % of Total | 9.3% | 30.2% |
|  | Preliminary | Count | 20 | 54 |
|  |  | % within candidate | 37.0% | 100.0% |
|  |  | % of Total | 9.3% | 25.1% |
| Total | | Count | 86 | 215 |
|  |  | % within candidate | 40.0% | 100.0% |
|  |  | % of Total | 40.0% | 100.0% |

| **Chi-Square Tests** | | | |
| --- | --- | --- | --- |
|  | Value | df | Asymp. Sig. (2-sided) |
| Pearson Chi-Square | 15.198^a^ | 8 | .055 |
| Likelihood Ratio | 15.346 | 8 | .053 |
| Linear-by-Linear Association | .537 | 1 | .464 |
| N of Valid Cases | 215 |  |  |

| a. 6 cells (40.0%) have expected count less than 5. The minimum expected count is .75. |
| --- |

**candidate * morning_report**

| **Crosstab** | | | | | | |
| --- | --- | --- | --- | --- | --- | --- |
|  | | | morning_report | | | |
|  |  |  | 1 | 2 | 3 | 4 |
| candidate | US categorical | Count | 3 | 9 | 17 | 30 |
|  |  | % within candidate | 3.1% | 9.4% | 17.7% | 31.3% |
|  |  | % of Total | 1.4% | 4.2% | 7.9% | 14.0% |
|  | IM categorical | Count | 3 | 4 | 12 | 19 |
|  |  | % within candidate | 4.6% | 6.2% | 18.5% | 29.2% |
|  |  | % of Total | 1.4% | 1.9% | 5.6% | 8.8% |
|  | Preliminary | Count | 0 | 1 | 4 | 17 |
|  |  | % within candidate | 0.0% | 1.9% | 7.4% | 31.5% |
|  |  | % of Total | 0.0% | 0.5% | 1.9% | 7.9% |
| Total | | Count | 6 | 14 | 33 | 66 |
|  |  | % within candidate | 2.8% | 6.5% | 15.3% | 30.7% |
|  |  | % of Total | 2.8% | 6.5% | 15.3% | 30.7% |

| **Crosstab** | | | | |
| --- | --- | --- | --- | --- |
|  | | | morning_report | Total |
|  |  |  | 5 |  |
| candidate | US categorical | Count | 37 | 96 |
|  |  | % within candidate | 38.5% | 100.0% |
|  |  | % of Total | 17.2% | 44.7% |
|  | IM categorical | Count | 27 | 65 |
|  |  | % within candidate | 41.5% | 100.0% |
|  |  | % of Total | 12.6% | 30.2% |
|  | Preliminary | Count | 32 | 54 |
|  |  | % within candidate | 59.3% | 100.0% |
|  |  | % of Total | 14.9% | 25.1% |
| Total | | Count | 96 | 215 |
|  |  | % within candidate | 44.7% | 100.0% |
|  |  | % of Total | 44.7% | 100.0% |

| **Chi-Square Tests** | | | |
| --- | --- | --- | --- |
|  | Value | df | Asymp. Sig. (2-sided) |
| Pearson Chi-Square | 11.911^a^ | 8 | .155 |
| Likelihood Ratio | 14.146 | 8 | .078 |
| Linear-by-Linear Association | 8.378 | 1 | .004 |
| N of Valid Cases | 215 |  |  |

| a. 5 cells (33.3%) have expected count less than 5. The minimum expected count is 1.51. |
| --- |

**candidate * lunch**

| **Crosstab** | | | | | | |
| --- | --- | --- | --- | --- | --- | --- |
|  | | | lunch | | | |
|  |  |  | 1 | 2 | 3 | 4 |
| candidate | US categorical | Count | 15 | 18 | 36 | 21 |
|  |  | % within candidate | 15.6% | 18.8% | 37.5% | 21.9% |
|  |  | % of Total | 7.0% | 8.4% | 16.7% | 9.8% |
|  | IM categorical | Count | 2 | 8 | 21 | 18 |
|  |  | % within candidate | 3.1% | 12.3% | 32.3% | 27.7% |
|  |  | % of Total | 0.9% | 3.7% | 9.8% | 8.4% |
|  | Preliminary | Count | 8 | 12 | 25 | 8 |
|  |  | % within candidate | 14.8% | 22.2% | 46.3% | 14.8% |
|  |  | % of Total | 3.7% | 5.6% | 11.6% | 3.7% |
| Total | | Count | 25 | 38 | 82 | 47 |
|  |  | % within candidate | 11.6% | 17.7% | 38.1% | 21.9% |
|  |  | % of Total | 11.6% | 17.7% | 38.1% | 21.9% |

| **Crosstab** | | | | | |
| --- | --- | --- | --- | --- | --- |
|  | | | lunch | | |
|  |  |  | 5 | 33 |  |
| candidate | US categorical | Count | 6 | 0 | 96 |
|  |  | % within candidate | 6.3% | 0.0% | 100.0% |
|  |  | % of Total | 2.8% | 0.0% | 44.7% |
|  | IM categorical | Count | 15 | 1 | 65 |
|  |  | % within candidate | 23.1% | 1.5% | 100.0% |
|  |  | % of Total | 7.0% | 0.5% | 30.2% |
|  | Preliminary | Count | 1 | 0 | 54 |
|  |  | % within candidate | 1.9% | 0.0% | 100.0% |
|  |  | % of Total | 0.5% | 0.0% | 25.1% |
| Total | | Count | 22 | 1 | 215 |
|  |  | % within candidate | 10.2% | 0.5% | 100.0% |
|  |  | % of Total | 10.2% | 0.5% | 100.0% |

| **Chi-Square Tests** | | | |
| --- | --- | --- | --- |
|  | Value | df | Asymp. Sig. (2-sided) |
| Pearson Chi-Square | 29.384^a^ | 10 | .001 |
| Likelihood Ratio | 30.528 | 10 | .001 |
| Linear-by-Linear Association | .015 | 1 | .901 |
| N of Valid Cases | 215 |  |  |

| a. 3 cells (16.7%) have expected count less than 5. The minimum expected count is .25. |
| --- |

**candidate * dinner**

| **Crosstab** | | | | | |
| --- | --- | --- | --- | --- | --- |
|  | | | dinner | | Total |
|  |  |  | yes | no |  |
| candidate | US categorical | Count | 63 | 33 | 96 |
|  |  | % within candidate | 65.6% | 34.4% | 100.0% |
|  |  | % of Total | 29.3% | 15.3% | 44.7% |
|  | IM categorical | Count | 41 | 24 | 65 |
|  |  | % within candidate | 63.1% | 36.9% | 100.0% |
|  |  | % of Total | 19.1% | 11.2% | 30.2% |
|  | Preliminary | Count | 17 | 37 | 54 |
|  |  | % within candidate | 31.5% | 68.5% | 100.0% |
|  |  | % of Total | 7.9% | 17.2% | 25.1% |
| Total | | Count | 121 | 94 | 215 |
|  |  | % within candidate | 56.3% | 43.7% | 100.0% |
|  |  | % of Total | 56.3% | 43.7% | 100.0% |

| **Chi-Square Tests** | | | |
| --- | --- | --- | --- |
|  | Value | df | Asymp. Sig. (2-sided) |
| Pearson Chi-Square | 18.124^a^ | 2 | .000 |
| Likelihood Ratio | 18.219 | 2 | .000 |
| Linear-by-Linear Association | 14.267 | 1 | .000 |
| N of Valid Cases | 215 |  |  |

| a. 0 cells (0.0%) have expected count less than 5. The minimum expected count is 23.61. |
| --- |

**candidate * tour_optional**

| **Crosstab** | | | | | |
| --- | --- | --- | --- | --- | --- |
|  | | | tour_optional | | Total |
|  |  |  | yes | no |  |
| candidate | US categorical | Count | 29 | 67 | 96 |
|  |  | % within candidate | 30.2% | 69.8% | 100.0% |
|  |  | % of Total | 13.5% | 31.2% | 44.7% |
|  | IM categorical | Count | 14 | 51 | 65 |
|  |  | % within candidate | 21.5% | 78.5% | 100.0% |
|  |  | % of Total | 6.5% | 23.7% | 30.2% |
|  | Preliminary | Count | 31 | 23 | 54 |
|  |  | % within candidate | 57.4% | 42.6% | 100.0% |
|  |  | % of Total | 14.4% | 10.7% | 25.1% |
| Total | | Count | 74 | 141 | 215 |
|  |  | % within candidate | 34.4% | 65.6% | 100.0% |
|  |  | % of Total | 34.4% | 65.6% | 100.0% |

| **Chi-Square Tests** | | | |
| --- | --- | --- | --- |
|  | Value | df | Asymp. Sig. (2-sided) |
| Pearson Chi-Square | 18.174^a^ | 2 | .000 |
| Likelihood Ratio | 17.799 | 2 | .000 |
| Linear-by-Linear Association | 8.421 | 1 | .004 |
| N of Valid Cases | 215 |  |  |

| a. 0 cells (0.0%) have expected count less than 5. The minimum expected count is 18.59. |
| --- |

**candidate * exit_interview**

| **Crosstab** | | | | | |
| --- | --- | --- | --- | --- | --- |
|  | | | exit_interview | | Total |
|  |  |  | neccesary | unneccesary |  |
| candidate | US categorical | Count | 39 | 57 | 96 |
|  |  | % within candidate | 40.6% | 59.4% | 100.0% |
|  |  | % of Total | 18.1% | 26.5% | 44.7% |
|  | IM categorical | Count | 46 | 19 | 65 |
|  |  | % within candidate | 70.8% | 29.2% | 100.0% |
|  |  | % of Total | 21.4% | 8.8% | 30.2% |
|  | Preliminary | Count | 11 | 43 | 54 |
|  |  | % within candidate | 20.4% | 79.6% | 100.0% |
|  |  | % of Total | 5.1% | 20.0% | 25.1% |
| Total | | Count | 96 | 119 | 215 |
|  |  | % within candidate | 44.7% | 55.3% | 100.0% |
|  |  | % of Total | 44.7% | 55.3% | 100.0% |

| **Chi-Square Tests** | | | |
| --- | --- | --- | --- |
|  | Value | df | Asymp. Sig. (2-sided) |
| Pearson Chi-Square | 31.453^a^ | 2 | .000 |
| Likelihood Ratio | 32.759 | 2 | .000 |
| Linear-by-Linear Association | 2.428 | 1 | .119 |
| N of Valid Cases | 215 |  |  |

| a. 0 cells (0.0%) have expected count less than 5. The minimum expected count is 24.11. |
| --- |

**CATEGROICAL VERSUS PRELIMS**

**Cat_prelim * INT_phone**

| **Crosstab** | | | | | | |
| --- | --- | --- | --- | --- | --- | --- |
|  | | | INT_phone | | | |
|  |  |  | 1 | 2 | 3 | 4 |
| Cat_prelim | categorical | Count | 2 | 7 | 33 | 48 |
|  |  | % within Cat_prelim | 1.2% | 4.3% | 20.5% | 29.8% |
|  |  | % of Total | 0.9% | 3.3% | 15.3% | 22.3% |
|  | preliminary | Count | 2 | 11 | 18 | 10 |
|  |  | % within Cat_prelim | 3.7% | 20.4% | 33.3% | 18.5% |
|  |  | % of Total | 0.9% | 5.1% | 8.4% | 4.7% |
| Total | | Count | 4 | 18 | 51 | 58 |
|  |  | % within Cat_prelim | 1.9% | 8.4% | 23.7% | 27.0% |
|  |  | % of Total | 1.9% | 8.4% | 23.7% | 27.0% |

| **Crosstab** | | | | |
| --- | --- | --- | --- | --- |
|  | | | INT_phone | Total |
|  |  |  | 5 |  |
| Cat_prelim | categorical | Count | 71 | 161 |
|  |  | % within Cat_prelim | 44.1% | 100.0% |
|  |  | % of Total | 33.0% | 74.9% |
|  | preliminary | Count | 13 | 54 |
|  |  | % within Cat_prelim | 24.1% | 100.0% |
|  |  | % of Total | 6.0% | 25.1% |
| Total | | Count | 84 | 215 |
|  |  | % within Cat_prelim | 39.1% | 100.0% |
|  |  | % of Total | 39.1% | 100.0% |

| **Chi-Square Tests** | | | |
| --- | --- | --- | --- |
|  | Value | df | Asymp. Sig. (2-sided) |
| Pearson Chi-Square | 22.588^a^ | 4 | .000 |
| Likelihood Ratio | 20.814 | 4 | .000 |
| Linear-by-Linear Association | 18.691 | 1 | .000 |
| N of Valid Cases | 215 |  |  |

| a. 3 cells (30.0%) have expected count less than 5. The minimum expected count is 1.00. |
| --- |

**Cat_prelim * INT_lunch**

| **Crosstab** | | | | | | |
| --- | --- | --- | --- | --- | --- | --- |
|  | | | INT_lunch | | | |
|  |  |  | 1 | 2 | 3 | 4 |
| Cat_prelim | categorical | Count | 1 | 85 | 51 | 20 |
|  |  | % within Cat_prelim | 0.6% | 52.8% | 31.7% | 12.4% |
|  |  | % of Total | 0.5% | 39.5% | 23.7% | 9.3% |
|  | preliminary | Count | 0 | 26 | 19 | 9 |
|  |  | % within Cat_prelim | 0.0% | 48.1% | 35.2% | 16.7% |
|  |  | % of Total | 0.0% | 12.1% | 8.8% | 4.2% |
| Total | | Count | 1 | 111 | 70 | 29 |
|  |  | % within Cat_prelim | 0.5% | 51.6% | 32.6% | 13.5% |
|  |  | % of Total | 0.5% | 51.6% | 32.6% | 13.5% |

| **Crosstab** | | | | |
| --- | --- | --- | --- | --- |
|  | | | INT_lunch | Total |
|  |  |  | 5 |  |
| Cat_prelim | categorical | Count | 4 | 161 |
|  |  | % within Cat_prelim | 2.5% | 100.0% |
|  |  | % of Total | 1.9% | 74.9% |
|  | preliminary | Count | 0 | 54 |
|  |  | % within Cat_prelim | 0.0% | 100.0% |
|  |  | % of Total | 0.0% | 25.1% |
| Total | | Count | 4 | 215 |
|  |  | % within Cat_prelim | 1.9% | 100.0% |
|  |  | % of Total | 1.9% | 100.0% |

| **Chi-Square Tests** | | | |
| --- | --- | --- | --- |
|  | Value | df | Asymp. Sig. (2-sided) |
| Pearson Chi-Square | 2.539^a^ | 4 | .638 |
| Likelihood Ratio | 3.730 | 4 | .444 |
| Linear-by-Linear Association | .173 | 1 | .677 |
| N of Valid Cases | 215 |  |  |

| a. 4 cells (40.0%) have expected count less than 5. The minimum expected count is .25. |
| --- |

**Cat_prelim * INT_one_on_one**

| **Crosstab** | | | | | | |
| --- | --- | --- | --- | --- | --- | --- |
|  | | | INT_one_on_one | | | |
|  |  |  | 1 | 2 | 4 | 5 |
| Cat_prelim | categorical | Count | 153 | 4 | 1 | 3 |
|  |  | % within Cat_prelim | 95.0% | 2.5% | 0.6% | 1.9% |
|  |  | % of Total | 71.2% | 1.9% | 0.5% | 1.4% |
|  | preliminary | Count | 52 | 2 | 0 | 0 |
|  |  | % within Cat_prelim | 96.3% | 3.7% | 0.0% | 0.0% |
|  |  | % of Total | 24.2% | 0.9% | 0.0% | 0.0% |
| Total | | Count | 205 | 6 | 1 | 3 |
|  |  | % within Cat_prelim | 95.3% | 2.8% | 0.5% | 1.4% |
|  |  | % of Total | 95.3% | 2.8% | 0.5% | 1.4% |

| **Crosstab** | | | |
| --- | --- | --- | --- |
|  | | | Total |
|  |  |  |  |
| Cat_prelim | categorical | Count | 161 |
|  |  | % within Cat_prelim | 100.0% |
|  |  | % of Total | 74.9% |
|  | preliminary | Count | 54 |
|  |  | % within Cat_prelim | 100.0% |
|  |  | % of Total | 25.1% |
| Total | | Count | 215 |
|  |  | % within Cat_prelim | 100.0% |
|  |  | % of Total | 100.0% |

| **Chi-Square Tests** | | | |
| --- | --- | --- | --- |
|  | Value | df | Asymp. Sig. (2-sided) |
| Pearson Chi-Square | 1.564^a^ | 3 | .668 |
| Likelihood Ratio | 2.523 | 3 | .471 |
| Linear-by-Linear Association | .931 | 1 | .335 |
| N of Valid Cases | 215 |  |  |

| a. 6 cells (75.0%) have expected count less than 5. The minimum expected count is .25. |
| --- |

**Cat_prelim * INT_panel**

| **Crosstab** | | | | | | |
| --- | --- | --- | --- | --- | --- | --- |
|  | | | INT_panel | | | |
|  |  |  | 1 | 2 | 3 | 4 |
| Cat_prelim | categorical | Count | 2 | 60 | 51 | 41 |
|  |  | % within Cat_prelim | 1.2% | 37.3% | 31.7% | 25.5% |
|  |  | % of Total | 0.9% | 27.9% | 23.7% | 19.1% |
|  | preliminary | Count | 0 | 15 | 10 | 24 |
|  |  | % within Cat_prelim | 0.0% | 27.8% | 18.5% | 44.4% |
|  |  | % of Total | 0.0% | 7.0% | 4.7% | 11.2% |
| Total | | Count | 2 | 75 | 61 | 65 |
|  |  | % within Cat_prelim | 0.9% | 34.9% | 28.4% | 30.2% |
|  |  | % of Total | 0.9% | 34.9% | 28.4% | 30.2% |

| **Crosstab** | | | | |
| --- | --- | --- | --- | --- |
|  | | | INT_panel | Total |
|  |  |  | 5 |  |
| Cat_prelim | categorical | Count | 7 | 161 |
|  |  | % within Cat_prelim | 4.3% | 100.0% |
|  |  | % of Total | 3.3% | 74.9% |
|  | preliminary | Count | 5 | 54 |
|  |  | % within Cat_prelim | 9.3% | 100.0% |
|  |  | % of Total | 2.3% | 25.1% |
| Total | | Count | 12 | 215 |
|  |  | % within Cat_prelim | 5.6% | 100.0% |
|  |  | % of Total | 5.6% | 100.0% |

| **Chi-Square Tests** | | | |
| --- | --- | --- | --- |
|  | Value | df | Asymp. Sig. (2-sided) |
| Pearson Chi-Square | 10.748^a^ | 4 | .030 |
| Likelihood Ratio | 10.951 | 4 | .027 |
| Linear-by-Linear Association | 7.358 | 1 | .007 |
| N of Valid Cases | 215 |  |  |

| a. 3 cells (30.0%) have expected count less than 5. The minimum expected count is .50. |
| --- |

**Cat_prelim * INT_group**

| **Crosstab** | | | | | | |
| --- | --- | --- | --- | --- | --- | --- |
|  | | | INT_group | | | |
|  |  |  | 1 | 2 | 3 | 4 |
| Cat_prelim | categorical | Count | 3 | 5 | 26 | 51 |
|  |  | % within Cat_prelim | 1.9% | 3.1% | 16.1% | 31.7% |
|  |  | % of Total | 1.4% | 2.3% | 12.1% | 23.7% |
|  | preliminary | Count | 0 | 0 | 7 | 11 |
|  |  | % within Cat_prelim | 0.0% | 0.0% | 13.0% | 20.4% |
|  |  | % of Total | 0.0% | 0.0% | 3.3% | 5.1% |
| Total | | Count | 3 | 5 | 33 | 62 |
|  |  | % within Cat_prelim | 1.4% | 2.3% | 15.3% | 28.8% |
|  |  | % of Total | 1.4% | 2.3% | 15.3% | 28.8% |

| **Crosstab** | | | | |
| --- | --- | --- | --- | --- |
|  | | | INT_group | Total |
|  |  |  | 5 |  |
| Cat_prelim | categorical | Count | 76 | 161 |
|  |  | % within Cat_prelim | 47.2% | 100.0% |
|  |  | % of Total | 35.3% | 74.9% |
|  | preliminary | Count | 36 | 54 |
|  |  | % within Cat_prelim | 66.7% | 100.0% |
|  |  | % of Total | 16.7% | 25.1% |
| Total | | Count | 112 | 215 |
|  |  | % within Cat_prelim | 52.1% | 100.0% |
|  |  | % of Total | 52.1% | 100.0% |

| **Chi-Square Tests** | | | |
| --- | --- | --- | --- |
|  | Value | df | Asymp. Sig. (2-sided) |
| Pearson Chi-Square | 7.683^a^ | 4 | .104 |
| Likelihood Ratio | 9.622 | 4 | .047 |
| Linear-by-Linear Association | 5.860 | 1 | .015 |
| N of Valid Cases | 215 |  |  |

| a. 4 cells (40.0%) have expected count less than 5. The minimum expected count is .75. |
| --- |

**Cat_prelim * start7_8**

| **Crosstab** | | | | | | |
| --- | --- | --- | --- | --- | --- | --- |
|  | | | start7_8 | | | |
|  |  |  | 1 | 2 | 3 | 4 |
| Cat_prelim | categorical | Count | 25 | 37 | 39 | 22 |
|  |  | % within Cat_prelim | 15.5% | 23.0% | 24.2% | 13.7% |
|  |  | % of Total | 11.6% | 17.2% | 18.1% | 10.2% |
|  | preliminary | Count | 7 | 8 | 17 | 8 |
|  |  | % within Cat_prelim | 13.0% | 14.8% | 31.5% | 14.8% |
|  |  | % of Total | 3.3% | 3.7% | 7.9% | 3.7% |
| Total | | Count | 32 | 45 | 56 | 30 |
|  |  | % within Cat_prelim | 14.9% | 20.9% | 26.0% | 14.0% |
|  |  | % of Total | 14.9% | 20.9% | 26.0% | 14.0% |

| **Crosstab** | | | | |
| --- | --- | --- | --- | --- |
|  | | | start7_8 | Total |
|  |  |  | 5 |  |
| Cat_prelim | categorical | Count | 38 | 161 |
|  |  | % within Cat_prelim | 23.6% | 100.0% |
|  |  | % of Total | 17.7% | 74.9% |
|  | preliminary | Count | 14 | 54 |
|  |  | % within Cat_prelim | 25.9% | 100.0% |
|  |  | % of Total | 6.5% | 25.1% |
| Total | | Count | 52 | 215 |
|  |  | % within Cat_prelim | 24.2% | 100.0% |
|  |  | % of Total | 24.2% | 100.0% |

| **Chi-Square Tests** | | | |
| --- | --- | --- | --- |
|  | Value | df | Asymp. Sig. (2-sided) |
| Pearson Chi-Square | 2.414^a^ | 4 | .660 |
| Likelihood Ratio | 2.484 | 4 | .647 |
| Linear-by-Linear Association | .773 | 1 | .379 |
| N of Valid Cases | 215 |  |  |

| a. 0 cells (0.0%) have expected count less than 5. The minimum expected count is 7.53. |
| --- |

**Cat_prelim * start8_9**

| **Crosstab** | | | | | | |
| --- | --- | --- | --- | --- | --- | --- |
|  | | | start8_9 | | | |
|  |  |  | 1 | 2 | 3 | 4 |
| Cat_prelim | categorical | Count | 84 | 47 | 9 | 17 |
|  |  | % within Cat_prelim | 52.2% | 29.2% | 5.6% | 10.6% |
|  |  | % of Total | 39.1% | 21.9% | 4.2% | 7.9% |
|  | preliminary | Count | 31 | 14 | 5 | 4 |
|  |  | % within Cat_prelim | 57.4% | 25.9% | 9.3% | 7.4% |
|  |  | % of Total | 14.4% | 6.5% | 2.3% | 1.9% |
| Total | | Count | 115 | 61 | 14 | 21 |
|  |  | % within Cat_prelim | 53.5% | 28.4% | 6.5% | 9.8% |
|  |  | % of Total | 53.5% | 28.4% | 6.5% | 9.8% |

| **Crosstab** | | | | |
| --- | --- | --- | --- | --- |
|  | | | start8_9 | Total |
|  |  |  | 5 |  |
| Cat_prelim | categorical | Count | 4 | 161 |
|  |  | % within Cat_prelim | 2.5% | 100.0% |
|  |  | % of Total | 1.9% | 74.9% |
|  | preliminary | Count | 0 | 54 |
|  |  | % within Cat_prelim | 0.0% | 100.0% |
|  |  | % of Total | 0.0% | 25.1% |
| Total | | Count | 4 | 215 |
|  |  | % within Cat_prelim | 1.9% | 100.0% |
|  |  | % of Total | 1.9% | 100.0% |

| **Chi-Square Tests** | | | |
| --- | --- | --- | --- |
|  | Value | df | Asymp. Sig. (2-sided) |
| Pearson Chi-Square | 2.948^a^ | 4 | .567 |
| Likelihood Ratio | 3.883 | 4 | .422 |
| Linear-by-Linear Association | .851 | 1 | .356 |
| N of Valid Cases | 215 |  |  |

| a. 3 cells (30.0%) have expected count less than 5. The minimum expected count is 1.00. |
| --- |

**Cat_prelim * start9_10**

| **Crosstab** | | | | | | |
| --- | --- | --- | --- | --- | --- | --- |
|  | | | start9_10 | | | |
|  |  |  | 1 | 2 | 3 | 4 |
| Cat_prelim | categorical | Count | 42 | 52 | 62 | 4 |
|  |  | % within Cat_prelim | 26.1% | 32.3% | 38.5% | 2.5% |
|  |  | % of Total | 19.5% | 24.2% | 28.8% | 1.9% |
|  | preliminary | Count | 14 | 24 | 14 | 1 |
|  |  | % within Cat_prelim | 25.9% | 44.4% | 25.9% | 1.9% |
|  |  | % of Total | 6.5% | 11.2% | 6.5% | 0.5% |
| Total | | Count | 56 | 76 | 76 | 5 |
|  |  | % within Cat_prelim | 26.0% | 35.3% | 35.3% | 2.3% |
|  |  | % of Total | 26.0% | 35.3% | 35.3% | 2.3% |

| **Crosstab** | | | | |
| --- | --- | --- | --- | --- |
|  | | | start9_10 | Total |
|  |  |  | 5 |  |
| Cat_prelim | categorical | Count | 1 | 161 |
|  |  | % within Cat_prelim | 0.6% | 100.0% |
|  |  | % of Total | 0.5% | 74.9% |
|  | preliminary | Count | 1 | 54 |
|  |  | % within Cat_prelim | 1.9% | 100.0% |
|  |  | % of Total | 0.5% | 25.1% |
| Total | | Count | 2 | 215 |
|  |  | % within Cat_prelim | 0.9% | 100.0% |
|  |  | % of Total | 0.9% | 100.0% |

| **Chi-Square Tests** | | | |
| --- | --- | --- | --- |
|  | Value | df | Asymp. Sig. (2-sided) |
| Pearson Chi-Square | 4.227^a^ | 4 | .376 |
| Likelihood Ratio | 4.185 | 4 | .382 |
| Linear-by-Linear Association | .527 | 1 | .468 |
| N of Valid Cases | 215 |  |  |

| a. 4 cells (40.0%) have expected count less than 5. The minimum expected count is .50. |
| --- |

**Cat_prelim * start10_11**

| **Crosstab** | | | | | | |
| --- | --- | --- | --- | --- | --- | --- |
|  | | | start10_11 | | | |
|  |  |  | 1 | 2 | 3 | 4 |
| Cat_prelim | categorical | Count | 5 | 21 | 39 | 95 |
|  |  | % within Cat_prelim | 3.1% | 13.0% | 24.2% | 59.0% |
|  |  | % of Total | 2.3% | 9.8% | 18.1% | 44.2% |
|  | preliminary | Count | 1 | 7 | 14 | 31 |
|  |  | % within Cat_prelim | 1.9% | 13.0% | 25.9% | 57.4% |
|  |  | % of Total | 0.5% | 3.3% | 6.5% | 14.4% |
| Total | | Count | 6 | 28 | 53 | 126 |
|  |  | % within Cat_prelim | 2.8% | 13.0% | 24.7% | 58.6% |
|  |  | % of Total | 2.8% | 13.0% | 24.7% | 58.6% |

| **Crosstab** | | | | |
| --- | --- | --- | --- | --- |
|  | | | start10_11 | Total |
|  |  |  | 5 |  |
| Cat_prelim | categorical | Count | 1 | 161 |
|  |  | % within Cat_prelim | 0.6% | 100.0% |
|  |  | % of Total | 0.5% | 74.9% |
|  | preliminary | Count | 1 | 54 |
|  |  | % within Cat_prelim | 1.9% | 100.0% |
|  |  | % of Total | 0.5% | 25.1% |
| Total | | Count | 2 | 215 |
|  |  | % within Cat_prelim | 0.9% | 100.0% |
|  |  | % of Total | 0.9% | 100.0% |

| **Chi-Square Tests** | | | |
| --- | --- | --- | --- |
|  | Value | df | Asymp. Sig. (2-sided) |
| Pearson Chi-Square | .952^a^ | 4 | .917 |
| Likelihood Ratio | .883 | 4 | .927 |
| Linear-by-Linear Association | .069 | 1 | .792 |
| N of Valid Cases | 215 |  |  |

| a. 4 cells (40.0%) have expected count less than 5. The minimum expected count is .50. |
| --- |

**Cat_prelim * start11_12**

| **Crosstab** | | | | | | |
| --- | --- | --- | --- | --- | --- | --- |
|  | | | start11_12 | | | |
|  |  |  | 1 | 2 | 3 | 4 |
| Cat_prelim | categorical | Count | 5 | 4 | 12 | 23 |
|  |  | % within Cat_prelim | 3.1% | 2.5% | 7.5% | 14.3% |
|  |  | % of Total | 2.3% | 1.9% | 5.6% | 10.7% |
|  | preliminary | Count | 1 | 1 | 4 | 10 |
|  |  | % within Cat_prelim | 1.9% | 1.9% | 7.4% | 18.5% |
|  |  | % of Total | 0.5% | 0.5% | 1.9% | 4.7% |
| Total | | Count | 6 | 5 | 16 | 33 |
|  |  | % within Cat_prelim | 2.8% | 2.3% | 7.4% | 15.3% |
|  |  | % of Total | 2.8% | 2.3% | 7.4% | 15.3% |

| **Crosstab** | | | | |
| --- | --- | --- | --- | --- |
|  | | | start11_12 | Total |
|  |  |  | 5 |  |
| Cat_prelim | categorical | Count | 117 | 161 |
|  |  | % within Cat_prelim | 72.7% | 100.0% |
|  |  | % of Total | 54.4% | 74.9% |
|  | preliminary | Count | 38 | 54 |
|  |  | % within Cat_prelim | 70.4% | 100.0% |
|  |  | % of Total | 17.7% | 25.1% |
| Total | | Count | 155 | 215 |
|  |  | % within Cat_prelim | 72.1% | 100.0% |
|  |  | % of Total | 72.1% | 100.0% |

| **Chi-Square Tests** | | | |
| --- | --- | --- | --- |
|  | Value | df | Asymp. Sig. (2-sided) |
| Pearson Chi-Square | .799^a^ | 4 | .939 |
| Likelihood Ratio | .805 | 4 | .938 |
| Linear-by-Linear Association | .035 | 1 | .851 |
| N of Valid Cases | 215 |  |  |

| a. 5 cells (50.0%) have expected count less than 5. The minimum expected count is 1.26. |
| --- |

**Cat_prelim * lengthLT2**

| **Crosstab** | | | | | | |
| --- | --- | --- | --- | --- | --- | --- |
|  | | | lengthLT2 | | | |
|  |  |  | 1 | 2 | 3 | 4 |
| Cat_prelim | categorical | Count | 3 | 8 | 21 | 50 |
|  |  | % within Cat_prelim | 1.9% | 5.0% | 13.0% | 31.1% |
|  |  | % of Total | 1.4% | 3.7% | 9.8% | 23.3% |
|  | preliminary | Count | 3 | 7 | 18 | 18 |
|  |  | % within Cat_prelim | 5.6% | 13.0% | 33.3% | 33.3% |
|  |  | % of Total | 1.4% | 3.3% | 8.4% | 8.4% |
| Total | | Count | 6 | 15 | 39 | 68 |
|  |  | % within Cat_prelim | 2.8% | 7.0% | 18.1% | 31.6% |
|  |  | % of Total | 2.8% | 7.0% | 18.1% | 31.6% |

| **Crosstab** | | | | |
| --- | --- | --- | --- | --- |
|  | | | lengthLT2 | Total |
|  |  |  | 5 |  |
| Cat_prelim | categorical | Count | 79 | 161 |
|  |  | % within Cat_prelim | 49.1% | 100.0% |
|  |  | % of Total | 36.7% | 74.9% |
|  | preliminary | Count | 8 | 54 |
|  |  | % within Cat_prelim | 14.8% | 100.0% |
|  |  | % of Total | 3.7% | 25.1% |
| Total | | Count | 87 | 215 |
|  |  | % within Cat_prelim | 40.5% | 100.0% |
|  |  | % of Total | 40.5% | 100.0% |

| **Chi-Square Tests** | | | |
| --- | --- | --- | --- |
|  | Value | df | Asymp. Sig. (2-sided) |
| Pearson Chi-Square | 26.648^a^ | 4 | .000 |
| Likelihood Ratio | 27.450 | 4 | .000 |
| Linear-by-Linear Association | 24.013 | 1 | .000 |
| N of Valid Cases | 215 |  |  |

| a. 3 cells (30.0%) have expected count less than 5. The minimum expected count is 1.51. |
| --- |

**Cat_prelim * length2**

| **Crosstab** | | | | | | |
| --- | --- | --- | --- | --- | --- | --- |
|  | | | length2 | | | |
|  |  |  | 1 | 2 | 3 | 4 |
| Cat_prelim | categorical | Count | 15 | 39 | 55 | 52 |
|  |  | % within Cat_prelim | 9.3% | 24.2% | 34.2% | 32.3% |
|  |  | % of Total | 7.0% | 18.1% | 25.6% | 24.2% |
|  | preliminary | Count | 16 | 22 | 14 | 2 |
|  |  | % within Cat_prelim | 29.6% | 40.7% | 25.9% | 3.7% |
|  |  | % of Total | 7.4% | 10.2% | 6.5% | 0.9% |
| Total | | Count | 31 | 61 | 69 | 54 |
|  |  | % within Cat_prelim | 14.4% | 28.4% | 32.1% | 25.1% |
|  |  | % of Total | 14.4% | 28.4% | 32.1% | 25.1% |

| **Crosstab** | | | |
| --- | --- | --- | --- |
|  | | | Total |
|  |  |  |  |
| Cat_prelim | categorical | Count | 161 |
|  |  | % within Cat_prelim | 100.0% |
|  |  | % of Total | 74.9% |
|  | preliminary | Count | 54 |
|  |  | % within Cat_prelim | 100.0% |
|  |  | % of Total | 25.1% |
| Total | | Count | 215 |
|  |  | % within Cat_prelim | 100.0% |
|  |  | % of Total | 100.0% |

| **Chi-Square Tests** | | | |
| --- | --- | --- | --- |
|  | Value | df | Asymp. Sig. (2-sided) |
| Pearson Chi-Square | 29.479^a^ | 3 | .000 |
| Likelihood Ratio | 32.931 | 3 | .000 |
| Linear-by-Linear Association | 29.333 | 1 | .000 |
| N of Valid Cases | 215 |  |  |

| a. 0 cells (0.0%) have expected count less than 5. The minimum expected count is 7.79. |
| --- |

**Cat_prelim * length4**

| **Crosstab** | | | | | | |
| --- | --- | --- | --- | --- | --- | --- |
|  | | | length4 | | | |
|  |  |  | 1 | 2 | 3 | 4 |
| Cat_prelim | categorical | Count | 95 | 40 | 22 | 2 |
|  |  | % within Cat_prelim | 59.0% | 24.8% | 13.7% | 1.2% |
|  |  | % of Total | 44.2% | 18.6% | 10.2% | 0.9% |
|  | preliminary | Count | 33 | 10 | 11 | 0 |
|  |  | % within Cat_prelim | 61.1% | 18.5% | 20.4% | 0.0% |
|  |  | % of Total | 15.3% | 4.7% | 5.1% | 0.0% |
| Total | | Count | 128 | 50 | 33 | 2 |
|  |  | % within Cat_prelim | 59.5% | 23.3% | 15.3% | 0.9% |
|  |  | % of Total | 59.5% | 23.3% | 15.3% | 0.9% |

| **Crosstab** | | | | |
| --- | --- | --- | --- | --- |
|  | | | length4 | Total |
|  |  |  | 5 |  |
| Cat_prelim | categorical | Count | 2 | 161 |
|  |  | % within Cat_prelim | 1.2% | 100.0% |
|  |  | % of Total | 0.9% | 74.9% |
|  | preliminary | Count | 0 | 54 |
|  |  | % within Cat_prelim | 0.0% | 100.0% |
|  |  | % of Total | 0.0% | 25.1% |
| Total | | Count | 2 | 215 |
|  |  | % within Cat_prelim | 0.9% | 100.0% |
|  |  | % of Total | 0.9% | 100.0% |

| **Chi-Square Tests** | | | |
| --- | --- | --- | --- |
|  | Value | df | Asymp. Sig. (2-sided) |
| Pearson Chi-Square | 3.252^a^ | 4 | .517 |
| Likelihood Ratio | 4.188 | 4 | .381 |
| Linear-by-Linear Association | .015 | 1 | .904 |
| N of Valid Cases | 215 |  |  |

| a. 4 cells (40.0%) have expected count less than 5. The minimum expected count is .50. |
| --- |

**Cat_prelim * length6**

| **Crosstab** | | | | | | |
| --- | --- | --- | --- | --- | --- | --- |
|  | | | length6 | | | |
|  |  |  | 1 | 2 | 3 | 4 |
| Cat_prelim | categorical | Count | 42 | 62 | 30 | 25 |
|  |  | % within Cat_prelim | 26.1% | 38.5% | 18.6% | 15.5% |
|  |  | % of Total | 19.5% | 28.8% | 14.0% | 11.6% |
|  | preliminary | Count | 2 | 14 | 10 | 28 |
|  |  | % within Cat_prelim | 3.7% | 25.9% | 18.5% | 51.9% |
|  |  | % of Total | 0.9% | 6.5% | 4.7% | 13.0% |
| Total | | Count | 44 | 76 | 40 | 53 |
|  |  | % within Cat_prelim | 20.5% | 35.3% | 18.6% | 24.7% |
|  |  | % of Total | 20.5% | 35.3% | 18.6% | 24.7% |

| **Crosstab** | | | | |
| --- | --- | --- | --- | --- |
|  | | | length6 | Total |
|  |  |  | 5 |  |
| Cat_prelim | categorical | Count | 2 | 161 |
|  |  | % within Cat_prelim | 1.2% | 100.0% |
|  |  | % of Total | 0.9% | 74.9% |
|  | preliminary | Count | 0 | 54 |
|  |  | % within Cat_prelim | 0.0% | 100.0% |
|  |  | % of Total | 0.0% | 25.1% |
| Total | | Count | 2 | 215 |
|  |  | % within Cat_prelim | 0.9% | 100.0% |
|  |  | % of Total | 0.9% | 100.0% |

| **Chi-Square Tests** | | | |
| --- | --- | --- | --- |
|  | Value | df | Asymp. Sig. (2-sided) |
| Pearson Chi-Square | 34.026^a^ | 4 | .000 |
| Likelihood Ratio | 35.176 | 4 | .000 |
| Linear-by-Linear Association | 27.703 | 1 | .000 |
| N of Valid Cases | 215 |  |  |

| a. 2 cells (20.0%) have expected count less than 5. The minimum expected count is .50. |
| --- |

**Cat_prelim * length8**

| **Crosstab** | | | | | | |
| --- | --- | --- | --- | --- | --- | --- |
|  | | | length8 | | | |
|  |  |  | 1 | 2 | 3 | 4 |
| Cat_prelim | categorical | Count | 6 | 12 | 33 | 32 |
|  |  | % within Cat_prelim | 3.7% | 7.5% | 20.5% | 19.9% |
|  |  | % of Total | 2.8% | 5.6% | 15.3% | 14.9% |
|  | preliminary | Count | 0 | 1 | 1 | 6 |
|  |  | % within Cat_prelim | 0.0% | 1.9% | 1.9% | 11.1% |
|  |  | % of Total | 0.0% | 0.5% | 0.5% | 2.8% |
| Total | | Count | 6 | 13 | 34 | 38 |
|  |  | % within Cat_prelim | 2.8% | 6.0% | 15.8% | 17.7% |
|  |  | % of Total | 2.8% | 6.0% | 15.8% | 17.7% |

| **Crosstab** | | | | |
| --- | --- | --- | --- | --- |
|  | | | length8 | Total |
|  |  |  | 5 |  |
| Cat_prelim | categorical | Count | 78 | 161 |
|  |  | % within Cat_prelim | 48.4% | 100.0% |
|  |  | % of Total | 36.3% | 74.9% |
|  | preliminary | Count | 46 | 54 |
|  |  | % within Cat_prelim | 85.2% | 100.0% |
|  |  | % of Total | 21.4% | 25.1% |
| Total | | Count | 124 | 215 |
|  |  | % within Cat_prelim | 57.7% | 100.0% |
|  |  | % of Total | 57.7% | 100.0% |

| **Chi-Square Tests** | | | |
| --- | --- | --- | --- |
|  | Value | df | Asymp. Sig. (2-sided) |
| Pearson Chi-Square | 24.221^a^ | 4 | .000 |
| Likelihood Ratio | 29.581 | 4 | .000 |
| Linear-by-Linear Association | 20.591 | 1 | .000 |
| N of Valid Cases | 215 |  |  |

| a. 3 cells (30.0%) have expected count less than 5. The minimum expected count is 1.51. |
| --- |

**Cat_prelim * faculty1**

| **Crosstab** | | | | | | |
| --- | --- | --- | --- | --- | --- | --- |
|  | | | faculty1 | | | |
|  |  |  | 1 | 2 | 3 | 4 |
| Cat_prelim | categorical | Count | 7 | 28 | 64 | 37 |
|  |  | % within Cat_prelim | 4.3% | 17.4% | 39.8% | 23.0% |
|  |  | % of Total | 3.3% | 13.0% | 29.8% | 17.2% |
|  | preliminary | Count | 3 | 11 | 17 | 14 |
|  |  | % within Cat_prelim | 5.6% | 20.4% | 31.5% | 25.9% |
|  |  | % of Total | 1.4% | 5.1% | 7.9% | 6.5% |
| Total | | Count | 10 | 39 | 81 | 51 |
|  |  | % within Cat_prelim | 4.7% | 18.1% | 37.7% | 23.7% |
|  |  | % of Total | 4.7% | 18.1% | 37.7% | 23.7% |

| **Crosstab** | | | | |
| --- | --- | --- | --- | --- |
|  | | | faculty1 | Total |
|  |  |  | 5 |  |
| Cat_prelim | categorical | Count | 25 | 161 |
|  |  | % within Cat_prelim | 15.5% | 100.0% |
|  |  | % of Total | 11.6% | 74.9% |
|  | preliminary | Count | 9 | 54 |
|  |  | % within Cat_prelim | 16.7% | 100.0% |
|  |  | % of Total | 4.2% | 25.1% |
| Total | | Count | 34 | 215 |
|  |  | % within Cat_prelim | 15.8% | 100.0% |
|  |  | % of Total | 15.8% | 100.0% |

| **Chi-Square Tests** | | | |
| --- | --- | --- | --- |
|  | Value | df | Asymp. Sig. (2-sided) |
| Pearson Chi-Square | 1.240^a^ | 4 | .872 |
| Likelihood Ratio | 1.256 | 4 | .869 |
| Linear-by-Linear Association | .000 | 1 | .992 |
| N of Valid Cases | 215 |  |  |

| a. 1 cells (10.0%) have expected count less than 5. The minimum expected count is 2.51. |
| --- |

**Cat_prelim * faculty2**

| **Crosstab** | | | | | | |
| --- | --- | --- | --- | --- | --- | --- |
|  | | | faculty2 | | | |
|  |  |  | 1 | 2 | 3 | 4 |
| Cat_prelim | categorical | Count | 113 | 32 | 7 | 5 |
|  |  | % within Cat_prelim | 70.2% | 19.9% | 4.3% | 3.1% |
|  |  | % of Total | 52.6% | 14.9% | 3.3% | 2.3% |
|  | preliminary | Count | 34 | 16 | 2 | 2 |
|  |  | % within Cat_prelim | 63.0% | 29.6% | 3.7% | 3.7% |
|  |  | % of Total | 15.8% | 7.4% | 0.9% | 0.9% |
| Total | | Count | 147 | 48 | 9 | 7 |
|  |  | % within Cat_prelim | 68.4% | 22.3% | 4.2% | 3.3% |
|  |  | % of Total | 68.4% | 22.3% | 4.2% | 3.3% |

| **Crosstab** | | | | |
| --- | --- | --- | --- | --- |
|  | | | faculty2 | Total |
|  |  |  | 5 |  |
| Cat_prelim | categorical | Count | 4 | 161 |
|  |  | % within Cat_prelim | 2.5% | 100.0% |
|  |  | % of Total | 1.9% | 74.9% |
|  | preliminary | Count | 0 | 54 |
|  |  | % within Cat_prelim | 0.0% | 100.0% |
|  |  | % of Total | 0.0% | 25.1% |
| Total | | Count | 4 | 215 |
|  |  | % within Cat_prelim | 1.9% | 100.0% |
|  |  | % of Total | 1.9% | 100.0% |

| **Chi-Square Tests** | | | |
| --- | --- | --- | --- |
|  | Value | df | Asymp. Sig. (2-sided) |
| Pearson Chi-Square | 3.458^a^ | 4 | .484 |
| Likelihood Ratio | 4.331 | 4 | .363 |
| Linear-by-Linear Association | .001 | 1 | .981 |
| N of Valid Cases | 215 |  |  |

| a. 4 cells (40.0%) have expected count less than 5. The minimum expected count is 1.00. |
| --- |

**Cat_prelim * faculty3**

| **Crosstab** | | | | | | |
| --- | --- | --- | --- | --- | --- | --- |
|  | | | faculty3 | | | |
|  |  |  | 1 | 2 | 3 | 4 |
| Cat_prelim | categorical | Count | 33 | 89 | 37 | 2 |
|  |  | % within Cat_prelim | 20.5% | 55.3% | 23.0% | 1.2% |
|  |  | % of Total | 15.3% | 41.4% | 17.2% | 0.9% |
|  | preliminary | Count | 17 | 23 | 14 | 0 |
|  |  | % within Cat_prelim | 31.5% | 42.6% | 25.9% | 0.0% |
|  |  | % of Total | 7.9% | 10.7% | 6.5% | 0.0% |
| Total | | Count | 50 | 112 | 51 | 2 |
|  |  | % within Cat_prelim | 23.3% | 52.1% | 23.7% | 0.9% |
|  |  | % of Total | 23.3% | 52.1% | 23.7% | 0.9% |

| **Crosstab** | | | |
| --- | --- | --- | --- |
|  | | | Total |
|  |  |  |  |
| Cat_prelim | categorical | Count | 161 |
|  |  | % within Cat_prelim | 100.0% |
|  |  | % of Total | 74.9% |
|  | preliminary | Count | 54 |
|  |  | % within Cat_prelim | 100.0% |
|  |  | % of Total | 25.1% |
| Total | | Count | 215 |
|  |  | % within Cat_prelim | 100.0% |
|  |  | % of Total | 100.0% |

| **Chi-Square Tests** | | | |
| --- | --- | --- | --- |
|  | Value | df | Asymp. Sig. (2-sided) |
| Pearson Chi-Square | 4.166^a^ | 3 | .244 |
| Likelihood Ratio | 4.570 | 3 | .206 |
| Linear-by-Linear Association | .880 | 1 | .348 |
| N of Valid Cases | 215 |  |  |

| a. 2 cells (25.0%) have expected count less than 5. The minimum expected count is .50. |
| --- |

**Cat_prelim * faculty4**

| **Crosstab** | | | | | | |
| --- | --- | --- | --- | --- | --- | --- |
|  | | | faculty4 | | | |
|  |  |  | 1 | 2 | 3 | 4 |
| Cat_prelim | categorical | Count | 4 | 11 | 49 | 97 |
|  |  | % within Cat_prelim | 2.5% | 6.8% | 30.4% | 60.2% |
|  |  | % of Total | 1.9% | 5.1% | 22.8% | 45.1% |
|  | preliminary | Count | 0 | 4 | 19 | 31 |
|  |  | % within Cat_prelim | 0.0% | 7.4% | 35.2% | 57.4% |
|  |  | % of Total | 0.0% | 1.9% | 8.8% | 14.4% |
| Total | | Count | 4 | 15 | 68 | 128 |
|  |  | % within Cat_prelim | 1.9% | 7.0% | 31.6% | 59.5% |
|  |  | % of Total | 1.9% | 7.0% | 31.6% | 59.5% |

| **Crosstab** | | | |
| --- | --- | --- | --- |
|  | | | Total |
|  |  |  |  |
| Cat_prelim | categorical | Count | 161 |
|  |  | % within Cat_prelim | 100.0% |
|  |  | % of Total | 74.9% |
|  | preliminary | Count | 54 |
|  |  | % within Cat_prelim | 100.0% |
|  |  | % of Total | 25.1% |
| Total | | Count | 215 |
|  |  | % within Cat_prelim | 100.0% |
|  |  | % of Total | 100.0% |

| **Chi-Square Tests** | | | |
| --- | --- | --- | --- |
|  | Value | df | Asymp. Sig. (2-sided) |
| Pearson Chi-Square | 1.704^a^ | 3 | .636 |
| Likelihood Ratio | 2.670 | 3 | .445 |
| Linear-by-Linear Association | .019 | 1 | .889 |
| N of Valid Cases | 215 |  |  |

| a. 3 cells (37.5%) have expected count less than 5. The minimum expected count is 1.00. |
| --- |

**Cat_prelim * facultyGT4**

| **Crosstab** | | | | | | |
| --- | --- | --- | --- | --- | --- | --- |
|  | | | facultyGT4 | | | |
|  |  |  | 1 | 2 | 3 | 4 |
| Cat_prelim | categorical | Count | 4 | 1 | 4 | 20 |
|  |  | % within Cat_prelim | 2.5% | 0.6% | 2.5% | 12.4% |
|  |  | % of Total | 1.9% | 0.5% | 1.9% | 9.3% |
|  | preliminary | Count | 0 | 0 | 2 | 7 |
|  |  | % within Cat_prelim | 0.0% | 0.0% | 3.7% | 13.0% |
|  |  | % of Total | 0.0% | 0.0% | 0.9% | 3.3% |
| Total | | Count | 4 | 1 | 6 | 27 |
|  |  | % within Cat_prelim | 1.9% | 0.5% | 2.8% | 12.6% |
|  |  | % of Total | 1.9% | 0.5% | 2.8% | 12.6% |

| **Crosstab** | | | | |
| --- | --- | --- | --- | --- |
|  | | | facultyGT4 | Total |
|  |  |  | 5 |  |
| Cat_prelim | categorical | Count | 132 | 161 |
|  |  | % within Cat_prelim | 82.0% | 100.0% |
|  |  | % of Total | 61.4% | 74.9% |
|  | preliminary | Count | 45 | 54 |
|  |  | % within Cat_prelim | 83.3% | 100.0% |
|  |  | % of Total | 20.9% | 25.1% |
| Total | | Count | 177 | 215 |
|  |  | % within Cat_prelim | 82.3% | 100.0% |
|  |  | % of Total | 82.3% | 100.0% |

| **Chi-Square Tests** | | | |
| --- | --- | --- | --- |
|  | Value | df | Asymp. Sig. (2-sided) |
| Pearson Chi-Square | 1.911^a^ | 4 | .752 |
| Likelihood Ratio | 3.113 | 4 | .539 |
| Linear-by-Linear Association | .622 | 1 | .430 |
| N of Valid Cases | 215 |  |  |

| a. 6 cells (60.0%) have expected count less than 5. The minimum expected count is .25. |
| --- |

**Cat_prelim * lengthLT15**

| **Crosstab** | | | | | | |
| --- | --- | --- | --- | --- | --- | --- |
|  | | | lengthLT15 | | | |
|  |  |  | 1 | 2 | 3 | 4 |
| Cat_prelim | categorical | Count | 12 | 65 | 50 | 18 |
|  |  | % within Cat_prelim | 7.5% | 40.4% | 31.1% | 11.2% |
|  |  | % of Total | 5.6% | 30.2% | 23.3% | 8.4% |
|  | preliminary | Count | 10 | 33 | 10 | 1 |
|  |  | % within Cat_prelim | 18.5% | 61.1% | 18.5% | 1.9% |
|  |  | % of Total | 4.7% | 15.3% | 4.7% | 0.5% |
| Total | | Count | 22 | 98 | 60 | 19 |
|  |  | % within Cat_prelim | 10.2% | 45.6% | 27.9% | 8.8% |
|  |  | % of Total | 10.2% | 45.6% | 27.9% | 8.8% |

| **Crosstab** | | | | |
| --- | --- | --- | --- | --- |
|  | | | lengthLT15 | Total |
|  |  |  | 5 |  |
| Cat_prelim | categorical | Count | 16 | 161 |
|  |  | % within Cat_prelim | 9.9% | 100.0% |
|  |  | % of Total | 7.4% | 74.9% |
|  | preliminary | Count | 0 | 54 |
|  |  | % within Cat_prelim | 0.0% | 100.0% |
|  |  | % of Total | 0.0% | 25.1% |
| Total | | Count | 16 | 215 |
|  |  | % within Cat_prelim | 7.4% | 100.0% |
|  |  | % of Total | 7.4% | 100.0% |

| **Chi-Square Tests** | | | |
| --- | --- | --- | --- |
|  | Value | df | Asymp. Sig. (2-sided) |
| Pearson Chi-Square | 20.280^a^ | 4 | .000 |
| Likelihood Ratio | 24.919 | 4 | .000 |
| Linear-by-Linear Association | 19.503 | 1 | .000 |
| N of Valid Cases | 215 |  |  |

| a. 2 cells (20.0%) have expected count less than 5. The minimum expected count is 4.02. |
| --- |

**Cat_prelim * length15_30**

| **Crosstab** | | | | | | |
| --- | --- | --- | --- | --- | --- | --- |
|  | | | length15_30 | | | |
|  |  |  | 1 | 2 | 3 | 4 |
| Cat_prelim | categorical | Count | 123 | 28 | 5 | 4 |
|  |  | % within Cat_prelim | 76.4% | 17.4% | 3.1% | 2.5% |
|  |  | % of Total | 57.2% | 13.0% | 2.3% | 1.9% |
|  | preliminary | Count | 43 | 11 | 0 | 0 |
|  |  | % within Cat_prelim | 79.6% | 20.4% | 0.0% | 0.0% |
|  |  | % of Total | 20.0% | 5.1% | 0.0% | 0.0% |
| Total | | Count | 166 | 39 | 5 | 4 |
|  |  | % within Cat_prelim | 77.2% | 18.1% | 2.3% | 1.9% |
|  |  | % of Total | 77.2% | 18.1% | 2.3% | 1.9% |

| **Crosstab** | | | | |
| --- | --- | --- | --- | --- |
|  | | | length15_30 | Total |
|  |  |  | 5 |  |
| Cat_prelim | categorical | Count | 1 | 161 |
|  |  | % within Cat_prelim | 0.6% | 100.0% |
|  |  | % of Total | 0.5% | 74.9% |
|  | preliminary | Count | 0 | 54 |
|  |  | % within Cat_prelim | 0.0% | 100.0% |
|  |  | % of Total | 0.0% | 25.1% |
| Total | | Count | 1 | 215 |
|  |  | % within Cat_prelim | 0.5% | 100.0% |
|  |  | % of Total | 0.5% | 100.0% |

| **Chi-Square Tests** | | | |
| --- | --- | --- | --- |
|  | Value | df | Asymp. Sig. (2-sided) |
| Pearson Chi-Square | 3.607^a^ | 4 | .462 |
| Likelihood Ratio | 6.032 | 4 | .197 |
| Linear-by-Linear Association | 1.643 | 1 | .200 |
| N of Valid Cases | 215 |  |  |

| a. 6 cells (60.0%) have expected count less than 5. The minimum expected count is .25. |
| --- |

**Cat_prelim * length30_45**

| **Crosstab** | | | | | | |
| --- | --- | --- | --- | --- | --- | --- |
|  | | | length30_45 | | | |
|  |  |  | 1 | 2 | 3 | 4 |
| Cat_prelim | categorical | Count | 20 | 61 | 79 | 1 |
|  |  | % within Cat_prelim | 12.4% | 37.9% | 49.1% | 0.6% |
|  |  | % of Total | 9.3% | 28.4% | 36.7% | 0.5% |
|  | preliminary | Count | 1 | 10 | 43 | 0 |
|  |  | % within Cat_prelim | 1.9% | 18.5% | 79.6% | 0.0% |
|  |  | % of Total | 0.5% | 4.7% | 20.0% | 0.0% |
| Total | | Count | 21 | 71 | 122 | 1 |
|  |  | % within Cat_prelim | 9.8% | 33.0% | 56.7% | 0.5% |
|  |  | % of Total | 9.8% | 33.0% | 56.7% | 0.5% |

| **Crosstab** | | | |
| --- | --- | --- | --- |
|  | | | Total |
|  |  |  |  |
| Cat_prelim | categorical | Count | 161 |
|  |  | % within Cat_prelim | 100.0% |
|  |  | % of Total | 74.9% |
|  | preliminary | Count | 54 |
|  |  | % within Cat_prelim | 100.0% |
|  |  | % of Total | 25.1% |
| Total | | Count | 215 |
|  |  | % within Cat_prelim | 100.0% |
|  |  | % of Total | 100.0% |

| **Chi-Square Tests** | | | |
| --- | --- | --- | --- |
|  | Value | df | Asymp. Sig. (2-sided) |
| Pearson Chi-Square | 16.211^a^ | 3 | .001 |
| Likelihood Ratio | 18.244 | 3 | .000 |
| Linear-by-Linear Association | 14.100 | 1 | .000 |
| N of Valid Cases | 215 |  |  |

| a. 2 cells (25.0%) have expected count less than 5. The minimum expected count is .25. |
| --- |

**Cat_prelim * length45_60**

| **Crosstab** | | | | | | |
| --- | --- | --- | --- | --- | --- | --- |
|  | | | length45_60 | | | |
|  |  |  | 1 | 2 | 3 | 4 |
| Cat_prelim | categorical | Count | 3 | 6 | 27 | 125 |
|  |  | % within Cat_prelim | 1.9% | 3.7% | 16.8% | 77.6% |
|  |  | % of Total | 1.4% | 2.8% | 12.6% | 58.1% |
|  | preliminary | Count | 0 | 0 | 1 | 53 |
|  |  | % within Cat_prelim | 0.0% | 0.0% | 1.9% | 98.1% |
|  |  | % of Total | 0.0% | 0.0% | 0.5% | 24.7% |
| Total | | Count | 3 | 6 | 28 | 178 |
|  |  | % within Cat_prelim | 1.4% | 2.8% | 13.0% | 82.8% |
|  |  | % of Total | 1.4% | 2.8% | 13.0% | 82.8% |

| **Crosstab** | | | |
| --- | --- | --- | --- |
|  | | | Total |
|  |  |  |  |
| Cat_prelim | categorical | Count | 161 |
|  |  | % within Cat_prelim | 100.0% |
|  |  | % of Total | 74.9% |
|  | preliminary | Count | 54 |
|  |  | % within Cat_prelim | 100.0% |
|  |  | % of Total | 25.1% |
| Total | | Count | 215 |
|  |  | % within Cat_prelim | 100.0% |
|  |  | % of Total | 100.0% |

| **Chi-Square Tests** | | | |
| --- | --- | --- | --- |
|  | Value | df | Asymp. Sig. (2-sided) |
| Pearson Chi-Square | 11.983^a^ | 3 | .007 |
| Likelihood Ratio | 16.938 | 3 | .001 |
| Linear-by-Linear Association | 9.974 | 1 | .002 |
| N of Valid Cases | 215 |  |  |

| a. 4 cells (50.0%) have expected count less than 5. The minimum expected count is .75. |
| --- |

**Cat_prelim * lengthGT60**

| **Crosstab** | | | | | | |
| --- | --- | --- | --- | --- | --- | --- |
|  | | | lengthGT60 | | | |
|  |  |  | 1 | 2 | 4 | 5 |
| Cat_prelim | categorical | Count | 4 | 1 | 13 | 143 |
|  |  | % within Cat_prelim | 2.5% | 0.6% | 8.1% | 88.8% |
|  |  | % of Total | 1.9% | 0.5% | 6.0% | 66.5% |
|  | preliminary | Count | 0 | 0 | 0 | 54 |
|  |  | % within Cat_prelim | 0.0% | 0.0% | 0.0% | 100.0% |
|  |  | % of Total | 0.0% | 0.0% | 0.0% | 25.1% |
| Total | | Count | 4 | 1 | 13 | 197 |
|  |  | % within Cat_prelim | 1.9% | 0.5% | 6.0% | 91.6% |
|  |  | % of Total | 1.9% | 0.5% | 6.0% | 91.6% |

| **Crosstab** | | | |
| --- | --- | --- | --- |
|  | | | Total |
|  |  |  |  |
| Cat_prelim | categorical | Count | 161 |
|  |  | % within Cat_prelim | 100.0% |
|  |  | % of Total | 74.9% |
|  | preliminary | Count | 54 |
|  |  | % within Cat_prelim | 100.0% |
|  |  | % of Total | 25.1% |
| Total | | Count | 215 |
|  |  | % within Cat_prelim | 100.0% |
|  |  | % of Total | 100.0% |

| **Chi-Square Tests** | | | |
| --- | --- | --- | --- |
|  | Value | df | Asymp. Sig. (2-sided) |
| Pearson Chi-Square | 6.589^a^ | 3 | .086 |
| Likelihood Ratio | 10.953 | 3 | .012 |
| Linear-by-Linear Association | 4.208 | 1 | .040 |
| N of Valid Cases | 215 |  |  |

| a. 5 cells (62.5%) have expected count less than 5. The minimum expected count is .25. |
| --- |

**Cat_prelim * INT_res_chief**

| **Crosstab** | | | | | | |
| --- | --- | --- | --- | --- | --- | --- |
|  | | | INT_res_chief | | | |
|  |  |  | 1 | 2 | 3 | 4 |
| Cat_prelim | categorical | Count | 5 | 18 | 21 | 54 |
|  |  | % within Cat_prelim | 3.1% | 11.2% | 13.0% | 33.5% |
|  |  | % of Total | 2.3% | 8.4% | 9.8% | 25.1% |
|  | preliminary | Count | 2 | 3 | 12 | 17 |
|  |  | % within Cat_prelim | 3.7% | 5.6% | 22.2% | 31.5% |
|  |  | % of Total | 0.9% | 1.4% | 5.6% | 7.9% |
| Total | | Count | 7 | 21 | 33 | 71 |
|  |  | % within Cat_prelim | 3.3% | 9.8% | 15.3% | 33.0% |
|  |  | % of Total | 3.3% | 9.8% | 15.3% | 33.0% |

| **Crosstab** | | | | |
| --- | --- | --- | --- | --- |
|  | | | INT_res_chief | Total |
|  |  |  | 5 |  |
| Cat_prelim | categorical | Count | 63 | 161 |
|  |  | % within Cat_prelim | 39.1% | 100.0% |
|  |  | % of Total | 29.3% | 74.9% |
|  | preliminary | Count | 20 | 54 |
|  |  | % within Cat_prelim | 37.0% | 100.0% |
|  |  | % of Total | 9.3% | 25.1% |
| Total | | Count | 83 | 215 |
|  |  | % within Cat_prelim | 38.6% | 100.0% |
|  |  | % of Total | 38.6% | 100.0% |

| **Chi-Square Tests** | | | |
| --- | --- | --- | --- |
|  | Value | df | Asymp. Sig. (2-sided) |
| Pearson Chi-Square | 3.672^a^ | 4 | .452 |
| Likelihood Ratio | 3.665 | 4 | .453 |
| Linear-by-Linear Association | .011 | 1 | .917 |
| N of Valid Cases | 215 |  |  |

| a. 1 cells (10.0%) have expected count less than 5. The minimum expected count is 1.76. |
| --- |

**Cat_prelim * INT_faculty**

| **Crosstab** | | | | | | |
| --- | --- | --- | --- | --- | --- | --- |
|  | | | INT_faculty | | | |
|  |  |  | 1 | 2 | 3 | 4 |
| Cat_prelim | categorical | Count | 21 | 10 | 56 | 64 |
|  |  | % within Cat_prelim | 13.0% | 6.2% | 34.8% | 39.8% |
|  |  | % of Total | 9.8% | 4.7% | 26.0% | 29.8% |
|  | preliminary | Count | 6 | 7 | 15 | 22 |
|  |  | % within Cat_prelim | 11.1% | 13.0% | 27.8% | 40.7% |
|  |  | % of Total | 2.8% | 3.3% | 7.0% | 10.2% |
| Total | | Count | 27 | 17 | 71 | 86 |
|  |  | % within Cat_prelim | 12.6% | 7.9% | 33.0% | 40.0% |
|  |  | % of Total | 12.6% | 7.9% | 33.0% | 40.0% |

| **Crosstab** | | | | |
| --- | --- | --- | --- | --- |
|  | | | INT_faculty | Total |
|  |  |  | 5 |  |
| Cat_prelim | categorical | Count | 10 | 161 |
|  |  | % within Cat_prelim | 6.2% | 100.0% |
|  |  | % of Total | 4.7% | 74.9% |
|  | preliminary | Count | 4 | 54 |
|  |  | % within Cat_prelim | 7.4% | 100.0% |
|  |  | % of Total | 1.9% | 25.1% |
| Total | | Count | 14 | 215 |
|  |  | % within Cat_prelim | 6.5% | 100.0% |
|  |  | % of Total | 6.5% | 100.0% |

| **Chi-Square Tests** | | | |
| --- | --- | --- | --- |
|  | Value | df | Asymp. Sig. (2-sided) |
| Pearson Chi-Square | 3.151^a^ | 4 | .533 |
| Likelihood Ratio | 2.937 | 4 | .568 |
| Linear-by-Linear Association | .001 | 1 | .977 |
| N of Valid Cases | 215 |  |  |

| a. 2 cells (20.0%) have expected count less than 5. The minimum expected count is 3.52. |
| --- |

**Cat_prelim * INT_APD**

| **Crosstab** | | | | | | |
| --- | --- | --- | --- | --- | --- | --- |
|  | | | INT_APD | | | |
|  |  |  | 1 | 2 | 3 | 4 |
| Cat_prelim | categorical | Count | 13 | 88 | 48 | 11 |
|  |  | % within Cat_prelim | 8.1% | 54.7% | 29.8% | 6.8% |
|  |  | % of Total | 6.0% | 40.9% | 22.3% | 5.1% |
|  | preliminary | Count | 5 | 32 | 12 | 5 |
|  |  | % within Cat_prelim | 9.3% | 59.3% | 22.2% | 9.3% |
|  |  | % of Total | 2.3% | 14.9% | 5.6% | 2.3% |
| Total | | Count | 18 | 120 | 60 | 16 |
|  |  | % within Cat_prelim | 8.4% | 55.8% | 27.9% | 7.4% |
|  |  | % of Total | 8.4% | 55.8% | 27.9% | 7.4% |

| **Crosstab** | | | | |
| --- | --- | --- | --- | --- |
|  | | | INT_APD | Total |
|  |  |  | 5 |  |
| Cat_prelim | categorical | Count | 1 | 161 |
|  |  | % within Cat_prelim | 0.6% | 100.0% |
|  |  | % of Total | 0.5% | 74.9% |
|  | preliminary | Count | 0 | 54 |
|  |  | % within Cat_prelim | 0.0% | 100.0% |
|  |  | % of Total | 0.0% | 25.1% |
| Total | | Count | 1 | 215 |
|  |  | % within Cat_prelim | 0.5% | 100.0% |
|  |  | % of Total | 0.5% | 100.0% |

| **Chi-Square Tests** | | | |
| --- | --- | --- | --- |
|  | Value | df | Asymp. Sig. (2-sided) |
| Pearson Chi-Square | 1.712^a^ | 4 | .789 |
| Likelihood Ratio | 1.979 | 4 | .740 |
| Linear-by-Linear Association | .235 | 1 | .628 |
| N of Valid Cases | 215 |  |  |

| a. 4 cells (40.0%) have expected count less than 5. The minimum expected count is .25. |
| --- |

**Cat_prelim * INT_PD**

| **Crosstab** | | | | | | |
| --- | --- | --- | --- | --- | --- | --- |
|  | | | INT_PD | | | |
|  |  |  | 1 | 2 | 3 | 4 |
| Cat_prelim | categorical | Count | 117 | 23 | 12 | 5 |
|  |  | % within Cat_prelim | 72.7% | 14.3% | 7.5% | 3.1% |
|  |  | % of Total | 54.4% | 10.7% | 5.6% | 2.3% |
|  | preliminary | Count | 39 | 7 | 7 | 1 |
|  |  | % within Cat_prelim | 72.2% | 13.0% | 13.0% | 1.9% |
|  |  | % of Total | 18.1% | 3.3% | 3.3% | 0.5% |
| Total | | Count | 156 | 30 | 19 | 6 |
|  |  | % within Cat_prelim | 72.6% | 14.0% | 8.8% | 2.8% |
|  |  | % of Total | 72.6% | 14.0% | 8.8% | 2.8% |

| **Crosstab** | | | | |
| --- | --- | --- | --- | --- |
|  | | | INT_PD | Total |
|  |  |  | 5 |  |
| Cat_prelim | categorical | Count | 4 | 161 |
|  |  | % within Cat_prelim | 2.5% | 100.0% |
|  |  | % of Total | 1.9% | 74.9% |
|  | preliminary | Count | 0 | 54 |
|  |  | % within Cat_prelim | 0.0% | 100.0% |
|  |  | % of Total | 0.0% | 25.1% |
| Total | | Count | 4 | 215 |
|  |  | % within Cat_prelim | 1.9% | 100.0% |
|  |  | % of Total | 1.9% | 100.0% |

| **Chi-Square Tests** | | | |
| --- | --- | --- | --- |
|  | Value | df | Asymp. Sig. (2-sided) |
| Pearson Chi-Square | 3.010^a^ | 4 | .556 |
| Likelihood Ratio | 3.892 | 4 | .421 |
| Linear-by-Linear Association | .079 | 1 | .779 |
| N of Valid Cases | 215 |  |  |

| a. 5 cells (50.0%) have expected count less than 5. The minimum expected count is 1.00. |
| --- |

**Cat_prelim * INT_chair**

| **Crosstab** | | | | | | |
| --- | --- | --- | --- | --- | --- | --- |
|  | | | INT_chair | | | |
|  |  |  | 1 | 2 | 3 | 4 |
| Cat_prelim | categorical | Count | 5 | 22 | 24 | 27 |
|  |  | % within Cat_prelim | 3.1% | 13.7% | 14.9% | 16.8% |
|  |  | % of Total | 2.3% | 10.2% | 11.2% | 12.6% |
|  | preliminary | Count | 2 | 5 | 8 | 9 |
|  |  | % within Cat_prelim | 3.7% | 9.3% | 14.8% | 16.7% |
|  |  | % of Total | 0.9% | 2.3% | 3.7% | 4.2% |
| Total | | Count | 7 | 27 | 32 | 36 |
|  |  | % within Cat_prelim | 3.3% | 12.6% | 14.9% | 16.7% |
|  |  | % of Total | 3.3% | 12.6% | 14.9% | 16.7% |

| **Crosstab** | | | | |
| --- | --- | --- | --- | --- |
|  | | | INT_chair | Total |
|  |  |  | 5 |  |
| Cat_prelim | categorical | Count | 83 | 161 |
|  |  | % within Cat_prelim | 51.6% | 100.0% |
|  |  | % of Total | 38.6% | 74.9% |
|  | preliminary | Count | 30 | 54 |
|  |  | % within Cat_prelim | 55.6% | 100.0% |
|  |  | % of Total | 14.0% | 25.1% |
| Total | | Count | 113 | 215 |
|  |  | % within Cat_prelim | 52.6% | 100.0% |
|  |  | % of Total | 52.6% | 100.0% |

| **Chi-Square Tests** | | | |
| --- | --- | --- | --- |
|  | Value | df | Asymp. Sig. (2-sided) |
| Pearson Chi-Square | .793^a^ | 4 | .939 |
| Likelihood Ratio | .833 | 4 | .934 |
| Linear-by-Linear Association | .338 | 1 | .561 |
| N of Valid Cases | 215 |  |  |

| a. 1 cells (10.0%) have expected count less than 5. The minimum expected count is 1.76. |
| --- |

**Cat_prelim * Qstraightforward**

| **Crosstab** | | | | | | |
| --- | --- | --- | --- | --- | --- | --- |
|  | | | Qstraightforward | | | |
|  |  |  | 1 | 2 | 3 | 4 |
| Cat_prelim | categorical | Count | 116 | 19 | 12 | 11 |
|  |  | % within Cat_prelim | 72.0% | 11.8% | 7.5% | 6.8% |
|  |  | % of Total | 54.0% | 8.8% | 5.6% | 5.1% |
|  | preliminary | Count | 52 | 1 | 0 | 1 |
|  |  | % within Cat_prelim | 96.3% | 1.9% | 0.0% | 1.9% |
|  |  | % of Total | 24.2% | 0.5% | 0.0% | 0.5% |
| Total | | Count | 168 | 20 | 12 | 12 |
|  |  | % within Cat_prelim | 78.1% | 9.3% | 5.6% | 5.6% |
|  |  | % of Total | 78.1% | 9.3% | 5.6% | 5.6% |

| **Crosstab** | | | | |
| --- | --- | --- | --- | --- |
|  | | | Qstraightforward | Total |
|  |  |  | 5 |  |
| Cat_prelim | categorical | Count | 3 | 161 |
|  |  | % within Cat_prelim | 1.9% | 100.0% |
|  |  | % of Total | 1.4% | 74.9% |
|  | preliminary | Count | 0 | 54 |
|  |  | % within Cat_prelim | 0.0% | 100.0% |
|  |  | % of Total | 0.0% | 25.1% |
| Total | | Count | 3 | 215 |
|  |  | % within Cat_prelim | 1.4% | 100.0% |
|  |  | % of Total | 1.4% | 100.0% |

| **Chi-Square Tests** | | | |
| --- | --- | --- | --- |
|  | Value | df | Asymp. Sig. (2-sided) |
| Pearson Chi-Square | 14.174^a^ | 4 | .007 |
| Likelihood Ratio | 19.638 | 4 | .001 |
| Linear-by-Linear Association | 10.464 | 1 | .001 |
| N of Valid Cases | 215 |  |  |

| a. 4 cells (40.0%) have expected count less than 5. The minimum expected count is .75. |
| --- |

**Cat_prelim * Qbehavior**

| **Crosstab** | | | | | | |
| --- | --- | --- | --- | --- | --- | --- |
|  | | | Qbehavior | | | |
|  |  |  | 1 | 2 | 3 | 4 |
| Cat_prelim | categorical | Count | 27 | 78 | 49 | 5 |
|  |  | % within Cat_prelim | 16.8% | 48.4% | 30.4% | 3.1% |
|  |  | % of Total | 12.6% | 36.3% | 22.8% | 2.3% |
|  | preliminary | Count | 2 | 32 | 15 | 3 |
|  |  | % within Cat_prelim | 3.7% | 59.3% | 27.8% | 5.6% |
|  |  | % of Total | 0.9% | 14.9% | 7.0% | 1.4% |
| Total | | Count | 29 | 110 | 64 | 8 |
|  |  | % within Cat_prelim | 13.5% | 51.2% | 29.8% | 3.7% |
|  |  | % of Total | 13.5% | 51.2% | 29.8% | 3.7% |

| **Crosstab** | | | | |
| --- | --- | --- | --- | --- |
|  | | | Qbehavior | Total |
|  |  |  | 5 |  |
| Cat_prelim | categorical | Count | 2 | 161 |
|  |  | % within Cat_prelim | 1.2% | 100.0% |
|  |  | % of Total | 0.9% | 74.9% |
|  | preliminary | Count | 2 | 54 |
|  |  | % within Cat_prelim | 3.7% | 100.0% |
|  |  | % of Total | 0.9% | 25.1% |
| Total | | Count | 4 | 215 |
|  |  | % within Cat_prelim | 1.9% | 100.0% |
|  |  | % of Total | 1.9% | 100.0% |

| **Chi-Square Tests** | | | |
| --- | --- | --- | --- |
|  | Value | df | Asymp. Sig. (2-sided) |
| Pearson Chi-Square | 8.107^a^ | 4 | .088 |
| Likelihood Ratio | 9.317 | 4 | .054 |
| Linear-by-Linear Association | 3.127 | 1 | .077 |
| N of Valid Cases | 215 |  |  |

| a. 3 cells (30.0%) have expected count less than 5. The minimum expected count is 1.00. |
| --- |

**Cat_prelim * Qsituation**

| **Crosstab** | | | | | | |
| --- | --- | --- | --- | --- | --- | --- |
|  | | | Qsituation | | | |
|  |  |  | 1 | 2 | 3 | 4 |
| Cat_prelim | categorical | Count | 11 | 55 | 78 | 13 |
|  |  | % within Cat_prelim | 6.8% | 34.2% | 48.4% | 8.1% |
|  |  | % of Total | 5.1% | 25.6% | 36.3% | 6.0% |
|  | preliminary | Count | 0 | 15 | 28 | 9 |
|  |  | % within Cat_prelim | 0.0% | 27.8% | 51.9% | 16.7% |
|  |  | % of Total | 0.0% | 7.0% | 13.0% | 4.2% |
| Total | | Count | 11 | 70 | 106 | 22 |
|  |  | % within Cat_prelim | 5.1% | 32.6% | 49.3% | 10.2% |
|  |  | % of Total | 5.1% | 32.6% | 49.3% | 10.2% |

| **Crosstab** | | | | |
| --- | --- | --- | --- | --- |
|  | | | Qsituation | Total |
|  |  |  | 5 |  |
| Cat_prelim | categorical | Count | 4 | 161 |
|  |  | % within Cat_prelim | 2.5% | 100.0% |
|  |  | % of Total | 1.9% | 74.9% |
|  | preliminary | Count | 2 | 54 |
|  |  | % within Cat_prelim | 3.7% | 100.0% |
|  |  | % of Total | 0.9% | 25.1% |
| Total | | Count | 6 | 215 |
|  |  | % within Cat_prelim | 2.8% | 100.0% |
|  |  | % of Total | 2.8% | 100.0% |

| **Chi-Square Tests** | | | |
| --- | --- | --- | --- |
|  | Value | df | Asymp. Sig. (2-sided) |
| Pearson Chi-Square | 7.423^a^ | 4 | .115 |
| Likelihood Ratio | 9.806 | 4 | .044 |
| Linear-by-Linear Association | 5.790 | 1 | .016 |
| N of Valid Cases | 215 |  |  |

| a. 3 cells (30.0%) have expected count less than 5. The minimum expected count is 1.51. |
| --- |

**Cat_prelim * Qteaser**

| **Crosstab** | | | | | | |
| --- | --- | --- | --- | --- | --- | --- |
|  | | | Qteaser | | | |
|  |  |  | 1 | 2 | 3 | 4 |
| Cat_prelim | categorical | Count | 4 | 9 | 15 | 96 |
|  |  | % within Cat_prelim | 2.5% | 5.6% | 9.3% | 59.6% |
|  |  | % of Total | 1.9% | 4.2% | 7.0% | 44.7% |
|  | preliminary | Count | 0 | 6 | 8 | 28 |
|  |  | % within Cat_prelim | 0.0% | 11.1% | 14.8% | 51.9% |
|  |  | % of Total | 0.0% | 2.8% | 3.7% | 13.0% |
| Total | | Count | 4 | 15 | 23 | 124 |
|  |  | % within Cat_prelim | 1.9% | 7.0% | 10.7% | 57.7% |
|  |  | % of Total | 1.9% | 7.0% | 10.7% | 57.7% |

| **Crosstab** | | | | |
| --- | --- | --- | --- | --- |
|  | | | Qteaser | Total |
|  |  |  | 5 |  |
| Cat_prelim | categorical | Count | 37 | 161 |
|  |  | % within Cat_prelim | 23.0% | 100.0% |
|  |  | % of Total | 17.2% | 74.9% |
|  | preliminary | Count | 12 | 54 |
|  |  | % within Cat_prelim | 22.2% | 100.0% |
|  |  | % of Total | 5.6% | 25.1% |
| Total | | Count | 49 | 215 |
|  |  | % within Cat_prelim | 22.8% | 100.0% |
|  |  | % of Total | 22.8% | 100.0% |

| **Chi-Square Tests** | | | |
| --- | --- | --- | --- |
|  | Value | df | Asymp. Sig. (2-sided) |
| Pearson Chi-Square | 4.685^a^ | 4 | .321 |
| Likelihood Ratio | 5.417 | 4 | .247 |
| Linear-by-Linear Association | .503 | 1 | .478 |
| N of Valid Cases | 215 |  |  |

| a. 3 cells (30.0%) have expected count less than 5. The minimum expected count is 1.00. |
| --- |

**Cat_prelim * Qmedical**

| **Crosstab** | | | | | | |
| --- | --- | --- | --- | --- | --- | --- |
|  | | | Qmedical | | | |
|  |  |  | 1 | 3 | 4 | 5 |
| Cat_prelim | categorical | Count | 3 | 7 | 36 | 115 |
|  |  | % within Cat_prelim | 1.9% | 4.3% | 22.4% | 71.4% |
|  |  | % of Total | 1.4% | 3.3% | 16.7% | 53.5% |
|  | preliminary | Count | 0 | 3 | 13 | 38 |
|  |  | % within Cat_prelim | 0.0% | 5.6% | 24.1% | 70.4% |
|  |  | % of Total | 0.0% | 1.4% | 6.0% | 17.7% |
| Total | | Count | 3 | 10 | 49 | 153 |
|  |  | % within Cat_prelim | 1.4% | 4.7% | 22.8% | 71.2% |
|  |  | % of Total | 1.4% | 4.7% | 22.8% | 71.2% |

| **Crosstab** | | | |
| --- | --- | --- | --- |
|  | | | Total |
|  |  |  |  |
| Cat_prelim | categorical | Count | 161 |
|  |  | % within Cat_prelim | 100.0% |
|  |  | % of Total | 74.9% |
|  | preliminary | Count | 54 |
|  |  | % within Cat_prelim | 100.0% |
|  |  | % of Total | 25.1% |
| Total | | Count | 215 |
|  |  | % within Cat_prelim | 100.0% |
|  |  | % of Total | 100.0% |

| **Chi-Square Tests** | | | |
| --- | --- | --- | --- |
|  | Value | df | Asymp. Sig. (2-sided) |
| Pearson Chi-Square | 1.192^a^ | 3 | .755 |
| Likelihood Ratio | 1.915 | 3 | .590 |
| Linear-by-Linear Association | .090 | 1 | .764 |
| N of Valid Cases | 215 |  |  |

| a. 3 cells (37.5%) have expected count less than 5. The minimum expected count is .75. |
| --- |

**Cat_prelim * ResIA_LT15**

| **Crosstab** | | | | | | |
| --- | --- | --- | --- | --- | --- | --- |
|  | | | ResIA_LT15 | | | |
|  |  |  | 1 | 2 | 3 | 4 |
| Cat_prelim | categorical | Count | 3 | 6 | 12 | 24 |
|  |  | % within Cat_prelim | 1.9% | 3.7% | 7.5% | 14.9% |
|  |  | % of Total | 1.4% | 2.8% | 5.6% | 11.2% |
|  | preliminary | Count | 1 | 3 | 6 | 11 |
|  |  | % within Cat_prelim | 1.9% | 5.6% | 11.1% | 20.4% |
|  |  | % of Total | 0.5% | 1.4% | 2.8% | 5.1% |
| Total | | Count | 4 | 9 | 18 | 35 |
|  |  | % within Cat_prelim | 1.9% | 4.2% | 8.4% | 16.3% |
|  |  | % of Total | 1.9% | 4.2% | 8.4% | 16.3% |

| **Crosstab** | | | | |
| --- | --- | --- | --- | --- |
|  | | | ResIA_LT15 | Total |
|  |  |  | 5 |  |
| Cat_prelim | categorical | Count | 116 | 161 |
|  |  | % within Cat_prelim | 72.0% | 100.0% |
|  |  | % of Total | 54.0% | 74.9% |
|  | preliminary | Count | 33 | 54 |
|  |  | % within Cat_prelim | 61.1% | 100.0% |
|  |  | % of Total | 15.3% | 25.1% |
| Total | | Count | 149 | 215 |
|  |  | % within Cat_prelim | 69.3% | 100.0% |
|  |  | % of Total | 69.3% | 100.0% |

| **Chi-Square Tests** | | | |
| --- | --- | --- | --- |
|  | Value | df | Asymp. Sig. (2-sided) |
| Pearson Chi-Square | 2.409^a^ | 4 | .661 |
| Likelihood Ratio | 2.334 | 4 | .675 |
| Linear-by-Linear Association | 1.500 | 1 | .221 |
| N of Valid Cases | 215 |  |  |

| a. 4 cells (40.0%) have expected count less than 5. The minimum expected count is 1.00. |
| --- |

**Cat_prelim * ResIA15_30**

| **Crosstab** | | | | | | |
| --- | --- | --- | --- | --- | --- | --- |
|  | | | ResIA15_30 | | | |
|  |  |  | 1 | 2 | 3 | 4 |
| Cat_prelim | categorical | Count | 21 | 28 | 20 | 90 |
|  |  | % within Cat_prelim | 13.0% | 17.4% | 12.4% | 55.9% |
|  |  | % of Total | 9.8% | 13.0% | 9.3% | 41.9% |
|  | preliminary | Count | 6 | 15 | 12 | 21 |
|  |  | % within Cat_prelim | 11.1% | 27.8% | 22.2% | 38.9% |
|  |  | % of Total | 2.8% | 7.0% | 5.6% | 9.8% |
| Total | | Count | 27 | 43 | 32 | 111 |
|  |  | % within Cat_prelim | 12.6% | 20.0% | 14.9% | 51.6% |
|  |  | % of Total | 12.6% | 20.0% | 14.9% | 51.6% |

| **Crosstab** | | | | |
| --- | --- | --- | --- | --- |
|  | | | ResIA15_30 | Total |
|  |  |  | 5 |  |
| Cat_prelim | categorical | Count | 2 | 161 |
|  |  | % within Cat_prelim | 1.2% | 100.0% |
|  |  | % of Total | 0.9% | 74.9% |
|  | preliminary | Count | 0 | 54 |
|  |  | % within Cat_prelim | 0.0% | 100.0% |
|  |  | % of Total | 0.0% | 25.1% |
| Total | | Count | 2 | 215 |
|  |  | % within Cat_prelim | 0.9% | 100.0% |
|  |  | % of Total | 0.9% | 100.0% |

| **Chi-Square Tests** | | | |
| --- | --- | --- | --- |
|  | Value | df | Asymp. Sig. (2-sided) |
| Pearson Chi-Square | 7.848^a^ | 4 | .097 |
| Likelihood Ratio | 8.109 | 4 | .088 |
| Linear-by-Linear Association | 2.182 | 1 | .140 |
| N of Valid Cases | 215 |  |  |

| a. 2 cells (20.0%) have expected count less than 5. The minimum expected count is .50. |
| --- |

**Cat_prelim * ResIA30_45**

| **Crosstab** | | | | | | |
| --- | --- | --- | --- | --- | --- | --- |
|  | | | ResIA30_45 | | | Total |
|  |  |  | 1 | 2 | 3 |  |
| Cat_prelim | categorical | Count | 54 | 43 | 64 | 161 |
|  |  | % within Cat_prelim | 33.5% | 26.7% | 39.8% | 100.0% |
|  |  | % of Total | 25.1% | 20.0% | 29.8% | 74.9% |
|  | preliminary | Count | 30 | 10 | 14 | 54 |
|  |  | % within Cat_prelim | 55.6% | 18.5% | 25.9% | 100.0% |
|  |  | % of Total | 14.0% | 4.7% | 6.5% | 25.1% |
| Total | | Count | 84 | 53 | 78 | 215 |
|  |  | % within Cat_prelim | 39.1% | 24.7% | 36.3% | 100.0% |
|  |  | % of Total | 39.1% | 24.7% | 36.3% | 100.0% |

| **Chi-Square Tests** | | | |
| --- | --- | --- | --- |
|  | Value | df | Asymp. Sig. (2-sided) |
| Pearson Chi-Square | 8.247^a^ | 2 | .016 |
| Likelihood Ratio | 8.105 | 2 | .017 |
| Linear-by-Linear Association | 6.869 | 1 | .009 |
| N of Valid Cases | 215 |  |  |

| a. 0 cells (0.0%) have expected count less than 5. The minimum expected count is 13.31. |
| --- |

**Cat_prelim * ResIA45_60**

| **Crosstab** | | | | | | |
| --- | --- | --- | --- | --- | --- | --- |
|  | | | ResIA45_60 | | | |
|  |  |  | 1 | 2 | 3 | 4 |
| Cat_prelim | categorical | Count | 54 | 60 | 26 | 19 |
|  |  | % within Cat_prelim | 33.5% | 37.3% | 16.1% | 11.8% |
|  |  | % of Total | 25.1% | 27.9% | 12.1% | 8.8% |
|  | preliminary | Count | 11 | 22 | 11 | 10 |
|  |  | % within Cat_prelim | 20.4% | 40.7% | 20.4% | 18.5% |
|  |  | % of Total | 5.1% | 10.2% | 5.1% | 4.7% |
| Total | | Count | 65 | 82 | 37 | 29 |
|  |  | % within Cat_prelim | 30.2% | 38.1% | 17.2% | 13.5% |
|  |  | % of Total | 30.2% | 38.1% | 17.2% | 13.5% |

| **Crosstab** | | | | |
| --- | --- | --- | --- | --- |
|  | | | ResIA45_60 | Total |
|  |  |  | 5 |  |
| Cat_prelim | categorical | Count | 2 | 161 |
|  |  | % within Cat_prelim | 1.2% | 100.0% |
|  |  | % of Total | 0.9% | 74.9% |
|  | preliminary | Count | 0 | 54 |
|  |  | % within Cat_prelim | 0.0% | 100.0% |
|  |  | % of Total | 0.0% | 25.1% |
| Total | | Count | 2 | 215 |
|  |  | % within Cat_prelim | 0.9% | 100.0% |
|  |  | % of Total | 0.9% | 100.0% |

| **Chi-Square Tests** | | | |
| --- | --- | --- | --- |
|  | Value | df | Asymp. Sig. (2-sided) |
| Pearson Chi-Square | 4.890^a^ | 4 | .299 |
| Likelihood Ratio | 5.475 | 4 | .242 |
| Linear-by-Linear Association | 2.763 | 1 | .096 |
| N of Valid Cases | 215 |  |  |

| a. 2 cells (20.0%) have expected count less than 5. The minimum expected count is .50. |
| --- |

**Cat_prelim * ResIA_GT60**

| **Crosstab** | | | | | | |
| --- | --- | --- | --- | --- | --- | --- |
|  | | | ResIA_GT60 | | | |
|  |  |  | 1 | 2 | 3 | 4 |
| Cat_prelim | categorical | Count | 29 | 23 | 39 | 28 |
|  |  | % within Cat_prelim | 18.0% | 14.3% | 24.2% | 17.4% |
|  |  | % of Total | 13.5% | 10.7% | 18.1% | 13.0% |
|  | preliminary | Count | 6 | 4 | 11 | 12 |
|  |  | % within Cat_prelim | 11.1% | 7.4% | 20.4% | 22.2% |
|  |  | % of Total | 2.8% | 1.9% | 5.1% | 5.6% |
| Total | | Count | 35 | 27 | 50 | 40 |
|  |  | % within Cat_prelim | 16.3% | 12.6% | 23.3% | 18.6% |
|  |  | % of Total | 16.3% | 12.6% | 23.3% | 18.6% |

| **Crosstab** | | | | |
| --- | --- | --- | --- | --- |
|  | | | ResIA_GT60 | Total |
|  |  |  | 5 |  |
| Cat_prelim | categorical | Count | 42 | 161 |
|  |  | % within Cat_prelim | 26.1% | 100.0% |
|  |  | % of Total | 19.5% | 74.9% |
|  | preliminary | Count | 21 | 54 |
|  |  | % within Cat_prelim | 38.9% | 100.0% |
|  |  | % of Total | 9.8% | 25.1% |
| Total | | Count | 63 | 215 |
|  |  | % within Cat_prelim | 29.3% | 100.0% |
|  |  | % of Total | 29.3% | 100.0% |

| **Chi-Square Tests** | | | |
| --- | --- | --- | --- |
|  | Value | df | Asymp. Sig. (2-sided) |
| Pearson Chi-Square | 5.734^a^ | 4 | .220 |
| Likelihood Ratio | 5.869 | 4 | .209 |
| Linear-by-Linear Association | 5.176 | 1 | .023 |
| N of Valid Cases | 215 |  |  |

| a. 0 cells (0.0%) have expected count less than 5. The minimum expected count is 6.78. |
| --- |

**Cat_prelim * october**

| **Crosstab** | | | | | | |
| --- | --- | --- | --- | --- | --- | --- |
|  | | | october | | | |
|  |  |  | 1 | 2 | 3 | 4 |
| Cat_prelim | categorical | Count | 19 | 32 | 39 | 52 |
|  |  | % within Cat_prelim | 11.8% | 19.9% | 24.2% | 32.3% |
|  |  | % of Total | 8.8% | 14.9% | 18.1% | 24.2% |
|  | preliminary | Count | 14 | 10 | 13 | 14 |
|  |  | % within Cat_prelim | 25.9% | 18.5% | 24.1% | 25.9% |
|  |  | % of Total | 6.5% | 4.7% | 6.0% | 6.5% |
| Total | | Count | 33 | 42 | 52 | 66 |
|  |  | % within Cat_prelim | 15.3% | 19.5% | 24.2% | 30.7% |
|  |  | % of Total | 15.3% | 19.5% | 24.2% | 30.7% |

| **Crosstab** | | | | |
| --- | --- | --- | --- | --- |
|  | | | october | Total |
|  |  |  | 5 |  |
| Cat_prelim | categorical | Count | 19 | 161 |
|  |  | % within Cat_prelim | 11.8% | 100.0% |
|  |  | % of Total | 8.8% | 74.9% |
|  | preliminary | Count | 3 | 54 |
|  |  | % within Cat_prelim | 5.6% | 100.0% |
|  |  | % of Total | 1.4% | 25.1% |
| Total | | Count | 22 | 215 |
|  |  | % within Cat_prelim | 10.2% | 100.0% |
|  |  | % of Total | 10.2% | 100.0% |

| **Chi-Square Tests** | | | |
| --- | --- | --- | --- |
|  | Value | df | Asymp. Sig. (2-sided) |
| Pearson Chi-Square | 7.371^a^ | 4 | .118 |
| Likelihood Ratio | 7.039 | 4 | .134 |
| Linear-by-Linear Association | 5.524 | 1 | .019 |
| N of Valid Cases | 215 |  |  |

| a. 0 cells (0.0%) have expected count less than 5. The minimum expected count is 5.53. |
| --- |

**Cat_prelim * november**

| **Crosstab** | | | | | | |
| --- | --- | --- | --- | --- | --- | --- |
|  | | | november | | | |
|  |  |  | 1 | 2 | 3 | 4 |
| Cat_prelim | categorical | Count | 108 | 39 | 9 | 4 |
|  |  | % within Cat_prelim | 67.1% | 24.2% | 5.6% | 2.5% |
|  |  | % of Total | 50.2% | 18.1% | 4.2% | 1.9% |
|  | preliminary | Count | 20 | 21 | 7 | 6 |
|  |  | % within Cat_prelim | 37.0% | 38.9% | 13.0% | 11.1% |
|  |  | % of Total | 9.3% | 9.8% | 3.3% | 2.8% |
| Total | | Count | 128 | 60 | 16 | 10 |
|  |  | % within Cat_prelim | 59.5% | 27.9% | 7.4% | 4.7% |
|  |  | % of Total | 59.5% | 27.9% | 7.4% | 4.7% |

| **Crosstab** | | | | |
| --- | --- | --- | --- | --- |
|  | | | november | Total |
|  |  |  | 5 |  |
| Cat_prelim | categorical | Count | 1 | 161 |
|  |  | % within Cat_prelim | 0.6% | 100.0% |
|  |  | % of Total | 0.5% | 74.9% |
|  | preliminary | Count | 0 | 54 |
|  |  | % within Cat_prelim | 0.0% | 100.0% |
|  |  | % of Total | 0.0% | 25.1% |
| Total | | Count | 1 | 215 |
|  |  | % within Cat_prelim | 0.5% | 100.0% |
|  |  | % of Total | 0.5% | 100.0% |

| **Chi-Square Tests** | | | |
| --- | --- | --- | --- |
|  | Value | df | Asymp. Sig. (2-sided) |
| Pearson Chi-Square | 19.006^a^ | 4 | .001 |
| Likelihood Ratio | 18.318 | 4 | .001 |
| Linear-by-Linear Association | 15.453 | 1 | .000 |
| N of Valid Cases | 215 |  |  |

| a. 4 cells (40.0%) have expected count less than 5. The minimum expected count is .25. |
| --- |

**Cat_prelim * december**

| **Crosstab** | | | | | | |
| --- | --- | --- | --- | --- | --- | --- |
|  | | | december | | | |
|  |  |  | 1 | 2 | 3 | 4 |
| Cat_prelim | categorical | Count | 29 | 78 | 51 | 1 |
|  |  | % within Cat_prelim | 18.0% | 48.4% | 31.7% | 0.6% |
|  |  | % of Total | 13.5% | 36.3% | 23.7% | 0.5% |
|  | preliminary | Count | 15 | 14 | 20 | 2 |
|  |  | % within Cat_prelim | 27.8% | 25.9% | 37.0% | 3.7% |
|  |  | % of Total | 7.0% | 6.5% | 9.3% | 0.9% |
| Total | | Count | 44 | 92 | 71 | 3 |
|  |  | % within Cat_prelim | 20.5% | 42.8% | 33.0% | 1.4% |
|  |  | % of Total | 20.5% | 42.8% | 33.0% | 1.4% |

| **Crosstab** | | | | |
| --- | --- | --- | --- | --- |
|  | | | december | Total |
|  |  |  | 5 |  |
| Cat_prelim | categorical | Count | 2 | 161 |
|  |  | % within Cat_prelim | 1.2% | 100.0% |
|  |  | % of Total | 0.9% | 74.9% |
|  | preliminary | Count | 3 | 54 |
|  |  | % within Cat_prelim | 5.6% | 100.0% |
|  |  | % of Total | 1.4% | 25.1% |
| Total | | Count | 5 | 215 |
|  |  | % within Cat_prelim | 2.3% | 100.0% |
|  |  | % of Total | 2.3% | 100.0% |

| **Chi-Square Tests** | | | |
| --- | --- | --- | --- |
|  | Value | df | Asymp. Sig. (2-sided) |
| Pearson Chi-Square | 13.018^a^ | 4 | .011 |
| Likelihood Ratio | 12.445 | 4 | .014 |
| Linear-by-Linear Association | 1.159 | 1 | .282 |
| N of Valid Cases | 215 |  |  |

| a. 4 cells (40.0%) have expected count less than 5. The minimum expected count is .75. |
| --- |

**Cat_prelim * january**

| **Crosstab** | | | | | | |
| --- | --- | --- | --- | --- | --- | --- |
|  | | | january | | | |
|  |  |  | 1 | 2 | 3 | 4 |
| Cat_prelim | categorical | Count | 2 | 12 | 60 | 86 |
|  |  | % within Cat_prelim | 1.2% | 7.5% | 37.3% | 53.4% |
|  |  | % of Total | 0.9% | 5.6% | 27.9% | 40.0% |
|  | preliminary | Count | 5 | 6 | 12 | 30 |
|  |  | % within Cat_prelim | 9.3% | 11.1% | 22.2% | 55.6% |
|  |  | % of Total | 2.3% | 2.8% | 5.6% | 14.0% |
| Total | | Count | 7 | 18 | 72 | 116 |
|  |  | % within Cat_prelim | 3.3% | 8.4% | 33.5% | 54.0% |
|  |  | % of Total | 3.3% | 8.4% | 33.5% | 54.0% |

| **Crosstab** | | | | |
| --- | --- | --- | --- | --- |
|  | | | january | Total |
|  |  |  | 5 |  |
| Cat_prelim | categorical | Count | 1 | 161 |
|  |  | % within Cat_prelim | 0.6% | 100.0% |
|  |  | % of Total | 0.5% | 74.9% |
|  | preliminary | Count | 1 | 54 |
|  |  | % within Cat_prelim | 1.9% | 100.0% |
|  |  | % of Total | 0.5% | 25.1% |
| Total | | Count | 2 | 215 |
|  |  | % within Cat_prelim | 0.9% | 100.0% |
|  |  | % of Total | 0.9% | 100.0% |

| **Chi-Square Tests** | | | |
| --- | --- | --- | --- |
|  | Value | df | Asymp. Sig. (2-sided) |
| Pearson Chi-Square | 12.055^a^ | 4 | .017 |
| Likelihood Ratio | 10.795 | 4 | .029 |
| Linear-by-Linear Association | 1.471 | 1 | .225 |
| N of Valid Cases | 215 |  |  |

| a. 4 cells (40.0%) have expected count less than 5. The minimum expected count is .50. |
| --- |

**Cat_prelim * february**

| **Crosstab** | | | | | | |
| --- | --- | --- | --- | --- | --- | --- |
|  | | | february | | | |
|  |  |  | 1 | 2 | 3 | 4 |
| Cat_prelim | categorical | Count | 3 | 0 | 2 | 18 |
|  |  | % within Cat_prelim | 1.9% | 0.0% | 1.2% | 11.2% |
|  |  | % of Total | 1.4% | 0.0% | 0.9% | 8.4% |
|  | preliminary | Count | 0 | 3 | 2 | 2 |
|  |  | % within Cat_prelim | 0.0% | 5.6% | 3.7% | 3.7% |
|  |  | % of Total | 0.0% | 1.4% | 0.9% | 0.9% |
| Total | | Count | 3 | 3 | 4 | 20 |
|  |  | % within Cat_prelim | 1.4% | 1.4% | 1.9% | 9.3% |
|  |  | % of Total | 1.4% | 1.4% | 1.9% | 9.3% |

| **Crosstab** | | | | |
| --- | --- | --- | --- | --- |
|  | | | february | Total |
|  |  |  | 5 |  |
| Cat_prelim | categorical | Count | 138 | 161 |
|  |  | % within Cat_prelim | 85.7% | 100.0% |
|  |  | % of Total | 64.2% | 74.9% |
|  | preliminary | Count | 47 | 54 |
|  |  | % within Cat_prelim | 87.0% | 100.0% |
|  |  | % of Total | 21.9% | 25.1% |
| Total | | Count | 185 | 215 |
|  |  | % within Cat_prelim | 86.0% | 100.0% |
|  |  | % of Total | 86.0% | 100.0% |

| **Chi-Square Tests** | | | |
| --- | --- | --- | --- |
|  | Value | df | Asymp. Sig. (2-sided) |
| Pearson Chi-Square | 13.706^a^ | 4 | .008 |
| Likelihood Ratio | 14.108 | 4 | .007 |
| Linear-by-Linear Association | .384 | 1 | .535 |
| N of Valid Cases | 215 |  |  |

| a. 6 cells (60.0%) have expected count less than 5. The minimum expected count is .75. |
| --- |

**Cat_prelim * overview**

| **Crosstab** | | | | | | |
| --- | --- | --- | --- | --- | --- | --- |
|  | | | overview | | | |
|  |  |  | 1 | 2 | 3 | 4 |
| Cat_prelim | categorical | Count | 67 | 59 | 19 | 9 |
|  |  | % within Cat_prelim | 41.6% | 36.6% | 11.8% | 5.6% |
|  |  | % of Total | 31.2% | 27.4% | 8.8% | 4.2% |
|  | preliminary | Count | 22 | 20 | 10 | 1 |
|  |  | % within Cat_prelim | 40.7% | 37.0% | 18.5% | 1.9% |
|  |  | % of Total | 10.2% | 9.3% | 4.7% | 0.5% |
| Total | | Count | 89 | 79 | 29 | 10 |
|  |  | % within Cat_prelim | 41.4% | 36.7% | 13.5% | 4.7% |
|  |  | % of Total | 41.4% | 36.7% | 13.5% | 4.7% |

| **Crosstab** | | | | |
| --- | --- | --- | --- | --- |
|  | | | overview | Total |
|  |  |  | 5 |  |
| Cat_prelim | categorical | Count | 7 | 161 |
|  |  | % within Cat_prelim | 4.3% | 100.0% |
|  |  | % of Total | 3.3% | 74.9% |
|  | preliminary | Count | 1 | 54 |
|  |  | % within Cat_prelim | 1.9% | 100.0% |
|  |  | % of Total | 0.5% | 25.1% |
| Total | | Count | 8 | 215 |
|  |  | % within Cat_prelim | 3.7% | 100.0% |
|  |  | % of Total | 3.7% | 100.0% |

| **Chi-Square Tests** | | | |
| --- | --- | --- | --- |
|  | Value | df | Asymp. Sig. (2-sided) |
| Pearson Chi-Square | 3.254^a^ | 4 | .516 |
| Likelihood Ratio | 3.522 | 4 | .474 |
| Linear-by-Linear Association | .206 | 1 | .650 |
| N of Valid Cases | 215 |  |  |

| a. 2 cells (20.0%) have expected count less than 5. The minimum expected count is 2.01. |
| --- |

**Cat_prelim * interview**

| **Crosstab** | | | | | | |
| --- | --- | --- | --- | --- | --- | --- |
|  | | | interview | | | |
|  |  |  | 1 | 2 | 3 | 4 |
| Cat_prelim | categorical | Count | 69 | 59 | 19 | 10 |
|  |  | % within Cat_prelim | 42.9% | 36.6% | 11.8% | 6.2% |
|  |  | % of Total | 32.1% | 27.4% | 8.8% | 4.7% |
|  | preliminary | Count | 23 | 21 | 8 | 2 |
|  |  | % within Cat_prelim | 42.6% | 38.9% | 14.8% | 3.7% |
|  |  | % of Total | 10.7% | 9.8% | 3.7% | 0.9% |
| Total | | Count | 92 | 80 | 27 | 12 |
|  |  | % within Cat_prelim | 42.8% | 37.2% | 12.6% | 5.6% |
|  |  | % of Total | 42.8% | 37.2% | 12.6% | 5.6% |

| **Crosstab** | | | | |
| --- | --- | --- | --- | --- |
|  | | | interview | Total |
|  |  |  | 5 |  |
| Cat_prelim | categorical | Count | 4 | 161 |
|  |  | % within Cat_prelim | 2.5% | 100.0% |
|  |  | % of Total | 1.9% | 74.9% |
|  | preliminary | Count | 0 | 54 |
|  |  | % within Cat_prelim | 0.0% | 100.0% |
|  |  | % of Total | 0.0% | 25.1% |
| Total | | Count | 4 | 215 |
|  |  | % within Cat_prelim | 1.9% | 100.0% |
|  |  | % of Total | 1.9% | 100.0% |

| **Chi-Square Tests** | | | |
| --- | --- | --- | --- |
|  | Value | df | Asymp. Sig. (2-sided) |
| Pearson Chi-Square | 2.145^a^ | 4 | .709 |
| Likelihood Ratio | 3.148 | 4 | .533 |
| Linear-by-Linear Association | .367 | 1 | .545 |
| N of Valid Cases | 215 |  |  |

| a. 3 cells (30.0%) have expected count less than 5. The minimum expected count is 1.00. |
| --- |

**Cat_prelim * tour**

| **Crosstab** | | | | | | |
| --- | --- | --- | --- | --- | --- | --- |
|  | | | tour | | | |
|  |  |  | 1 | 2 | 3 | 4 |
| Cat_prelim | categorical | Count | 2 | 4 | 35 | 54 |
|  |  | % within Cat_prelim | 1.2% | 2.5% | 21.7% | 33.5% |
|  |  | % of Total | 0.9% | 1.9% | 16.3% | 25.1% |
|  | preliminary | Count | 1 | 0 | 7 | 26 |
|  |  | % within Cat_prelim | 1.9% | 0.0% | 13.0% | 48.1% |
|  |  | % of Total | 0.5% | 0.0% | 3.3% | 12.1% |
| Total | | Count | 3 | 4 | 42 | 80 |
|  |  | % within Cat_prelim | 1.4% | 1.9% | 19.5% | 37.2% |
|  |  | % of Total | 1.4% | 1.9% | 19.5% | 37.2% |

| **Crosstab** | | | | |
| --- | --- | --- | --- | --- |
|  | | | tour | Total |
|  |  |  | 5 |  |
| Cat_prelim | categorical | Count | 66 | 161 |
|  |  | % within Cat_prelim | 41.0% | 100.0% |
|  |  | % of Total | 30.7% | 74.9% |
|  | preliminary | Count | 20 | 54 |
|  |  | % within Cat_prelim | 37.0% | 100.0% |
|  |  | % of Total | 9.3% | 25.1% |
| Total | | Count | 86 | 215 |
|  |  | % within Cat_prelim | 40.0% | 100.0% |
|  |  | % of Total | 40.0% | 100.0% |

| **Chi-Square Tests** | | | |
| --- | --- | --- | --- |
|  | Value | df | Asymp. Sig. (2-sided) |
| Pearson Chi-Square | 5.521^a^ | 4 | .238 |
| Likelihood Ratio | 6.509 | 4 | .164 |
| Linear-by-Linear Association | .327 | 1 | .567 |
| N of Valid Cases | 215 |  |  |

| a. 4 cells (40.0%) have expected count less than 5. The minimum expected count is .75. |
| --- |

**Cat_prelim * morning_report**

| **Crosstab** | | | | | | |
| --- | --- | --- | --- | --- | --- | --- |
|  | | | morning_report | | | |
|  |  |  | 1 | 2 | 3 | 4 |
| Cat_prelim | categorical | Count | 6 | 13 | 29 | 49 |
|  |  | % within Cat_prelim | 3.7% | 8.1% | 18.0% | 30.4% |
|  |  | % of Total | 2.8% | 6.0% | 13.5% | 22.8% |
|  | preliminary | Count | 0 | 1 | 4 | 17 |
|  |  | % within Cat_prelim | 0.0% | 1.9% | 7.4% | 31.5% |
|  |  | % of Total | 0.0% | 0.5% | 1.9% | 7.9% |
| Total | | Count | 6 | 14 | 33 | 66 |
|  |  | % within Cat_prelim | 2.8% | 6.5% | 15.3% | 30.7% |
|  |  | % of Total | 2.8% | 6.5% | 15.3% | 30.7% |

| **Crosstab** | | | | |
| --- | --- | --- | --- | --- |
|  | | | morning_report | Total |
|  |  |  | 5 |  |
| Cat_prelim | categorical | Count | 64 | 161 |
|  |  | % within Cat_prelim | 39.8% | 100.0% |
|  |  | % of Total | 29.8% | 74.9% |
|  | preliminary | Count | 32 | 54 |
|  |  | % within Cat_prelim | 59.3% | 100.0% |
|  |  | % of Total | 14.9% | 25.1% |
| Total | | Count | 96 | 215 |
|  |  | % within Cat_prelim | 44.7% | 100.0% |
|  |  | % of Total | 44.7% | 100.0% |

| **Chi-Square Tests** | | | |
| --- | --- | --- | --- |
|  | Value | df | Asymp. Sig. (2-sided) |
| Pearson Chi-Square | 10.841^a^ | 4 | .028 |
| Likelihood Ratio | 13.253 | 4 | .010 |
| Linear-by-Linear Association | 10.515 | 1 | .001 |
| N of Valid Cases | 215 |  |  |

| a. 3 cells (30.0%) have expected count less than 5. The minimum expected count is 1.51. |
| --- |

**Cat_prelim * lunch**

| **Crosstab** | | | | | | |
| --- | --- | --- | --- | --- | --- | --- |
|  | | | lunch | | | |
|  |  |  | 1 | 2 | 3 | 4 |
| Cat_prelim | categorical | Count | 17 | 26 | 57 | 39 |
|  |  | % within Cat_prelim | 10.6% | 16.1% | 35.4% | 24.2% |
|  |  | % of Total | 7.9% | 12.1% | 26.5% | 18.1% |
|  | preliminary | Count | 8 | 12 | 25 | 8 |
|  |  | % within Cat_prelim | 14.8% | 22.2% | 46.3% | 14.8% |
|  |  | % of Total | 3.7% | 5.6% | 11.6% | 3.7% |
| Total | | Count | 25 | 38 | 82 | 47 |
|  |  | % within Cat_prelim | 11.6% | 17.7% | 38.1% | 21.9% |
|  |  | % of Total | 11.6% | 17.7% | 38.1% | 21.9% |

| **Crosstab** | | | | | |
| --- | --- | --- | --- | --- | --- |
|  | | | lunch | | |
|  |  |  | 5 | 33 |  |
| Cat_prelim | categorical | Count | 21 | 1 | 161 |
|  |  | % within Cat_prelim | 13.0% | 0.6% | 100.0% |
|  |  | % of Total | 9.8% | 0.5% | 74.9% |
|  | preliminary | Count | 1 | 0 | 54 |
|  |  | % within Cat_prelim | 1.9% | 0.0% | 100.0% |
|  |  | % of Total | 0.5% | 0.0% | 25.1% |
| Total | | Count | 22 | 1 | 215 |
|  |  | % within Cat_prelim | 10.2% | 0.5% | 100.0% |
|  |  | % of Total | 10.2% | 0.5% | 100.0% |

| **Chi-Square Tests** | | | |
| --- | --- | --- | --- |
|  | Value | df | Asymp. Sig. (2-sided) |
| Pearson Chi-Square | 9.654^a^ | 5 | .086 |
| Likelihood Ratio | 11.739 | 5 | .039 |
| Linear-by-Linear Association | 3.131 | 1 | .077 |
| N of Valid Cases | 215 |  |  |

| a. 2 cells (16.7%) have expected count less than 5. The minimum expected count is .25. |
| --- |

**Cat_prelim * dinner**

| **Crosstab** | | | | | |
| --- | --- | --- | --- | --- | --- |
|  | | | dinner | | Total |
|  |  |  | yes | no |  |
| Cat_prelim | categorical | Count | 104 | 57 | 161 |
|  |  | % within Cat_prelim | 64.6% | 35.4% | 100.0% |
|  |  | % of Total | 48.4% | 26.5% | 74.9% |
|  | preliminary | Count | 17 | 37 | 54 |
|  |  | % within Cat_prelim | 31.5% | 68.5% | 100.0% |
|  |  | % of Total | 7.9% | 17.2% | 25.1% |
| Total | | Count | 121 | 94 | 215 |
|  |  | % within Cat_prelim | 56.3% | 43.7% | 100.0% |
|  |  | % of Total | 56.3% | 43.7% | 100.0% |

| **Chi-Square Tests** | | | | | |
| --- | --- | --- | --- | --- | --- |
|  | Value | df | Asymp. Sig. (2-sided) | Exact Sig. (2-sided) | Exact Sig. (1-sided) |
| Pearson Chi-Square | 18.021^a^ | 1 | .000 |  |  |
| Continuity Correction^b^ | 16.701 | 1 | .000 |  |  |
| Likelihood Ratio | 18.109 | 1 | .000 |  |  |
| Fisher's Exact Test |  |  |  | .000 | .000 |
| Linear-by-Linear Association | 17.938 | 1 | .000 |  |  |
| N of Valid Cases | 215 |  |  |  |  |

| a. 0 cells (0.0%) have expected count less than 5. The minimum expected count is 23.61. |
| --- |
| b. Computed only for a 2x2 table |

**Cat_prelim * tour_optional**

| **Crosstab** | | | | | |
| --- | --- | --- | --- | --- | --- |
|  | | | tour_optional | | Total |
|  |  |  | yes | no |  |
| Cat_prelim | categorical | Count | 43 | 118 | 161 |
|  |  | % within Cat_prelim | 26.7% | 73.3% | 100.0% |
|  |  | % of Total | 20.0% | 54.9% | 74.9% |
|  | preliminary | Count | 31 | 23 | 54 |
|  |  | % within Cat_prelim | 57.4% | 42.6% | 100.0% |
|  |  | % of Total | 14.4% | 10.7% | 25.1% |
| Total | | Count | 74 | 141 | 215 |
|  |  | % within Cat_prelim | 34.4% | 65.6% | 100.0% |
|  |  | % of Total | 34.4% | 65.6% | 100.0% |

| **Chi-Square Tests** | | | | | |
| --- | --- | --- | --- | --- | --- |
|  | Value | df | Asymp. Sig. (2-sided) | Exact Sig. (2-sided) | Exact Sig. (1-sided) |
| Pearson Chi-Square | 16.884^a^ | 1 | .000 |  |  |
| Continuity Correction^b^ | 15.551 | 1 | .000 |  |  |
| Likelihood Ratio | 16.285 | 1 | .000 |  |  |
| Fisher's Exact Test |  |  |  | .000 | .000 |
| Linear-by-Linear Association | 16.805 | 1 | .000 |  |  |
| N of Valid Cases | 215 |  |  |  |  |

| a. 0 cells (0.0%) have expected count less than 5. The minimum expected count is 18.59. |
| --- |
| b. Computed only for a 2x2 table |

**Cat_prelim * exit_interview**

| **Crosstab** | | | | | |
| --- | --- | --- | --- | --- | --- |
|  | | | exit_interview | | Total |
|  |  |  | neccesary | unneccesary |  |
| Cat_prelim | categorical | Count | 85 | 76 | 161 |
|  |  | % within Cat_prelim | 52.8% | 47.2% | 100.0% |
|  |  | % of Total | 39.5% | 35.3% | 74.9% |
|  | preliminary | Count | 11 | 43 | 54 |
|  |  | % within Cat_prelim | 20.4% | 79.6% | 100.0% |
|  |  | % of Total | 5.1% | 20.0% | 25.1% |
| Total | | Count | 96 | 119 | 215 |
|  |  | % within Cat_prelim | 44.7% | 55.3% | 100.0% |
|  |  | % of Total | 44.7% | 55.3% | 100.0% |

| **Chi-Square Tests** | | | | | |
| --- | --- | --- | --- | --- | --- |
|  | Value | df | Asymp. Sig. (2-sided) | Exact Sig. (2-sided) | Exact Sig. (1-sided) |
| Pearson Chi-Square | 17.202^a^ | 1 | .000 |  |  |
| Continuity Correction^b^ | 15.915 | 1 | .000 |  |  |
| Likelihood Ratio | 18.305 | 1 | .000 |  |  |
| Fisher's Exact Test |  |  |  | .000 | .000 |
| Linear-by-Linear Association | 17.122 | 1 | .000 |  |  |
| N of Valid Cases | 215 |  |  |  |  |

| a. 0 cells (0.0%) have expected count less than 5. The minimum expected count is 24.11. |
| --- |
| b. Computed only for a 2x2 table |

**RECODE**

**candidate * INT_phone_recode**

| **Crosstab** | | | | | |
| --- | --- | --- | --- | --- | --- |
|  | | | INT_phone_recode | | Total |
|  |  |  | preferred | not preferred |  |
| candidate | US categorical | Count | 3 | 69 | 72 |
|  |  | % within candidate | 4.2% | 95.8% | 100.0% |
|  |  | % of Total | 1.8% | 42.1% | 43.9% |
|  | IM categorical | Count | 6 | 50 | 56 |
|  |  | % within candidate | 10.7% | 89.3% | 100.0% |
|  |  | % of Total | 3.7% | 30.5% | 34.1% |
|  | Preliminary | Count | 13 | 23 | 36 |
|  |  | % within candidate | 36.1% | 63.9% | 100.0% |
|  |  | % of Total | 7.9% | 14.0% | 22.0% |
| Total | | Count | 22 | 142 | 164 |
|  |  | % within candidate | 13.4% | 86.6% | 100.0% |
|  |  | % of Total | 13.4% | 86.6% | 100.0% |

| **Chi-Square Tests** | | | |
| --- | --- | --- | --- |
|  | Value | df | Asymp. Sig. (2-sided) |
| Pearson Chi-Square | 21.619^a^ | 2 | .000 |
| Likelihood Ratio | 19.126 | 2 | .000 |
| Linear-by-Linear Association | 18.799 | 1 | .000 |
| N of Valid Cases | 164 |  |  |

| a. 1 cells (16.7%) have expected count less than 5. The minimum expected count is 4.83. |
| --- |

**candidate * INT_lunch_recode**

| **Crosstab** | | | | | |
| --- | --- | --- | --- | --- | --- |
|  | | | INT_lunch_recode | | Total |
|  |  |  | 1 | 2 |  |
| candidate | US categorical | Count | 59 | 6 | 65 |
|  |  | % within candidate | 90.8% | 9.2% | 100.0% |
|  |  | % of Total | 40.7% | 4.1% | 44.8% |
|  | IM categorical | Count | 27 | 18 | 45 |
|  |  | % within candidate | 60.0% | 40.0% | 100.0% |
|  |  | % of Total | 18.6% | 12.4% | 31.0% |
|  | Preliminary | Count | 26 | 9 | 35 |
|  |  | % within candidate | 74.3% | 25.7% | 100.0% |
|  |  | % of Total | 17.9% | 6.2% | 24.1% |
| Total | | Count | 112 | 33 | 145 |
|  |  | % within candidate | 77.2% | 22.8% | 100.0% |
|  |  | % of Total | 77.2% | 22.8% | 100.0% |

| **Chi-Square Tests** | | | |
| --- | --- | --- | --- |
|  | Value | df | Asymp. Sig. (2-sided) |
| Pearson Chi-Square | 14.550^a^ | 2 | .001 |
| Likelihood Ratio | 15.045 | 2 | .001 |
| Linear-by-Linear Association | 5.817 | 1 | .016 |
| N of Valid Cases | 145 |  |  |

| a. 0 cells (0.0%) have expected count less than 5. The minimum expected count is 7.97. |
| --- |

**candidate * INT_one_on_one_recode**

| **Crosstab** | | | | | |
| --- | --- | --- | --- | --- | --- |
|  | | | INT_one_on_one_recode | | Total |
|  |  |  | 1 | 2 |  |
| candidate | US categorical | Count | 94 | 2 | 96 |
|  |  | % within candidate | 97.9% | 2.1% | 100.0% |
|  |  | % of Total | 43.7% | 0.9% | 44.7% |
|  | IM categorical | Count | 63 | 2 | 65 |
|  |  | % within candidate | 96.9% | 3.1% | 100.0% |
|  |  | % of Total | 29.3% | 0.9% | 30.2% |
|  | Preliminary | Count | 54 | 0 | 54 |
|  |  | % within candidate | 100.0% | 0.0% | 100.0% |
|  |  | % of Total | 25.1% | 0.0% | 25.1% |
| Total | | Count | 211 | 4 | 215 |
|  |  | % within candidate | 98.1% | 1.9% | 100.0% |
|  |  | % of Total | 98.1% | 1.9% | 100.0% |

| **Chi-Square Tests** | | | |
| --- | --- | --- | --- |
|  | Value | df | Asymp. Sig. (2-sided) |
| Pearson Chi-Square | 1.577^a^ | 2 | .455 |
| Likelihood Ratio | 2.494 | 2 | .287 |
| Linear-by-Linear Association | .571 | 1 | .450 |
| N of Valid Cases | 215 |  |  |

| a. 3 cells (50.0%) have expected count less than 5. The minimum expected count is 1.00. |
| --- |

**candidate * INT_panel2_recode**

| **Crosstab** | | | | | |
| --- | --- | --- | --- | --- | --- |
|  | | | INT_panel2_recode | | Total |
|  |  |  | 1 | 2 |  |
| candidate | US categorical | Count | 33 | 34 | 67 |
|  |  | % within candidate | 49.3% | 50.7% | 100.0% |
|  |  | % of Total | 21.4% | 22.1% | 43.5% |
|  | IM categorical | Count | 29 | 14 | 43 |
|  |  | % within candidate | 67.4% | 32.6% | 100.0% |
|  |  | % of Total | 18.8% | 9.1% | 27.9% |
|  | Preliminary | Count | 15 | 29 | 44 |
|  |  | % within candidate | 34.1% | 65.9% | 100.0% |
|  |  | % of Total | 9.7% | 18.8% | 28.6% |
| Total | | Count | 77 | 77 | 154 |
|  |  | % within candidate | 50.0% | 50.0% | 100.0% |
|  |  | % of Total | 50.0% | 50.0% | 100.0% |

| **Chi-Square Tests** | | | |
| --- | --- | --- | --- |
|  | Value | df | Asymp. Sig. (2-sided) |
| Pearson Chi-Square | 9.702^a^ | 2 | .008 |
| Likelihood Ratio | 9.892 | 2 | .007 |
| Linear-by-Linear Association | 1.561 | 1 | .212 |
| N of Valid Cases | 154 |  |  |

| a. 0 cells (0.0%) have expected count less than 5. The minimum expected count is 21.50. |
| --- |

**candidate * INT_group_recode**

| **Crosstab** | | | | | |
| --- | --- | --- | --- | --- | --- |
|  | | | INT_group_recode | | Total |
|  |  |  | 1 | 2 |  |
| candidate | US categorical | Count | 3 | 81 | 84 |
|  |  | % within candidate | 3.6% | 96.4% | 100.0% |
|  |  | % of Total | 1.6% | 44.5% | 46.2% |
|  | IM categorical | Count | 5 | 46 | 51 |
|  |  | % within candidate | 9.8% | 90.2% | 100.0% |
|  |  | % of Total | 2.7% | 25.3% | 28.0% |
|  | Preliminary | Count | 0 | 47 | 47 |
|  |  | % within candidate | 0.0% | 100.0% | 100.0% |
|  |  | % of Total | 0.0% | 25.8% | 25.8% |
| Total | | Count | 8 | 174 | 182 |
|  |  | % within candidate | 4.4% | 95.6% | 100.0% |
|  |  | % of Total | 4.4% | 95.6% | 100.0% |

| **Chi-Square Tests** | | | |
| --- | --- | --- | --- |
|  | Value | df | Asymp. Sig. (2-sided) |
| Pearson Chi-Square | 5.846^a^ | 2 | .054 |
| Likelihood Ratio | 7.035 | 2 | .030 |
| Linear-by-Linear Association | .362 | 1 | .548 |
| N of Valid Cases | 182 |  |  |

| a. 3 cells (50.0%) have expected count less than 5. The minimum expected count is 2.07. |
| --- |

**candidate * start7_8_recode**

| **Crosstab** | | | | | |
| --- | --- | --- | --- | --- | --- |
|  | | | start7_8_recode | | Total |
|  |  |  | 1 | 2 |  |
| candidate | US categorical | Count | 43 | 31 | 74 |
|  |  | % within candidate | 58.1% | 41.9% | 100.0% |
|  |  | % of Total | 27.0% | 19.5% | 46.5% |
|  | IM categorical | Count | 19 | 29 | 48 |
|  |  | % within candidate | 39.6% | 60.4% | 100.0% |
|  |  | % of Total | 11.9% | 18.2% | 30.2% |
|  | Preliminary | Count | 15 | 22 | 37 |
|  |  | % within candidate | 40.5% | 59.5% | 100.0% |
|  |  | % of Total | 9.4% | 13.8% | 23.3% |
| Total | | Count | 77 | 82 | 159 |
|  |  | % within candidate | 48.4% | 51.6% | 100.0% |
|  |  | % of Total | 48.4% | 51.6% | 100.0% |

| **Chi-Square Tests** | | | |
| --- | --- | --- | --- |
|  | Value | df | Asymp. Sig. (2-sided) |
| Pearson Chi-Square | 5.202^a^ | 2 | .074 |
| Likelihood Ratio | 5.228 | 2 | .073 |
| Linear-by-Linear Association | 3.950 | 1 | .047 |
| N of Valid Cases | 159 |  |  |

| a. 0 cells (0.0%) have expected count less than 5. The minimum expected count is 17.92. |
| --- |

**candidate * start8_9_recode**

| **Crosstab** | | | | | |
| --- | --- | --- | --- | --- | --- |
|  | | | start8_9_recode | | Total |
|  |  |  | 1 | 2 |  |
| candidate | US categorical | Count | 84 | 8 | 92 |
|  |  | % within candidate | 91.3% | 8.7% | 100.0% |
|  |  | % of Total | 41.8% | 4.0% | 45.8% |
|  | IM categorical | Count | 47 | 13 | 60 |
|  |  | % within candidate | 78.3% | 21.7% | 100.0% |
|  |  | % of Total | 23.4% | 6.5% | 29.9% |
|  | Preliminary | Count | 45 | 4 | 49 |
|  |  | % within candidate | 91.8% | 8.2% | 100.0% |
|  |  | % of Total | 22.4% | 2.0% | 24.4% |
| Total | | Count | 176 | 25 | 201 |
|  |  | % within candidate | 87.6% | 12.4% | 100.0% |
|  |  | % of Total | 87.6% | 12.4% | 100.0% |

| **Chi-Square Tests** | | | |
| --- | --- | --- | --- |
|  | Value | df | Asymp. Sig. (2-sided) |
| Pearson Chi-Square | 6.697^a^ | 2 | .035 |
| Likelihood Ratio | 6.186 | 2 | .045 |
| Linear-by-Linear Association | .126 | 1 | .723 |
| N of Valid Cases | 201 |  |  |

| a. 0 cells (0.0%) have expected count less than 5. The minimum expected count is 6.09. |
| --- |

**candidate * start9_10_recode**

| **Crosstab** | | | | | |
| --- | --- | --- | --- | --- | --- |
|  | | | start9_10_recode | | Total |
|  |  |  | 1 | 2 |  |
| candidate | US categorical | Count | 51 | 4 | 55 |
|  |  | % within candidate | 92.7% | 7.3% | 100.0% |
|  |  | % of Total | 36.7% | 2.9% | 39.6% |
|  | IM categorical | Count | 43 | 1 | 44 |
|  |  | % within candidate | 97.7% | 2.3% | 100.0% |
|  |  | % of Total | 30.9% | 0.7% | 31.7% |
|  | Preliminary | Count | 38 | 2 | 40 |
|  |  | % within candidate | 95.0% | 5.0% | 100.0% |
|  |  | % of Total | 27.3% | 1.4% | 28.8% |
| Total | | Count | 132 | 7 | 139 |
|  |  | % within candidate | 95.0% | 5.0% | 100.0% |
|  |  | % of Total | 95.0% | 5.0% | 100.0% |

| **Chi-Square Tests** | | | |
| --- | --- | --- | --- |
|  | Value | df | Asymp. Sig. (2-sided) |
| Pearson Chi-Square | 1.278^a^ | 2 | .528 |
| Likelihood Ratio | 1.385 | 2 | .500 |
| Linear-by-Linear Association | .344 | 1 | .557 |
| N of Valid Cases | 139 |  |  |

| a. 3 cells (50.0%) have expected count less than 5. The minimum expected count is 2.01. |
| --- |

**candidate * start10_11_recode**

| **Crosstab** | | | | | |
| --- | --- | --- | --- | --- | --- |
|  | | | start10_11_recode | | Total |
|  |  |  | 1 | 2 |  |
| candidate | US categorical | Count | 11 | 62 | 73 |
|  |  | % within candidate | 15.1% | 84.9% | 100.0% |
|  |  | % of Total | 6.8% | 38.3% | 45.1% |
|  | IM categorical | Count | 15 | 34 | 49 |
|  |  | % within candidate | 30.6% | 69.4% | 100.0% |
|  |  | % of Total | 9.3% | 21.0% | 30.2% |
|  | Preliminary | Count | 8 | 32 | 40 |
|  |  | % within candidate | 20.0% | 80.0% | 100.0% |
|  |  | % of Total | 4.9% | 19.8% | 24.7% |
| Total | | Count | 34 | 128 | 162 |
|  |  | % within candidate | 21.0% | 79.0% | 100.0% |
|  |  | % of Total | 21.0% | 79.0% | 100.0% |

| **Chi-Square Tests** | | | |
| --- | --- | --- | --- |
|  | Value | df | Asymp. Sig. (2-sided) |
| Pearson Chi-Square | 4.303^a^ | 2 | .116 |
| Likelihood Ratio | 4.184 | 2 | .123 |
| Linear-by-Linear Association | .869 | 1 | .351 |
| N of Valid Cases | 162 |  |  |

| a. 0 cells (0.0%) have expected count less than 5. The minimum expected count is 8.40. |
| --- |

**candidate * start11_12_recode**

| **Crosstab** | | | | | |
| --- | --- | --- | --- | --- | --- |
|  | | | start11_12_recode | | Total |
|  |  |  | 1 | 2 |  |
| candidate | US categorical | Count | 3 | 87 | 90 |
|  |  | % within candidate | 3.3% | 96.7% | 100.0% |
|  |  | % of Total | 1.5% | 43.7% | 45.2% |
|  | IM categorical | Count | 6 | 53 | 59 |
|  |  | % within candidate | 10.2% | 89.8% | 100.0% |
|  |  | % of Total | 3.0% | 26.6% | 29.6% |
|  | Preliminary | Count | 2 | 48 | 50 |
|  |  | % within candidate | 4.0% | 96.0% | 100.0% |
|  |  | % of Total | 1.0% | 24.1% | 25.1% |
| Total | | Count | 11 | 188 | 199 |
|  |  | % within candidate | 5.5% | 94.5% | 100.0% |
|  |  | % of Total | 5.5% | 94.5% | 100.0% |

| **Chi-Square Tests** | | | |
| --- | --- | --- | --- |
|  | Value | df | Asymp. Sig. (2-sided) |
| Pearson Chi-Square | 3.488^a^ | 2 | .175 |
| Likelihood Ratio | 3.182 | 2 | .204 |
| Linear-by-Linear Association | .212 | 1 | .645 |
| N of Valid Cases | 199 |  |  |

| a. 3 cells (50.0%) have expected count less than 5. The minimum expected count is 2.76. |
| --- |

**candidate * lengthLT2_recode**

| **Crosstab** | | | | | |
| --- | --- | --- | --- | --- | --- |
|  | | | lengthLT2_recode | | Total |
|  |  |  | 1 | 2 |  |
| candidate | US categorical | Count | 3 | 77 | 80 |
|  |  | % within candidate | 3.8% | 96.3% | 100.0% |
|  |  | % of Total | 1.7% | 43.8% | 45.5% |
|  | IM categorical | Count | 8 | 52 | 60 |
|  |  | % within candidate | 13.3% | 86.7% | 100.0% |
|  |  | % of Total | 4.5% | 29.5% | 34.1% |
|  | Preliminary | Count | 10 | 26 | 36 |
|  |  | % within candidate | 27.8% | 72.2% | 100.0% |
|  |  | % of Total | 5.7% | 14.8% | 20.5% |
| Total | | Count | 21 | 155 | 176 |
|  |  | % within candidate | 11.9% | 88.1% | 100.0% |
|  |  | % of Total | 11.9% | 88.1% | 100.0% |

| **Chi-Square Tests** | | | |
| --- | --- | --- | --- |
|  | Value | df | Asymp. Sig. (2-sided) |
| Pearson Chi-Square | 13.811^a^ | 2 | .001 |
| Likelihood Ratio | 13.431 | 2 | .001 |
| Linear-by-Linear Association | 13.523 | 1 | .000 |
| N of Valid Cases | 176 |  |  |

| a. 1 cells (16.7%) have expected count less than 5. The minimum expected count is 4.30. |
| --- |

**candidate * length2_recode**

| **Crosstab** | | | | | |
| --- | --- | --- | --- | --- | --- |
|  | | | length2_recode | | Total |
|  |  |  | 1 | 2 |  |
| candidate | US categorical | Count | 33 | 29 | 62 |
|  |  | % within candidate | 53.2% | 46.8% | 100.0% |
|  |  | % of Total | 22.6% | 19.9% | 42.5% |
|  | IM categorical | Count | 21 | 23 | 44 |
|  |  | % within candidate | 47.7% | 52.3% | 100.0% |
|  |  | % of Total | 14.4% | 15.8% | 30.1% |
|  | Preliminary | Count | 38 | 2 | 40 |
|  |  | % within candidate | 95.0% | 5.0% | 100.0% |
|  |  | % of Total | 26.0% | 1.4% | 27.4% |
| Total | | Count | 92 | 54 | 146 |
|  |  | % within candidate | 63.0% | 37.0% | 100.0% |
|  |  | % of Total | 63.0% | 37.0% | 100.0% |

| **Chi-Square Tests** | | | |
| --- | --- | --- | --- |
|  | Value | df | Asymp. Sig. (2-sided) |
| Pearson Chi-Square | 24.520^a^ | 2 | .000 |
| Likelihood Ratio | 29.915 | 2 | .000 |
| Linear-by-Linear Association | 15.364 | 1 | .000 |
| N of Valid Cases | 146 |  |  |

| a. 0 cells (0.0%) have expected count less than 5. The minimum expected count is 14.79. |
| --- |

**candidate * length4_recode**

| **Crosstab** | | | | | |
| --- | --- | --- | --- | --- | --- |
|  | | | length4_recode | | Total |
|  |  |  | 1 | 2 |  |
| candidate | US categorical | Count | 83 | 2 | 85 |
|  |  | % within candidate | 97.6% | 2.4% | 100.0% |
|  |  | % of Total | 45.6% | 1.1% | 46.7% |
|  | IM categorical | Count | 52 | 2 | 54 |
|  |  | % within candidate | 96.3% | 3.7% | 100.0% |
|  |  | % of Total | 28.6% | 1.1% | 29.7% |
|  | Preliminary | Count | 43 | 0 | 43 |
|  |  | % within candidate | 100.0% | 0.0% | 100.0% |
|  |  | % of Total | 23.6% | 0.0% | 23.6% |
| Total | | Count | 178 | 4 | 182 |
|  |  | % within candidate | 97.8% | 2.2% | 100.0% |
|  |  | % of Total | 97.8% | 2.2% | 100.0% |

| **Chi-Square Tests** | | | |
| --- | --- | --- | --- |
|  | Value | df | Asymp. Sig. (2-sided) |
| Pearson Chi-Square | 1.546^a^ | 2 | .462 |
| Likelihood Ratio | 2.394 | 2 | .302 |
| Linear-by-Linear Association | .454 | 1 | .501 |
| N of Valid Cases | 182 |  |  |

| a. 3 cells (50.0%) have expected count less than 5. The minimum expected count is .95. |
| --- |

**candidate * length6_recode**

| **Crosstab** | | | | | |
| --- | --- | --- | --- | --- | --- |
|  | | | length6_recode | | Total |
|  |  |  | 1 | 2 |  |
| candidate | US categorical | Count | 63 | 17 | 80 |
|  |  | % within candidate | 78.8% | 21.3% | 100.0% |
|  |  | % of Total | 36.0% | 9.7% | 45.7% |
|  | IM categorical | Count | 41 | 10 | 51 |
|  |  | % within candidate | 80.4% | 19.6% | 100.0% |
|  |  | % of Total | 23.4% | 5.7% | 29.1% |
|  | Preliminary | Count | 16 | 28 | 44 |
|  |  | % within candidate | 36.4% | 63.6% | 100.0% |
|  |  | % of Total | 9.1% | 16.0% | 25.1% |
| Total | | Count | 120 | 55 | 175 |
|  |  | % within candidate | 68.6% | 31.4% | 100.0% |
|  |  | % of Total | 68.6% | 31.4% | 100.0% |

| **Chi-Square Tests** | | | |
| --- | --- | --- | --- |
|  | Value | df | Asymp. Sig. (2-sided) |
| Pearson Chi-Square | 28.332^a^ | 2 | .000 |
| Likelihood Ratio | 26.946 | 2 | .000 |
| Linear-by-Linear Association | 19.703 | 1 | .000 |
| N of Valid Cases | 175 |  |  |

| a. 0 cells (0.0%) have expected count less than 5. The minimum expected count is 13.83. |
| --- |

**candidate * length8_recode**

| **Crosstab** | | | | | |
| --- | --- | --- | --- | --- | --- |
|  | | | length8_recode | | Total |
|  |  |  | 1 | 2 |  |
| candidate | US categorical | Count | 10 | 67 | 77 |
|  |  | % within candidate | 13.0% | 87.0% | 100.0% |
|  |  | % of Total | 5.5% | 37.0% | 42.5% |
|  | IM categorical | Count | 8 | 43 | 51 |
|  |  | % within candidate | 15.7% | 84.3% | 100.0% |
|  |  | % of Total | 4.4% | 23.8% | 28.2% |
|  | Preliminary | Count | 1 | 52 | 53 |
|  |  | % within candidate | 1.9% | 98.1% | 100.0% |
|  |  | % of Total | 0.6% | 28.7% | 29.3% |
| Total | | Count | 19 | 162 | 181 |
|  |  | % within candidate | 10.5% | 89.5% | 100.0% |
|  |  | % of Total | 10.5% | 89.5% | 100.0% |

| **Chi-Square Tests** | | | |
| --- | --- | --- | --- |
|  | Value | df | Asymp. Sig. (2-sided) |
| Pearson Chi-Square | 6.152^a^ | 2 | .046 |
| Likelihood Ratio | 7.887 | 2 | .019 |
| Linear-by-Linear Association | 3.505 | 1 | .061 |
| N of Valid Cases | 181 |  |  |

| a. 0 cells (0.0%) have expected count less than 5. The minimum expected count is 5.35. |
| --- |

**candidate * faculty1_recode**

| **Crosstab** | | | | | |
| --- | --- | --- | --- | --- | --- |
|  | | | faculty1_recode | | Total |
|  |  |  | 1 | 2 |  |
| candidate | US categorical | Count | 18 | 35 | 53 |
|  |  | % within candidate | 34.0% | 66.0% | 100.0% |
|  |  | % of Total | 13.4% | 26.1% | 39.6% |
|  | IM categorical | Count | 17 | 27 | 44 |
|  |  | % within candidate | 38.6% | 61.4% | 100.0% |
|  |  | % of Total | 12.7% | 20.1% | 32.8% |
|  | Preliminary | Count | 14 | 23 | 37 |
|  |  | % within candidate | 37.8% | 62.2% | 100.0% |
|  |  | % of Total | 10.4% | 17.2% | 27.6% |
| Total | | Count | 49 | 85 | 134 |
|  |  | % within candidate | 36.6% | 63.4% | 100.0% |
|  |  | % of Total | 36.6% | 63.4% | 100.0% |

| **Chi-Square Tests** | | | |
| --- | --- | --- | --- |
|  | Value | df | Asymp. Sig. (2-sided) |
| Pearson Chi-Square | .262^a^ | 2 | .877 |
| Likelihood Ratio | .263 | 2 | .877 |
| Linear-by-Linear Association | .166 | 1 | .683 |
| N of Valid Cases | 134 |  |  |

| a. 0 cells (0.0%) have expected count less than 5. The minimum expected count is 13.53. |
| --- |

**candidate * faculty2_recode**

| **Crosstab** | | | | | |
| --- | --- | --- | --- | --- | --- |
|  | | | faculty2_recode | | Total |
|  |  |  | 1 | 2 |  |
| candidate | US categorical | Count | 90 | 4 | 94 |
|  |  | % within candidate | 95.7% | 4.3% | 100.0% |
|  |  | % of Total | 43.7% | 1.9% | 45.6% |
|  | IM categorical | Count | 55 | 5 | 60 |
|  |  | % within candidate | 91.7% | 8.3% | 100.0% |
|  |  | % of Total | 26.7% | 2.4% | 29.1% |
|  | Preliminary | Count | 50 | 2 | 52 |
|  |  | % within candidate | 96.2% | 3.8% | 100.0% |
|  |  | % of Total | 24.3% | 1.0% | 25.2% |
| Total | | Count | 195 | 11 | 206 |
|  |  | % within candidate | 94.7% | 5.3% | 100.0% |
|  |  | % of Total | 94.7% | 5.3% | 100.0% |

| **Chi-Square Tests** | | | |
| --- | --- | --- | --- |
|  | Value | df | Asymp. Sig. (2-sided) |
| Pearson Chi-Square | 1.512^a^ | 2 | .470 |
| Likelihood Ratio | 1.403 | 2 | .496 |
| Linear-by-Linear Association | .008 | 1 | .927 |
| N of Valid Cases | 206 |  |  |

| a. 2 cells (33.3%) have expected count less than 5. The minimum expected count is 2.78. |
| --- |

**candidate * faculty3_recode**

| **Crosstab** | | | | | |
| --- | --- | --- | --- | --- | --- |
|  | | | faculty3_recode | | Total |
|  |  |  | 1 | 2 |  |
| candidate | US categorical | Count | 75 | 2 | 77 |
|  |  | % within candidate | 97.4% | 2.6% | 100.0% |
|  |  | % of Total | 45.7% | 1.2% | 47.0% |
|  | IM categorical | Count | 47 | 0 | 47 |
|  |  | % within candidate | 100.0% | 0.0% | 100.0% |
|  |  | % of Total | 28.7% | 0.0% | 28.7% |
|  | Preliminary | Count | 40 | 0 | 40 |
|  |  | % within candidate | 100.0% | 0.0% | 100.0% |
|  |  | % of Total | 24.4% | 0.0% | 24.4% |
| Total | | Count | 162 | 2 | 164 |
|  |  | % within candidate | 98.8% | 1.2% | 100.0% |
|  |  | % of Total | 98.8% | 1.2% | 100.0% |

| **Chi-Square Tests** | | | |
| --- | --- | --- | --- |
|  | Value | df | Asymp. Sig. (2-sided) |
| Pearson Chi-Square | 2.288^a^ | 2 | .319 |
| Likelihood Ratio | 3.052 | 2 | .217 |
| Linear-by-Linear Association | 1.821 | 1 | .177 |
| N of Valid Cases | 164 |  |  |

| a. 3 cells (50.0%) have expected count less than 5. The minimum expected count is .49. |
| --- |

**candidate * faculty4_recode**

| **Crosstab** | | | | | |
| --- | --- | --- | --- | --- | --- |
|  | | | faculty4_recode | | Total |
|  |  |  | 1 | 2 |  |
| candidate | US categorical | Count | 6 | 59 | 65 |
|  |  | % within candidate | 9.2% | 90.8% | 100.0% |
|  |  | % of Total | 4.1% | 40.1% | 44.2% |
|  | IM categorical | Count | 9 | 38 | 47 |
|  |  | % within candidate | 19.1% | 80.9% | 100.0% |
|  |  | % of Total | 6.1% | 25.9% | 32.0% |
|  | Preliminary | Count | 4 | 31 | 35 |
|  |  | % within candidate | 11.4% | 88.6% | 100.0% |
|  |  | % of Total | 2.7% | 21.1% | 23.8% |
| Total | | Count | 19 | 128 | 147 |
|  |  | % within candidate | 12.9% | 87.1% | 100.0% |
|  |  | % of Total | 12.9% | 87.1% | 100.0% |

| **Chi-Square Tests** | | | |
| --- | --- | --- | --- |
|  | Value | df | Asymp. Sig. (2-sided) |
| Pearson Chi-Square | 2.476^a^ | 2 | .290 |
| Likelihood Ratio | 2.375 | 2 | .305 |
| Linear-by-Linear Association | .331 | 1 | .565 |
| N of Valid Cases | 147 |  |  |

| a. 1 cells (16.7%) have expected count less than 5. The minimum expected count is 4.52. |
| --- |

**candidate * facultyGT4_recode**

| **Crosstab** | | | | | |
| --- | --- | --- | --- | --- | --- |
|  | | | facultyGT4_recode | | Total |
|  |  |  | 1 | 2 |  |
| candidate | US categorical | Count | 3 | 92 | 95 |
|  |  | % within candidate | 3.2% | 96.8% | 100.0% |
|  |  | % of Total | 1.4% | 44.0% | 45.5% |
|  | IM categorical | Count | 2 | 60 | 62 |
|  |  | % within candidate | 3.2% | 96.8% | 100.0% |
|  |  | % of Total | 1.0% | 28.7% | 29.7% |
|  | Preliminary | Count | 0 | 52 | 52 |
|  |  | % within candidate | 0.0% | 100.0% | 100.0% |
|  |  | % of Total | 0.0% | 24.9% | 24.9% |
| Total | | Count | 5 | 204 | 209 |
|  |  | % within candidate | 2.4% | 97.6% | 100.0% |
|  |  | % of Total | 2.4% | 97.6% | 100.0% |

| **Chi-Square Tests** | | | |
| --- | --- | --- | --- |
|  | Value | df | Asymp. Sig. (2-sided) |
| Pearson Chi-Square | 1.697^a^ | 2 | .428 |
| Likelihood Ratio | 2.902 | 2 | .234 |
| Linear-by-Linear Association | 1.199 | 1 | .274 |
| N of Valid Cases | 209 |  |  |

| a. 3 cells (50.0%) have expected count less than 5. The minimum expected count is 1.24. |
| --- |

**candidate * lengthLT15_recode**

| **Crosstab** | | | | | |
| --- | --- | --- | --- | --- | --- |
|  | | | lengthLT15_recode | | Total |
|  |  |  | 1 | 2 |  |
| candidate | US categorical | Count | 34 | 27 | 61 |
|  |  | % within candidate | 55.7% | 44.3% | 100.0% |
|  |  | % of Total | 21.9% | 17.4% | 39.4% |
|  | IM categorical | Count | 43 | 7 | 50 |
|  |  | % within candidate | 86.0% | 14.0% | 100.0% |
|  |  | % of Total | 27.7% | 4.5% | 32.3% |
|  | Preliminary | Count | 43 | 1 | 44 |
|  |  | % within candidate | 97.7% | 2.3% | 100.0% |
|  |  | % of Total | 27.7% | 0.6% | 28.4% |
| Total | | Count | 120 | 35 | 155 |
|  |  | % within candidate | 77.4% | 22.6% | 100.0% |
|  |  | % of Total | 77.4% | 22.6% | 100.0% |

| **Chi-Square Tests** | | | |
| --- | --- | --- | --- |
|  | Value | df | Asymp. Sig. (2-sided) |
| Pearson Chi-Square | 28.889^a^ | 2 | .000 |
| Likelihood Ratio | 31.789 | 2 | .000 |
| Linear-by-Linear Association | 27.064 | 1 | .000 |
| N of Valid Cases | 155 |  |  |

| a. 0 cells (0.0%) have expected count less than 5. The minimum expected count is 9.94. |
| --- |

**candidate * length15_30_recode**

| **Crosstab** | | | | | |
| --- | --- | --- | --- | --- | --- |
|  | | | length15_30_recode | | Total |
|  |  |  | 1 | 2 |  |
| candidate | US categorical | Count | 90 | 2 | 92 |
|  |  | % within candidate | 97.8% | 2.2% | 100.0% |
|  |  | % of Total | 42.9% | 1.0% | 43.8% |
|  | IM categorical | Count | 61 | 3 | 64 |
|  |  | % within candidate | 95.3% | 4.7% | 100.0% |
|  |  | % of Total | 29.0% | 1.4% | 30.5% |
|  | Preliminary | Count | 54 | 0 | 54 |
|  |  | % within candidate | 100.0% | 0.0% | 100.0% |
|  |  | % of Total | 25.7% | 0.0% | 25.7% |
| Total | | Count | 205 | 5 | 210 |
|  |  | % within candidate | 97.6% | 2.4% | 100.0% |
|  |  | % of Total | 97.6% | 2.4% | 100.0% |

| **Chi-Square Tests** | | | |
| --- | --- | --- | --- |
|  | Value | df | Asymp. Sig. (2-sided) |
| Pearson Chi-Square | 2.799^a^ | 2 | .247 |
| Likelihood Ratio | 3.767 | 2 | .152 |
| Linear-by-Linear Association | .369 | 1 | .543 |
| N of Valid Cases | 210 |  |  |

| a. 3 cells (50.0%) have expected count less than 5. The minimum expected count is 1.29. |
| --- |

**candidate * length30_45_recode**

| **Crosstab** | | | | | |
| --- | --- | --- | --- | --- | --- |
|  | | | length30_45_recode | | Total |
|  |  |  | 1 | 2 |  |
| candidate | US categorical | Count | 60 | 1 | 61 |
|  |  | % within candidate | 98.4% | 1.6% | 100.0% |
|  |  | % of Total | 64.5% | 1.1% | 65.6% |
|  | IM categorical | Count | 21 | 0 | 21 |
|  |  | % within candidate | 100.0% | 0.0% | 100.0% |
|  |  | % of Total | 22.6% | 0.0% | 22.6% |
|  | Preliminary | Count | 11 | 0 | 11 |
|  |  | % within candidate | 100.0% | 0.0% | 100.0% |
|  |  | % of Total | 11.8% | 0.0% | 11.8% |
| Total | | Count | 92 | 1 | 93 |
|  |  | % within candidate | 98.9% | 1.1% | 100.0% |
|  |  | % of Total | 98.9% | 1.1% | 100.0% |

| **Chi-Square Tests** | | | |
| --- | --- | --- | --- |
|  | Value | df | Asymp. Sig. (2-sided) |
| Pearson Chi-Square | .530^a^ | 2 | .767 |
| Likelihood Ratio | .849 | 2 | .654 |
| Linear-by-Linear Association | .441 | 1 | .507 |
| N of Valid Cases | 93 |  |  |

| a. 3 cells (50.0%) have expected count less than 5. The minimum expected count is .12. |
| --- |

**candidate * length45_60_recode**

| **Crosstab** | | | | | |
| --- | --- | --- | --- | --- | --- |
|  | | | length45_60_recode | | Total |
|  |  |  | 1 | 2 |  |
| candidate | US categorical | Count | 6 | 68 | 74 |
|  |  | % within candidate | 8.1% | 91.9% | 100.0% |
|  |  | % of Total | 3.2% | 36.4% | 39.6% |
|  | IM categorical | Count | 3 | 57 | 60 |
|  |  | % within candidate | 5.0% | 95.0% | 100.0% |
|  |  | % of Total | 1.6% | 30.5% | 32.1% |
|  | Preliminary | Count | 0 | 53 | 53 |
|  |  | % within candidate | 0.0% | 100.0% | 100.0% |
|  |  | % of Total | 0.0% | 28.3% | 28.3% |
| Total | | Count | 9 | 178 | 187 |
|  |  | % within candidate | 4.8% | 95.2% | 100.0% |
|  |  | % of Total | 4.8% | 95.2% | 100.0% |

| **Chi-Square Tests** | | | |
| --- | --- | --- | --- |
|  | Value | df | Asymp. Sig. (2-sided) |
| Pearson Chi-Square | 4.438^a^ | 2 | .109 |
| Likelihood Ratio | 6.700 | 2 | .035 |
| Linear-by-Linear Association | 4.336 | 1 | .037 |
| N of Valid Cases | 187 |  |  |

| a. 3 cells (50.0%) have expected count less than 5. The minimum expected count is 2.55. |
| --- |

**candidate * lengthGT60_recode**

| **Crosstab** | | | | | |
| --- | --- | --- | --- | --- | --- |
|  | | | lengthGT60_recode | | Total |
|  |  |  | 1 | 2 |  |
| candidate | US categorical | Count | 2 | 94 | 96 |
|  |  | % within candidate | 2.1% | 97.9% | 100.0% |
|  |  | % of Total | 0.9% | 43.7% | 44.7% |
|  | IM categorical | Count | 3 | 62 | 65 |
|  |  | % within candidate | 4.6% | 95.4% | 100.0% |
|  |  | % of Total | 1.4% | 28.8% | 30.2% |
|  | Preliminary | Count | 0 | 54 | 54 |
|  |  | % within candidate | 0.0% | 100.0% | 100.0% |
|  |  | % of Total | 0.0% | 25.1% | 25.1% |
| Total | | Count | 5 | 210 | 215 |
|  |  | % within candidate | 2.3% | 97.7% | 100.0% |
|  |  | % of Total | 2.3% | 97.7% | 100.0% |

| **Chi-Square Tests** | | | |
| --- | --- | --- | --- |
|  | Value | df | Asymp. Sig. (2-sided) |
| Pearson Chi-Square | 2.811^a^ | 2 | .245 |
| Likelihood Ratio | 3.738 | 2 | .154 |
| Linear-by-Linear Association | .324 | 1 | .569 |
| N of Valid Cases | 215 |  |  |

| a. 3 cells (50.0%) have expected count less than 5. The minimum expected count is 1.26. |
| --- |

**candidate * INT_res_chief_recode**

| **Crosstab** | | | | | |
| --- | --- | --- | --- | --- | --- |
|  | | | INT_res_chief_recode | | Total |
|  |  |  | 1 | 2 |  |
| candidate | US categorical | Count | 18 | 65 | 83 |
|  |  | % within candidate | 21.7% | 78.3% | 100.0% |
|  |  | % of Total | 9.9% | 35.7% | 45.6% |
|  | IM categorical | Count | 5 | 52 | 57 |
|  |  | % within candidate | 8.8% | 91.2% | 100.0% |
|  |  | % of Total | 2.7% | 28.6% | 31.3% |
|  | Preliminary | Count | 5 | 37 | 42 |
|  |  | % within candidate | 11.9% | 88.1% | 100.0% |
|  |  | % of Total | 2.7% | 20.3% | 23.1% |
| Total | | Count | 28 | 154 | 182 |
|  |  | % within candidate | 15.4% | 84.6% | 100.0% |
|  |  | % of Total | 15.4% | 84.6% | 100.0% |

| **Chi-Square Tests** | | | |
| --- | --- | --- | --- |
|  | Value | df | Asymp. Sig. (2-sided) |
| Pearson Chi-Square | 4.838^a^ | 2 | .089 |
| Likelihood Ratio | 4.924 | 2 | .085 |
| Linear-by-Linear Association | 2.956 | 1 | .086 |
| N of Valid Cases | 182 |  |  |

| a. 0 cells (0.0%) have expected count less than 5. The minimum expected count is 6.46. |
| --- |

**candidate * INT_faculty_recode**

| **Crosstab** | | | | | |
| --- | --- | --- | --- | --- | --- |
|  | | | INT_faculty_recode | | Total |
|  |  |  | 1 | 2 |  |
| candidate | US categorical | Count | 23 | 34 | 57 |
|  |  | % within candidate | 40.4% | 59.6% | 100.0% |
|  |  | % of Total | 16.0% | 23.6% | 39.6% |
|  | IM categorical | Count | 8 | 40 | 48 |
|  |  | % within candidate | 16.7% | 83.3% | 100.0% |
|  |  | % of Total | 5.6% | 27.8% | 33.3% |
|  | Preliminary | Count | 13 | 26 | 39 |
|  |  | % within candidate | 33.3% | 66.7% | 100.0% |
|  |  | % of Total | 9.0% | 18.1% | 27.1% |
| Total | | Count | 44 | 100 | 144 |
|  |  | % within candidate | 30.6% | 69.4% | 100.0% |
|  |  | % of Total | 30.6% | 69.4% | 100.0% |

| **Chi-Square Tests** | | | |
| --- | --- | --- | --- |
|  | Value | df | Asymp. Sig. (2-sided) |
| Pearson Chi-Square | 7.083^a^ | 2 | .029 |
| Likelihood Ratio | 7.479 | 2 | .024 |
| Linear-by-Linear Association | 1.011 | 1 | .315 |
| N of Valid Cases | 144 |  |  |

| a. 0 cells (0.0%) have expected count less than 5. The minimum expected count is 11.92. |
| --- |

**candidate * INT_APD_recode**

| **Crosstab** | | | | | |
| --- | --- | --- | --- | --- | --- |
|  | | | INT_APD_recode | | Total |
|  |  |  | 1 | 2 |  |
| candidate | US categorical | Count | 67 | 5 | 72 |
|  |  | % within candidate | 93.1% | 6.9% | 100.0% |
|  |  | % of Total | 43.2% | 3.2% | 46.5% |
|  | IM categorical | Count | 34 | 7 | 41 |
|  |  | % within candidate | 82.9% | 17.1% | 100.0% |
|  |  | % of Total | 21.9% | 4.5% | 26.5% |
|  | Preliminary | Count | 37 | 5 | 42 |
|  |  | % within candidate | 88.1% | 11.9% | 100.0% |
|  |  | % of Total | 23.9% | 3.2% | 27.1% |
| Total | | Count | 138 | 17 | 155 |
|  |  | % within candidate | 89.0% | 11.0% | 100.0% |
|  |  | % of Total | 89.0% | 11.0% | 100.0% |

| **Chi-Square Tests** | | | |
| --- | --- | --- | --- |
|  | Value | df | Asymp. Sig. (2-sided) |
| Pearson Chi-Square | 2.796^a^ | 2 | .247 |
| Likelihood Ratio | 2.754 | 2 | .252 |
| Linear-by-Linear Association | 1.018 | 1 | .313 |
| N of Valid Cases | 155 |  |  |

| a. 2 cells (33.3%) have expected count less than 5. The minimum expected count is 4.50. |
| --- |

**candidate * INT_PD_recode**

| **Crosstab** | | | | | |
| --- | --- | --- | --- | --- | --- |
|  | | | INT_PD_recode | | Total |
|  |  |  | 1 | 2 |  |
| candidate | US categorical | Count | 79 | 7 | 86 |
|  |  | % within candidate | 91.9% | 8.1% | 100.0% |
|  |  | % of Total | 40.3% | 3.6% | 43.9% |
|  | IM categorical | Count | 61 | 2 | 63 |
|  |  | % within candidate | 96.8% | 3.2% | 100.0% |
|  |  | % of Total | 31.1% | 1.0% | 32.1% |
|  | Preliminary | Count | 46 | 1 | 47 |
|  |  | % within candidate | 97.9% | 2.1% | 100.0% |
|  |  | % of Total | 23.5% | 0.5% | 24.0% |
| Total | | Count | 186 | 10 | 196 |
|  |  | % within candidate | 94.9% | 5.1% | 100.0% |
|  |  | % of Total | 94.9% | 5.1% | 100.0% |

| **Chi-Square Tests** | | | |
| --- | --- | --- | --- |
|  | Value | df | Asymp. Sig. (2-sided) |
| Pearson Chi-Square | 2.981^a^ | 2 | .225 |
| Likelihood Ratio | 3.045 | 2 | .218 |
| Linear-by-Linear Association | 2.639 | 1 | .104 |
| N of Valid Cases | 196 |  |  |

| a. 3 cells (50.0%) have expected count less than 5. The minimum expected count is 2.40. |
| --- |

**candidate * INT_chair_recode**

| **Crosstab** | | | | | |
| --- | --- | --- | --- | --- | --- |
|  | | | INT_chair_recode | | Total |
|  |  |  | 1 | 2 |  |
| candidate | US categorical | Count | 5 | 81 | 86 |
|  |  | % within candidate | 5.8% | 94.2% | 100.0% |
|  |  | % of Total | 2.7% | 44.3% | 47.0% |
|  | IM categorical | Count | 22 | 29 | 51 |
|  |  | % within candidate | 43.1% | 56.9% | 100.0% |
|  |  | % of Total | 12.0% | 15.8% | 27.9% |
|  | Preliminary | Count | 7 | 39 | 46 |
|  |  | % within candidate | 15.2% | 84.8% | 100.0% |
|  |  | % of Total | 3.8% | 21.3% | 25.1% |
| Total | | Count | 34 | 149 | 183 |
|  |  | % within candidate | 18.6% | 81.4% | 100.0% |
|  |  | % of Total | 18.6% | 81.4% | 100.0% |

| **Chi-Square Tests** | | | |
| --- | --- | --- | --- |
|  | Value | df | Asymp. Sig. (2-sided) |
| Pearson Chi-Square | 29.940^a^ | 2 | .000 |
| Likelihood Ratio | 28.579 | 2 | .000 |
| Linear-by-Linear Association | 4.745 | 1 | .029 |
| N of Valid Cases | 183 |  |  |

| a. 0 cells (0.0%) have expected count less than 5. The minimum expected count is 8.55. |
| --- |

**candidate * Qstraightforward_recode**

| **Crosstab** | | | | | |
| --- | --- | --- | --- | --- | --- |
|  | | | Qstraightforward_recode | | Total |
|  |  |  | 1 | 2 |  |
| candidate | US categorical | Count | 89 | 4 | 93 |
|  |  | % within candidate | 95.7% | 4.3% | 100.0% |
|  |  | % of Total | 43.8% | 2.0% | 45.8% |
|  | IM categorical | Count | 46 | 10 | 56 |
|  |  | % within candidate | 82.1% | 17.9% | 100.0% |
|  |  | % of Total | 22.7% | 4.9% | 27.6% |
|  | Preliminary | Count | 53 | 1 | 54 |
|  |  | % within candidate | 98.1% | 1.9% | 100.0% |
|  |  | % of Total | 26.1% | 0.5% | 26.6% |
| Total | | Count | 188 | 15 | 203 |
|  |  | % within candidate | 92.6% | 7.4% | 100.0% |
|  |  | % of Total | 92.6% | 7.4% | 100.0% |

| **Chi-Square Tests** | | | |
| --- | --- | --- | --- |
|  | Value | df | Asymp. Sig. (2-sided) |
| Pearson Chi-Square | 12.683^a^ | 2 | .002 |
| Likelihood Ratio | 11.510 | 2 | .003 |
| Linear-by-Linear Association | .001 | 1 | .970 |
| N of Valid Cases | 203 |  |  |

| a. 2 cells (33.3%) have expected count less than 5. The minimum expected count is 3.99. |
| --- |

**candidate * Qbehavior_recode**

| **Crosstab** | | | | | |
| --- | --- | --- | --- | --- | --- |
|  | | | Qbehavior_recode | | Total |
|  |  |  | 1 | 2 |  |
| candidate | US categorical | Count | 64 | 2 | 66 |
|  |  | % within candidate | 97.0% | 3.0% | 100.0% |
|  |  | % of Total | 42.4% | 1.3% | 43.7% |
|  | IM categorical | Count | 41 | 5 | 46 |
|  |  | % within candidate | 89.1% | 10.9% | 100.0% |
|  |  | % of Total | 27.2% | 3.3% | 30.5% |
|  | Preliminary | Count | 34 | 5 | 39 |
|  |  | % within candidate | 87.2% | 12.8% | 100.0% |
|  |  | % of Total | 22.5% | 3.3% | 25.8% |
| Total | | Count | 139 | 12 | 151 |
|  |  | % within candidate | 92.1% | 7.9% | 100.0% |
|  |  | % of Total | 92.1% | 7.9% | 100.0% |

| **Chi-Square Tests** | | | |
| --- | --- | --- | --- |
|  | Value | df | Asymp. Sig. (2-sided) |
| Pearson Chi-Square | 3.984^a^ | 2 | .136 |
| Likelihood Ratio | 4.374 | 2 | .112 |
| Linear-by-Linear Association | 3.589 | 1 | .058 |
| N of Valid Cases | 151 |  |  |

| a. 2 cells (33.3%) have expected count less than 5. The minimum expected count is 3.10. |
| --- |

**candidate * Qsituation_recode**

| **Crosstab** | | | | | |
| --- | --- | --- | --- | --- | --- |
|  | | | Qsituation_recode | | Total |
|  |  |  | 1 | 2 |  |
| candidate | US categorical | Count | 32 | 8 | 40 |
|  |  | % within candidate | 80.0% | 20.0% | 100.0% |
|  |  | % of Total | 29.4% | 7.3% | 36.7% |
|  | IM categorical | Count | 34 | 9 | 43 |
|  |  | % within candidate | 79.1% | 20.9% | 100.0% |
|  |  | % of Total | 31.2% | 8.3% | 39.4% |
|  | Preliminary | Count | 15 | 11 | 26 |
|  |  | % within candidate | 57.7% | 42.3% | 100.0% |
|  |  | % of Total | 13.8% | 10.1% | 23.9% |
| Total | | Count | 81 | 28 | 109 |
|  |  | % within candidate | 74.3% | 25.7% | 100.0% |
|  |  | % of Total | 74.3% | 25.7% | 100.0% |

| **Chi-Square Tests** | | | |
| --- | --- | --- | --- |
|  | Value | df | Asymp. Sig. (2-sided) |
| Pearson Chi-Square | 4.950^a^ | 2 | .084 |
| Likelihood Ratio | 4.631 | 2 | .099 |
| Linear-by-Linear Association | 3.518 | 1 | .061 |
| N of Valid Cases | 109 |  |  |

| a. 0 cells (0.0%) have expected count less than 5. The minimum expected count is 6.68. |
| --- |

**candidate * Qteaser_recode**

| **Crosstab** | | | | | |
| --- | --- | --- | --- | --- | --- |
|  | | | Qteaser_recode | | Total |
|  |  |  | 1 | 2 |  |
| candidate | US categorical | Count | 6 | 87 | 93 |
|  |  | % within candidate | 6.5% | 93.5% | 100.0% |
|  |  | % of Total | 3.1% | 45.3% | 48.4% |
|  | IM categorical | Count | 7 | 46 | 53 |
|  |  | % within candidate | 13.2% | 86.8% | 100.0% |
|  |  | % of Total | 3.6% | 24.0% | 27.6% |
|  | Preliminary | Count | 6 | 40 | 46 |
|  |  | % within candidate | 13.0% | 87.0% | 100.0% |
|  |  | % of Total | 3.1% | 20.8% | 24.0% |
| Total | | Count | 19 | 173 | 192 |
|  |  | % within candidate | 9.9% | 90.1% | 100.0% |
|  |  | % of Total | 9.9% | 90.1% | 100.0% |

| **Chi-Square Tests** | | | |
| --- | --- | --- | --- |
|  | Value | df | Asymp. Sig. (2-sided) |
| Pearson Chi-Square | 2.400^a^ | 2 | .301 |
| Likelihood Ratio | 2.460 | 2 | .292 |
| Linear-by-Linear Association | 1.893 | 1 | .169 |
| N of Valid Cases | 192 |  |  |

| a. 1 cells (16.7%) have expected count less than 5. The minimum expected count is 4.55. |
| --- |

**candidate * Qmedical_recode**

| **Crosstab** | | | | | |
| --- | --- | --- | --- | --- | --- |
|  | | | Qmedical_recode | | Total |
|  |  |  | 1 | 2 |  |
| candidate | US categorical | Count | 1 | 91 | 92 |
|  |  | % within candidate | 1.1% | 98.9% | 100.0% |
|  |  | % of Total | 0.5% | 44.4% | 44.9% |
|  | IM categorical | Count | 2 | 60 | 62 |
|  |  | % within candidate | 3.2% | 96.8% | 100.0% |
|  |  | % of Total | 1.0% | 29.3% | 30.2% |
|  | Preliminary | Count | 0 | 51 | 51 |
|  |  | % within candidate | 0.0% | 100.0% | 100.0% |
|  |  | % of Total | 0.0% | 24.9% | 24.9% |
| Total | | Count | 3 | 202 | 205 |
|  |  | % within candidate | 1.5% | 98.5% | 100.0% |
|  |  | % of Total | 1.5% | 98.5% | 100.0% |

| **Chi-Square Tests** | | | |
| --- | --- | --- | --- |
|  | Value | df | Asymp. Sig. (2-sided) |
| Pearson Chi-Square | 2.183^a^ | 2 | .336 |
| Likelihood Ratio | 2.599 | 2 | .273 |
| Linear-by-Linear Association | .082 | 1 | .775 |
| N of Valid Cases | 205 |  |  |

| a. 3 cells (50.0%) have expected count less than 5. The minimum expected count is .75. |
| --- |

**candidate * ResIA_LT15_recode**

| **Crosstab** | | | | | |
| --- | --- | --- | --- | --- | --- |
|  | | | ResIA_LT15_recode | | Total |
|  |  |  | 1 | 2 |  |
| candidate | US categorical | Count | 1 | 88 | 89 |
|  |  | % within candidate | 1.1% | 98.9% | 100.0% |
|  |  | % of Total | 0.5% | 44.7% | 45.2% |
|  | IM categorical | Count | 8 | 52 | 60 |
|  |  | % within candidate | 13.3% | 86.7% | 100.0% |
|  |  | % of Total | 4.1% | 26.4% | 30.5% |
|  | Preliminary | Count | 4 | 44 | 48 |
|  |  | % within candidate | 8.3% | 91.7% | 100.0% |
|  |  | % of Total | 2.0% | 22.3% | 24.4% |
| Total | | Count | 13 | 184 | 197 |
|  |  | % within candidate | 6.6% | 93.4% | 100.0% |
|  |  | % of Total | 6.6% | 93.4% | 100.0% |

| **Chi-Square Tests** | | | |
| --- | --- | --- | --- |
|  | Value | df | Asymp. Sig. (2-sided) |
| Pearson Chi-Square | 8.978^a^ | 2 | .011 |
| Likelihood Ratio | 10.174 | 2 | .006 |
| Linear-by-Linear Association | 4.090 | 1 | .043 |
| N of Valid Cases | 197 |  |  |

| a. 2 cells (33.3%) have expected count less than 5. The minimum expected count is 3.17. |
| --- |

**candidate * ResIA15_30_recode**

| **Crosstab** | | | | | |
| --- | --- | --- | --- | --- | --- |
|  | | | ResIA15_30_recode | | Total |
|  |  |  | 1 | 2 |  |
| candidate | US categorical | Count | 28 | 60 | 88 |
|  |  | % within candidate | 31.8% | 68.2% | 100.0% |
|  |  | % of Total | 15.3% | 32.8% | 48.1% |
|  | IM categorical | Count | 21 | 32 | 53 |
|  |  | % within candidate | 39.6% | 60.4% | 100.0% |
|  |  | % of Total | 11.5% | 17.5% | 29.0% |
|  | Preliminary | Count | 21 | 21 | 42 |
|  |  | % within candidate | 50.0% | 50.0% | 100.0% |
|  |  | % of Total | 11.5% | 11.5% | 23.0% |
| Total | | Count | 70 | 113 | 183 |
|  |  | % within candidate | 38.3% | 61.7% | 100.0% |
|  |  | % of Total | 38.3% | 61.7% | 100.0% |

| **Chi-Square Tests** | | | |
| --- | --- | --- | --- |
|  | Value | df | Asymp. Sig. (2-sided) |
| Pearson Chi-Square | 4.039^a^ | 2 | .133 |
| Likelihood Ratio | 4.008 | 2 | .135 |
| Linear-by-Linear Association | 3.991 | 1 | .046 |
| N of Valid Cases | 183 |  |  |

| a. 0 cells (0.0%) have expected count less than 5. The minimum expected count is 16.07. |
| --- |

**candidate * ResIA30_45_recode**

| **Crosstab** | | | | |
| --- | --- | --- | --- | --- |
|  | | | ResIA30_45_recode | Total |
|  |  |  | 1 |  |
| candidate | US categorical | Count | 57 | 57 |
|  |  | % within candidate | 100.0% | 100.0% |
|  |  | % of Total | 41.6% | 41.6% |
|  | IM categorical | Count | 40 | 40 |
|  |  | % within candidate | 100.0% | 100.0% |
|  |  | % of Total | 29.2% | 29.2% |
|  | Preliminary | Count | 40 | 40 |
|  |  | % within candidate | 100.0% | 100.0% |
|  |  | % of Total | 29.2% | 29.2% |
| Total | | Count | 137 | 137 |
|  |  | % within candidate | 100.0% | 100.0% |
|  |  | % of Total | 100.0% | 100.0% |

| **Chi-Square Tests** | |
| --- | --- |
|  | Value |
| Pearson Chi-Square | .^a^ |
| N of Valid Cases | 137 |

| a. No statistics are computed because ResIA30_45_recode is a constant. |
| --- |

**candidate * ResIA45_60_recode**

| **Crosstab** | | | | | |
| --- | --- | --- | --- | --- | --- |
|  | | | ResIA45_60_recode | | Total |
|  |  |  | 1 | 2 |  |
| candidate | US categorical | Count | 69 | 7 | 76 |
|  |  | % within candidate | 90.8% | 9.2% | 100.0% |
|  |  | % of Total | 38.8% | 3.9% | 42.7% |
|  | IM categorical | Count | 45 | 14 | 59 |
|  |  | % within candidate | 76.3% | 23.7% | 100.0% |
|  |  | % of Total | 25.3% | 7.9% | 33.1% |
|  | Preliminary | Count | 33 | 10 | 43 |
|  |  | % within candidate | 76.7% | 23.3% | 100.0% |
|  |  | % of Total | 18.5% | 5.6% | 24.2% |
| Total | | Count | 147 | 31 | 178 |
|  |  | % within candidate | 82.6% | 17.4% | 100.0% |
|  |  | % of Total | 82.6% | 17.4% | 100.0% |

| **Chi-Square Tests** | | | |
| --- | --- | --- | --- |
|  | Value | df | Asymp. Sig. (2-sided) |
| Pearson Chi-Square | 6.212^a^ | 2 | .045 |
| Likelihood Ratio | 6.600 | 2 | .037 |
| Linear-by-Linear Association | 4.686 | 1 | .030 |
| N of Valid Cases | 178 |  |  |

| a. 0 cells (0.0%) have expected count less than 5. The minimum expected count is 7.49. |
| --- |

**candidate * ResIA_GT60_recode**

| **Crosstab** | | | | | |
| --- | --- | --- | --- | --- | --- |
|  | | | ResIA_GT60_recode | | Total |
|  |  |  | 1 | 2 |  |
| candidate | US categorical | Count | 37 | 37 | 74 |
|  |  | % within candidate | 50.0% | 50.0% | 100.0% |
|  |  | % of Total | 22.4% | 22.4% | 44.8% |
|  | IM categorical | Count | 15 | 33 | 48 |
|  |  | % within candidate | 31.3% | 68.8% | 100.0% |
|  |  | % of Total | 9.1% | 20.0% | 29.1% |
|  | Preliminary | Count | 10 | 33 | 43 |
|  |  | % within candidate | 23.3% | 76.7% | 100.0% |
|  |  | % of Total | 6.1% | 20.0% | 26.1% |
| Total | | Count | 62 | 103 | 165 |
|  |  | % within candidate | 37.6% | 62.4% | 100.0% |
|  |  | % of Total | 37.6% | 62.4% | 100.0% |

| **Chi-Square Tests** | | | |
| --- | --- | --- | --- |
|  | Value | df | Asymp. Sig. (2-sided) |
| Pearson Chi-Square | 9.448^a^ | 2 | .009 |
| Likelihood Ratio | 9.591 | 2 | .008 |
| Linear-by-Linear Association | 8.982 | 1 | .003 |
| N of Valid Cases | 165 |  |  |

| a. 0 cells (0.0%) have expected count less than 5. The minimum expected count is 16.16. |
| --- |

**candidate * october_recode**

| **Crosstab** | | | | | |
| --- | --- | --- | --- | --- | --- |
|  | | | october_recode | | Total |
|  |  |  | 1 | 2 |  |
| candidate | US categorical | Count | 31 | 40 | 71 |
|  |  | % within candidate | 43.7% | 56.3% | 100.0% |
|  |  | % of Total | 19.0% | 24.5% | 43.6% |
|  | IM categorical | Count | 20 | 31 | 51 |
|  |  | % within candidate | 39.2% | 60.8% | 100.0% |
|  |  | % of Total | 12.3% | 19.0% | 31.3% |
|  | Preliminary | Count | 24 | 17 | 41 |
|  |  | % within candidate | 58.5% | 41.5% | 100.0% |
|  |  | % of Total | 14.7% | 10.4% | 25.2% |
| Total | | Count | 75 | 88 | 163 |
|  |  | % within candidate | 46.0% | 54.0% | 100.0% |
|  |  | % of Total | 46.0% | 54.0% | 100.0% |

| **Chi-Square Tests** | | | |
| --- | --- | --- | --- |
|  | Value | df | Asymp. Sig. (2-sided) |
| Pearson Chi-Square | 3.695^a^ | 2 | .158 |
| Likelihood Ratio | 3.698 | 2 | .157 |
| Linear-by-Linear Association | 1.739 | 1 | .187 |
| N of Valid Cases | 163 |  |  |

| a. 0 cells (0.0%) have expected count less than 5. The minimum expected count is 18.87. |
| --- |

**candidate * november_recode**

| **Crosstab** | | | | | |
| --- | --- | --- | --- | --- | --- |
|  | | | november_recode | | Total |
|  |  |  | 1 | 2 |  |
| candidate | US categorical | Count | 92 | 2 | 94 |
|  |  | % within candidate | 97.9% | 2.1% | 100.0% |
|  |  | % of Total | 46.2% | 1.0% | 47.2% |
|  | IM categorical | Count | 55 | 3 | 58 |
|  |  | % within candidate | 94.8% | 5.2% | 100.0% |
|  |  | % of Total | 27.6% | 1.5% | 29.1% |
|  | Preliminary | Count | 41 | 6 | 47 |
|  |  | % within candidate | 87.2% | 12.8% | 100.0% |
|  |  | % of Total | 20.6% | 3.0% | 23.6% |
| Total | | Count | 188 | 11 | 199 |
|  |  | % within candidate | 94.5% | 5.5% | 100.0% |
|  |  | % of Total | 94.5% | 5.5% | 100.0% |

| **Chi-Square Tests** | | | |
| --- | --- | --- | --- |
|  | Value | df | Asymp. Sig. (2-sided) |
| Pearson Chi-Square | 6.810^a^ | 2 | .033 |
| Likelihood Ratio | 6.209 | 2 | .045 |
| Linear-by-Linear Association | 6.385 | 1 | .012 |
| N of Valid Cases | 199 |  |  |

| a. 2 cells (33.3%) have expected count less than 5. The minimum expected count is 2.60. |
| --- |

**candidate * december_recode**

| **Crosstab** | | | | | |
| --- | --- | --- | --- | --- | --- |
|  | | | december_recode | | Total |
|  |  |  | 1 | 2 |  |
| candidate | US categorical | Count | 63 | 1 | 64 |
|  |  | % within candidate | 98.4% | 1.6% | 100.0% |
|  |  | % of Total | 43.8% | 0.7% | 44.4% |
|  | IM categorical | Count | 44 | 2 | 46 |
|  |  | % within candidate | 95.7% | 4.3% | 100.0% |
|  |  | % of Total | 30.6% | 1.4% | 31.9% |
|  | Preliminary | Count | 29 | 5 | 34 |
|  |  | % within candidate | 85.3% | 14.7% | 100.0% |
|  |  | % of Total | 20.1% | 3.5% | 23.6% |
| Total | | Count | 136 | 8 | 144 |
|  |  | % within candidate | 94.4% | 5.6% | 100.0% |
|  |  | % of Total | 94.4% | 5.6% | 100.0% |

| **Chi-Square Tests** | | | |
| --- | --- | --- | --- |
|  | Value | df | Asymp. Sig. (2-sided) |
| Pearson Chi-Square | 7.498^a^ | 2 | .024 |
| Likelihood Ratio | 6.642 | 2 | .036 |
| Linear-by-Linear Association | 6.624 | 1 | .010 |
| N of Valid Cases | 144 |  |  |

| a. 3 cells (50.0%) have expected count less than 5. The minimum expected count is 1.89. |
| --- |

**candidate * january_recode**

| **Crosstab** | | | | | |
| --- | --- | --- | --- | --- | --- |
|  | | | january_recode | | Total |
|  |  |  | 1 | 2 |  |
| candidate | US categorical | Count | 4 | 55 | 59 |
|  |  | % within candidate | 6.8% | 93.2% | 100.0% |
|  |  | % of Total | 2.8% | 38.5% | 41.3% |
|  | IM categorical | Count | 10 | 32 | 42 |
|  |  | % within candidate | 23.8% | 76.2% | 100.0% |
|  |  | % of Total | 7.0% | 22.4% | 29.4% |
|  | Preliminary | Count | 11 | 31 | 42 |
|  |  | % within candidate | 26.2% | 73.8% | 100.0% |
|  |  | % of Total | 7.7% | 21.7% | 29.4% |
| Total | | Count | 25 | 118 | 143 |
|  |  | % within candidate | 17.5% | 82.5% | 100.0% |
|  |  | % of Total | 17.5% | 82.5% | 100.0% |

| **Chi-Square Tests** | | | |
| --- | --- | --- | --- |
|  | Value | df | Asymp. Sig. (2-sided) |
| Pearson Chi-Square | 8.058^a^ | 2 | .018 |
| Likelihood Ratio | 8.887 | 2 | .012 |
| Linear-by-Linear Association | 6.916 | 1 | .009 |
| N of Valid Cases | 143 |  |  |

| a. 0 cells (0.0%) have expected count less than 5. The minimum expected count is 7.34. |
| --- |

**candidate * february_recode**

| **Crosstab** | | | | | |
| --- | --- | --- | --- | --- | --- |
|  | | | february_recode | | Total |
|  |  |  | 1 | 2 |  |
| candidate | US categorical | Count | 2 | 94 | 96 |
|  |  | % within candidate | 2.1% | 97.9% | 100.0% |
|  |  | % of Total | 0.9% | 44.5% | 45.5% |
|  | IM categorical | Count | 1 | 62 | 63 |
|  |  | % within candidate | 1.6% | 98.4% | 100.0% |
|  |  | % of Total | 0.5% | 29.4% | 29.9% |
|  | Preliminary | Count | 3 | 49 | 52 |
|  |  | % within candidate | 5.8% | 94.2% | 100.0% |
|  |  | % of Total | 1.4% | 23.2% | 24.6% |
| Total | | Count | 6 | 205 | 211 |
|  |  | % within candidate | 2.8% | 97.2% | 100.0% |
|  |  | % of Total | 2.8% | 97.2% | 100.0% |

| **Chi-Square Tests** | | | |
| --- | --- | --- | --- |
|  | Value | df | Asymp. Sig. (2-sided) |
| Pearson Chi-Square | 2.172^a^ | 2 | .338 |
| Likelihood Ratio | 1.896 | 2 | .387 |
| Linear-by-Linear Association | 1.315 | 1 | .251 |
| N of Valid Cases | 211 |  |  |

| a. 3 cells (50.0%) have expected count less than 5. The minimum expected count is 1.48. |
| --- |

**candidate * overview_recode**

| **Crosstab** | | | | | |
| --- | --- | --- | --- | --- | --- |
|  | | | overview_recode | | Total |
|  |  |  | 1 | 2 |  |
| candidate | US categorical | Count | 70 | 8 | 78 |
|  |  | % within candidate | 89.7% | 10.3% | 100.0% |
|  |  | % of Total | 37.6% | 4.3% | 41.9% |
|  | IM categorical | Count | 56 | 8 | 64 |
|  |  | % within candidate | 87.5% | 12.5% | 100.0% |
|  |  | % of Total | 30.1% | 4.3% | 34.4% |
|  | Preliminary | Count | 42 | 2 | 44 |
|  |  | % within candidate | 95.5% | 4.5% | 100.0% |
|  |  | % of Total | 22.6% | 1.1% | 23.7% |
| Total | | Count | 168 | 18 | 186 |
|  |  | % within candidate | 90.3% | 9.7% | 100.0% |
|  |  | % of Total | 90.3% | 9.7% | 100.0% |

| **Chi-Square Tests** | | | |
| --- | --- | --- | --- |
|  | Value | df | Asymp. Sig. (2-sided) |
| Pearson Chi-Square | 1.939^a^ | 2 | .379 |
| Likelihood Ratio | 2.188 | 2 | .335 |
| Linear-by-Linear Association | .722 | 1 | .396 |
| N of Valid Cases | 186 |  |  |

| a. 1 cells (16.7%) have expected count less than 5. The minimum expected count is 4.26. |
| --- |

**candidate * interview_recode**

| **Crosstab** | | | | | |
| --- | --- | --- | --- | --- | --- |
|  | | | interview_recode | | Total |
|  |  |  | 1 | 2 |  |
| candidate | US categorical | Count | 73 | 12 | 85 |
|  |  | % within candidate | 85.9% | 14.1% | 100.0% |
|  |  | % of Total | 38.8% | 6.4% | 45.2% |
|  | IM categorical | Count | 55 | 2 | 57 |
|  |  | % within candidate | 96.5% | 3.5% | 100.0% |
|  |  | % of Total | 29.3% | 1.1% | 30.3% |
|  | Preliminary | Count | 44 | 2 | 46 |
|  |  | % within candidate | 95.7% | 4.3% | 100.0% |
|  |  | % of Total | 23.4% | 1.1% | 24.5% |
| Total | | Count | 172 | 16 | 188 |
|  |  | % within candidate | 91.5% | 8.5% | 100.0% |
|  |  | % of Total | 91.5% | 8.5% | 100.0% |

| **Chi-Square Tests** | | | |
| --- | --- | --- | --- |
|  | Value | df | Asymp. Sig. (2-sided) |
| Pearson Chi-Square | 6.287^a^ | 2 | .043 |
| Likelihood Ratio | 6.453 | 2 | .040 |
| Linear-by-Linear Association | 4.639 | 1 | .031 |
| N of Valid Cases | 188 |  |  |

| a. 2 cells (33.3%) have expected count less than 5. The minimum expected count is 3.91. |
| --- |

**candidate * tour_recode**

| **Crosstab** | | | | | |
| --- | --- | --- | --- | --- | --- |
|  | | | tour_recode | | Total |
|  |  |  | 1 | 2 |  |
| candidate | US categorical | Count | 4 | 78 | 82 |
|  |  | % within candidate | 4.9% | 95.1% | 100.0% |
|  |  | % of Total | 2.3% | 45.1% | 47.4% |
|  | IM categorical | Count | 2 | 42 | 44 |
|  |  | % within candidate | 4.5% | 95.5% | 100.0% |
|  |  | % of Total | 1.2% | 24.3% | 25.4% |
|  | Preliminary | Count | 1 | 46 | 47 |
|  |  | % within candidate | 2.1% | 97.9% | 100.0% |
|  |  | % of Total | 0.6% | 26.6% | 27.2% |
| Total | | Count | 7 | 166 | 173 |
|  |  | % within candidate | 4.0% | 96.0% | 100.0% |
|  |  | % of Total | 4.0% | 96.0% | 100.0% |

| **Chi-Square Tests** | | | |
| --- | --- | --- | --- |
|  | Value | df | Asymp. Sig. (2-sided) |
| Pearson Chi-Square | .620^a^ | 2 | .733 |
| Likelihood Ratio | .700 | 2 | .705 |
| Linear-by-Linear Association | .527 | 1 | .468 |
| N of Valid Cases | 173 |  |  |

| a. 3 cells (50.0%) have expected count less than 5. The minimum expected count is 1.78. |
| --- |

**candidate * morning_report_recode**

| **Crosstab** | | | | | |
| --- | --- | --- | --- | --- | --- |
|  | | | morning_report_recode | | Total |
|  |  |  | 1 | 2 |  |
| candidate | US categorical | Count | 12 | 67 | 79 |
|  |  | % within candidate | 15.2% | 84.8% | 100.0% |
|  |  | % of Total | 6.6% | 36.8% | 43.4% |
|  | IM categorical | Count | 7 | 46 | 53 |
|  |  | % within candidate | 13.2% | 86.8% | 100.0% |
|  |  | % of Total | 3.8% | 25.3% | 29.1% |
|  | Preliminary | Count | 1 | 49 | 50 |
|  |  | % within candidate | 2.0% | 98.0% | 100.0% |
|  |  | % of Total | 0.5% | 26.9% | 27.5% |
| Total | | Count | 20 | 162 | 182 |
|  |  | % within candidate | 11.0% | 89.0% | 100.0% |
|  |  | % of Total | 11.0% | 89.0% | 100.0% |

| **Chi-Square Tests** | | | |
| --- | --- | --- | --- |
|  | Value | df | Asymp. Sig. (2-sided) |
| Pearson Chi-Square | 5.822^a^ | 2 | .054 |
| Likelihood Ratio | 7.565 | 2 | .023 |
| Linear-by-Linear Association | 4.990 | 1 | .025 |
| N of Valid Cases | 182 |  |  |

| a. 0 cells (0.0%) have expected count less than 5. The minimum expected count is 5.49. |
| --- |

**candidate * lunch_recode**

| **Crosstab** | | | | | |
| --- | --- | --- | --- | --- | --- |
|  | | | lunch_recode | | Total |
|  |  |  | 1 | 2 |  |
| candidate | US categorical | Count | 33 | 27 | 60 |
|  |  | % within candidate | 55.0% | 45.0% | 100.0% |
|  |  | % of Total | 25.0% | 20.5% | 45.5% |
|  | IM categorical | Count | 10 | 33 | 43 |
|  |  | % within candidate | 23.3% | 76.7% | 100.0% |
|  |  | % of Total | 7.6% | 25.0% | 32.6% |
|  | Preliminary | Count | 20 | 9 | 29 |
|  |  | % within candidate | 69.0% | 31.0% | 100.0% |
|  |  | % of Total | 15.2% | 6.8% | 22.0% |
| Total | | Count | 63 | 69 | 132 |
|  |  | % within candidate | 47.7% | 52.3% | 100.0% |
|  |  | % of Total | 47.7% | 52.3% | 100.0% |

| **Chi-Square Tests** | | | |
| --- | --- | --- | --- |
|  | Value | df | Asymp. Sig. (2-sided) |
| Pearson Chi-Square | 16.837^a^ | 2 | .000 |
| Likelihood Ratio | 17.576 | 2 | .000 |
| Linear-by-Linear Association | .157 | 1 | .692 |
| N of Valid Cases | 132 |  |  |

| a. 0 cells (0.0%) have expected count less than 5. The minimum expected count is 13.84. |
| --- |

**CATEGORICAL VERSUS PRELIM RECODE**

**Cat_prelim * INT_phone_recode**

| **Crosstab** | | | | | |
| --- | --- | --- | --- | --- | --- |
|  | | | INT_phone_recode | | Total |
|  |  |  | preferred | not preferred |  |
| Cat_prelim | categorical | Count | 9 | 119 | 128 |
|  |  | % within Cat_prelim | 7.0% | 93.0% | 100.0% |
|  |  | % of Total | 5.5% | 72.6% | 78.0% |
|  | preliminary | Count | 13 | 23 | 36 |
|  |  | % within Cat_prelim | 36.1% | 63.9% | 100.0% |
|  |  | % of Total | 7.9% | 14.0% | 22.0% |
| Total | | Count | 22 | 142 | 164 |
|  |  | % within Cat_prelim | 13.4% | 86.6% | 100.0% |
|  |  | % of Total | 13.4% | 86.6% | 100.0% |

| **Chi-Square Tests** | | | | | |
| --- | --- | --- | --- | --- | --- |
|  | Value | df | Asymp. Sig. (2-sided) | Exact Sig. (2-sided) | Exact Sig. (1-sided) |
| Pearson Chi-Square | 20.456^a^ | 1 | .000 |  |  |
| Continuity Correction^b^ | 18.029 | 1 | .000 |  |  |
| Likelihood Ratio | 17.065 | 1 | .000 |  |  |
| Fisher's Exact Test |  |  |  | .000 | .000 |
| Linear-by-Linear Association | 20.332 | 1 | .000 |  |  |
| N of Valid Cases | 164 |  |  |  |  |

| a. 1 cells (25.0%) have expected count less than 5. The minimum expected count is 4.83. |
| --- |
| b. Computed only for a 2x2 table |

**Cat_prelim * INT_lunch_recode**

| **Crosstab** | | | | | |
| --- | --- | --- | --- | --- | --- |
|  | | | INT_lunch_recode | | Total |
|  |  |  | 1 | 2 |  |
| Cat_prelim | categorical | Count | 86 | 24 | 110 |
|  |  | % within Cat_prelim | 78.2% | 21.8% | 100.0% |
|  |  | % of Total | 59.3% | 16.6% | 75.9% |
|  | preliminary | Count | 26 | 9 | 35 |
|  |  | % within Cat_prelim | 74.3% | 25.7% | 100.0% |
|  |  | % of Total | 17.9% | 6.2% | 24.1% |
| Total | | Count | 112 | 33 | 145 |
|  |  | % within Cat_prelim | 77.2% | 22.8% | 100.0% |
|  |  | % of Total | 77.2% | 22.8% | 100.0% |

| **Chi-Square Tests** | | | | | |
| --- | --- | --- | --- | --- | --- |
|  | Value | df | Asymp. Sig. (2-sided) | Exact Sig. (2-sided) | Exact Sig. (1-sided) |
| Pearson Chi-Square | .229^a^ | 1 | .632 |  |  |
| Continuity Correction^b^ | .061 | 1 | .805 |  |  |
| Likelihood Ratio | .225 | 1 | .635 |  |  |
| Fisher's Exact Test |  |  |  | .648 | .395 |
| Linear-by-Linear Association | .228 | 1 | .633 |  |  |
| N of Valid Cases | 145 |  |  |  |  |

| a. 0 cells (0.0%) have expected count less than 5. The minimum expected count is 7.97. |
| --- |
| b. Computed only for a 2x2 table |

**Cat_prelim * INT_one_on_one_recode**

| **Crosstab** | | | | | |
| --- | --- | --- | --- | --- | --- |
|  | | | INT_one_on_one_recode | | Total |
|  |  |  | 1 | 2 |  |
| Cat_prelim | categorical | Count | 157 | 4 | 161 |
|  |  | % within Cat_prelim | 97.5% | 2.5% | 100.0% |
|  |  | % of Total | 73.0% | 1.9% | 74.9% |
|  | preliminary | Count | 54 | 0 | 54 |
|  |  | % within Cat_prelim | 100.0% | 0.0% | 100.0% |
|  |  | % of Total | 25.1% | 0.0% | 25.1% |
| Total | | Count | 211 | 4 | 215 |
|  |  | % within Cat_prelim | 98.1% | 1.9% | 100.0% |
|  |  | % of Total | 98.1% | 1.9% | 100.0% |

| **Chi-Square Tests** | | | | | |
| --- | --- | --- | --- | --- | --- |
|  | Value | df | Asymp. Sig. (2-sided) | Exact Sig. (2-sided) | Exact Sig. (1-sided) |
| Pearson Chi-Square | 1.367^a^ | 1 | .242 |  |  |
| Continuity Correction^b^ | .345 | 1 | .557 |  |  |
| Likelihood Ratio | 2.339 | 1 | .126 |  |  |
| Fisher's Exact Test |  |  |  | .574 | .311 |
| Linear-by-Linear Association | 1.361 | 1 | .243 |  |  |
| N of Valid Cases | 215 |  |  |  |  |

| a. 2 cells (50.0%) have expected count less than 5. The minimum expected count is 1.00. |
| --- |
| b. Computed only for a 2x2 table |

**Cat_prelim * INT_panel2_recode**

| **Crosstab** | | | | | |
| --- | --- | --- | --- | --- | --- |
|  | | | INT_panel2_recode | | Total |
|  |  |  | 1 | 2 |  |
| Cat_prelim | categorical | Count | 62 | 48 | 110 |
|  |  | % within Cat_prelim | 56.4% | 43.6% | 100.0% |
|  |  | % of Total | 40.3% | 31.2% | 71.4% |
|  | preliminary | Count | 15 | 29 | 44 |
|  |  | % within Cat_prelim | 34.1% | 65.9% | 100.0% |
|  |  | % of Total | 9.7% | 18.8% | 28.6% |
| Total | | Count | 77 | 77 | 154 |
|  |  | % within Cat_prelim | 50.0% | 50.0% | 100.0% |
|  |  | % of Total | 50.0% | 50.0% | 100.0% |

| **Chi-Square Tests** | | | | | |
| --- | --- | --- | --- | --- | --- |
|  | Value | df | Asymp. Sig. (2-sided) | Exact Sig. (2-sided) | Exact Sig. (1-sided) |
| Pearson Chi-Square | 6.236^a^ | 1 | .013 |  |  |
| Continuity Correction^b^ | 5.377 | 1 | .020 |  |  |
| Likelihood Ratio | 6.320 | 1 | .012 |  |  |
| Fisher's Exact Test |  |  |  | .020 | .010 |
| Linear-by-Linear Association | 6.196 | 1 | .013 |  |  |
| N of Valid Cases | 154 |  |  |  |  |

| a. 0 cells (0.0%) have expected count less than 5. The minimum expected count is 22.00. |
| --- |
| b. Computed only for a 2x2 table |

**Cat_prelim * INT_group_recode**

| **Crosstab** | | | | | |
| --- | --- | --- | --- | --- | --- |
|  | | | INT_group_recode | | Total |
|  |  |  | 1 | 2 |  |
| Cat_prelim | categorical | Count | 8 | 127 | 135 |
|  |  | % within Cat_prelim | 5.9% | 94.1% | 100.0% |
|  |  | % of Total | 4.4% | 69.8% | 74.2% |
|  | preliminary | Count | 0 | 47 | 47 |
|  |  | % within Cat_prelim | 0.0% | 100.0% | 100.0% |
|  |  | % of Total | 0.0% | 25.8% | 25.8% |
| Total | | Count | 8 | 174 | 182 |
|  |  | % within Cat_prelim | 4.4% | 95.6% | 100.0% |
|  |  | % of Total | 4.4% | 95.6% | 100.0% |

| **Chi-Square Tests** | | | | | |
| --- | --- | --- | --- | --- | --- |
|  | Value | df | Asymp. Sig. (2-sided) | Exact Sig. (2-sided) | Exact Sig. (1-sided) |
| Pearson Chi-Square | 2.913^a^ | 1 | .088 |  |  |
| Continuity Correction^b^ | 1.674 | 1 | .196 |  |  |
| Likelihood Ratio | 4.907 | 1 | .027 |  |  |
| Fisher's Exact Test |  |  |  | .115 | .087 |
| Linear-by-Linear Association | 2.897 | 1 | .089 |  |  |
| N of Valid Cases | 182 |  |  |  |  |

| a. 1 cells (25.0%) have expected count less than 5. The minimum expected count is 2.07. |
| --- |
| b. Computed only for a 2x2 table |

**Cat_prelim * start7_8_recode**

| **Crosstab** | | | | | |
| --- | --- | --- | --- | --- | --- |
|  | | | start7_8_recode | | Total |
|  |  |  | 1 | 2 |  |
| Cat_prelim | categorical | Count | 62 | 60 | 122 |
|  |  | % within Cat_prelim | 50.8% | 49.2% | 100.0% |
|  |  | % of Total | 39.0% | 37.7% | 76.7% |
|  | preliminary | Count | 15 | 22 | 37 |
|  |  | % within Cat_prelim | 40.5% | 59.5% | 100.0% |
|  |  | % of Total | 9.4% | 13.8% | 23.3% |
| Total | | Count | 77 | 82 | 159 |
|  |  | % within Cat_prelim | 48.4% | 51.6% | 100.0% |
|  |  | % of Total | 48.4% | 51.6% | 100.0% |

| **Chi-Square Tests** | | | | | |
| --- | --- | --- | --- | --- | --- |
|  | Value | df | Asymp. Sig. (2-sided) | Exact Sig. (2-sided) | Exact Sig. (1-sided) |
| Pearson Chi-Square | 1.201^a^ | 1 | .273 |  |  |
| Continuity Correction^b^ | .825 | 1 | .364 |  |  |
| Likelihood Ratio | 1.208 | 1 | .272 |  |  |
| Fisher's Exact Test |  |  |  | .348 | .182 |
| Linear-by-Linear Association | 1.194 | 1 | .275 |  |  |
| N of Valid Cases | 159 |  |  |  |  |

| a. 0 cells (0.0%) have expected count less than 5. The minimum expected count is 17.92. |
| --- |
| b. Computed only for a 2x2 table |

**Cat_prelim * start8_9_recode**

| **Crosstab** | | | | | |
| --- | --- | --- | --- | --- | --- |
|  | | | start8_9_recode | | Total |
|  |  |  | 1 | 2 |  |
| Cat_prelim | categorical | Count | 131 | 21 | 152 |
|  |  | % within Cat_prelim | 86.2% | 13.8% | 100.0% |
|  |  | % of Total | 65.2% | 10.4% | 75.6% |
|  | preliminary | Count | 45 | 4 | 49 |
|  |  | % within Cat_prelim | 91.8% | 8.2% | 100.0% |
|  |  | % of Total | 22.4% | 2.0% | 24.4% |
| Total | | Count | 176 | 25 | 201 |
|  |  | % within Cat_prelim | 87.6% | 12.4% | 100.0% |
|  |  | % of Total | 87.6% | 12.4% | 100.0% |

| **Chi-Square Tests** | | | | | |
| --- | --- | --- | --- | --- | --- |
|  | Value | df | Asymp. Sig. (2-sided) | Exact Sig. (2-sided) | Exact Sig. (1-sided) |
| Pearson Chi-Square | 1.087^a^ | 1 | .297 |  |  |
| Continuity Correction^b^ | .630 | 1 | .427 |  |  |
| Likelihood Ratio | 1.178 | 1 | .278 |  |  |
| Fisher's Exact Test |  |  |  | .455 | .218 |
| Linear-by-Linear Association | 1.082 | 1 | .298 |  |  |
| N of Valid Cases | 201 |  |  |  |  |

| a. 0 cells (0.0%) have expected count less than 5. The minimum expected count is 6.09. |
| --- |
| b. Computed only for a 2x2 table |

**Cat_prelim * start9_10_recode**

| **Crosstab** | | | | | |
| --- | --- | --- | --- | --- | --- |
|  | | | start9_10_recode | | Total |
|  |  |  | 1 | 2 |  |
| Cat_prelim | categorical | Count | 94 | 5 | 99 |
|  |  | % within Cat_prelim | 94.9% | 5.1% | 100.0% |
|  |  | % of Total | 67.6% | 3.6% | 71.2% |
|  | preliminary | Count | 38 | 2 | 40 |
|  |  | % within Cat_prelim | 95.0% | 5.0% | 100.0% |
|  |  | % of Total | 27.3% | 1.4% | 28.8% |
| Total | | Count | 132 | 7 | 139 |
|  |  | % within Cat_prelim | 95.0% | 5.0% | 100.0% |
|  |  | % of Total | 95.0% | 5.0% | 100.0% |

| **Chi-Square Tests** | | | | | |
| --- | --- | --- | --- | --- | --- |
|  | Value | df | Asymp. Sig. (2-sided) | Exact Sig. (2-sided) | Exact Sig. (1-sided) |
| Pearson Chi-Square | .000^a^ | 1 | .990 |  |  |
| Continuity Correction^b^ | .000 | 1 | 1.000 |  |  |
| Likelihood Ratio | .000 | 1 | .990 |  |  |
| Fisher's Exact Test |  |  |  | 1.000 | .677 |
| Linear-by-Linear Association | .000 | 1 | .990 |  |  |
| N of Valid Cases | 139 |  |  |  |  |

| a. 2 cells (50.0%) have expected count less than 5. The minimum expected count is 2.01. |
| --- |
| b. Computed only for a 2x2 table |

**Cat_prelim * start10_11_recode**

| **Crosstab** | | | | | |
| --- | --- | --- | --- | --- | --- |
|  | | | start10_11_recode | | Total |
|  |  |  | 1 | 2 |  |
| Cat_prelim | categorical | Count | 26 | 96 | 122 |
|  |  | % within Cat_prelim | 21.3% | 78.7% | 100.0% |
|  |  | % of Total | 16.0% | 59.3% | 75.3% |
|  | preliminary | Count | 8 | 32 | 40 |
|  |  | % within Cat_prelim | 20.0% | 80.0% | 100.0% |
|  |  | % of Total | 4.9% | 19.8% | 24.7% |
| Total | | Count | 34 | 128 | 162 |
|  |  | % within Cat_prelim | 21.0% | 79.0% | 100.0% |
|  |  | % of Total | 21.0% | 79.0% | 100.0% |

| **Chi-Square Tests** | | | | | |
| --- | --- | --- | --- | --- | --- |
|  | Value | df | Asymp. Sig. (2-sided) | Exact Sig. (2-sided) | Exact Sig. (1-sided) |
| Pearson Chi-Square | .031^a^ | 1 | .860 |  |  |
| Continuity Correction^b^ | .000 | 1 | 1.000 |  |  |
| Likelihood Ratio | .031 | 1 | .859 |  |  |
| Fisher's Exact Test |  |  |  | 1.000 | .528 |
| Linear-by-Linear Association | .031 | 1 | .860 |  |  |
| N of Valid Cases | 162 |  |  |  |  |

| a. 0 cells (0.0%) have expected count less than 5. The minimum expected count is 8.40. |
| --- |
| b. Computed only for a 2x2 table |

**Cat_prelim * start11_12_recode**

| **Crosstab** | | | | | |
| --- | --- | --- | --- | --- | --- |
|  | | | start11_12_recode | | Total |
|  |  |  | 1 | 2 |  |
| Cat_prelim | categorical | Count | 9 | 140 | 149 |
|  |  | % within Cat_prelim | 6.0% | 94.0% | 100.0% |
|  |  | % of Total | 4.5% | 70.4% | 74.9% |
|  | preliminary | Count | 2 | 48 | 50 |
|  |  | % within Cat_prelim | 4.0% | 96.0% | 100.0% |
|  |  | % of Total | 1.0% | 24.1% | 25.1% |
| Total | | Count | 11 | 188 | 199 |
|  |  | % within Cat_prelim | 5.5% | 94.5% | 100.0% |
|  |  | % of Total | 5.5% | 94.5% | 100.0% |

| **Chi-Square Tests** | | | | | |
| --- | --- | --- | --- | --- | --- |
|  | Value | df | Asymp. Sig. (2-sided) | Exact Sig. (2-sided) | Exact Sig. (1-sided) |
| Pearson Chi-Square | .298^a^ | 1 | .585 |  |  |
| Continuity Correction^b^ | .036 | 1 | .850 |  |  |
| Likelihood Ratio | .319 | 1 | .572 |  |  |
| Fisher's Exact Test |  |  |  | .734 | .447 |
| Linear-by-Linear Association | .297 | 1 | .586 |  |  |
| N of Valid Cases | 199 |  |  |  |  |

| a. 1 cells (25.0%) have expected count less than 5. The minimum expected count is 2.76. |
| --- |
| b. Computed only for a 2x2 table |

**Cat_prelim * lengthLT2_recode**

| **Crosstab** | | | | | |
| --- | --- | --- | --- | --- | --- |
|  | | | lengthLT2_recode | | Total |
|  |  |  | 1 | 2 |  |
| Cat_prelim | categorical | Count | 11 | 129 | 140 |
|  |  | % within Cat_prelim | 7.9% | 92.1% | 100.0% |
|  |  | % of Total | 6.3% | 73.3% | 79.5% |
|  | preliminary | Count | 10 | 26 | 36 |
|  |  | % within Cat_prelim | 27.8% | 72.2% | 100.0% |
|  |  | % of Total | 5.7% | 14.8% | 20.5% |
| Total | | Count | 21 | 155 | 176 |
|  |  | % within Cat_prelim | 11.9% | 88.1% | 100.0% |
|  |  | % of Total | 11.9% | 88.1% | 100.0% |

| **Chi-Square Tests** | | | | | |
| --- | --- | --- | --- | --- | --- |
|  | Value | df | Asymp. Sig. (2-sided) | Exact Sig. (2-sided) | Exact Sig. (1-sided) |
| Pearson Chi-Square | 10.814^a^ | 1 | .001 |  |  |
| Continuity Correction^b^ | 9.002 | 1 | .003 |  |  |
| Likelihood Ratio | 9.063 | 1 | .003 |  |  |
| Fisher's Exact Test |  |  |  | .003 | .003 |
| Linear-by-Linear Association | 10.753 | 1 | .001 |  |  |
| N of Valid Cases | 176 |  |  |  |  |

| a. 1 cells (25.0%) have expected count less than 5. The minimum expected count is 4.30. |
| --- |
| b. Computed only for a 2x2 table |

**Cat_prelim * length2_recode**

| **Crosstab** | | | | | |
| --- | --- | --- | --- | --- | --- |
|  | | | length2_recode | | Total |
|  |  |  | 1 | 2 |  |
| Cat_prelim | categorical | Count | 54 | 52 | 106 |
|  |  | % within Cat_prelim | 50.9% | 49.1% | 100.0% |
|  |  | % of Total | 37.0% | 35.6% | 72.6% |
|  | preliminary | Count | 38 | 2 | 40 |
|  |  | % within Cat_prelim | 95.0% | 5.0% | 100.0% |
|  |  | % of Total | 26.0% | 1.4% | 27.4% |
| Total | | Count | 92 | 54 | 146 |
|  |  | % within Cat_prelim | 63.0% | 37.0% | 100.0% |
|  |  | % of Total | 63.0% | 37.0% | 100.0% |

| **Chi-Square Tests** | | | | | |
| --- | --- | --- | --- | --- | --- |
|  | Value | df | Asymp. Sig. (2-sided) | Exact Sig. (2-sided) | Exact Sig. (1-sided) |
| Pearson Chi-Square | 24.186^a^ | 1 | .000 |  |  |
| Continuity Correction^b^ | 22.332 | 1 | .000 |  |  |
| Likelihood Ratio | 29.603 | 1 | .000 |  |  |
| Fisher's Exact Test |  |  |  | .000 | .000 |
| Linear-by-Linear Association | 24.020 | 1 | .000 |  |  |
| N of Valid Cases | 146 |  |  |  |  |

| a. 0 cells (0.0%) have expected count less than 5. The minimum expected count is 14.79. |
| --- |
| b. Computed only for a 2x2 table |

**Cat_prelim * length4_recode**

| **Crosstab** | | | | | |
| --- | --- | --- | --- | --- | --- |
|  | | | length4_recode | | Total |
|  |  |  | 1 | 2 |  |
| Cat_prelim | categorical | Count | 135 | 4 | 139 |
|  |  | % within Cat_prelim | 97.1% | 2.9% | 100.0% |
|  |  | % of Total | 74.2% | 2.2% | 76.4% |
|  | preliminary | Count | 43 | 0 | 43 |
|  |  | % within Cat_prelim | 100.0% | 0.0% | 100.0% |
|  |  | % of Total | 23.6% | 0.0% | 23.6% |
| Total | | Count | 178 | 4 | 182 |
|  |  | % within Cat_prelim | 97.8% | 2.2% | 100.0% |
|  |  | % of Total | 97.8% | 2.2% | 100.0% |

| **Chi-Square Tests** | | | | | |
| --- | --- | --- | --- | --- | --- |
|  | Value | df | Asymp. Sig. (2-sided) | Exact Sig. (2-sided) | Exact Sig. (1-sided) |
| Pearson Chi-Square | 1.265^a^ | 1 | .261 |  |  |
| Continuity Correction^b^ | .281 | 1 | .596 |  |  |
| Likelihood Ratio | 2.184 | 1 | .139 |  |  |
| Fisher's Exact Test |  |  |  | .574 | .337 |
| Linear-by-Linear Association | 1.258 | 1 | .262 |  |  |
| N of Valid Cases | 182 |  |  |  |  |

| a. 2 cells (50.0%) have expected count less than 5. The minimum expected count is .95. |
| --- |
| b. Computed only for a 2x2 table |

**Cat_prelim * length6_recode**

| **Crosstab** | | | | | |
| --- | --- | --- | --- | --- | --- |
|  | | | length6_recode | | Total |
|  |  |  | 1 | 2 |  |
| Cat_prelim | categorical | Count | 104 | 27 | 131 |
|  |  | % within Cat_prelim | 79.4% | 20.6% | 100.0% |
|  |  | % of Total | 59.4% | 15.4% | 74.9% |
|  | preliminary | Count | 16 | 28 | 44 |
|  |  | % within Cat_prelim | 36.4% | 63.6% | 100.0% |
|  |  | % of Total | 9.1% | 16.0% | 25.1% |
| Total | | Count | 120 | 55 | 175 |
|  |  | % within Cat_prelim | 68.6% | 31.4% | 100.0% |
|  |  | % of Total | 68.6% | 31.4% | 100.0% |

| **Chi-Square Tests** | | | | | |
| --- | --- | --- | --- | --- | --- |
|  | Value | df | Asymp. Sig. (2-sided) | Exact Sig. (2-sided) | Exact Sig. (1-sided) |
| Pearson Chi-Square | 28.293^a^ | 1 | .000 |  |  |
| Continuity Correction^b^ | 26.331 | 1 | .000 |  |  |
| Likelihood Ratio | 26.895 | 1 | .000 |  |  |
| Fisher's Exact Test |  |  |  | .000 | .000 |
| Linear-by-Linear Association | 28.131 | 1 | .000 |  |  |
| N of Valid Cases | 175 |  |  |  |  |

| a. 0 cells (0.0%) have expected count less than 5. The minimum expected count is 13.83. |
| --- |
| b. Computed only for a 2x2 table |

**Cat_prelim * length8_recode**

| **Crosstab** | | | | | |
| --- | --- | --- | --- | --- | --- |
|  | | | length8_recode | | Total |
|  |  |  | 1 | 2 |  |
| Cat_prelim | categorical | Count | 18 | 110 | 128 |
|  |  | % within Cat_prelim | 14.1% | 85.9% | 100.0% |
|  |  | % of Total | 9.9% | 60.8% | 70.7% |
|  | preliminary | Count | 1 | 52 | 53 |
|  |  | % within Cat_prelim | 1.9% | 98.1% | 100.0% |
|  |  | % of Total | 0.6% | 28.7% | 29.3% |
| Total | | Count | 19 | 162 | 181 |
|  |  | % within Cat_prelim | 10.5% | 89.5% | 100.0% |
|  |  | % of Total | 10.5% | 89.5% | 100.0% |

| **Chi-Square Tests** | | | | | |
| --- | --- | --- | --- | --- | --- |
|  | Value | df | Asymp. Sig. (2-sided) | Exact Sig. (2-sided) | Exact Sig. (1-sided) |
| Pearson Chi-Square | 5.914^a^ | 1 | .015 |  |  |
| Continuity Correction^b^ | 4.689 | 1 | .030 |  |  |
| Likelihood Ratio | 7.704 | 1 | .006 |  |  |
| Fisher's Exact Test |  |  |  | .015 | .009 |
| Linear-by-Linear Association | 5.881 | 1 | .015 |  |  |
| N of Valid Cases | 181 |  |  |  |  |

| a. 0 cells (0.0%) have expected count less than 5. The minimum expected count is 5.56. |
| --- |
| b. Computed only for a 2x2 table |

**Cat_prelim * faculty1_recode**

| **Crosstab** | | | | | |
| --- | --- | --- | --- | --- | --- |
|  | | | faculty1_recode | | Total |
|  |  |  | 1 | 2 |  |
| Cat_prelim | categorical | Count | 35 | 62 | 97 |
|  |  | % within Cat_prelim | 36.1% | 63.9% | 100.0% |
|  |  | % of Total | 26.1% | 46.3% | 72.4% |
|  | preliminary | Count | 14 | 23 | 37 |
|  |  | % within Cat_prelim | 37.8% | 62.2% | 100.0% |
|  |  | % of Total | 10.4% | 17.2% | 27.6% |
| Total | | Count | 49 | 85 | 134 |
|  |  | % within Cat_prelim | 36.6% | 63.4% | 100.0% |
|  |  | % of Total | 36.6% | 63.4% | 100.0% |

| **Chi-Square Tests** | | | | | |
| --- | --- | --- | --- | --- | --- |
|  | Value | df | Asymp. Sig. (2-sided) | Exact Sig. (2-sided) | Exact Sig. (1-sided) |
| Pearson Chi-Square | .036^a^ | 1 | .850 |  |  |
| Continuity Correction^b^ | .000 | 1 | 1.000 |  |  |
| Likelihood Ratio | .035 | 1 | .851 |  |  |
| Fisher's Exact Test |  |  |  | .844 | .502 |
| Linear-by-Linear Association | .035 | 1 | .851 |  |  |
| N of Valid Cases | 134 |  |  |  |  |

| a. 0 cells (0.0%) have expected count less than 5. The minimum expected count is 13.53. |
| --- |
| b. Computed only for a 2x2 table |

**Cat_prelim * faculty2_recode**

| **Crosstab** | | | | | |
| --- | --- | --- | --- | --- | --- |
|  | | | faculty2_recode | | Total |
|  |  |  | 1 | 2 |  |
| Cat_prelim | categorical | Count | 145 | 9 | 154 |
|  |  | % within Cat_prelim | 94.2% | 5.8% | 100.0% |
|  |  | % of Total | 70.4% | 4.4% | 74.8% |
|  | preliminary | Count | 50 | 2 | 52 |
|  |  | % within Cat_prelim | 96.2% | 3.8% | 100.0% |
|  |  | % of Total | 24.3% | 1.0% | 25.2% |
| Total | | Count | 195 | 11 | 206 |
|  |  | % within Cat_prelim | 94.7% | 5.3% | 100.0% |
|  |  | % of Total | 94.7% | 5.3% | 100.0% |

| **Chi-Square Tests** | | | | | |
| --- | --- | --- | --- | --- | --- |
|  | Value | df | Asymp. Sig. (2-sided) | Exact Sig. (2-sided) | Exact Sig. (1-sided) |
| Pearson Chi-Square | .307^a^ | 1 | .580 |  |  |
| Continuity Correction^b^ | .039 | 1 | .844 |  |  |
| Likelihood Ratio | .328 | 1 | .567 |  |  |
| Fisher's Exact Test |  |  |  | .734 | .444 |
| Linear-by-Linear Association | .306 | 1 | .580 |  |  |
| N of Valid Cases | 206 |  |  |  |  |

| a. 1 cells (25.0%) have expected count less than 5. The minimum expected count is 2.78. |
| --- |
| b. Computed only for a 2x2 table |

**Cat_prelim * faculty3_recode**

| **Crosstab** | | | | | |
| --- | --- | --- | --- | --- | --- |
|  | | | faculty3_recode | | Total |
|  |  |  | 1 | 2 |  |
| Cat_prelim | categorical | Count | 122 | 2 | 124 |
|  |  | % within Cat_prelim | 98.4% | 1.6% | 100.0% |
|  |  | % of Total | 74.4% | 1.2% | 75.6% |
|  | preliminary | Count | 40 | 0 | 40 |
|  |  | % within Cat_prelim | 100.0% | 0.0% | 100.0% |
|  |  | % of Total | 24.4% | 0.0% | 24.4% |
| Total | | Count | 162 | 2 | 164 |
|  |  | % within Cat_prelim | 98.8% | 1.2% | 100.0% |
|  |  | % of Total | 98.8% | 1.2% | 100.0% |

| **Chi-Square Tests** | | | | | |
| --- | --- | --- | --- | --- | --- |
|  | Value | df | Asymp. Sig. (2-sided) | Exact Sig. (2-sided) | Exact Sig. (1-sided) |
| Pearson Chi-Square | .653^a^ | 1 | .419 |  |  |
| Continuity Correction^b^ | .000 | 1 | 1.000 |  |  |
| Likelihood Ratio | 1.126 | 1 | .289 |  |  |
| Fisher's Exact Test |  |  |  | 1.000 | .571 |
| Linear-by-Linear Association | .649 | 1 | .420 |  |  |
| N of Valid Cases | 164 |  |  |  |  |

| a. 2 cells (50.0%) have expected count less than 5. The minimum expected count is .49. |
| --- |
| b. Computed only for a 2x2 table |

**Cat_prelim * faculty4_recode**

| **Crosstab** | | | | | |
| --- | --- | --- | --- | --- | --- |
|  | | | faculty4_recode | | Total |
|  |  |  | 1 | 2 |  |
| Cat_prelim | categorical | Count | 15 | 97 | 112 |
|  |  | % within Cat_prelim | 13.4% | 86.6% | 100.0% |
|  |  | % of Total | 10.2% | 66.0% | 76.2% |
|  | preliminary | Count | 4 | 31 | 35 |
|  |  | % within Cat_prelim | 11.4% | 88.6% | 100.0% |
|  |  | % of Total | 2.7% | 21.1% | 23.8% |
| Total | | Count | 19 | 128 | 147 |
|  |  | % within Cat_prelim | 12.9% | 87.1% | 100.0% |
|  |  | % of Total | 12.9% | 87.1% | 100.0% |

| **Chi-Square Tests** | | | | | |
| --- | --- | --- | --- | --- | --- |
|  | Value | df | Asymp. Sig. (2-sided) | Exact Sig. (2-sided) | Exact Sig. (1-sided) |
| Pearson Chi-Square | .091^a^ | 1 | .762 |  |  |
| Continuity Correction^b^ | .000 | 1 | .989 |  |  |
| Likelihood Ratio | .094 | 1 | .760 |  |  |
| Fisher's Exact Test |  |  |  | 1.000 | .510 |
| Linear-by-Linear Association | .091 | 1 | .763 |  |  |
| N of Valid Cases | 147 |  |  |  |  |

| a. 1 cells (25.0%) have expected count less than 5. The minimum expected count is 4.52. |
| --- |
| b. Computed only for a 2x2 table |

**Cat_prelim * facultyGT4_recode**

| **Crosstab** | | | | | |
| --- | --- | --- | --- | --- | --- |
|  | | | facultyGT4_recode | | Total |
|  |  |  | 1 | 2 |  |
| Cat_prelim | categorical | Count | 5 | 152 | 157 |
|  |  | % within Cat_prelim | 3.2% | 96.8% | 100.0% |
|  |  | % of Total | 2.4% | 72.7% | 75.1% |
|  | preliminary | Count | 0 | 52 | 52 |
|  |  | % within Cat_prelim | 0.0% | 100.0% | 100.0% |
|  |  | % of Total | 0.0% | 24.9% | 24.9% |
| Total | | Count | 5 | 204 | 209 |
|  |  | % within Cat_prelim | 2.4% | 97.6% | 100.0% |
|  |  | % of Total | 2.4% | 97.6% | 100.0% |

| **Chi-Square Tests** | | | | | |
| --- | --- | --- | --- | --- | --- |
|  | Value | df | Asymp. Sig. (2-sided) | Exact Sig. (2-sided) | Exact Sig. (1-sided) |
| Pearson Chi-Square | 1.697^a^ | 1 | .193 |  |  |
| Continuity Correction^b^ | .607 | 1 | .436 |  |  |
| Likelihood Ratio | 2.901 | 1 | .089 |  |  |
| Fisher's Exact Test |  |  |  | .335 | .235 |
| Linear-by-Linear Association | 1.689 | 1 | .194 |  |  |
| N of Valid Cases | 209 |  |  |  |  |

| a. 2 cells (50.0%) have expected count less than 5. The minimum expected count is 1.24. |
| --- |
| b. Computed only for a 2x2 table |

**Cat_prelim * lengthLT15_recode**

| **Crosstab** | | | | | |
| --- | --- | --- | --- | --- | --- |
|  | | | lengthLT15_recode | | Total |
|  |  |  | 1 | 2 |  |
| Cat_prelim | categorical | Count | 77 | 34 | 111 |
|  |  | % within Cat_prelim | 69.4% | 30.6% | 100.0% |
|  |  | % of Total | 49.7% | 21.9% | 71.6% |
|  | preliminary | Count | 43 | 1 | 44 |
|  |  | % within Cat_prelim | 97.7% | 2.3% | 100.0% |
|  |  | % of Total | 27.7% | 0.6% | 28.4% |
| Total | | Count | 120 | 35 | 155 |
|  |  | % within Cat_prelim | 77.4% | 22.6% | 100.0% |
|  |  | % of Total | 77.4% | 22.6% | 100.0% |

| **Chi-Square Tests** | | | | | |
| --- | --- | --- | --- | --- | --- |
|  | Value | df | Asymp. Sig. (2-sided) | Exact Sig. (2-sided) | Exact Sig. (1-sided) |
| Pearson Chi-Square | 14.495^a^ | 1 | .000 |  |  |
| Continuity Correction^b^ | 12.918 | 1 | .000 |  |  |
| Likelihood Ratio | 19.267 | 1 | .000 |  |  |
| Fisher's Exact Test |  |  |  | .000 | .000 |
| Linear-by-Linear Association | 14.401 | 1 | .000 |  |  |
| N of Valid Cases | 155 |  |  |  |  |

| a. 0 cells (0.0%) have expected count less than 5. The minimum expected count is 9.94. |
| --- |
| b. Computed only for a 2x2 table |

**Cat_prelim * length15_30_recode**

| **Crosstab** | | | | | |
| --- | --- | --- | --- | --- | --- |
|  | | | length15_30_recode | | Total |
|  |  |  | 1 | 2 |  |
| Cat_prelim | categorical | Count | 151 | 5 | 156 |
|  |  | % within Cat_prelim | 96.8% | 3.2% | 100.0% |
|  |  | % of Total | 71.9% | 2.4% | 74.3% |
|  | preliminary | Count | 54 | 0 | 54 |
|  |  | % within Cat_prelim | 100.0% | 0.0% | 100.0% |
|  |  | % of Total | 25.7% | 0.0% | 25.7% |
| Total | | Count | 205 | 5 | 210 |
|  |  | % within Cat_prelim | 97.6% | 2.4% | 100.0% |
|  |  | % of Total | 97.6% | 2.4% | 100.0% |

| **Chi-Square Tests** | | | | | |
| --- | --- | --- | --- | --- | --- |
|  | Value | df | Asymp. Sig. (2-sided) | Exact Sig. (2-sided) | Exact Sig. (1-sided) |
| Pearson Chi-Square | 1.773^a^ | 1 | .183 |  |  |
| Continuity Correction^b^ | .662 | 1 | .416 |  |  |
| Likelihood Ratio | 3.015 | 1 | .083 |  |  |
| Fisher's Exact Test |  |  |  | .331 | .222 |
| Linear-by-Linear Association | 1.765 | 1 | .184 |  |  |
| N of Valid Cases | 210 |  |  |  |  |

| a. 2 cells (50.0%) have expected count less than 5. The minimum expected count is 1.29. |
| --- |
| b. Computed only for a 2x2 table |

**Cat_prelim * length30_45_recode**

| **Crosstab** | | | | | |
| --- | --- | --- | --- | --- | --- |
|  | | | length30_45_recode | | Total |
|  |  |  | 1 | 2 |  |
| Cat_prelim | categorical | Count | 81 | 1 | 82 |
|  |  | % within Cat_prelim | 98.8% | 1.2% | 100.0% |
|  |  | % of Total | 87.1% | 1.1% | 88.2% |
|  | preliminary | Count | 11 | 0 | 11 |
|  |  | % within Cat_prelim | 100.0% | 0.0% | 100.0% |
|  |  | % of Total | 11.8% | 0.0% | 11.8% |
| Total | | Count | 92 | 1 | 93 |
|  |  | % within Cat_prelim | 98.9% | 1.1% | 100.0% |
|  |  | % of Total | 98.9% | 1.1% | 100.0% |

| **Chi-Square Tests** | | | | | |
| --- | --- | --- | --- | --- | --- |
|  | Value | df | Asymp. Sig. (2-sided) | Exact Sig. (2-sided) | Exact Sig. (1-sided) |
| Pearson Chi-Square | .136^a^ | 1 | .713 |  |  |
| Continuity Correction^b^ | .000 | 1 | 1.000 |  |  |
| Likelihood Ratio | .253 | 1 | .615 |  |  |
| Fisher's Exact Test |  |  |  | 1.000 | .882 |
| Linear-by-Linear Association | .134 | 1 | .714 |  |  |
| N of Valid Cases | 93 |  |  |  |  |

| a. 2 cells (50.0%) have expected count less than 5. The minimum expected count is .12. |
| --- |
| b. Computed only for a 2x2 table |

**Cat_prelim * length45_60_recode**

| **Crosstab** | | | | | |
| --- | --- | --- | --- | --- | --- |
|  | | | length45_60_recode | | Total |
|  |  |  | 1 | 2 |  |
| Cat_prelim | categorical | Count | 9 | 125 | 134 |
|  |  | % within Cat_prelim | 6.7% | 93.3% | 100.0% |
|  |  | % of Total | 4.8% | 66.8% | 71.7% |
|  | preliminary | Count | 0 | 53 | 53 |
|  |  | % within Cat_prelim | 0.0% | 100.0% | 100.0% |
|  |  | % of Total | 0.0% | 28.3% | 28.3% |
| Total | | Count | 9 | 178 | 187 |
|  |  | % within Cat_prelim | 4.8% | 95.2% | 100.0% |
|  |  | % of Total | 4.8% | 95.2% | 100.0% |

| **Chi-Square Tests** | | | | | |
| --- | --- | --- | --- | --- | --- |
|  | Value | df | Asymp. Sig. (2-sided) | Exact Sig. (2-sided) | Exact Sig. (1-sided) |
| Pearson Chi-Square | 3.740^a^ | 1 | .053 |  |  |
| Continuity Correction^b^ | 2.417 | 1 | .120 |  |  |
| Likelihood Ratio | 6.177 | 1 | .013 |  |  |
| Fisher's Exact Test |  |  |  | .063 | .046 |
| Linear-by-Linear Association | 3.720 | 1 | .054 |  |  |
| N of Valid Cases | 187 |  |  |  |  |

| a. 1 cells (25.0%) have expected count less than 5. The minimum expected count is 2.55. |
| --- |
| b. Computed only for a 2x2 table |

**Cat_prelim * lengthGT60_recode**

| **Crosstab** | | | | | |
| --- | --- | --- | --- | --- | --- |
|  | | | lengthGT60_recode | | Total |
|  |  |  | 1 | 2 |  |
| Cat_prelim | categorical | Count | 5 | 156 | 161 |
|  |  | % within Cat_prelim | 3.1% | 96.9% | 100.0% |
|  |  | % of Total | 2.3% | 72.6% | 74.9% |
|  | preliminary | Count | 0 | 54 | 54 |
|  |  | % within Cat_prelim | 0.0% | 100.0% | 100.0% |
|  |  | % of Total | 0.0% | 25.1% | 25.1% |
| Total | | Count | 5 | 210 | 215 |
|  |  | % within Cat_prelim | 2.3% | 97.7% | 100.0% |
|  |  | % of Total | 2.3% | 97.7% | 100.0% |

| **Chi-Square Tests** | | | | | |
| --- | --- | --- | --- | --- | --- |
|  | Value | df | Asymp. Sig. (2-sided) | Exact Sig. (2-sided) | Exact Sig. (1-sided) |
| Pearson Chi-Square | 1.717^a^ | 1 | .190 |  |  |
| Continuity Correction^b^ | .622 | 1 | .430 |  |  |
| Likelihood Ratio | 2.932 | 1 | .087 |  |  |
| Fisher's Exact Test |  |  |  | .334 | .232 |
| Linear-by-Linear Association | 1.709 | 1 | .191 |  |  |
| N of Valid Cases | 215 |  |  |  |  |

| a. 2 cells (50.0%) have expected count less than 5. The minimum expected count is 1.26. |
| --- |
| b. Computed only for a 2x2 table |

**Cat_prelim * INT_res_chief_recode**

| **Crosstab** | | | | | |
| --- | --- | --- | --- | --- | --- |
|  | | | INT_res_chief_recode | | Total |
|  |  |  | 1 | 2 |  |
| Cat_prelim | categorical | Count | 23 | 117 | 140 |
|  |  | % within Cat_prelim | 16.4% | 83.6% | 100.0% |
|  |  | % of Total | 12.6% | 64.3% | 76.9% |
|  | preliminary | Count | 5 | 37 | 42 |
|  |  | % within Cat_prelim | 11.9% | 88.1% | 100.0% |
|  |  | % of Total | 2.7% | 20.3% | 23.1% |
| Total | | Count | 28 | 154 | 182 |
|  |  | % within Cat_prelim | 15.4% | 84.6% | 100.0% |
|  |  | % of Total | 15.4% | 84.6% | 100.0% |

| **Chi-Square Tests** | | | | | |
| --- | --- | --- | --- | --- | --- |
|  | Value | df | Asymp. Sig. (2-sided) | Exact Sig. (2-sided) | Exact Sig. (1-sided) |
| Pearson Chi-Square | .508^a^ | 1 | .476 |  |  |
| Continuity Correction^b^ | .220 | 1 | .639 |  |  |
| Likelihood Ratio | .533 | 1 | .465 |  |  |
| Fisher's Exact Test |  |  |  | .628 | .329 |
| Linear-by-Linear Association | .505 | 1 | .477 |  |  |
| N of Valid Cases | 182 |  |  |  |  |

| a. 0 cells (0.0%) have expected count less than 5. The minimum expected count is 6.46. |
| --- |
| b. Computed only for a 2x2 table |

**Cat_prelim * INT_faculty_recode**

| **Crosstab** | | | | | |
| --- | --- | --- | --- | --- | --- |
|  | | | INT_faculty_recode | | Total |
|  |  |  | 1 | 2 |  |
| Cat_prelim | categorical | Count | 31 | 74 | 105 |
|  |  | % within Cat_prelim | 29.5% | 70.5% | 100.0% |
|  |  | % of Total | 21.5% | 51.4% | 72.9% |
|  | preliminary | Count | 13 | 26 | 39 |
|  |  | % within Cat_prelim | 33.3% | 66.7% | 100.0% |
|  |  | % of Total | 9.0% | 18.1% | 27.1% |
| Total | | Count | 44 | 100 | 144 |
|  |  | % within Cat_prelim | 30.6% | 69.4% | 100.0% |
|  |  | % of Total | 30.6% | 69.4% | 100.0% |

| **Chi-Square Tests** | | | | | |
| --- | --- | --- | --- | --- | --- |
|  | Value | df | Asymp. Sig. (2-sided) | Exact Sig. (2-sided) | Exact Sig. (1-sided) |
| Pearson Chi-Square | .194^a^ | 1 | .659 |  |  |
| Continuity Correction^b^ | .056 | 1 | .812 |  |  |
| Likelihood Ratio | .193 | 1 | .661 |  |  |
| Fisher's Exact Test |  |  |  | .687 | .402 |
| Linear-by-Linear Association | .193 | 1 | .660 |  |  |
| N of Valid Cases | 144 |  |  |  |  |

| a. 0 cells (0.0%) have expected count less than 5. The minimum expected count is 11.92. |
| --- |
| b. Computed only for a 2x2 table |

**Cat_prelim * INT_APD_recode**

| **Crosstab** | | | | | |
| --- | --- | --- | --- | --- | --- |
|  | | | INT_APD_recode | | Total |
|  |  |  | 1 | 2 |  |
| Cat_prelim | categorical | Count | 101 | 12 | 113 |
|  |  | % within Cat_prelim | 89.4% | 10.6% | 100.0% |
|  |  | % of Total | 65.2% | 7.7% | 72.9% |
|  | preliminary | Count | 37 | 5 | 42 |
|  |  | % within Cat_prelim | 88.1% | 11.9% | 100.0% |
|  |  | % of Total | 23.9% | 3.2% | 27.1% |
| Total | | Count | 138 | 17 | 155 |
|  |  | % within Cat_prelim | 89.0% | 11.0% | 100.0% |
|  |  | % of Total | 89.0% | 11.0% | 100.0% |

| **Chi-Square Tests** | | | | | |
| --- | --- | --- | --- | --- | --- |
|  | Value | df | Asymp. Sig. (2-sided) | Exact Sig. (2-sided) | Exact Sig. (1-sided) |
| Pearson Chi-Square | .052^a^ | 1 | .820 |  |  |
| Continuity Correction^b^ | .000 | 1 | 1.000 |  |  |
| Likelihood Ratio | .051 | 1 | .821 |  |  |
| Fisher's Exact Test |  |  |  | .779 | .510 |
| Linear-by-Linear Association | .051 | 1 | .821 |  |  |
| N of Valid Cases | 155 |  |  |  |  |

| a. 1 cells (25.0%) have expected count less than 5. The minimum expected count is 4.61. |
| --- |
| b. Computed only for a 2x2 table |

**Cat_prelim * INT_PD_recode**

| **Crosstab** | | | | | |
| --- | --- | --- | --- | --- | --- |
|  | | | INT_PD_recode | | Total |
|  |  |  | 1 | 2 |  |
| Cat_prelim | categorical | Count | 140 | 9 | 149 |
|  |  | % within Cat_prelim | 94.0% | 6.0% | 100.0% |
|  |  | % of Total | 71.4% | 4.6% | 76.0% |
|  | preliminary | Count | 46 | 1 | 47 |
|  |  | % within Cat_prelim | 97.9% | 2.1% | 100.0% |
|  |  | % of Total | 23.5% | 0.5% | 24.0% |
| Total | | Count | 186 | 10 | 196 |
|  |  | % within Cat_prelim | 94.9% | 5.1% | 100.0% |
|  |  | % of Total | 94.9% | 5.1% | 100.0% |

| **Chi-Square Tests** | | | | | |
| --- | --- | --- | --- | --- | --- |
|  | Value | df | Asymp. Sig. (2-sided) | Exact Sig. (2-sided) | Exact Sig. (1-sided) |
| Pearson Chi-Square | 1.130^a^ | 1 | .288 |  |  |
| Continuity Correction^b^ | .466 | 1 | .495 |  |  |
| Likelihood Ratio | 1.347 | 1 | .246 |  |  |
| Fisher's Exact Test |  |  |  | .456 | .261 |
| Linear-by-Linear Association | 1.124 | 1 | .289 |  |  |
| N of Valid Cases | 196 |  |  |  |  |

| a. 1 cells (25.0%) have expected count less than 5. The minimum expected count is 2.40. |
| --- |
| b. Computed only for a 2x2 table |

**Cat_prelim * INT_chair_recode**

| **Crosstab** | | | | | |
| --- | --- | --- | --- | --- | --- |
|  | | | INT_chair_recode | | Total |
|  |  |  | 1 | 2 |  |
| Cat_prelim | categorical | Count | 27 | 110 | 137 |
|  |  | % within Cat_prelim | 19.7% | 80.3% | 100.0% |
|  |  | % of Total | 14.8% | 60.1% | 74.9% |
|  | preliminary | Count | 7 | 39 | 46 |
|  |  | % within Cat_prelim | 15.2% | 84.8% | 100.0% |
|  |  | % of Total | 3.8% | 21.3% | 25.1% |
| Total | | Count | 34 | 149 | 183 |
|  |  | % within Cat_prelim | 18.6% | 81.4% | 100.0% |
|  |  | % of Total | 18.6% | 81.4% | 100.0% |

| **Chi-Square Tests** | | | | | |
| --- | --- | --- | --- | --- | --- |
|  | Value | df | Asymp. Sig. (2-sided) | Exact Sig. (2-sided) | Exact Sig. (1-sided) |
| Pearson Chi-Square | .459^a^ | 1 | .498 |  |  |
| Continuity Correction^b^ | .210 | 1 | .647 |  |  |
| Likelihood Ratio | .475 | 1 | .491 |  |  |
| Fisher's Exact Test |  |  |  | .662 | .330 |
| Linear-by-Linear Association | .457 | 1 | .499 |  |  |
| N of Valid Cases | 183 |  |  |  |  |

| a. 0 cells (0.0%) have expected count less than 5. The minimum expected count is 8.55. |
| --- |
| b. Computed only for a 2x2 table |

**Cat_prelim * Qstraightforward_recode**

| **Crosstab** | | | | | |
| --- | --- | --- | --- | --- | --- |
|  | | | Qstraightforward_recode | | Total |
|  |  |  | 1 | 2 |  |
| Cat_prelim | categorical | Count | 135 | 14 | 149 |
|  |  | % within Cat_prelim | 90.6% | 9.4% | 100.0% |
|  |  | % of Total | 66.5% | 6.9% | 73.4% |
|  | preliminary | Count | 53 | 1 | 54 |
|  |  | % within Cat_prelim | 98.1% | 1.9% | 100.0% |
|  |  | % of Total | 26.1% | 0.5% | 26.6% |
| Total | | Count | 188 | 15 | 203 |
|  |  | % within Cat_prelim | 92.6% | 7.4% | 100.0% |
|  |  | % of Total | 92.6% | 7.4% | 100.0% |

| **Chi-Square Tests** | | | | | |
| --- | --- | --- | --- | --- | --- |
|  | Value | df | Asymp. Sig. (2-sided) | Exact Sig. (2-sided) | Exact Sig. (1-sided) |
| Pearson Chi-Square | 3.296^a^ | 1 | .069 |  |  |
| Continuity Correction^b^ | 2.286 | 1 | .131 |  |  |
| Likelihood Ratio | 4.200 | 1 | .040 |  |  |
| Fisher's Exact Test |  |  |  | .076 | .055 |
| Linear-by-Linear Association | 3.280 | 1 | .070 |  |  |
| N of Valid Cases | 203 |  |  |  |  |

| a. 1 cells (25.0%) have expected count less than 5. The minimum expected count is 3.99. |
| --- |
| b. Computed only for a 2x2 table |

**Cat_prelim * Qbehavior_recode**

| **Crosstab** | | | | | |
| --- | --- | --- | --- | --- | --- |
|  | | | Qbehavior_recode | | Total |
|  |  |  | 1 | 2 |  |
| Cat_prelim | categorical | Count | 105 | 7 | 112 |
|  |  | % within Cat_prelim | 93.8% | 6.3% | 100.0% |
|  |  | % of Total | 69.5% | 4.6% | 74.2% |
|  | preliminary | Count | 34 | 5 | 39 |
|  |  | % within Cat_prelim | 87.2% | 12.8% | 100.0% |
|  |  | % of Total | 22.5% | 3.3% | 25.8% |
| Total | | Count | 139 | 12 | 151 |
|  |  | % within Cat_prelim | 92.1% | 7.9% | 100.0% |
|  |  | % of Total | 92.1% | 7.9% | 100.0% |

| **Chi-Square Tests** | | | | | |
| --- | --- | --- | --- | --- | --- |
|  | Value | df | Asymp. Sig. (2-sided) | Exact Sig. (2-sided) | Exact Sig. (1-sided) |
| Pearson Chi-Square | 1.707^a^ | 1 | .191 |  |  |
| Continuity Correction^b^ | .927 | 1 | .336 |  |  |
| Likelihood Ratio | 1.557 | 1 | .212 |  |  |
| Fisher's Exact Test |  |  |  | .299 | .166 |
| Linear-by-Linear Association | 1.696 | 1 | .193 |  |  |
| N of Valid Cases | 151 |  |  |  |  |

| a. 1 cells (25.0%) have expected count less than 5. The minimum expected count is 3.10. |
| --- |
| b. Computed only for a 2x2 table |

**Cat_prelim * Qsituation_recode**

| **Crosstab** | | | | | |
| --- | --- | --- | --- | --- | --- |
|  | | | Qsituation_recode | | Total |
|  |  |  | 1 | 2 |  |
| Cat_prelim | categorical | Count | 66 | 17 | 83 |
|  |  | % within Cat_prelim | 79.5% | 20.5% | 100.0% |
|  |  | % of Total | 60.6% | 15.6% | 76.1% |
|  | preliminary | Count | 15 | 11 | 26 |
|  |  | % within Cat_prelim | 57.7% | 42.3% | 100.0% |
|  |  | % of Total | 13.8% | 10.1% | 23.9% |
| Total | | Count | 81 | 28 | 109 |
|  |  | % within Cat_prelim | 74.3% | 25.7% | 100.0% |
|  |  | % of Total | 74.3% | 25.7% | 100.0% |

| **Chi-Square Tests** | | | | | |
| --- | --- | --- | --- | --- | --- |
|  | Value | df | Asymp. Sig. (2-sided) | Exact Sig. (2-sided) | Exact Sig. (1-sided) |
| Pearson Chi-Square | 4.941^a^ | 1 | .026 |  |  |
| Continuity Correction^b^ | 3.863 | 1 | .049 |  |  |
| Likelihood Ratio | 4.620 | 1 | .032 |  |  |
| Fisher's Exact Test |  |  |  | .039 | .027 |
| Linear-by-Linear Association | 4.895 | 1 | .027 |  |  |
| N of Valid Cases | 109 |  |  |  |  |

| a. 0 cells (0.0%) have expected count less than 5. The minimum expected count is 6.68. |
| --- |
| b. Computed only for a 2x2 table |

**Cat_prelim * Qteaser_recode**

| **Crosstab** | | | | | |
| --- | --- | --- | --- | --- | --- |
|  | | | Qteaser_recode | | Total |
|  |  |  | 1 | 2 |  |
| Cat_prelim | categorical | Count | 13 | 133 | 146 |
|  |  | % within Cat_prelim | 8.9% | 91.1% | 100.0% |
|  |  | % of Total | 6.8% | 69.3% | 76.0% |
|  | preliminary | Count | 6 | 40 | 46 |
|  |  | % within Cat_prelim | 13.0% | 87.0% | 100.0% |
|  |  | % of Total | 3.1% | 20.8% | 24.0% |
| Total | | Count | 19 | 173 | 192 |
|  |  | % within Cat_prelim | 9.9% | 90.1% | 100.0% |
|  |  | % of Total | 9.9% | 90.1% | 100.0% |

| **Chi-Square Tests** | | | | | |
| --- | --- | --- | --- | --- | --- |
|  | Value | df | Asymp. Sig. (2-sided) | Exact Sig. (2-sided) | Exact Sig. (1-sided) |
| Pearson Chi-Square | .672^a^ | 1 | .412 |  |  |
| Continuity Correction^b^ | .288 | 1 | .591 |  |  |
| Likelihood Ratio | .636 | 1 | .425 |  |  |
| Fisher's Exact Test |  |  |  | .405 | .286 |
| Linear-by-Linear Association | .669 | 1 | .414 |  |  |
| N of Valid Cases | 192 |  |  |  |  |

| a. 1 cells (25.0%) have expected count less than 5. The minimum expected count is 4.55. |
| --- |
| b. Computed only for a 2x2 table |

**Cat_prelim * Qmedical_recode**

| **Crosstab** | | | | | |
| --- | --- | --- | --- | --- | --- |
|  | | | Qmedical_recode | | Total |
|  |  |  | 1 | 2 |  |
| Cat_prelim | categorical | Count | 3 | 151 | 154 |
|  |  | % within Cat_prelim | 1.9% | 98.1% | 100.0% |
|  |  | % of Total | 1.5% | 73.7% | 75.1% |
|  | preliminary | Count | 0 | 51 | 51 |
|  |  | % within Cat_prelim | 0.0% | 100.0% | 100.0% |
|  |  | % of Total | 0.0% | 24.9% | 24.9% |
| Total | | Count | 3 | 202 | 205 |
|  |  | % within Cat_prelim | 1.5% | 98.5% | 100.0% |
|  |  | % of Total | 1.5% | 98.5% | 100.0% |

| **Chi-Square Tests** | | | | | |
| --- | --- | --- | --- | --- | --- |
|  | Value | df | Asymp. Sig. (2-sided) | Exact Sig. (2-sided) | Exact Sig. (1-sided) |
| Pearson Chi-Square | 1.008^a^ | 1 | .315 |  |  |
| Continuity Correction^b^ | .110 | 1 | .740 |  |  |
| Likelihood Ratio | 1.731 | 1 | .188 |  |  |
| Fisher's Exact Test |  |  |  | .575 | .422 |
| Linear-by-Linear Association | 1.003 | 1 | .317 |  |  |
| N of Valid Cases | 205 |  |  |  |  |

| a. 2 cells (50.0%) have expected count less than 5. The minimum expected count is .75. |
| --- |
| b. Computed only for a 2x2 table |

**Cat_prelim * ResIA_LT15_recode**

| **Crosstab** | | | | | |
| --- | --- | --- | --- | --- | --- |
|  | | | ResIA_LT15_recode | | Total |
|  |  |  | 1 | 2 |  |
| Cat_prelim | categorical | Count | 9 | 140 | 149 |
|  |  | % within Cat_prelim | 6.0% | 94.0% | 100.0% |
|  |  | % of Total | 4.6% | 71.1% | 75.6% |
|  | preliminary | Count | 4 | 44 | 48 |
|  |  | % within Cat_prelim | 8.3% | 91.7% | 100.0% |
|  |  | % of Total | 2.0% | 22.3% | 24.4% |
| Total | | Count | 13 | 184 | 197 |
|  |  | % within Cat_prelim | 6.6% | 93.4% | 100.0% |
|  |  | % of Total | 6.6% | 93.4% | 100.0% |

| **Chi-Square Tests** | | | | | |
| --- | --- | --- | --- | --- | --- |
|  | Value | df | Asymp. Sig. (2-sided) | Exact Sig. (2-sided) | Exact Sig. (1-sided) |
| Pearson Chi-Square | .310^a^ | 1 | .578 |  |  |
| Continuity Correction^b^ | .049 | 1 | .824 |  |  |
| Likelihood Ratio | .295 | 1 | .587 |  |  |
| Fisher's Exact Test |  |  |  | .522 | .394 |
| Linear-by-Linear Association | .308 | 1 | .579 |  |  |
| N of Valid Cases | 197 |  |  |  |  |

| a. 1 cells (25.0%) have expected count less than 5. The minimum expected count is 3.17. |
| --- |
| b. Computed only for a 2x2 table |

**Cat_prelim * ResIA15_30_recode**

| **Crosstab** | | | | | |
| --- | --- | --- | --- | --- | --- |
|  | | | ResIA15_30_recode | | Total |
|  |  |  | 1 | 2 |  |
| Cat_prelim | categorical | Count | 49 | 92 | 141 |
|  |  | % within Cat_prelim | 34.8% | 65.2% | 100.0% |
|  |  | % of Total | 26.8% | 50.3% | 77.0% |
|  | preliminary | Count | 21 | 21 | 42 |
|  |  | % within Cat_prelim | 50.0% | 50.0% | 100.0% |
|  |  | % of Total | 11.5% | 11.5% | 23.0% |
| Total | | Count | 70 | 113 | 183 |
|  |  | % within Cat_prelim | 38.3% | 61.7% | 100.0% |
|  |  | % of Total | 38.3% | 61.7% | 100.0% |

| **Chi-Square Tests** | | | | | |
| --- | --- | --- | --- | --- | --- |
|  | Value | df | Asymp. Sig. (2-sided) | Exact Sig. (2-sided) | Exact Sig. (1-sided) |
| Pearson Chi-Square | 3.186^a^ | 1 | .074 |  |  |
| Continuity Correction^b^ | 2.573 | 1 | .109 |  |  |
| Likelihood Ratio | 3.126 | 1 | .077 |  |  |
| Fisher's Exact Test |  |  |  | .103 | .055 |
| Linear-by-Linear Association | 3.168 | 1 | .075 |  |  |
| N of Valid Cases | 183 |  |  |  |  |

| a. 0 cells (0.0%) have expected count less than 5. The minimum expected count is 16.07. |
| --- |
| b. Computed only for a 2x2 table |

**Cat_prelim * ResIA30_45_recode**

| **Crosstab** | | | | |
| --- | --- | --- | --- | --- |
|  | | | ResIA30_45_recode | Total |
|  |  |  | 1 |  |
| Cat_prelim | categorical | Count | 97 | 97 |
|  |  | % within Cat_prelim | 100.0% | 100.0% |
|  |  | % of Total | 70.8% | 70.8% |
|  | preliminary | Count | 40 | 40 |
|  |  | % within Cat_prelim | 100.0% | 100.0% |
|  |  | % of Total | 29.2% | 29.2% |
| Total | | Count | 137 | 137 |
|  |  | % within Cat_prelim | 100.0% | 100.0% |
|  |  | % of Total | 100.0% | 100.0% |

| **Chi-Square Tests** | |
| --- | --- |
|  | Value |
| Pearson Chi-Square | .^a^ |
| N of Valid Cases | 137 |

| a. No statistics are computed because ResIA30_45_recode is a constant. |
| --- |

**Cat_prelim * ResIA45_60_recode**

| **Crosstab** | | | | | |
| --- | --- | --- | --- | --- | --- |
|  | | | ResIA45_60_recode | | Total |
|  |  |  | 1 | 2 |  |
| Cat_prelim | categorical | Count | 114 | 21 | 135 |
|  |  | % within Cat_prelim | 84.4% | 15.6% | 100.0% |
|  |  | % of Total | 64.0% | 11.8% | 75.8% |
|  | preliminary | Count | 33 | 10 | 43 |
|  |  | % within Cat_prelim | 76.7% | 23.3% | 100.0% |
|  |  | % of Total | 18.5% | 5.6% | 24.2% |
| Total | | Count | 147 | 31 | 178 |
|  |  | % within Cat_prelim | 82.6% | 17.4% | 100.0% |
|  |  | % of Total | 82.6% | 17.4% | 100.0% |

| **Chi-Square Tests** | | | | | |
| --- | --- | --- | --- | --- | --- |
|  | Value | df | Asymp. Sig. (2-sided) | Exact Sig. (2-sided) | Exact Sig. (1-sided) |
| Pearson Chi-Square | 1.344^a^ | 1 | .246 |  |  |
| Continuity Correction^b^ | .862 | 1 | .353 |  |  |
| Likelihood Ratio | 1.278 | 1 | .258 |  |  |
| Fisher's Exact Test |  |  |  | .255 | .175 |
| Linear-by-Linear Association | 1.337 | 1 | .248 |  |  |
| N of Valid Cases | 178 |  |  |  |  |

| a. 0 cells (0.0%) have expected count less than 5. The minimum expected count is 7.49. |
| --- |
| b. Computed only for a 2x2 table |

**Cat_prelim * ResIA_GT60_recode**

| **Crosstab** | | | | | |
| --- | --- | --- | --- | --- | --- |
|  | | | ResIA_GT60_recode | | Total |
|  |  |  | 1 | 2 |  |
| Cat_prelim | categorical | Count | 52 | 70 | 122 |
|  |  | % within Cat_prelim | 42.6% | 57.4% | 100.0% |
|  |  | % of Total | 31.5% | 42.4% | 73.9% |
|  | preliminary | Count | 10 | 33 | 43 |
|  |  | % within Cat_prelim | 23.3% | 76.7% | 100.0% |
|  |  | % of Total | 6.1% | 20.0% | 26.1% |
| Total | | Count | 62 | 103 | 165 |
|  |  | % within Cat_prelim | 37.6% | 62.4% | 100.0% |
|  |  | % of Total | 37.6% | 62.4% | 100.0% |

| **Chi-Square Tests** | | | | | |
| --- | --- | --- | --- | --- | --- |
|  | Value | df | Asymp. Sig. (2-sided) | Exact Sig. (2-sided) | Exact Sig. (1-sided) |
| Pearson Chi-Square | 5.084^a^ | 1 | .024 |  |  |
| Continuity Correction^b^ | 4.292 | 1 | .038 |  |  |
| Likelihood Ratio | 5.339 | 1 | .021 |  |  |
| Fisher's Exact Test |  |  |  | .028 | .018 |
| Linear-by-Linear Association | 5.053 | 1 | .025 |  |  |
| N of Valid Cases | 165 |  |  |  |  |

| a. 0 cells (0.0%) have expected count less than 5. The minimum expected count is 16.16. |
| --- |
| b. Computed only for a 2x2 table |

**Cat_prelim * october_recode**

| **Crosstab** | | | | | |
| --- | --- | --- | --- | --- | --- |
|  | | | october_recode | | Total |
|  |  |  | 1 | 2 |  |
| Cat_prelim | categorical | Count | 51 | 71 | 122 |
|  |  | % within Cat_prelim | 41.8% | 58.2% | 100.0% |
|  |  | % of Total | 31.3% | 43.6% | 74.8% |
|  | preliminary | Count | 24 | 17 | 41 |
|  |  | % within Cat_prelim | 58.5% | 41.5% | 100.0% |
|  |  | % of Total | 14.7% | 10.4% | 25.2% |
| Total | | Count | 75 | 88 | 163 |
|  |  | % within Cat_prelim | 46.0% | 54.0% | 100.0% |
|  |  | % of Total | 46.0% | 54.0% | 100.0% |

| **Chi-Square Tests** | | | | | |
| --- | --- | --- | --- | --- | --- |
|  | Value | df | Asymp. Sig. (2-sided) | Exact Sig. (2-sided) | Exact Sig. (1-sided) |
| Pearson Chi-Square | 3.459^a^ | 1 | .063 |  |  |
| Continuity Correction^b^ | 2.818 | 1 | .093 |  |  |
| Likelihood Ratio | 3.457 | 1 | .063 |  |  |
| Fisher's Exact Test |  |  |  | .072 | .047 |
| Linear-by-Linear Association | 3.438 | 1 | .064 |  |  |
| N of Valid Cases | 163 |  |  |  |  |

| a. 0 cells (0.0%) have expected count less than 5. The minimum expected count is 18.87. |
| --- |
| b. Computed only for a 2x2 table |

**Cat_prelim * november_recode**

| **Crosstab** | | | | | |
| --- | --- | --- | --- | --- | --- |
|  | | | november_recode | | Total |
|  |  |  | 1 | 2 |  |
| Cat_prelim | categorical | Count | 147 | 5 | 152 |
|  |  | % within Cat_prelim | 96.7% | 3.3% | 100.0% |
|  |  | % of Total | 73.9% | 2.5% | 76.4% |
|  | preliminary | Count | 41 | 6 | 47 |
|  |  | % within Cat_prelim | 87.2% | 12.8% | 100.0% |
|  |  | % of Total | 20.6% | 3.0% | 23.6% |
| Total | | Count | 188 | 11 | 199 |
|  |  | % within Cat_prelim | 94.5% | 5.5% | 100.0% |
|  |  | % of Total | 94.5% | 5.5% | 100.0% |

| **Chi-Square Tests** | | | | | |
| --- | --- | --- | --- | --- | --- |
|  | Value | df | Asymp. Sig. (2-sided) | Exact Sig. (2-sided) | Exact Sig. (1-sided) |
| Pearson Chi-Square | 6.174^a^ | 1 | .013 |  |  |
| Continuity Correction^b^ | 4.492 | 1 | .034 |  |  |
| Likelihood Ratio | 5.201 | 1 | .023 |  |  |
| Fisher's Exact Test |  |  |  | .023 | .023 |
| Linear-by-Linear Association | 6.143 | 1 | .013 |  |  |
| N of Valid Cases | 199 |  |  |  |  |

| a. 1 cells (25.0%) have expected count less than 5. The minimum expected count is 2.60. |
| --- |
| b. Computed only for a 2x2 table |

**Cat_prelim * december_recode**

| **Crosstab** | | | | | |
| --- | --- | --- | --- | --- | --- |
|  | | | december_recode | | Total |
|  |  |  | 1 | 2 |  |
| Cat_prelim | categorical | Count | 107 | 3 | 110 |
|  |  | % within Cat_prelim | 97.3% | 2.7% | 100.0% |
|  |  | % of Total | 74.3% | 2.1% | 76.4% |
|  | preliminary | Count | 29 | 5 | 34 |
|  |  | % within Cat_prelim | 85.3% | 14.7% | 100.0% |
|  |  | % of Total | 20.1% | 3.5% | 23.6% |
| Total | | Count | 136 | 8 | 144 |
|  |  | % within Cat_prelim | 94.4% | 5.6% | 100.0% |
|  |  | % of Total | 94.4% | 5.6% | 100.0% |

| **Chi-Square Tests** | | | | | |
| --- | --- | --- | --- | --- | --- |
|  | Value | df | Asymp. Sig. (2-sided) | Exact Sig. (2-sided) | Exact Sig. (1-sided) |
| Pearson Chi-Square | 7.103^a^ | 1 | .008 |  |  |
| Continuity Correction^b^ | 5.003 | 1 | .025 |  |  |
| Likelihood Ratio | 5.869 | 1 | .015 |  |  |
| Fisher's Exact Test |  |  |  | .018 | .018 |
| Linear-by-Linear Association | 7.053 | 1 | .008 |  |  |
| N of Valid Cases | 144 |  |  |  |  |

| a. 1 cells (25.0%) have expected count less than 5. The minimum expected count is 1.89. |
| --- |
| b. Computed only for a 2x2 table |

**Cat_prelim * january_recode**

| **Crosstab** | | | | | |
| --- | --- | --- | --- | --- | --- |
|  | | | january_recode | | Total |
|  |  |  | 1 | 2 |  |
| Cat_prelim | categorical | Count | 14 | 87 | 101 |
|  |  | % within Cat_prelim | 13.9% | 86.1% | 100.0% |
|  |  | % of Total | 9.8% | 60.8% | 70.6% |
|  | preliminary | Count | 11 | 31 | 42 |
|  |  | % within Cat_prelim | 26.2% | 73.8% | 100.0% |
|  |  | % of Total | 7.7% | 21.7% | 29.4% |
| Total | | Count | 25 | 118 | 143 |
|  |  | % within Cat_prelim | 17.5% | 82.5% | 100.0% |
|  |  | % of Total | 17.5% | 82.5% | 100.0% |

| **Chi-Square Tests** | | | | | |
| --- | --- | --- | --- | --- | --- |
|  | Value | df | Asymp. Sig. (2-sided) | Exact Sig. (2-sided) | Exact Sig. (1-sided) |
| Pearson Chi-Square | 3.126^a^ | 1 | .077 |  |  |
| Continuity Correction^b^ | 2.329 | 1 | .127 |  |  |
| Likelihood Ratio | 2.952 | 1 | .086 |  |  |
| Fisher's Exact Test |  |  |  | .093 | .066 |
| Linear-by-Linear Association | 3.104 | 1 | .078 |  |  |
| N of Valid Cases | 143 |  |  |  |  |

| a. 0 cells (0.0%) have expected count less than 5. The minimum expected count is 7.34. |
| --- |
| b. Computed only for a 2x2 table |

**Cat_prelim * february_recode**

| **Crosstab** | | | | | |
| --- | --- | --- | --- | --- | --- |
|  | | | february_recode | | Total |
|  |  |  | 1 | 2 |  |
| Cat_prelim | categorical | Count | 3 | 156 | 159 |
|  |  | % within Cat_prelim | 1.9% | 98.1% | 100.0% |
|  |  | % of Total | 1.4% | 73.9% | 75.4% |
|  | preliminary | Count | 3 | 49 | 52 |
|  |  | % within Cat_prelim | 5.8% | 94.2% | 100.0% |
|  |  | % of Total | 1.4% | 23.2% | 24.6% |
| Total | | Count | 6 | 205 | 211 |
|  |  | % within Cat_prelim | 2.8% | 97.2% | 100.0% |
|  |  | % of Total | 2.8% | 97.2% | 100.0% |

| **Chi-Square Tests** | | | | | |
| --- | --- | --- | --- | --- | --- |
|  | Value | df | Asymp. Sig. (2-sided) | Exact Sig. (2-sided) | Exact Sig. (1-sided) |
| Pearson Chi-Square | 2.138^a^ | 1 | .144 |  |  |
| Continuity Correction^b^ | .964 | 1 | .326 |  |  |
| Likelihood Ratio | 1.845 | 1 | .174 |  |  |
| Fisher's Exact Test |  |  |  | .161 | .161 |
| Linear-by-Linear Association | 2.128 | 1 | .145 |  |  |
| N of Valid Cases | 211 |  |  |  |  |

| a. 2 cells (50.0%) have expected count less than 5. The minimum expected count is 1.48. |
| --- |
| b. Computed only for a 2x2 table |

**Cat_prelim * overview_recode**

| **Crosstab** | | | | | |
| --- | --- | --- | --- | --- | --- |
|  | | | overview_recode | | Total |
|  |  |  | 1 | 2 |  |
| Cat_prelim | categorical | Count | 126 | 16 | 142 |
|  |  | % within Cat_prelim | 88.7% | 11.3% | 100.0% |
|  |  | % of Total | 67.7% | 8.6% | 76.3% |
|  | preliminary | Count | 42 | 2 | 44 |
|  |  | % within Cat_prelim | 95.5% | 4.5% | 100.0% |
|  |  | % of Total | 22.6% | 1.1% | 23.7% |
| Total | | Count | 168 | 18 | 186 |
|  |  | % within Cat_prelim | 90.3% | 9.7% | 100.0% |
|  |  | % of Total | 90.3% | 9.7% | 100.0% |

| **Chi-Square Tests** | | | | | |
| --- | --- | --- | --- | --- | --- |
|  | Value | df | Asymp. Sig. (2-sided) | Exact Sig. (2-sided) | Exact Sig. (1-sided) |
| Pearson Chi-Square | 1.737^a^ | 1 | .188 |  |  |
| Continuity Correction^b^ | 1.053 | 1 | .305 |  |  |
| Likelihood Ratio | 2.012 | 1 | .156 |  |  |
| Fisher's Exact Test |  |  |  | .250 | .152 |
| Linear-by-Linear Association | 1.727 | 1 | .189 |  |  |
| N of Valid Cases | 186 |  |  |  |  |

| a. 1 cells (25.0%) have expected count less than 5. The minimum expected count is 4.26. |
| --- |
| b. Computed only for a 2x2 table |

**Cat_prelim * interview_recode**

| **Crosstab** | | | | | |
| --- | --- | --- | --- | --- | --- |
|  | | | interview_recode | | Total |
|  |  |  | 1 | 2 |  |
| Cat_prelim | categorical | Count | 128 | 14 | 142 |
|  |  | % within Cat_prelim | 90.1% | 9.9% | 100.0% |
|  |  | % of Total | 68.1% | 7.4% | 75.5% |
|  | preliminary | Count | 44 | 2 | 46 |
|  |  | % within Cat_prelim | 95.7% | 4.3% | 100.0% |
|  |  | % of Total | 23.4% | 1.1% | 24.5% |
| Total | | Count | 172 | 16 | 188 |
|  |  | % within Cat_prelim | 91.5% | 8.5% | 100.0% |
|  |  | % of Total | 91.5% | 8.5% | 100.0% |

| **Chi-Square Tests** | | | | | |
| --- | --- | --- | --- | --- | --- |
|  | Value | df | Asymp. Sig. (2-sided) | Exact Sig. (2-sided) | Exact Sig. (1-sided) |
| Pearson Chi-Square | 1.355^a^ | 1 | .244 |  |  |
| Continuity Correction^b^ | .740 | 1 | .390 |  |  |
| Likelihood Ratio | 1.546 | 1 | .214 |  |  |
| Fisher's Exact Test |  |  |  | .365 | .199 |
| Linear-by-Linear Association | 1.348 | 1 | .246 |  |  |
| N of Valid Cases | 188 |  |  |  |  |

| a. 1 cells (25.0%) have expected count less than 5. The minimum expected count is 3.91. |
| --- |
| b. Computed only for a 2x2 table |

**Cat_prelim * tour_recode**

| **Crosstab** | | | | | |
| --- | --- | --- | --- | --- | --- |
|  | | | tour_recode | | Total |
|  |  |  | 1 | 2 |  |
| Cat_prelim | categorical | Count | 6 | 120 | 126 |
|  |  | % within Cat_prelim | 4.8% | 95.2% | 100.0% |
|  |  | % of Total | 3.5% | 69.4% | 72.8% |
|  | preliminary | Count | 1 | 46 | 47 |
|  |  | % within Cat_prelim | 2.1% | 97.9% | 100.0% |
|  |  | % of Total | 0.6% | 26.6% | 27.2% |
| Total | | Count | 7 | 166 | 173 |
|  |  | % within Cat_prelim | 4.0% | 96.0% | 100.0% |
|  |  | % of Total | 4.0% | 96.0% | 100.0% |

| **Chi-Square Tests** | | | | | |
| --- | --- | --- | --- | --- | --- |
|  | Value | df | Asymp. Sig. (2-sided) | Exact Sig. (2-sided) | Exact Sig. (1-sided) |
| Pearson Chi-Square | .612^a^ | 1 | .434 |  |  |
| Continuity Correction^b^ | .121 | 1 | .727 |  |  |
| Likelihood Ratio | .693 | 1 | .405 |  |  |
| Fisher's Exact Test |  |  |  | .676 | .388 |
| Linear-by-Linear Association | .608 | 1 | .435 |  |  |
| N of Valid Cases | 173 |  |  |  |  |

| a. 1 cells (25.0%) have expected count less than 5. The minimum expected count is 1.90. |
| --- |
| b. Computed only for a 2x2 table |

**Cat_prelim * morning_report_recode**

| **Crosstab** | | | | | |
| --- | --- | --- | --- | --- | --- |
|  | | | morning_report_recode | | Total |
|  |  |  | 1 | 2 |  |
| Cat_prelim | categorical | Count | 19 | 113 | 132 |
|  |  | % within Cat_prelim | 14.4% | 85.6% | 100.0% |
|  |  | % of Total | 10.4% | 62.1% | 72.5% |
|  | preliminary | Count | 1 | 49 | 50 |
|  |  | % within Cat_prelim | 2.0% | 98.0% | 100.0% |
|  |  | % of Total | 0.5% | 26.9% | 27.5% |
| Total | | Count | 20 | 162 | 182 |
|  |  | % within Cat_prelim | 11.0% | 89.0% | 100.0% |
|  |  | % of Total | 11.0% | 89.0% | 100.0% |

| **Chi-Square Tests** | | | | | |
| --- | --- | --- | --- | --- | --- |
|  | Value | df | Asymp. Sig. (2-sided) | Exact Sig. (2-sided) | Exact Sig. (1-sided) |
| Pearson Chi-Square | 5.695^a^ | 1 | .017 |  |  |
| Continuity Correction^b^ | 4.498 | 1 | .034 |  |  |
| Likelihood Ratio | 7.463 | 1 | .006 |  |  |
| Fisher's Exact Test |  |  |  | .016 | .010 |
| Linear-by-Linear Association | 5.664 | 1 | .017 |  |  |
| N of Valid Cases | 182 |  |  |  |  |

| a. 0 cells (0.0%) have expected count less than 5. The minimum expected count is 5.49. |
| --- |
| b. Computed only for a 2x2 table |

**Cat_prelim * lunch_recode**

| **Crosstab** | | | | | |
| --- | --- | --- | --- | --- | --- |
|  | | | lunch_recode | | Total |
|  |  |  | 1 | 2 |  |
| Cat_prelim | categorical | Count | 43 | 60 | 103 |
|  |  | % within Cat_prelim | 41.7% | 58.3% | 100.0% |
|  |  | % of Total | 32.6% | 45.5% | 78.0% |
|  | preliminary | Count | 20 | 9 | 29 |
|  |  | % within Cat_prelim | 69.0% | 31.0% | 100.0% |
|  |  | % of Total | 15.2% | 6.8% | 22.0% |
| Total | | Count | 63 | 69 | 132 |
|  |  | % within Cat_prelim | 47.7% | 52.3% | 100.0% |
|  |  | % of Total | 47.7% | 52.3% | 100.0% |

| **Chi-Square Tests** | | | | | |
| --- | --- | --- | --- | --- | --- |
|  | Value | df | Asymp. Sig. (2-sided) | Exact Sig. (2-sided) | Exact Sig. (1-sided) |
| Pearson Chi-Square | 6.719^a^ | 1 | .010 |  |  |
| Continuity Correction^b^ | 5.673 | 1 | .017 |  |  |
| Likelihood Ratio | 6.825 | 1 | .009 |  |  |
| Fisher's Exact Test |  |  |  | .012 | .008 |
| Linear-by-Linear Association | 6.668 | 1 | .010 |  |  |
| N of Valid Cases | 132 |  |  |  |  |

| a. 0 cells (0.0%) have expected count less than 5. The minimum expected count is 13.84. |
| --- |
| b. Computed only for a 2x2 table |
